# Discovery of Trace Amine-Associated Receptor 1 (TAAR1) Agonist 2-(5-(4′-Chloro-[1,1′-biphenyl]-4-yl)-4*H*-1,2,4-triazol-3-yl)ethan-1-amine (LK00764) for the Treatment of Psychotic Disorders

**DOI:** 10.3390/biom12111650

**Published:** 2022-11-07

**Authors:** Mikhail Krasavin, Alexey Lukin, Ilya Sukhanov, Andrey S. Gerasimov, Savelii Kuvarzin, Evgeniya V. Efimova, Mariia Dorofeikova, Anna Nichugovskaya, Andrey Matveev, Kirill Onokhin, Konstantin Zakharov, Maxim Gureev, Raul R. Gainetdinov

**Affiliations:** 1 Department of Medicinal Chemistry, Institute of Chemistry, Saint Petersburg State University, 199034 Saint Petersburg, Russia; 2 Institute for Medicine and Life Sciences, Immanuel Kant Baltic Federal University, 236041 Kaliningrad, Russia; 3 Lomonosov Institute of Fine Chemical Technologies, MIREA—Russian Technological University, 119454 Moscow, Russia; 4 Valdman Institute of Pharmacology, Pavlov First Saint Peterburg State Medical University, 197022 Saint Petersburg, Russia; 5 Institute of Translational Biomedicine, Saint Petersburg State University, 199034 Saint Petersburg, Russia; 6 Accellena Research and Development Inc., 88A Sredniy pr. V.O., 199106 Saint Petersburg, Russia; 7 Center of Bio- and Chemoinformatics, I.M. Sechenov First Moscow State Medical University, 119991 Moscow, Russia

**Keywords:** schizophrenia, trace amine-associated receptor 1, agonism, 1,2,4-triazoles, dopamine transporter knockout rats, dopamine, MK-801-induced hyperactivity, spontaneous activity, locomotor hyperactivity, stress-induced hyperthermia

## Abstract

A focused in-house library of about 1000 compounds comprising various heterocyclic motifs in combination with structural fragments similar to β-phenylethylamine or tyramine was screened for the agonistic activity towards trace amine-associated receptor 1 (TAAR1). The screening yielded two closely related hits displaying EC50 values in the upper submicromolar range. Extensive analog synthesis and testing for TAAR1 agonism in a BRET-based cellular assay identified compound **62** (LK00764) with EC_50_ = 4.0 nM. The compound demonstrated notable efficacy in such schizophrenia-related in vivo tests as MK-801-induced hyperactivity and spontaneous activity in rats, locomotor hyperactivity of dopamine transporter knockout (DAT-KO) rats, and stress-induced hyperthermia (i.p. administration). Further preclinical studies are necessary to evaluate efficacy, safety and tolerability of this potent TAAR1 agonist for the potential development of this compound as a new pharmacotherapy option for schizophrenia and other psychiatric disorders.

## 1. Introduction

Schizophrenia is a chronic debilitating psychiatric disorder affecting about 1% of the human population worldwide. Schizophrenia is characterized by a complex set of abnormal mental manifestations, such as positive and negative symptoms as well as cognitive deficits [1]. Antipsychotics, that have commonly been used for the treatment of schizophrenia symptoms since their discovery in the 1950s, are categorized as typical or atypical. All clinically effective antipsychotics have a common mechanism of action involving the blockade of D_2_ dopamine receptors; however, the typical ones are more potent dopamine D_2_ receptor antagonists and, thus, cause more pronounced side effects such as hyperprolactinemia and extrapyramidal symptoms (EPS). Atypical antipsychotic drugs, in addition to the blockade of dopamine D_2_ receptors, also display antagonism at serotonin 5-HT_2A_ receptors and are often associated with an increased risk for metabolic symptoms such as weight gain and diabetes [2]. While both typical and atypical antipsychotics may effectively treat psychosis and other positive symptoms of schizophrenia, they are less effective at counteracting negative symptoms and, essentially, not effective regarding cognitive deficits. Thus, finding new pharmacotherapies for schizophrenia—which would be effective not only at positive symptoms but also at negative symptoms and cognitive deficits and would be devoid of significant side-effects—remains an important area of unmet need in the treatment of schizophrenia [3].

Over the last 60 years, there were many attempts to develop novel therapies for schizophrenia that exert antipsychotic action without blocking dopamine D_2_ receptors; however, essentially, all these approaches failed in the clinical trials. Recently, a new promising molecular target for the treatment of schizophrenia—trace amine-associated receptor 1 (TAAR1)—has been identified. TAAR1 is a member of a relatively recently discovered family of mammalian G protein-coupled receptors called TAARs, which are activated by trace amines [4,5]. Biogenic trace amines are structurally and functionally related to classical monoaminergic neurotransmitters, such as dopamine and serotonin, but are present in much lower concentrations in the brain. In humans, six functional TAARs have been discovered [6]. The best-characterized member of the TAAR family is TAAR1 [6]. TAAR1 is widely expressed throughout the brain and has been found in areas that are associated with the emergence of schizophrenia symptoms, including the frontal cortex as well as the ventral tegmental area and dorsal raphe nucleus, which produce dopamine and serotonin, respectively. Moreover, preclinical studies indicate that TAAR1 can modulate the activity of several neurotransmitter systems (such as dopamine, serotonin, and glutamate), which dysregulation is believed to be involved in the pathogenesis of schizophrenia. Preclinical studies have shown that TAAR1 agonists produce antipsychotic-like effects in several animal models [7].

Currently, two substances with TAAR1 agonistic activity, Ulotaront (SEP-363856, SEP-856, Sunovion Pharmaceuticals Inc.) and Ralmitaront (RG-7906, RO-6889450, F. Hoffmann-La Roche AG), are undergoing clinical trials for the treatment of schizophrenia, schizoaffective disorder, and Parkinson’s disease psychosis [6]. While the selective partial TAAR1 agonist Ralmitaront is currently being tested in Phase 2 clinical trials, Ulotaront (TAAR1 agonist with serotonin 5-HT_1A_ agonist activity) has entered Phase 3 clinical studies to evaluate the treatment of adults and adolescents with schizophrenia [8,9]. Results of Phase 2 studies with Ulotaront showed that this drug significantly reduced both positive and negative symptoms of schizophrenia, with a safety and tolerability profile comparable to placebo without significantly increasing weight, elevating prolactin or glucose, or inducing EPS. Based on such a promising therapeutic and safety profile in clinical trials, Ulotaront was awarded a Breakthrough Therapy designation by the US Food and Drug Administration (FDA). Since Ulotaront lacks an effect on dopamine D_2_ receptors [8], it has emerged as the first antipsychotic compound effective clinically in schizophrenia without blocking D_2_ dopamine receptors [9]. Additionally, the results of preclinical studies point to the possibility that TAAR1 agonists may have antianxiety [10], antidepressant [10,11], antiaddictive [12], and anticompulsive [13] effects.

**Figure 1:**
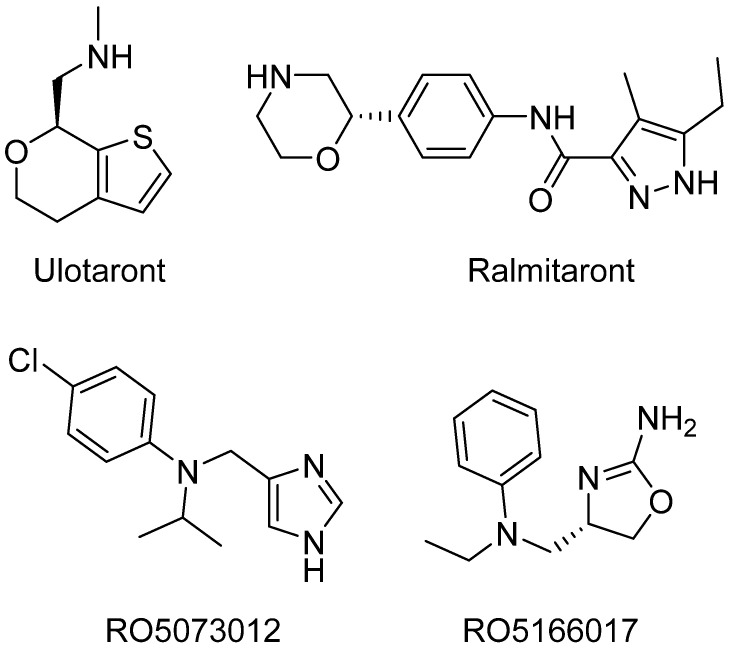
Advanced TAAR1 agonists at various stages of investigation: Ulotaront (phase III clinical trials), Ralmitaront (phase II clinical trials), RO5073012 (preclinical investigation), and RO5166017 (preclinical investigation).

Despite TAAR1 being the hot issue in psychopharmacology, a very limited number of potent and selective TAAR1 ligands are being investigated. Moreover, all preclinically used selective TAAR1 agonists chemically belong either to imidazole (e.g., RO5073012) [14] or to 2-aminooxazoline (e.g., RO5166017) [15] chemical classes (Figure 1). Although other chemical classes, such as biguanides derivates [16], have been identified as TAAR1 agonists, screening of those compounds has been limited to in silico and in vitro analysis. The present study was aimed at (1) identifying novel TAAR1 agonists, chemically distinct from known chemical classes, and (2) testing the compounds found to be active at the TAAR1 receptor for their effects in vivo using animal models relevant to schizophrenia. 

## 2. Materials and Methods

### 2.1. General Experimental Procedures

All reactions were conducted in oven-dried glassware in an atmosphere of nitrogen. Melting points were measured with a Buchi B-520 melting point apparatus and were not corrected. Analytical thin-layer chromatography was carried out on Silufol UV-254 silica gel plates using appropriate mixtures of ethyl acetate and hexane. Compounds were visualized with short-wavelength UV light. The ^1^H nuclear magnetic resonance (NMR) and ^13^C NMR spectra (see Supporting Information) were recorded on Bruker MSL-300 spectrometers in dimethyl sulfoxide (DMSO)-*d*_6_ or CDCl_3_ using tetramethylsilane as an internal standard. Mass spectra were recorded using a Shimadzu LCMS-2020 system with ESI. All reagents and solvents were obtained from commercial sources and used without purification.

### 2.2. General Procedure 1—Synthesis of Compounds ***6***–***57***

Compound **4** (1.2 equiv.) was dissolved in dry methanol (5 mL/mmol) and treated with acyl hydrazide **3** (1.0 equiv.). The reaction mixture was stirred at room temperature for 24–48 h while the progress of the reaction was monitored by TLC analysis (2% MeOH in chloroform). The volatiles were removed on a rotary evaporator and the residue was transferred into a flask equipped with a Vigreux column and melted neat at 180–200 °C for 1–2 h. On cooling, the residue was purified by column chromatography on silica gel using 2% MeOH in chloroform as an eluent. Fractions containing the triazole product were pooled, concentrated in vacuo, and the residue was treated with 4M solution of HCl in 1,4-dioxane (1 h, room temperature). Following concentration on a rotary evaporator, the crystalline residue was triturated with ether, the solids were filtered off, washed with more ether, and air-tried to provide an analytically pure title compound as crystalline residue.

#### 2.2.1. 2-(5-(4-Methoxyphenyl)-4*H*-1,2,4-triazol-3-yl)ethan-1-amine Hydrochloride (**6**)

Yield 0.21 g (60%). M.p. 248–250 °C. ^1^H NMR (300 MHz, DMSO-*d_6_*) δ 8.28 (s, 3H), 8.12 (d, J = 8.5 Hz, 2H), 7.12 (d, J = 8.6 Hz, 2H), 3.83 (s, 3H), 3.35–3.26 (m, 2H), 3.24–3.17 (m, 2H). ^13^C NMR (75 MHz, DMSO-*d_6_*) δ 161.5, 154.8, 154.5, 128.5, 118.0, 114.6, 55.5, 36.6, 24.1. HRMS (ESI) calcd for C_11_H_15_N_4_O [M + H^+^] 219.1240 Da, found 219.1240 Da.

#### 2.2.2. 2-(5-(3-Methoxyphenyl)-4*H*-1,2,4-triazol-3-yl)ethan-1-amine Hydrochloride (**7**)

Yield 0.92 g (48%). M.p. 235–236 °C. ^1^H NMR (300 MHz, DMSO-*d_6_*) δ 8.27 (br.s, 3H), 7.71–7.66 (m, 2H), 7.44 (t, J = 8.0 Hz, 1H), 7.11–7.05 (m, 1H), 3.82 (s, 3H), 3.33–3.24 (m, 2H), 3.22–3.14 (m, 2H). ^13^C NMR (75 MHz, DMSO-*d_6_*) δ 159.6, 156.1, 155.6, 130.2, 128.7, 118.6, 116.3, 111.4, 55.4, 36.8, 24.3. HRMS (ESI) calcd for C_11_H_15_N_4_O [M + H^+^] 219.1240 Da, found 219.1240 Da

#### 2.2.3. 2-(5-(3-[Trifluoromethyl]phenyl)-4*H*-1,2,4-triazol-3-yl)ethan-1-amine Hydrochloride (**8**)

Yield 0.11 g (22%). M.p. 242–244 °C. ^1^H NMR (300 MHz, DMSO-*d_6_*) δ 8.35 (br.s, 5H), 7.89–7.67 (m, 2H), 3.35–3.14 (m, 4H). ^13^C NMR (75 MHz, DMSO-*d_6_*) δ 156.9, 155.6, 130.4, 130.3, 130.0 (q, J = 31.8 Hz), 129.9, 126.2 (q, J = 3.5 Hz), 124.1 (q, J = 272.4 Hz), 122.4 (q, J = 3.8 Hz), 36.8, 24.2. HRMS (ESI) calcd for C_11_H_11_F_3_N_4_ [M + H^+^] 257.1009 Da, found 1009 Da.

#### 2.2.4. 2-(5-(4-[Trifluoromethoxy]phenyl)-4*H*-1,2,4-triazol-3-yl)ethan-1-amine Hydrochloride (**9**)

Yield 0.054 g (12%). M.p. 253–254 °C. ^1^H NMR (300 MHz, DMSO-*d_6_*) δ 8.28 (br.s, 3H), 8.20–8.14 (m, 2H), 7.53–7.47 (m, 2H), 3.30–3.21 (m, 2H), 3.20–3.12 (m, 2H). ^13^C NMR (75 MHz, DMSO-*d_6_*) δ 157.1, 155.9, 149.1 (q, J = 1.6 Hz), 128.6, 128.1, 121.5, 118.4 (q, J = 256.7 Hz), 36.9, 24.4. HRMS (ESI) calcd for C_11_H_11_F_3_N_4_O [M + H^+^] 273.0958 Da, found 273.0958 Da.

#### 2.2.5. 2-(5-(Pyridin-4-yl)-4*H*-1,2,4-triazol-3-yl)ethan-1-amine Hydrochloride (**10**)

Yield 0.06 g (18%). M.p. 257–258 ℃. ^1^H NMR (300 MHz, DMSO-*d_6_*) δ 8.95 (d, J = 6.6 Hz, 2H), 8.46 (d, J = 6.6 Hz, 2H), 8.31 (s, 1H), 3.34–3.18 (m, 4H). ^13^C NMR (75 MHz, DMSO-*d_6_*) δ 156.68, 156.08, 151.10, 145.15, 143.38, 124.02, 122.41, 36.63, 23.99. HRMS (ESI) calcd for C_9_H_11_N [M + H^+^] 190.1087 Da, found 190.1087 Da.

#### 2.2.6. 2-(5-(3,4-Dimethoxyphenyl)-4*H*-1,2,4-triazol-3-yl)ethan-1-amine Hydrochloride (**11**)

Yield 0.45 g (26%). M.p. 209–210 °C. ^1^H NMR (300 MHz, DMSO-*d_6_*) δ 8.07 (br.s, 3H), 7.6–7.59 (m, 2H), 7.1–7.07 (m, 1H), 3.84 (s, 3H), 3.81 (s, 3H), 3.29–3.17 (m, 2H), 3.13–3.03 (m, 2H). ^13^C NMR (75 MHz, DMSO-*d_6_*) δ 154.9, 154.6, 150.9, 148.8, 119.7, 117.7, 111.8, 109.7, 55.7, 55.5, 36.5, 24.0. HRMS (ESI) calcd for C_12_H_17_N_4_O_2_ [M + H^+^] 249.1346 Da, found 249.1346 Da.

#### 2.2.7. 2-(5-(4-Bromo-3-methylphenyl)-4*H*-1,2,4-triazol-3-yl)ethan-1-amine Hydrochloride (**12**)

Yield 0.139 g (36%). M.p. 251–252 °C. ^1^H NMR (300 MHz, DMSO-*d_6_*) δ 8.18 (br.s, 3H), 8.03–8.00 (m, 1H), 7.80–7.75 (m, 1H), 7.72–7.68 (m, 1H), 3.30–3.18 (m, 2H), 3.17–3.08 (m, 2H), 2.41 (s, 3H). ^13^C NMR (75 MHz, DMSO-*d_6_*) δ 156.9, 155.8, 138.2, 132.9, 128.6, 128.2, 125.8, 125.5, 37.0, 24.4, 22.6. HRMS (ESI) calcd for C_11_H_14_BrN_4_ [M + H^+^] 281.0396 Da, found 281.0396 Da.

#### 2.2.8. 2-(5-(Diphenylmethyl)-4*H*-1,2,4-triazol-3-yl)ethan-1-amine Hydrochloride (**13**)

Yield 0.15 g (42%). M.p. 236–237 °C. ^1^H NMR (300 MHz, DMSO-*d_6_*) δ 8.16 (br.s, 3H), 7.39–7.19 (m, 10H), 5.65 (s, 1H), 3.24–3.13 (m, 2H), 3.10–3.03 (m, 2H). ^13^C NMR (75 MHz, DMSO-*d_6_*) δ 158.9, 154.6, 140.4, 128.4, 128.4, 126.8, 48.2, 36.3, 23.9. HRMS (ESI) calcd for C_17_H_19_N_4_ [M + H^+^] 279.1604 Da, found 279.1604 Da.

#### 2.2.9. 2-(5-(4-Nitrophenyl)-4*H*-1,2,4-triazol-3-yl)ethan-1-amine Hydrochloride (**14**)

Yield 1.959 g (40%). M.p. 239–240 °C. ^1^H NMR (300 MHz, DMSO-*d_6_*) δ 8.39–8.25 (m, 4H), 8.20 (br.s, 3H), 3.31–3.21 (m, 2H), 3.19–3.11 (m, 2H). ^13^C NMR (75 MHz, DMSO-*d_6_*) δ 158.0, 155.9, 147.0, 136.4, 127.1, 124.4, 37.0, 24.3. HRMS (ESI) calcd for C_10_H_12_N_5_O_2_ [M + H^+^] 234.0986 Da, found 234.0986 Da.

#### 2.2.10. 2-(5-(4-[Trifluoromethyl]phenyl)-4*H*-1,2,4-triazol-3-yl)ethan-1-amine Hydrochloride (**15**)

Yield 0.629 g (46%). M.p. 254–255 °C. ^1^H NMR (300 MHz, DMSO-*d_6_*) δ 8.28–8.19 (m, 5H), 7.8–7.82 (m, 2H), 3.33–3.21 (m, 2H), 3.19–3.11 (m, 2H). ^13^C NMR (75 MHz, DMSO-*d_6_*) δ 157.8, 155.9, 133.8, 129.4 (q, J = 31.9 Hz), 126.6, 125.9 (q, J = 3.7 Hz), 124.2 (q, J = 272.1 Hz), 37.0, 24.4. HRMS (ESI) calcd for C_11_H_12_F_3_N_4_ [M + H^+^] 257.1009 Da, found 257.1009 Da.

#### 2.2.11. 2-(5-(Thiophen-2-yl)-4*H*-1,2,4-triazol-3-yl)ethan-1-amine Hydrochloride (**16**)

Yield 0.164 g (24%). M.p. 248–250 °C. ^1^H NMR (300 MHz, DMSO-*d_6_*) δ 8.17 (br.s, 3H), 7.71–7.62 (m, 2H), 7.19–7.14 (m, 1H), 3.27–3.15 (m, 2H), 3.13–3.04 (m, 2H). ^13^C NMR (75 MHz, DMSO-*d_6_*) δ 155.1, 153.3, 130.8, 127.6, 127.3, 126.1, 36.3, 23.7. HRMS (ESI) calcd for C_8_H_11_N_4_S [M + H^+^] 195.0699 Da, found 195.0699 Da.

#### 2.2.12. 2-(5-(Phenoxymethyl)-4*H*-1,2,4-triazol-3-yl)ethan-1-amine Hydrochloride (**17**)

Yield 0.283 g (14%). M.p. 206–207 °C. ^1^H NMR (300 MHz, DMSO-*d_6_*) δ 8.26 (br.s, 3H), 7.34–7.27 (m, 2H), 7.07–7.01 (m, 2H), 7.00–6.93 (m, 1H), 5.12 (s, 2H), 3.24–3.15 (m, 2H), 3.14–3.08 (m, 2H). ^13^C NMR (75 MHz, DMSO-*d_6_*) δ 157.7, 155.6, 155.3, 129.4, 121.0, 114.5, 61.6, 36.6, 24.1. HRMS (ESI) calcd for C_11_H_15_N_4_O [M + H^+^] 219.1240 Da, found 219.1240 Da.

#### 2.2.13. 2-(5-(3-[Trifluoromethyl]phenyl)-4*H*-1,2,4-triazol-3-yl)ethan-1-amine Hydrochloride (**18**)

Yield 1.25 g (60%). M.p. 288–290 °C. ^1^H NMR (300 MHz, DMSO-*d_6_*) δ 8.37–8.33 (m, 1H), 8.23 (br.s, 3H), 7.77–7.73 (m, 2H), 3.32–3.22 (m, 2H), 3.20–3.12 (m, 2H). ^13^C NMR (75 MHz, DMSO-*d_6_*) δ 155.1, 151.9, 128.3, 128.1, 126.8, 125.9, 36.7, 24.2. HRMS (ESI) calcd for C_8_H_11_N_4_S [M + H^+^] 195.0699 Da, found 195.0699 Da.

#### 2.2.14. 2-(5-(4-(Benzyloxy)phenyl)-4*H*-1,2,4-triazol-3-yl)ethan-1-amine Hydrochloride (**19**)

Yield 4.568g (54%). M.p. 238–240 °C. ^1^H NMR (300 MHz, DMSO-*d_6_*) δ 8.24 (br.s, 3H), 8.10–8.05 (m, 2H), 7.50–7.31 (m, 5H), 7.22–7.17 (m, 2H), 5.19 (s, 2H), 3.33–3.22 (m, 2H), 3.20–3.13 (m, 1H). ^13^C NMR (75 MHz, DMSO-*d_6_*) δ 160.0, 155.3, 155.0, 136.4, 128.2, 128.0, 127.8, 127.7, 118.9, 115.2, 69.3, 36.6, 24.1. HRMS (ESI) calcd for C_17_H_19_N_4_O [M + H^+^] 295.1553Da, found 295.1553 Da.

#### 2.2.15. 2-(5-(3-Bromophenyl)-4*H*-1,2,4-triazol-3-yl)ethan-1-amine Hydrochloride (**20**)

Yield 4.06 g (45%). M.p. 239–241 °C. ^1^H NMR (300 MHz, DMSO-*d_6_*) δ 8.33 (br.s, 3H), 8.24 (s, 1H), 8.07 (d, J = 7.8 Hz, 1H), 7.69–7.64 (m, 1H), 7.51–7.44 (m, 1H), 3.32–3.22 (m, 2H), 3.21–3.14 (m, 2H). ^13^C NMR (75 MHz, DMSO-*d_6_*) δ 156.4, 155.6, 132.5, 131.2, 131.1, 128.6, 125.1, 122.2, 36.8, 24.2. HRMS (ESI) calcd for C_10_H_12_BrN_4_ [M + H^+^] 267.0239 Da, found 267.0239 Da.

#### 2.2.16. 2-(5-(3,5-Dichlorophenyl)-4*H*-1,2,4-triazol-3-yl)ethan-1-amine Hydrochloride (**21**)

Yield 0.102 g (36%). M.p. 222–224 °C. ^1^H NMR (300 MHz, DMSO-*d_6_*) δ 8.31 (br.s, 3H), 8.02 (d, J = 1.8 Hz, 2H), 7.70–7.68 (m, 1H), 3.29–3.20 (m, 2H), 3.19–3.11 (m, 2H). ^13^C NMR (75 MHz, DMSO-*d_6_*) δ 156.6, 155.8, 134.7, 133.3, 128.7, 124.3, 36.8, 24.1. HRMS (ESI) calcd for C_10_H_11_Cl_2_N_4_ [M + H^+^] 257.0355 Da, found 257.0355 Da.

#### 2.2.17. 2-(5-Phenyl-4*H*-1,2,4-triazol-3-yl)ethan-1-amine Hydrochloride (**22**)

Yield 0.04 g (8%). M.p. 250–252 °C. ^1^H NMR (300 MHz, DMSO-*d_6_*) δ 8.17 (br.s, 3H), 8.08–8.02 (m, 2H), 7.55–7.44 (m, 3H), 3.32–3.20 (m, 2H), 3.17–3.09 (m, 2H). ^13^C NMR (75 MHz, DMSO-*d_6_*) δ 156.6, 154.1, 129.9, 129.0, 128.5, 126.1, 37.2, 24.6. HRMS (ESI) calcd for C_10_H_13_N_4_ [M + H^+^] 189.1135 Da, found 189.1135 Da.

#### 2.2.18. 2-(5-(2-Methylphenyl)-4*H*-1,2,4-triazol-3-yl)ethan-1-amine Hydrochloride (**24**)

Yield 0.110 g (22%). M.p. 235–236 °C. ^1^H NMR (300 MHz, DMSO-*d_6_*) δ 8.25 (br.s, 3H), 7.78 (d, J = 7.4 Hz, 1H), 7.43–7.29 (m, 3H), 3.33–3.22 (m, 2H), 3.21–3.14 (m, 2H), 2.53 (s, 3H). ^13^C NMR (75 MHz, DMSO-*d_6_*) δ 157.1, 155.1, 136.6, 131.1, 129.0, 129.2, 127.5, 125.9, 36.8, 30.6, 24.3. HRMS (ESI) calcd for C_11_H_15_N_4_ [M + H^+^] 203.1291 Da, found 203.1291 Da.

#### 2.2.19. 3-(5-(2-Aminoethyl)-4*H*-1,2,4-triazol-3-yl)-N,N-dimethylaniline Hydrochloride (**8**)

Yield 0.022 g (22%). M.p. 179–181 °C. ^1^H NMR (300 MHz, DMSO-*d_6_*) δ 8.42 (br.s, 3H), 8.28 (s, 1H), 7.97 (d, J = 6.9 Hz, 1H), 7.67–7.56 (m, 2H), 3.38–3.21 (m, 4H), 3.12 (s, 6H). ^13^C NMR (75 MHz, DMSO-*d_6_*) δ 156.0, 154.8, 146.0, 130.8, 128.8, 123.4, 120.6, 116.6, 44.2, 36.7, 24.1. HRMS (ESI) calcd for C_12_H_18_N_5_ [M + H^+^] 232.1557 Da, found 232.1557 Da.

#### 2.2.20. 2-(5-(4-Ethoxyphenyl)-4*H*-1,2,4-triazol-3-yl)ethan-1-amine Hydrochloride (**26**)

Yield 0.051 g (44%). M.p. 235–236 °C. ^1^H NMR (300 MHz, DMSO-*d_6_*) δ 8.16 (br.s, 3H), 8.01 (d, J = 8.7 Hz, 2H), 7.06 (d, J = 8.8 Hz, 2H), 4.10 (q, J = 6.9 Hz, 2H), 3.31–3.19 (m, 2H), 3.17–3.08 (m, 2H), 1.34 (t, J = 6.9 Hz, 3H). ^13^C NMR (75 MHz, DMSO-*d_6_*) δ 160.3, 156.8, 156.4, 128.1, 120.1, 115.1, 63.6, 37.3, 24.9, 14.8. HRMS (ESI) calcd for C_12_H_17_N_4_O [M + H^+^] 233.1397 Da, found 233.1397 Da.

#### 2.2.21. 2-(5-(4-Ethylphenyl)-4*H*-1,2,4-triazol-3-yl)ethan-1-amine Hydrochloride (**27**)

Yield 0.01 g (5%). M.p. 119–121 °C. ^1^H NMR (300 MHz, DMSO-*d_6_*) δ 8.21 (s, 3H), 7.99 (d, J = 6.8 Hz, 2H), 7.36 (d, J = 6.6 Hz, 2H), 3.35–3.21 (m, 2H), 3.19–3.09 (m, 2H), 2.66 (q, J = 6.7 Hz, 2H), 1.21 (s, J = 6.7 Hz, 3H). ^13^C NMR (75 MHz, DMSO-*d_6_*) 156.3, 155.8, 146.3, 128.4, 126.4, 124.9, 36.9, 28.1, 24.5, 15.3. HRMS (ESI) calcd for C_12_H_17_N_4_ [M + H^+^] 217.1447 Da, found 217.1447 Da.

#### 2.2.22. 2-(5-(2-Nitrophenyl)-4*H*-1,2,4-triazol-3-yl)ethan-1-amine Hydrochloride (**27**)

Yield 0.111 g (94%). M.p. 169–170 °C. ^1^H NMR (300 MHz, DMSO-*d_6_*) δ 8.36–8.23 (m, 6H), 3.33–3.13 (m, 4H). ^13^C NMR (75 MHz, DMSO-*d_6_*) δ 156.0, 154.8, 148.8, 132.1, 130.4, 130.1, 123.6, 123.3, 36.7, 24.0. HRMS (ESI) calcd for C_10_H_12_N_5_O_2_ [M + H^+^] 234.0986 Da, found 234.0986 Da.

#### 2.2.23. 2-(5-(4-Chlorophenyl)-4*H*-1,2,4-triazol-3-yl)ethan-1-amine Hydrochloride (**28**)

Yield 0.025 g (13%). M.p. 239–241 °C. ^1^H NMR (300 MHz, DMSO-*d_6_*) δ 8.12 (s, 2H), 8.05–8.00 (m, 2H), 7.58–7.53 (m, 2H), 3.29–3.17 (m, 2H), 3.14–3.05 (m, 2H). ^13^C NMR (75 MHz, DMSO-*d_6_*) δ 157.7, 156.4, 134.3, 129.2, 128.3, 127.9, 37.2, 24.6. HRMS (ESI) calcd for C_10_H_12_ClN_4_ [M + H^+^] 223.0745 Da, found 223.0745 Da.

#### 2.2.24. 2-(5-(2-Bromophenyl)-4*H*-1,2,4-triazol-3-yl)ethan-1-amine Hydrochloride (**29**)

Yield 0.044 g (33%). M.p. 194–196 °C. ^1^H NMR (300 MHz, DMSO-*d_6_*) δ 8.25 (s, 3H), 7.79–7.73 (m, 2H), 7.53–7.46 (m, 1H), 7.44–7.38 (m, 1H), 3.29–3.11 (m, 4H). ^13^C NMR (75 MHz, DMSO-*d_6_*) δ 157.4, 155.8, 133.8, 131.9, 131.4, 131.0, 128.0, 121.3, 37.2, 24.6. HRMS (ESI) calcd for C_10_H_12_BrN_4_ [M + H^+^] 267.0240 Da, found 267.0240 Da.

#### 2.2.25. 4-(5-(2-Aminoethyl)-4*H*-1,2,4-triazol-3-yl)-N,N-dimethylaniline (**30**)

Yield 0.017 g (9%). M.p. 244–246 °C. ^1^H NMR (300 MHz, DMSO-*d_6_*) δ 8.20 (s, 3H), 8.02 (d, J = 8.7 Hz, 2H), 6.91 (d, J = 8.1 Hz, 2H), 3.35–3.24 (m, 2H), 3.21–3.12 (m, 2H), 3.03 (s, 6H). ^13^C NMR (75 MHz, DMSO-*d_6_*) δ 154.2, 154.1, 151.8, 128.2, 112.3, 36.6, 25.5, 24.1. HRMS (ESI) calcd for C_12_H_18_N [M + H^+^] 232.1557 Da, found 232.1557 Da.

#### 2.2.26. 2-(5-(3,5-Dimethylphenyl)-4*H*-1,2,4-triazol-3-yl)ethan-1-amine Hydrochloride (**31**)

Yield 0.138 g (41%). M.p. 232–234 °C. ^1^H NMR (300 MHz, DMSO-*d_6_*) δ 8.34 (br.s, 3H), 7.76 (s, 2H), 7.17 (s, 1H), 3.37–3.16 (m, 4H), 2.33 (s, 6H). ^13^C NMR (75 MHz, DMSO-*d_6_*) δ 155.7, 155.2, 138.4, 132.2, 126.5, 124.3, 36.7, 24.2, 21.0. HRMS (ESI) calcd for C_12_H_17_N_4_ [M + H^+^] 217.1448Da, found 217.1448 Da.

#### 2.2.27. 2-(5-(3,4,5-Triethoxyphenyl)-4*H*-1,2,4-triazol-3-yl)ethan-1-amine Hydrochloride (**32**)

Yield 0.071 g (26%). M.p. 253–255 °C. ^1^H NMR (300 MHz, DMSO-*d_6_*) δ 8.22 (br.s, 3H), 7.48 (s, 2H), 4.11 (q, J = 6.8 Hz, 4H), 4.00 (q, J = 7.0 Hz, 2H), 3.34–3.23 (m, 2H), 3.21–3.12 (m, 2H), 1.36 (t, J = 6.9 Hz, 6H), 1.24 (t, J = 7.0 Hz, 3H). ^13^C NMR (75 MHz, DMSO-*d_6_*) δ 156.0, 155.9, 152.8, 138.7, 122.0, 104.8, 68.1, 64.3, 36.9, 24.5, 15.5, 14.8. HRMS (ESI) calcd for C_16_H_25_N_4_O_3_ [M + H^+^] 321.1921 Da, found 321.1921 Da.

#### 2.2.28. 2-(5-(3,5-Dimethoxyphenyl)-4*H*-1,2,4-triazol-3-yl)ethan-1-amine Hydrochloride (**33**)

Yield 0.031 g (15%). M.p. 245–247 °C. ^1^H NMR (300 MHz, DMSO-*d_6_*) δ 8.10 (br.s, 3H), 7.20 (d, J = 2.2 Hz, 2H), 6.59 (t, J = 2.1 Hz, 1H), 3.80 (s, 6H), 3.29–3.17 (m, 2H), 3.12–3.04 (m, 2H). ^13^C NMR (75 MHz, DMSO-*d_6_*) δ 160.8, 157.2, 154.1, 125.2, 143.0, 101.8, 55.5, 37.1, 24.7. HRMS (ESI) calcd for C_12_H_17_N_4_O [M + H^+^] 249.1346 Da, found 249.1346 Da.

#### 2.2.29. 2-(5-(4-Fluorophenyl)-4*H*-1,2,4-triazol-3-yl)ethan-1-amine Hydrochloride (**34**)

Yield 0.028 g (16%). M.p. 253–256 °C. ^1^H NMR (300 MHz, DMSO-*d_6_*) δ 8.25–8.04 (m, 5H), 7.46–7.27 (m, 2H), 3.30–3.19 (m, 2H), 3.17–3.08 (m, 2H). ^13^C NMR (75 MHz, DMSO-*d_6_*) δ 162.93 (d, J = 246.5 Hz), 156.89, 155.93, 128.38 (d, J = 8.7 Hz), 125.56, 115.97 (d, J = 22.0 Hz), 36.94, 24.45. HRMS (ESI) calcd for C_10_H_12_FN_4_ [M + H^+^] 207.1041 Da, found 207.1041 Da.

#### 2.2.30. 2-(5-(Quinolin-6-yl)-4*H*-1,2,4-triazol-3-yl)ethan-1-amine Hydrochloride (**35**)

Yield 0.037 g (18%). M.p. 265–267 °C. ^1^H NMR (300 MHz, DMSO-*d_6_*) δ 9.20 (d, J = 4.8 Hz, 1H), 9.05 (d, J = 7.6 Hz, 1H), 8.89 (s, 1H), 8.64 (d, J = 8.9 Hz, 1H), 8.38 (d, J = 8.8 Hz, 1H), 8.22 (br.s, 3H), 8.00–7.92 (m, 1H), 3.34–3.23 (m, 2H), 3.22–3.15 (m, 2H). ^13^C NMR (75 MHz, DMSO-*d_6_*) δ 158.4, 155.8, 146.6, 144.7, 140.1, 131.0, 130.6, 128.7, 125.6, 123.4, 122.7, 37.0, 24.3. HRMS (ESI) calcd for C_13_H_14_N_5_ [M + H^+^] 240.1244 Da, found 240.1244 Da.

#### 2.2.31. 2-(5-(3-Nitrophenyl)-4*H*-1,2,4-triazol-3-yl)ethan-1-amine Hydrochloride (**36**)

Yield 0.057 g (48%). M.p. 214–216 °C. ^1^H NMR (300 MHz, DMSO-*d_6_*) δ 8.78–8.76 (m, 1H), 8.44 (d, J = 7.8 Hz, 1H), 8.28 (dd, J = 8.2, 1.6 Hz, 1H), 8.18 (br.s, 3H), 7.79 (t, J = 8.0 Hz, 1H), 3.32–3.20 (m, 2H), 3.19–3.11 (m, 2H). ^13^C NMR (75 MHz, DMSO-*d_6_*) δ 157.9, 156.1, 148.5, 132.2, 132.0, 131.0, 124.2, 120.5, 37.2, 24.5. HRMS (ESI) calcd for C_10_H_12_N_5_O_2_ [M + H^+^] 234.0986 Da, found 234.0986 Da.

#### 2.2.32. 2-(5-(3,4-Dimethylphenyl)-4*H*-1,2,4-triazol-3-yl)ethan-1-amine Hydrochloride (**37**)

Yield 0.015 g (8%). M.p. 231–234 °C. ^1^H NMR (300 MHz, DMSO-*d_6_*) 8.14 (br.s, 3H), 7.84 (s, 1H), 7.79–7.73 (m, 1H), 7.27 (d, J = 7.8 Hz, 1H), 3.30–3.18 (m, 2H), 3.14–3.06 (m, 2H), 2.28 (s, 3H), 2.27 (s, 3H). ^13^C NMR (75 MHz, DMSO-*d_6_*) 156.3, 153.5, 138.7, 136.9, 135.1, 130.0, 127.2, 123.7, 37.0, 24.6, 19.5, 19.4 HRMS (ESI) calcd for C_12_H_17_N_4_ [M + H^+^] 217.1448 Da, found 217.1448 Da.

#### 2.2.33. 2-(5-(Pyrazin-2-yl)-4*H*-1,2,4-triazol-3-yl)ethan-1-amine Hydrochloride (**38**)

Yield 0.020 g (21%). M.p. 184–186 °C. ^1^H NMR (300 MHz, DMSO-*d_6_*) δ 9.29 (d, J = 1.3 Hz, 1H), 8.77–8.72 (m, 2H), 8.16 (br.s, 3H), 3.29–3.20 (m, 2H), 3.17–3.10 (m, 2H). ^13^C NMR (75 MHz, DMSO-*d_6_*) δ 157.8, 155.4, 145.5, 144.7, 143.6, 142.7, 37.3, 24.9. HRMS (ESI) calcd for C_8_H_11_N_6_ [M + H^+^] 191.1040 Da, found 191.1040 Da.

#### 2.2.34. 2-(5-(2-[Trifluoromethoxy]phenyl)-4*H*-1,2,4-triazol-3-yl)ethan-1-amine Hydrochloride (**39**)

Yield 0.053 g (39%). M.p. 208–211 °C. ^1^H NMR (300 MHz, DMSO-*d_6_*) δ 8.17–8.05 (m, 3H), 7.62–7.47 (m, 3H), 3.29–3.18 (m, 2H), 3.15–3.08 (m, 2H). ^13^C NMR (75 MHz, DMSO-*d_6_*) δ 155.18, 152.20, 145.18 (q, J = 1.7 Hz), 130.83, 130.47, 127.65, 123.78, 122.00, 119.96 (q, J = 256.5 Hz), 36.80, 24.11. HRMS (ESI) calcd for C_11_H_12_F_3_N_4_O [M + H^+^] 273.0957 Da, found 273.0957 Da.

#### 2.2.35. 2-(5-(4-Butoxyphenyl)-4*H*-1,2,4-triazol-3-yl)ethan-1-amine Hydrochloride (**40**)

Yield 0.019 g (15%). M.p. 205–207 °C. ^1^H NMR (300 MHz, DMSO-*d_6_*) δ 8.32 (br.s, 3H), 8.06 (d, J = 8.7 Hz, 2H), 7.08 (d, J = 8.8 Hz, 2H), 4.04 (t, J = 6.4 Hz, 2H), 3.33–3.12 (m, 4H), 1.76–1.65 (m, 2H), 1.50–1.37 (m, 2H), 0.93 (t, J = 7.4 Hz, 3H). ^13^C NMR (75 MHz, DMSO-*d_6_*) δ 160.4, 155.7, 155.6, 128.1, 119.2, 114.9, 67.4, 36.8, 30.6, 24.4, 18.7, 13.7. HRMS (ESI) calcd for C_14_H_21_N_4_O [M + H^+^] 261.1709 Da, found 261.1709 Da.

#### 2.2.36. 2-(5-(3-Iodophenyl)-4*H*-1,2,4-triazol-3-yl)ethan-1-amine Hydrochloride (**41**)

Yield 0.053 g (39%). M.p. 224–225 °C. ^1^H NMR (300 MHz, DMSO-*d_6_*) δ 8.37 (t, J = 1.6 Hz, 1H), 8.17 (br.s, 3H), 8.06–8.01 (m, 1H), 7.83–7.78 (m, 1H), 7.29 (t, J = 7.8 Hz, 1H), 3.28–3.17 (m, 2H), 3.15–3.07 (m, 2H). ^13^C NMR (75 MHz, DMSO-*d_6_*) δ 157.3, 156.6, 138.4, 134.5, 131.8, 131.4, 125.5, 95.4, 37.3, 24.7. HRMS (ESI) calcd for C_10_H_12_IN_4_ [M + H^+^] 315.0101 Da, found 315.0101 Da.

#### 2.2.37. 2-(5-(4-Iodo-3-methylphenyl)-4*H*-1,2,4-triazol-3-yl)ethan-1-amine Hydrochloride (**42**)

Yield 0.047 g (31%). M.p. 225–227 °C. ^1^H NMR (300 MHz, DMSO-*d_6_*) δ 8.21 (br.s, 3H), 8.00 (d, J = 1.8 Hz, 1H), 7.94 (d, J = 8.2 Hz, 1H), 7.59 (dd, J = 8.2, 2.0 Hz, 1H), 3.30–3.18 (m, 2H), 3.16–3.08 (m, 2H), 2.43 (s, 3H). ^13^C NMR (75 MHz, DMSO-*d_6_*) δ 157.5, 156.3, 141.6, 139.3, 129.5, 127.2, 125.3, 102.9, 37.1, 27.7, 24.6. HRMS (ESI) calcd for C_11_H_14_IN_4_ [M + H^+^] 329.0257 Da, found 329.0257 Da.

#### 2.2.38. 2-(5-(4-[(2-Fluorobenzyl)oxy]phenyl)-4*H*-1,2,4-triazol-3-yl)ethan-1-amine Hydrochloride (**43**)

Yield 0.023 g (15%). M.p. 232–235 °C. ^1^H NMR (300 MHz, DMSO-*d_6_*) δ 8.16 (br.s, 3H), 8.06–8.00 (m, 2H), 7.59 (t, J = 7.0 Hz, 1H), 7.49–7.40 (m, 1H), 7.32–7.17 (m, 4H), 5.22 (s, 2H), 3.31–3.19 (m, 2H), 3.17–3.08 (m, 2H). ^13^C NMR (75 MHz, DMSO-*d_6_*) δ 160.5 (d, J = 246.2 Hz), 159.5, 156.6, 156.4, 130.9 (d, J = 4.0 Hz), 130.6 (d, J = 8.2 Hz), 127.8, 124.6 (d, J = 3.4 Hz), 123.4 (d, J = 14.5 Hz), 120.8, 115.5 (d, J = 21.0 Hz), 115.1, 63.8, 37.1, 24.7. HRMS (ESI) calcd for C_17_H_18_FN_4_O [M + H^+^] 313.1459 Da, found 313.1459 Da.

#### 2.2.39. 2-(5-(4-[(3-Fluorobenzyl)oxy]phenyl)-4*H*-1,2,4-triazol-3-yl)ethan-1-amine Hydrochloride (**44**)

Yield 0.051 g (31%). M.p. 214–216 °C. ^1^H NMR (300 MHz, DMSO-*d_6_*) δ 8.20 (br.s, 3H), 8.08–8.02 (m, 2H), 7.51–7.40 (m, 1H), 7.35–7.28 (m, 2H), 7.23–7.13 (m, 3H), 5.21 (s, 2H), 3.32–3.20 (m, 2H), 3.18–3.10 (m, 2H). ^13^C NMR (75 MHz, DMSO-*d_6_*) δ 162.7 (d, J = 243.6 Hz), 160.1, 156.8, 156.6, 140.1 (d, J = 7.5 Hz), 131.0 (d, J = 8.3 Hz), 128.3, 124.1 (d, J = 1.6 Hz), 120.9, 115.8, 115.2 (d, J = 21.0 Hz), 114.8 (d, J = 21.7 Hz), 69.0, 37.5, 25.0. HRMS (ESI) calcd for C_17_H_18_FN_4_O [M + H^+^] 313.1459 Da, found 313.1459 Da.

#### 2.2.40. 2-(5-(4-[(4-Fluorobenzyl)oxy]phenyl)-4*H*-1,2,4-triazol-3-yl)ethan-1-amine Hydrochloride (**45**)

Yield 0.070 g (46%). M.p. 220–222 °C. ^1^H NMR (300 MHz, DMSO-*d_6_*) δ 8.20 (s, 3H), 8.08–8.01 (m, 2H), 7.53 (dd, J = 8.6, 5.6 Hz, 2H), 7.28–7.15 (m, 4H), 5.17 (s, 2H), 3.32–3.20 (m, 2H), 3.18–3.09 (m, 2H). ^13^C NMR (75 MHz, DMSO-*d_6_*) δ 162.3 (d, J = 243.8 Hz), 160.4, 156.3, 156.2, 133.4 (d, J = 3.0 Hz), 130.6 (d, J = 8.3 Hz), 128.5, 120.3, 115.8 (d, J = 21.4 Hz), 115.8, 69.2, 37.3, 24.9. HRMS (ESI) calcd for C_17_H_18_FN_4_O [M + H^+^] 313.1459 Da, found 313.1459 Da.

#### 2.2.41. 2-(5-(4-[(2-Fluorobenzyl)oxy]-3-methylphenyl)-4*H*-1,2,4-triazol-3-yl)ethan-1-amine Hydrochloride (**46**)

Yield 0.045 g (27%). M.p. 234–236 °C. ^1^H NMR (300 MHz, DMSO-*d_6_*) δ 8.18 (br.s, 3H), 7.96–7.91 (m, 2H), 7.63–7.57 (m, 1H), 7.48–7.40 (m, 1H), 7.31–7.23 (m, 3H), 5.24 (s, 2H), 3.32–3.19 (m, 2H), 3.16–3.09 (m, 2H), 2.22 (s, 3H). ^13^C NMR (75 MHz, DMSO-*d_6_*) δ 160.4 (d, J = 246.1 Hz), 157.9, 156.2, 156.0, 130.5 (d, J = 2.5 Hz), 130.4, 128.6, 126.8, 125.6, 124.6 (d, J = 3.4 Hz), 123.8 (d, J = 14.4 Hz), 119.8, 115.5 (d, J = 20.9 Hz), 112.1, 63.9, 37.0, 24.6, 16.1. HRMS (ESI) calcd for C_18_H_20_FN_4_O [M + H^+^] 327.1616 Da, found 327.1616 Da.

#### 2.2.42. 2-(5-(4-[(3-Fluorobenzyl)oxy]-3-methylphenyl)-4*H*-1,2,4-triazol-3-yl)ethan-1-amine Hydrochloride (**47**)

Yield 0.053 g (32%). M.p. 215–216 °C. ^1^H NMR (300 MHz, DMSO-*d_6_*) δ 8.19 (br.s, 3H), 7.97–7.89 (m, 2H), 7.51–7.42 (m, 1H), 7.37–7.28 (m, 2H), 7.22–7.13 (m, 2H), 5.24 (s, 2H), 3.33–3.19 (m, 2H), 3.18–3.09 (m, 2H), 2.28 (s, 3H). ^13^C NMR (75 MHz, DMSO-*d_6_*) δ 163.2 (d, J = 243.6 Hz), 159.2, 156.1, 155.8, 140.8 (d, J = 7.4 Hz), 131.5 (d, J = 8.3 Hz), 129.8, 127.9, 126.9, 124.2 (d, J = 2.7 Hz), 119.1, 115.6 (d, J = 20.9 Hz), 114.9 (d, J = 21.9 Hz), 113.1, 69.5, 37.6, 25.1, 17.1. HRMS (ESI) calcd for C_18_H_20_FN_4_O [M + H^+^] 327.1616 Da, found 327.1616 Da.

#### 2.2.43. 2-(5-(4-[(4-Fluorobenzyl)oxy]-3-methylphenyl)-4*H*-1,2,4-triazol-3-yl)ethan-1-amine Hydrochloride (**48**)

Yield 0.020 g (12%). M.p. 182–183 °C. ^1^H NMR (300 MHz, DMSO-*d_6_*) δ 8.12 (br.s, 3H), 7.90–7.85 (m, 2H), 7.57–7.48 (m, 2H), 7.28–7.14 (m, 3H), 5.18 (s, 2H), 3.30–3.15 (m, 2H), 3.12–3.04 (m, 2H), 2.24 (s, 3H). ^13^C NMR (75 MHz, DMSO-*d_6_*) δ 161.6 (d, J = 243.6 Hz), 157.5, 156.7, 156.4, 133.1 (d, J = 3.0 Hz), 129.5 (d, J = 8.3 Hz), 128.2, 126.5, 125.1, 120.2, 115.1 (d, J = 21.4 Hz), 111.9, 68.5, 37.0, 24.6, 16.0. HRMS (ESI) calcd for C_18_H_20_FN_4_O [M + H^+^] 327.1616 Da, found 327.1616 Da.

#### 2.2.44. 2-(5-(3-Methyl-4-[([4-(trifluoromethyl)benzyl]oxy)phenyl]-4*H*-1,2,4-triazol-3-yl)) Hydrochloride (**49**)

Yield 0.048 g (25%). M.p. 208–210 °C. ^1^H NMR (300 MHz, DMSO-*d_6_*) δ 8.18 (br.s, 3H), 7.95–7.88 (m, 2H), 7.82–7.67 (m, 4H), 7.17 (d, J = 8.4 Hz, 1H), 5.33 (s, 2H), 3.32–3.19 (m, 2H), 3.17–3.08 (m, 2H), 2.29 (s, 3H). ^13^C NMR (75 MHz, DMSO-*d_6_*) δ 158.0, 155.5, 155.3, 128.8, 128.4 (q, J = 31.7 Hz), 127.8, 127.8, 126.9, 125.8, 125.5 (q, J = 3.8 Hz), 124.3 (q, J = 272.0 Hz), 118.9, 112.1, 68.5, 36.8, 24.4, 16.2. HRMS (ESI) calcd for C_19_H_20_F_3_N_4_O [M + H^+^] 377.1584 Da, found 377.1584 Da.

#### 2.2.45. 2-(5-(2-[3,4-Difluorophenoxy]phenyl)-4*H*-1,2,4-triazol-3-yl) Hydrochloride (**50**)

Yield 0.017 g (7%). M.p. 196–199 °C. ^1^H NMR (300 MHz, DMSO-*d_6_*) δ 8.22 (s, 3H), 8.10–8.04 (m, 1H), 7.54–7.43 (m, 1H), 7.36–7.19 (m, 2H), 7.04 (dt, J = 5.9, 2.9 Hz, 1H), 6.95–6.84 (m, 1H), 6.61–6.55 (m, 1H), 3.28–3.17 (m, 2H), 3.17–3.07 (m, 2H). ^13^C NMR (75 MHz, DMSO-*d_6_*) δ 157.30–152.35 (m), 154.79, 151.94 (d, J = 1.7 Hz), 150.19–146.62 (m), 132.17–130.40 (m), 124.52, 123.63, 118.00, 117.77, 116.91–115.14 (m), 111.52–108.80 (m), 106.72, 37.30, 25.06. HRMS (ESI) calcd for C_16_H_15_F_2_N_4_O [M + H^+^] 317.1208 Da, found 317.1208 Da.

#### 2.2.46. 2-(5-(4-[3,4-Dimethylphenoxy]phenyl)-4*H*-1,2,4-triazol-3-yl)ethan-1-amine Hydrochloride (**51**)

Yield 0.01 g (5%). M.p. 201–203 °C. ^1^H NMR (300 MHz, DMSO-*d_6_*) δ 8.16 (s, 3H), 8.06–7.99 (m, 2H), 7.18 (d, J = 8.1 Hz, 1H), 7.09–7.02 (m, 2H), 6.91 (d, J = 2.4 Hz, 1H), 6.85–6.79 (m, 1H), 3.30–3.18 (m, 2H), 3.16–3.07 (m, 2H), 2.21 (s, 6H). ^13^C NMR (75 MHz, DMSO-*d_6_*) δ 158.5, 157.8, 156.6, 151.2, 134.2, 132.4, 131.0, 130.9, 129.5, 128.3, 120.5, 120.1, 118.0, 113.7, 37.4, 24.7, 20.6, 15.9. HRMS (ESI) calcd for C_18_H_21_N_4_O [M + H^+^] 309.1710 Da, found 309.1710 Da.

#### 2.2.47. 2-(5-(3-[2,4-Dimethylphenoxy]phenyl)-4*H*-1,2,4-triazol-3-yl)ethan-1-amine Hydrochloride Hydrochloride (**52**)

Yield 0.052 g (23%). M.p. 308–310 °C. ^1^H NMR (300 MHz, DMSO-*d_6_*) δ 8.19 (br.s, 3H), 7.76–7.66 (m, 1H), 7.52–7.35 (m, 2H), 7.15 (br.s, 1H), 7.09–7.00 (m, 1H), 6.98–6.83 (m, 2H), 3.26–3.04 (m, 4H), 2.28 (s, 3H), 2.10 (s, 3H). ^13^C NMR (75 MHz, DMSO-*d_6_*) δ 159.2, 157.1, 156.8, 153.7, 138.7, 132.6, 131.2, 128.2, 123.2, 120.9, 118.1, 117.1, 37.4, 24.9, 19.7, 18.9. HRMS (ESI) calcd for C_18_H_21_N_4_O [M + H^+^] 309.1710 Da, found 309.1710 Da.

#### 2.2.48. 2-(5-(3-[2-Chlorophenoxy]phenyl)-4*H*-1,2,4-triazol-3-yl)ethan-1-amine Hydrochloride (**53**)

Yield 0.92 g (4%). M.p. 202–210 °C. ^1^H NMR (300 MHz, DMSO-*d_6_*) δ 8.05 (br.s, 3H), 7.81–7.77 (m, 1H), 7.64 (dd, J = 8.0, 1.6 Hz, 1H), 7.54–7.47 (m, 2H), 7.45–7.36 (m, 1H), 7.31–7.24 (m, 1H), 7.21–7.17 (m, 1H), 7.04 (dd, J = 8.0, 2.6 Hz, 1H), 3.26–3.15 (m, 2H), 3.11–3.03 (m, 2H). ^13^C NMR (75 MHz, DMSO-*d_6_*) δ 157.2, 157.1, 154.4, 151.1, 130.9, 130.7, 129.0, 126.0, 125.0, 121.8, 121.0, 120.9, 118.3, 114.2, 37.1, 24.6. HRMS (ESI) calcd for C_16_H_16_ClN_4_O [M + H^+^] 315.1007 Da, found 315.1007 Da.

#### 2.2.49. 2-(5-(4-[4-Methylphenoxy]phenyl)-4*H*-1,2,4-triazol-3-yl)ethan-1-amine Hydrochloride (**54**)

Yield 0.03 g (13%). M.p. 234–236 °C. ^1^H NMR (300 MHz, DMSO-*d_6_*) δ 8.16 (br.s, 3H), 8.06–8.00 (m, 2H), 7.28–7.21 (m, 2H), 7.09–6.96 (m, 4H), 3.30–3.19 (m, 2H), 3.16–3.07 (m, 2H), 2.31 (s, 3H). ^13^C NMR (75 MHz, DMSO-*d_6_*) δ 158.9, 156.7, 156.3, 153.3, 133.5, 130.7, 128.1, 122.9, 119.6, 117.9, 37.0, 24.6, 20.4. HRMS (ESI) calcd for C_17_H_19_N_4_O [M + H^+^] 295.1553 Da, found 295.1553 Da.

#### 2.2.50. 3-(5-(2-Aminoethyl)-4*H*-1,2,4-triazol-3-yl)phenol Hydrochloride (**55**)

Yield 0.022 g (11%). M.p. 234–235 °C. ^1^H NMR (300 MHz, DMSO-*d_6_*) δ 8.18 (br.s, 3H), 7.50–7.43 (m, 2H), 7.29 (t, J = 7.8 Hz, 1H), 6.92–6.86 (m, 1H), 3.31–3.19 (m, 2H), 3.17–3.08 (m, 2H). ^13^C NMR (75 MHz, DMSO-*d_6_*) δ 157.8, 157.0, 156.3, 130.1, 129.4, 117.1, 117.0, 113.0, 37.1, 24.6. HRMS (ESI) calcd for C_10_H_13_N_4_O [M + H^+^] 205.1084 Da, found 205.1084 Da.

#### 2.2.51. 2-(5-(4-[(2-Fluorobenzyl)oxy]-3-iodophenyl)-4*H*-1,2,4-triazol-3-yl)ethan-1-amine Hydrochloride (**56**)

Yield 0.045 g (20%). M.p. 208–209 °C. ^1^H NMR (300 MHz, DMSO-*d_6_*) δ 8.47–8.44 (m, 1H), 8.14 (br.s, 3H), 8.08–8.00 (m, 1H), 7.71–7.63 (m, 1H), 7.49–7.40 (m, 1H), 7.33–7.24 (m, 3H), 5.30 (s, 2H), 3.30–3.18 (m, 2H), 3.15–3.06 (m, 2H). ^13^C NMR (75 MHz, DMSO-*d_6_*) δ 160.5 (d, J = 246.2 Hz), 158.0, 156.8, 156.4, 136.7, 130.8 (d, J = 7.9 Hz), 130.6, 127.8, 124.8, 124.0, 123.5 (d, J = 14.8 Hz), 115.7 (d, J = 19.4 Hz), 113.3, 87.3, 65.1, 37.3, 24.8. HRMS (ESI) calcd for C_17_H_17_FIN_4_O [M + H^+^] 439.0426 Da, found 439.0426 Da.

#### 2.2.52. 4-(5-(2-Aminoethyl)-4*H*-1,2,4-triazol-3-yl)-2-methylphenol Hydrochloride (**57**)

Yield 0.057 g (29%). M.p. 158–160 °C. ^1^H NMR (300 MHz, DMSO-*d_6_*) δ 8.31 (br.s, 3H), 8.01–7.97 (m, 1H), 7.94–7.88 (m, 1H), 7.03 (d, J = 8.4 Hz, 1H), 3.42–3.27 (m, 2H), 3.27–3.19 (m, 2H), 2.17 (s, 3H). ^13^C NMR (75 MHz, DMSO-*d_6_*) δ 159.27, 153.66, 153.49, 129.70, 126.46, 125.16, 115.22, 113.84, 36.40, 23.82, 15.99. HRMS (ESI) calcd for C_11_H_15_N_4_O [M + H^+^] 219.1240 Da, found 219.1240 Da.

### 2.3. General Procedure 2—Synthesis of Compounds ***58***–***67***

Compound **68** or **69** (2.06 mmol, 1 equiv.) was dissolved in dry tetrohydrofuran (10 mL). Then, 3,4-Dihydro-2*H*-pyran (0.93 mL, 10 mmol, 5 equiv.) and *p*-toluenesulfonic acid (35 mg, 0.21 mmol, 0.1 equiv.) were added and the resulting mixture was heated at reflux for 5 h. The course of the reaction was followed by thin-layer chromatography (eluent—chloroform). On completion, the reaction mixture was cooled to room temperature and partitioned between ethyl acetate (100 mL) and water (50 mL). The organic layer was separated, washed with 5% aq. K_2_CO_3_ and 3% aq. citric acid, filtered through a plug of anhydrous Na_2_SO_4_ and concentrated on a rotary evaporator. The yield of the THP protection step was deemed quantitative. The flask with the residue was flushed with argon and the residue (1 mmol) was weighed out, under argon, into an argon filled flask and dissolved in 1,4-dioxane. 20% aq. Na_2_CO_3_ (3.5 mL) and the respective boronic acid (2 mmol) were added, followed by Pd(PPh_3_)_4_ (0.03 mmol) and PdCl_2_(PPh_3_)_2_ (0.03 mmol). The mixture was heated at reflux under argon for 3–4 h while the progress of the reaction was monitored by thin-layer chromatography (eluent—2% methanol in chloroform). On completion, the reaction mixture was cooled to room temperature and partitioned between ethyl acetate (100 mL) and water (50 mL). The organic phase was washed with 5% aq. K_2_CO_3_, passed through a plug of anhydrous Na_2_SO_4_, and concentrated in vacuo and the residue was treated, fractionated, on silica gel using 50% ethyl acetate in hexane as eluent. Fractions containing the coupling product were pooled and concentrated to dryness. The residue was treated with 4M solution of HCl in 1,4-dioxane (1 h, room temperature). Following concentration on a rotary evaporator, the crystalline residue was triturated with ether and the solids were filtered off, washed with more ether, and air-tried to provide an analytically pure title compound as a crystalline residue.

#### 2.3.1. 2-(5-(4′-Methoxy-(1,1′-biphenyl)-4-yl)-4*H*-1,2,4-triazol-3-yl)ethan-1-amine Hydrochloride (**58**)

Yield 0.065 g (50%). M.p. 300–301 °C. ^1^HNMR (300 MHz, DMSO-*d_6_*) δ 8.23 (br.s, 3H), 8.13 (d, J = 8.2 Hz, 2H), 7.84–7.76 (m, 2H), 7.74–7.67 (m, 2H), 7.05 (d, J = 8.5 Hz, 2H), 3.81 (s, 3H), 3.33–3.22 (m, 2H), 3.20–3.12 (m, 2H). ^13^CNMR (75 MHz, DMSO-*d_6_*) δ 159.5, 157.0, 156.6, 141.3, 131.6, 128.0, 126.9, 126.7, 126.6, 114.7, 55.4, 37.2, 24.7. HRMS (ESI) calcd forC_17_H_19_N_4_O [M + H^+^] 295.1553 Da, found 295.1553 Da.

#### 2.3.2. 2-(5-(4′-Trifluoromethoxy-(1,1′-biphenyl)-4-yl)-4*H*-1,2,4-triazol-3-yl)ethan-1-amine Hydrochloride (**59**)

Yield 0.06 g (35%). M.p. 299–300 °C. ^1^H NMR (300 MHz, DMSO-*d_6_*) δ 8.25 (br.s, 3H), 8.17 (d, J = 8.3 Hz, 2H), 7.87 (t, J = 8.1 Hz, 4H), 7.48 (d, J = 8.3 Hz, 2H), 3.34–3.22 (m, 2H), 3.21–3.13 (m, 2H). ^13^C NMR (75 MHz, DMSO-*d_6_*) δ 157.3, 156.6, 148.1, 140.0, 138.7, 128.8, 128.3, 127.5, 126.9, 121.7, 120.3 (d, J = 256.3 Hz), 37.2, 24.6. HRMS (ESI) calcd for C_17_H_16_F_3_N_4_O [M + H^+^] 349.1271 Da, found 349.1271 Da.

#### 2.3.3. 2-(5-(3′-Fluoro-(1,1′-biphenyl)-4-yl)-4*H*-1,2,4-triazol-3-yl)ethan-1-amine Hydrochloride (**60**)

Yield 0.042 g (26%). M.p. 260–262 °C. ^1^H NMR (300 MHz, DMSO-*d_6_*) δ 8.20 (br.s, 3H), 8.15 (d, J = 8.2 Hz, 2H), 7.87 (d, J = 8.2 Hz, 2H), 7.67–7.47 (m, 3H), 7.29–7.18 (m, 1H), 3.33–3.21 (m, 2H), 3.19–3.10 (m, 2H). ^13^C NMR (75 MHz, DMSO-*d_6_*) δ 163.1 (d, J = 243.4 Hz), 157.4, 156.7, 142.1 (d, J = 8.0 Hz), 140.3, 131.4 (d, J = 8.4 Hz), 128.7, 127.8, 127.1, 123.2 (d, J = 2.4 Hz), 115.1 (d, J = 21.4 Hz), 113.9 (d, J = 22.2 Hz), 37.4, 25.0. HRMS (ESI) calcd for C_16_H_16_FN_4_ [M + H^+^] 283.1354 Da, found 283.1354 Da.

#### 2.3.4. 2-(5-(2′,4′-Difluoro-(1,1′-biphenyl)-4-yl)-4*H*-1,2,4-triazol-3-yl)ethan-1-amine Hydrochloride (**61**)

Yield 0.059 g (44%). M.p. 251–253 °C. ^1^H NMR (300 MHz, DMSO-*d_6_*) δ 8.12 (m, 5H), 7.73–7.60 (m, 3H), 7.45–7.34 (m, 1H), 7.29–7.18 (m, 1H), 3.32–3.20 (m, 2H), 3.18–3.09 (m, 2H). ^13^C NMR (75 MHz, DMSO-*d_6_*) (75 MHz, DMSO) δ 162.0 (dd, J = 208.5, 12.3 Hz), 158.7 (dd, J = 210.1, 12.4 Hz), 156.4, 155.7, 135.5, 131.8 (dd, J = 9.7, 4.6 Hz), 129.1 (d, J = 2.7 Hz), 127.6, 126.2, 123.9 (dd, J = 13.1, 3.8 Hz), 112.1 (dd, J = 21.1, 3.6 Hz), 104.7 (t, J = 26.6 Hz), 36.7, 24.2. HRMS (ESI) calcd for C_16_H_15_F_2_N_4_ [M + H^+^] 301.1259 Da, found 301.1259 Da.

#### 2.3.5. 2-(5-(4′-Chloro-(1,1′-biphenyl)-4-yl)-4H-1,2,4-triazol-3-yl)ethan-1-amine Hydrochloride (**62**, LK00764)

Yield 0.047 g (36%). M.p. 247–249 °C. ^1^H NMR (300 MHz, DMSO-*d_6_*) δ 8.23–8.09 (m, 5H), 7.87–7.74 (m, 4H), 7.58–7.51 (m, 2H), 3.32–3.20 (m, 2H), 3.18–3.09 (m, 2H). ^13^C NMR (75 MHz, DMSO-*d_6_*) δ 157.1, 156.4, 139.1, 138.0, 132.7, 129.0, 128.4, 128.1, 127.0, 126.7, 37.0, 24.6. HRMS (ESI) calcd for C_16_H_16_ClN_4_ [M + H^+^] 299.1058 Da, found 299.1058 Da.

#### 2.3.6. 2-(5-(3′-Trifluoromethyl-(1,1′-biphenyl)-4-yl)-4*H*-1,2,4-triazol-3-yl)ethan-1-amine Hydrochloride (**63**)

Yield 0.04 g (27%). M.p. 210–212 °C. ^1^H NMR (300 MHz, DMSO-*d_6_*) δ 8.27–8.14 (m, 5H), 8.11–8.02 (m, 2H), 7.97–7.89 (m, 2H), 7.81–7.69 (m, 2H), 3.34–3.22 (m, 2H), 3.19–3.11 (m, 2H). ^13^C NMR (75 MHz, DMSO-*d_6_*) δ 156.98, 156.29, 140.33, 139.54, 130.83, 130.19, 129.88 (q, J = 31.6 Hz), 128.53, 127.50, 126.75, 124.47 (q, J = 3.5 Hz), 124.22 (q, J = 272.5 Hz), 123.13 (q, J = 3.7 Hz), 36.97, 24.55. HRMS (ESI) calcd for C_17_H_16_F_3_N_4_ [M + H^+^] 333.1322 Da, found 333.1322 Da.

#### 2.3.7. 2-(5-(3′,4′-Dimethoxy-(1,1′-biphenyl)-4-yl)-4*H*-1,2,4-triazol-3-yl)ethan-1-amine Hydrochloride (**64**)

Yield 0.046 g (32%). M.p. 199–201 °C. ^1^H NMR (300 MHz, DMSO-*d_6_*) δ 8.28 (br.s, 3H), 8.16 (d, J = 8.4 Hz, 2H), 7.84 (d, J = 8.4 Hz, 2H), 7.33–7.27 (m, 2H), 7.08–7.03 (m, 1H), 3.86 (s, 3H), 3.80 (s, 3H), 3.35–3.25 (m, 2H), 3.23–3.16 (m, 2H). ^13^C NMR (75 MHz, DMSO-*d_6_*) δ 156.2, 155.9, 149.2, 149.1, 141.8, 131.8, 126.8, 126.7, 125.8, 119.1, 112.3, 110.4, 55.8, 55.7, 36.9, 24.5. HRMS (ESI) calcd for C_18_H_21_N_4_O_2_ [M + H^+^] 325.1659 Da, found 325.1659 Da.

#### 2.3.8. 2-(5-(3′,5′-Difluoro-(1,1′-biphenyl)-4-yl)-4*H*-1,2,4-triazol-3-yl)ethan-1-amine Hydrochloride (**65**)

Yield 0.05 g (38%). M.p. 260–262 °C. ^1^H NMR (300 MHz, DMSO-*d_6_*) δ 8.32 (br.s, 3H), 8.22–8.17 (m, 2H), 7.95–7.90 (m, 2H), 7.59–7.49 (m, 2H), 7.31–7.22 (m, 1H), 3.35–3.16 (m, 4H). ^13^C NMR (75 MHz, DMSO-*d_6_*) δ 162.2 (dd, J = 205.6, 12.3 Hz), 158.9 (dd, J = 207.4, 12.3 Hz), 157.5, 156.3, 135.3, 131.9 (dd, J = 9.7, 4.7 Hz), 129.2 (d, J = 2.9 Hz), 128.5, 126.2, 124.2 (dd, J = 13.3, 3.8 Hz), 112.2 (dd, J = 21.2, 3.6 Hz), 104.6 (dd, J = 26.9, 25.9 Hz), 37.0, 24.6. HRMS (ESI) calcd for C_16_H_15_F_2_N_4_ [M + H^+^] 301.1259 Da, found 301.1259 Da.

#### 2.3.9. 2-(5-(4′-(2′,4′-Difluoro-(1,1′-biphenyl)-3-yl)-4*H*-1,2,4-triazol-3-yl)ethan-1-amine Hydrochloride (**66**)

Yield 0.0262 g (19%). M.p. 255–257 °C. ^1^H NMR (300 MHz, DMSO-*d_6_*) δ 8.28 (br.s, 3H), 8.23 (s, 1H), 8.14–8.07 (m, 1H), 7.73–7.59 (m, 3H), 7.45–7.35 (m, 1H), 7.28–7.19 (m, 1H), 3.34–3.13 (m, 4H). ^13^C NMR (75 MHz, DMSO-*d_6_*) δ 162.1 (dd, J = 211.4, 12.4 Hz), 158.9 (dd, J = 213.0, 12.3 Hz), 157.0, 156.0, 134.9, 132.0 (dd, J = 9.7, 4.6 Hz), 130.2 (d, J = 2.3 Hz), 129.4, 128.9, 126.3 (d, J = 2.3 Hz), 125.6, 124.2 (dd, J = 13.3, 3.8 Hz), 112.2 (dd, J = 21.2, 3.7 Hz), 104.6 (t, J = 26.4 Hz), 36.9, 24.4. HRMS (ESI) calcd for C_16_H_15_F_2_N_4_ [M + H^+^] 301.1259 Da, found 301.1259 Da.

#### 2.3.10. 2-(5-(4′-(Trifluoromethoxy)-(1,1′-biphenyl)-3-yl)-4*H*-1,2,4-triazol-3-yl)ethan-1-amine Hydrochloride (**67**)

Yield 0.045 g (28%). M.p. 253–257 °C. ^1^H NMR (300 MHz, DMSO-*d_6_*) δ 8.38–8.35 (m, 1H), 8.18 (br.s, 3H), 8.09–8.04 (m, 1H), 7.91–7.85 (m, 2H), 7.82–7.77 (m, 1H), 7.66–7.59 (m, 1H), 7.53–7.47 (m, 2H), 3.31–3.21 (m, 2H), 3.18–3.11 (m, 2H). ^13^C NMR (75 MHz, DMSO-*d_6_*) δ 157.1, 156.5, 148.1 (q, J = 1.7 Hz), 139.4, 138.8, 129.8, 129.4, 128.8, 128.2, 125.5, 124.4, 121.6, 120.2 (q, J = 256.4 Hz), 37.0, 24.6. HRMS (ESI) calcd for C_17_H_16_F_3_N_4_O [M + H^+^] 349.1271 Da, found 349.1271 Da.

### 2.4. In Silico Modeling

The TAAR1 protein homology model was built with AlphaFold software [17]. The protein model (Q96RJ0) was downloaded from the Uniprot database [18]. The downloaded protein model was preprocessed with the use of a protein preparation wizard, included in Schrodinger Suite (version 2021-4). At this step, we eliminate typical errors in the protein model, such as invalid bond orders, protonation states, atom typing, missing amino acid sidechains, missing loops, clashes between sidechains, incorrect torsions, etc. All manipulations with proteins and ligands were carried out in the OPLS46 force field, used in Schrodinger Suite [19]. The three-dimensional structure of the ligands used for calculations was generated in the same forcefield—OPLS46 [19]. Moreover, for all ligands, possible protonation states were calculated with the use of the Epik module of Schrodinger Suite [20]. Ligand docking with the prepared TAAR1 protein model was performed with the use of a Glide induced-fit docking (IFD) method [21]. This takes into account protein flexibility in the presence of ligand. The IFD protocol supports direct grid box placement. The grid box for molecular docking was centered on the following TAAR1 residues: D103, I104, S107, V184, F186, T194, F195, F267, F268, I290, and Y294. The grid size is: 16 × 16 × 16 Å. Protein structure refinement during IFD was limited by 6Å of ligand poses with sidechain optimization. The first docking step generated 20 poses per ligand. For redocking (after protein structure refinement), structures within 30 kcal/mol of the best structure and within the top 15 structures per ligand were selected. The best-fitting binding pose was selected in accordance with docking solutions clustering and interactions with the crucial residues found in literature [9,16,22]. Final prioritization was determined by IFDscore and GlideScore values. The metadynamics calculations protocol was fully automatic: solvent—SPC, automatic counterions addition, system size in accordance to the protein–ligand complex with buffer zone of 10 Å. Equilibration: GCMC Solvate Pocket, muVT ensemble, T = 300 K, restraints on heavy atoms; GCMC Solvate Pocket, muVT ensemble, T = 300 K, no restraints; Brownian Dynamics NVT, T = 300 K, small timesteps, and restraints on solute heavy atoms. Main simulation—10 ns, NPT, T = 300 K. For each ligand, metadynamics simulation was repeated seven times, which was necessary for statistical weighting. On the basis of molecular metadynamics calculations, the protein–ligand complex stability was evaluated by RMSD value calculation for a ligand near protein. The second value—persistence CompScore, value including protein–ligand interactions, such as π–π stacking, hydrogen bonds, and salt bridges.

### 2.5. BRET Analysis

The Bioluminescence Resonance Energy Transfer approach (BRET) has been used for functional activity experiments. HEK-293 cells were transiently transfected with cDNA for TAARs and cAMP BRET biosensor (EPAC) and then plated in a 96-well plate, as described [23]. All compounds were tested at the initial concentration of 10 µM. Then, for active compounds, a concentration-response analysis was performed in order to calculate the EC_50_ values. A natural agonist of TAAR1 β-phenylethylamine (β-PEA) was used as a positive control.

### 2.6. In Vivo Efficacy Evaluation

#### 2.6.1. Subjects

Drug and test naïve Wistar (3–4 months old in the beginning of experiments) rats were purchased from the State Breeding Farm “Rappolovo” (St. Petersburg, Russia). The recently described dopamine transporter knockout rats (DAT-KOs) were raised in an animal facility of the Valdman Institute of Pharmacology. All rodents were housed under standard laboratory conditions: 12 h light/dark cycle (lights on at 08:00 h) at 21 ± 2 °C and 50 ± 20% humidity. The rats were housed in groups of three to five in standard T IV cages (Tecniplast, Buguggiate, VA, Italy). During the entire experiment, the rats had free access to filtered (“AQUAPHOR”, Saint Petersburg, Russia) tap water and standard laboratory rat chow (receipt ΠK 120-1, KKZ “Laboratorkorm”, Moscow, Russia). All experiments were carried out during the light period of the light/dark cycle after at least one week of habituation to the animal facility. The cages, corn cob bedding (“KKZ “Zolotoy pochatok”” LLC, Voronezh, Russia), and water bottles were changed once a week.

Experimental protocols were approved by the Local Animal Care and Use Committee of First Pavlov State Saint Petersburg Medical University.

#### 2.6.2. Compounds

MK-801 (dizocilpine, (+)-5-methyl-10,11-dihydro-5H-dibenzo-[*a,d*]-cyclohepten-5,10-imine maleate; Sigma-Aldrich, St. Louis, MO, USA) was dissolved in saline. Compound **62** (LK00764) was dissolved in 10% Tween 80 saline solution. All solutions were made fresh shortly before injections. Drug doses were tested in a pseudo-random order derived from Latin square design with at least 72 h between consecutive tests. All drugs and vehicles were administered intraperitoneally (i.p.) in the volume of 1 mL/kg. Drug doses are reported as salts. Selection of the MK-801 dose (0.2 mg/kg) was based the previous experience with the compound [24]. All locomotor activity tests (duration—60 min) were performed 15 min after the administration of compounds.

#### 2.6.3. Evaluation of Rat Locomotor Activity Following Drug i.p. Administration

We evaluated locomotor activity of rats in the Actometer apparatus. A detailed description of the apparatus has been presented before [25]. Shortly, each box of the apparatus was equipped by photocell-based infrared sensors to assess horizontal and vertical activity. Wistar rats were tested in the apparatus following a one-week habituation period (baseline period). During this period, the animals were placed in locomotor activity boxes for 60 min per day, 6 days a week to habituate them to the environment. The pharmacological challenge in DAT-KO rats was conducted without habituation to the apparatus.

#### 2.6.4. Stress-Induced Hyperthermia (SIH)Test

SIH test is commonly used to evaluate the potential anti-anxiety effect of drugs [26]. Thermometry was performed rectally in rats with a BIOSEB thermometer. Eight Wistar male rats were injected i.p. with vehicle or LK00764, dissolved in 10% tween 80 saline solution. Latin square design was used for the experiment. The first measurement was performed directly before the injection and the second measurement was performed 15 min later. The difference between the first and the second temperature was calculated.

#### 2.6.5. Statistical Analysis

We used nonparametric statistical methods to analyze the data of the locomotor experiments [25,26]. Alpha was set at 0.05. All statistical analyses were performed using SigmaPlot 12.5 (Systat Software Inc., San Jose, CA, USA). For analysis of the SIH test experiments, Kruskal–Wallis one-way ANOVA was applied.

## 3. Results and Discussion

### 3.1. Chemistry

**Figure 2:**
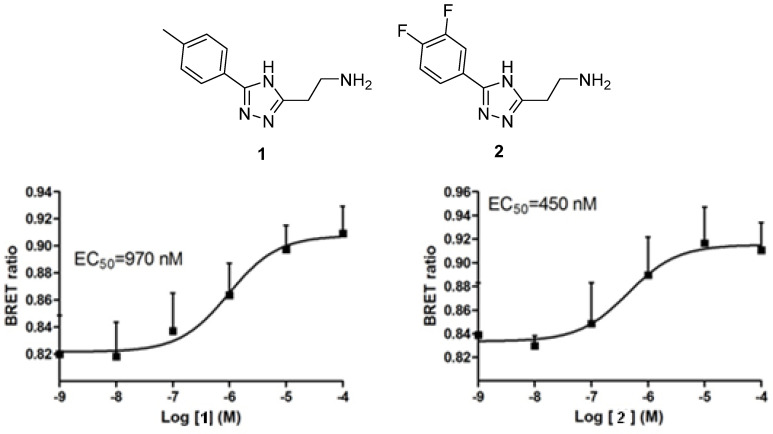
Structures and TAAR1 activation dose–response curves of screening hits **1** and **2** (n = 3).

In order to identify new compounds which do not belong to the chemotypes depicted in Figure 1, we performed screening of a focused in-house library of about 1000 compounds comprising various heterocyclic motifs in combination with structural fragments similar to β-PEA or tyramine, well established ligands of TAAR1, as well as other biogenic amine GPCRs. Such a strategy had been reported to deliver higher hit rates in screening a corporate compound collection containing compounds from historic biogenic amine-based drug discovery programs [15]. This hit finding effort identified two submicromolar agonists of TAAR1 (**1** and **2**), both of which displayed a dose-dependent activation of the receptor and were based on the same 2-(4*H*-1,2,4-triazol-3-yl)ethan-1-amine core not previously associated with TAAR1 modulation in the literature (Figure 2).

**Scheme 1:**
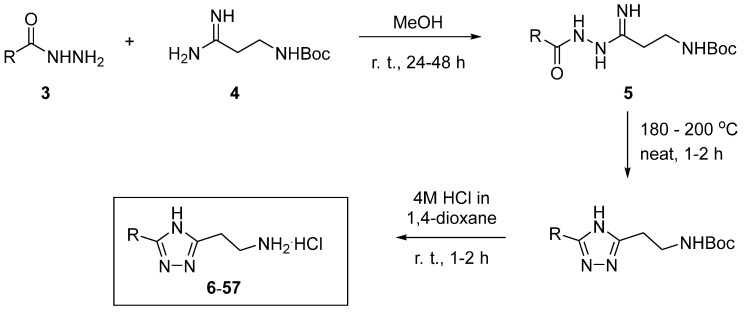
Synthesis of 2-(4*H*-1,2,4-triazol-3-yl)ethan-1-amine analogs **6**–**57** of the initial hits **1**–**2**.

In order to improve the potency of the newly identified chemotype and possibly understand structure–activity relationships, we performed a massive hit-to-lead analog synthesis by utilizing diversely substituted stock-available (hetero)aromatic carboxylic acid hydrazides **3** and Boc-protected amino amidine building block 4 [27], as depicted in Scheme 1. The three steps were conducted with only one intermittent chromatographic purification after the neat melting of *N*-acyl amidrazone **5**. Thus, the yields of hydrochlorides **6**–**57** were calculated over the three consecutive steps.

**Scheme 2:**
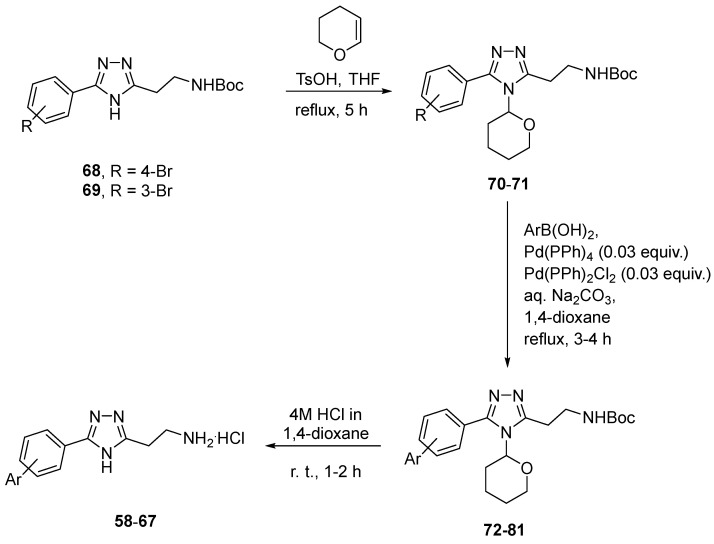
Synthesis of biphenyl derivatives **58**–**67**.

The resulting 2-(4*H*-1,2,4-triazol-3-yl)ethan-1-amine analogs **6**–**57** were tested against TAAR1, as described below. As the TAAR1 activation data for the initial set of compounds (**6**–**57**) indicated a clear preference for more lipophilic aromatic substituents on the 1,2,4-triazole core (see discussion in the 3.2. TAAR1 Agonistic Activity section below), we aimed at synthesizing a set of 2-(5-([1,1′-biphenyl]-4-yl)-4*H*-1,2,4-triazol-3-yl)ethan-1-amines **58**–**67**. For this purpose, Boc-protected 2-(5-(4-bromophenyl)-4*H*-1,2,4-triazol-3-yl)ethan-1-amine **68** and Boc-protected 2-(5-(3-bromophenyl)-4*H*-1,2,4-triazol-3-yl)ethan-1-amine **69** were viewed as common precursors which were synthesized on a multigram scale. Unfortunately, application of the standard Suzuki coupling protocol did not lead to appreciable conversion of either **68** or **69** in the attempted coupling. To circumvent this obstacle, the 1,2,4-triazole moiety in these starting materials was protected, with quantitative yield, with the tetrahydropyranyl group [28], and the resulting protected derivatives **70**–**71** were successfully coupled to a set of boronic acids under mixed Pd(0)/Pd(II) catalysis. Brief chromatographic purification of THP/Boc-protected derivatives **72**–**73** followed by treatment with 4M HCl in 1,4-dioxane led to simultaneous removal of both protecting groups and afforded biphenyl derivatives **58**–**67** as hydrochloride salts in modest yields over three steps (Scheme 2). These biphenyl derivatives were also tested for TAAR1 agonism, which led to the discovery of the lead compound in the series (vide infra).

### 3.2. TAAR1 Agonistic Activity

Hit expansion compounds **6**–**57** and **58**–**67** (see Supplementary Materials for characterization data) were tested for TAAR1 activation using the earlier-developed BRET assay [29]. In addition to EC_50_ values obtained for each compound, its maximum agonistic effect (E_max_) was compared to that produced by 1 μM β-phenylethylamine (β-PEA) TAAR1 agonist, employed in this assay as a reference [30]. The data obtained for the initial set of analogs are compiled in Table 1.

From the examination of the activity data obtained for the unbiased SAR survey set (**6**–**57**), it becomes apparent that a simple variation in substituents on the aromatic ring does not lead to substantial improvement in potency. Most of the active compounds manifested themselves as full TAAR1 agonists. Among other things, this initial SAR survey revealed that replacement of the aryl moiety with thienyl (*cf.* **16** and **18**) or nitrogen heteroaromatic (*cf.* **10**, **35** and **38**) as well as highly electron-rich moieties (*cf.* **11**, **24**, **30**, **32**) was detrimental to the potency. At the same time, substitution of the benzene ring with lipophilic groups in the *para*-position appears to have a positive effect on the agonistic activity (*cf.* **19**, **40**, **43**–**45**), which reaches into the double-digit nanomolar range. Interestingly, the position of the ‘walking fluorine’ in two groups of analogs—**43**–**45** and **46**–**48**—has little effect on the potency. However, methyl substitution in the proximal benzene ring (in **46**–**48**) led to a noticeable drop in the activity. A similar lowering of the activity results from the introduction of iodo substitutions in the phenyl ring (*cf.* **41**–**42** and **56**). Introduction of a hydroxy substituent in the phenyl ring (*cf.* **55** and **57**) completely ablates the agonistic activity.

As hinted at in Section 3.1. Chemistry, above, the latter observation inspired us to explore the rigidified biphenyl versions of the initial hits **58**–**67**, which were also tested against TAAR1 in BRET assay. The EC_50_ values obtained for this subset of analogs are compiled in Table 2.

The rigidified biphenyl analogs **58**–**67** appeared to display a much better, full agonistic profile with respect to TAAR1. Clearly, the linear *p*-biphenyl versions **58**–**65** were preferred over *m*-biphenyl counterparts **66**–**67**. Simply based on the best potency displayed by compound **62** (LK00764) (its potency (EC_50_ 4 nM) being more than 30 times higher than that of Ulotaront (EC_50_ 140 nM) [31], which received FDA Breakthrough Therapy Designation and is currently being investigated in Phase 3 clinical trials [9], it was nominated for further evaluation in rodent pharmacological tests sensitive to TAAR1 agonists [6,7,10,11] and relevant to the development of novel antipsychotics.

### 3.3. In Silico Modeling

**Figure 3:**
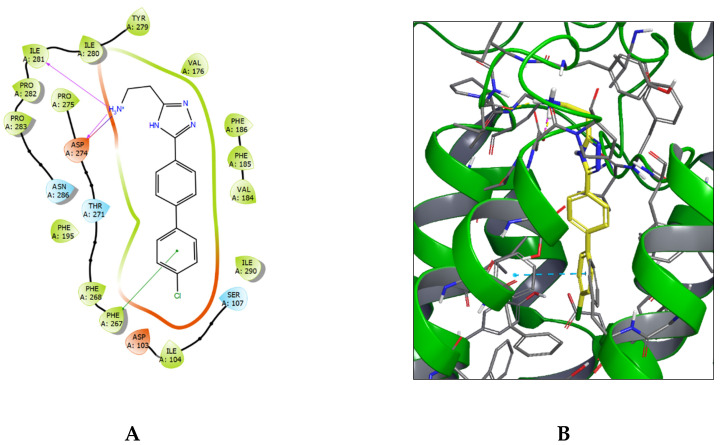
Ligand–protein complex of **62** (LK000764) with TAAR1; (**A**)—ligand interaction diagram, (**B**)—ligand positioning in TAAR1 protein structure (blue dashed line—π–π stacking interaction, yellow—hydrogen bond, magenta—salt bridge).

No crystal structure of TAAR1 is available in the Protein Data Bank (PDB). However, modelled structures of TAAR1-interactions obtained by homology modeling can be found in GproteinDb [32]. For instance, a homology model for TAAR1 has been built [9,33] based on the structure of the β2-adrenergic receptor [34] and used in virtual screening of new TAAR1 ligands [35]. Induced-fit docking of **62** (LK000764) showed a high affinity level of this ligand to TAAR1, which was confirmed by the calculated scoring function values: GScore (kcal/mol): −10.77; IFDScore(kcal/mol): −648.38. The analysis of the binding pose revealed that **62**, in its complex with TAAR1, forms a rich network of lipophilic protein–ligand contacts. The strongest lipophilic contacts were registered in the phenylalanine-rich region of TAAR1: Phe185/186 and Phe195/267/268. This fact was confirmed by the high calculated increment of lipophilic contacts in the scoring function value: −4.27 kcal/mol. The biphenyl moiety of **62** contributes most to these interactions, which is further supported by the π–π stacking interaction with the Phe267 aromatic ring. The other key interaction defining the binding pose is the salt bridge between Asp274 and the protonated amine moiety of **62**, possibly supported by hydrogen bonding to the same Asp274 residue and to the Ile281 backbone residue (Figure 3).

The protein–ligand complex of **62** (LK000764) was tested with the molecular metadynamics method in order to assess molecule retention stability in the TAAR1 active site [36]. The purpose of this method is to evaluate the stability of poses with a Desmond metadynamics [37] molecular dynamics simulation to determine the correctness of the docking pose. The stability was assessed in terms of the fluctuations of the ligand RMSD over the course of the simulation and the persistence of important contacts between the ligand and the receptor (and any other cofactors or solvent molecules) such as hydrogen bonds and π–π interactions. The collective variable for the metadynamics simulation is the ligand RMSD from its initial pose, evaluated after superposition of the binding sites to account for drift. To improve the statistics, multiple simulations were performed, and the results were averaged over the simulations.

### 3.4. In Vivo Pharmacological Characterization

#### 3.4.1. Effect on MK-801-Induced Hyperactivity and Spontaneous Activity in Rats

Glutamate NMDA receptor antagonist-induced hyperactivity in rodents is commonly used as a pharmacological animal model of schizophrenia in the screening of potential antipsychotics. It is known that the pharmacological activation of TAAR1 is able to decrease NMDA blockers-induced hyperactivity in rodents [8,38]. Thus, we used MK-801 (0.2 mg/kg, i.p.) treatment to induce hyperactivity in rats and analyzed the effects of compound **62** (LK00764) at doses of 0.3, 1, 3, 5, and 10 mg/kg, i.p. on locomotor activity of MK-801-treated Wistar rats.

**Figure 4:**
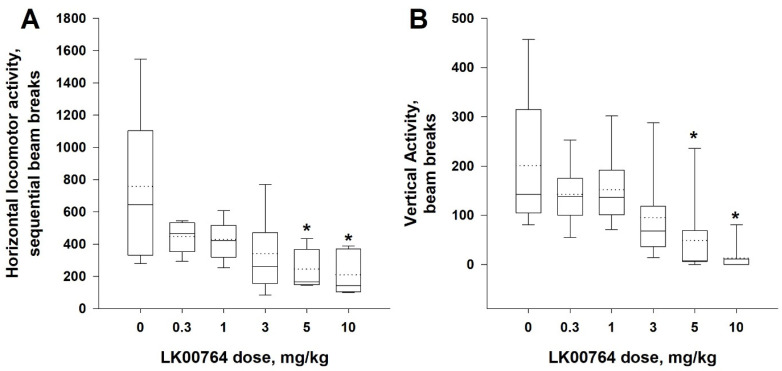
Effect of compound **62** (LK00764) on MK-801-induced hyperactivity. The mitigation action of LK00764 (0.3–10 mg/kg, i.p.) on locomotor hyperactivity following MK-801 (0.2 mg/kg, i.p.) administration in rats (n = 7). (**A**). Horizontal activity (**B**). Vertical activity. Data are represented as medians (solid lines), means (dotted lines), and 25th and 75th percentiles in a box-whisker plot, with whiskers indicating 10th and 90th percentiles. * *p* < 0.05 (Dunnett’s test) compared with vehicle treatment.

The Friedman test revealed that pretreatment with compound **62** (LK00764) dose-dependently mitigated MK-801-induced hyperactivity in rats (the main effect of factor ‘dose’ on horizontal activity: χ^2^ = 11.35, df = 5, *p* < 0.05; on vertical activity: χ^2^ = 20.47, df = 5, *p* < 0.01). The pairwise comparisons (Dunnett’s test compared with vehicle control treatment) indicated the significant (*p* < 0.05) effect of doses of 5 and 10 mg/kg on both horizontal and vertical locomotor activity (Figure 4A,B).

**Figure 5:**
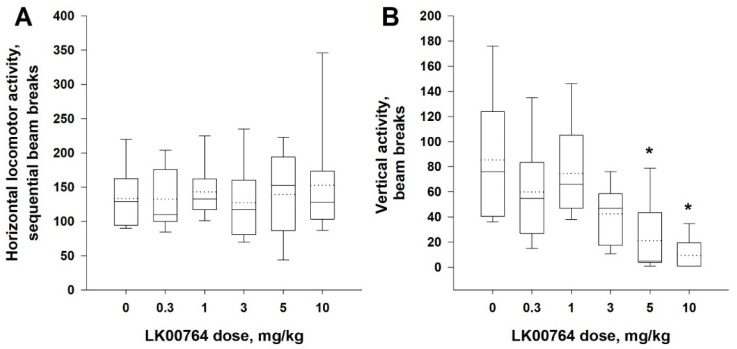
Effect of compound **62** (LK00764) locomotor activity of intact rats. The pretreatment with compound **62** (LK00764, 0.3–10 mg/kg, i.p.) did not affect horizontal (**A**), but dose-dependently decreased vertical, activity (**B**) of intact rats. Data are represented as medians (solid lines), means (dotted lines), and 25th and 75th percentiles in a box-whisker plot, with whiskers indicating 10th and 90th percentiles. * *p* < 0.05 (Dunnett’s test) compared to the vehicle-treated rats (n = 9).

Next, we analyzed the locomotor effects of compound **62** (LK00764, 0.3–10 mg/kg, i.p.) on the additional group of intact rats (n = 9). The administration of compound **62** (LK00764) did not affect horizontal but decreased vertical activity of untreated rats (Figure 5A,B). The Friedmann test demonstrated that the pretreatment with compound **62** (LK00764) did not affect the horizontal activity of Wistar rats (the main effect of the factor ‘dose’: χ^2^ = 3.63, df = 5, *p* = 0.60) but diminished vertical activity of the animals (the main effect of the factor ‘dose’: χ^2^ = 27.11, df = 5, *p* < 0.001).

#### 3.4.2. Effects on Spontaneous Locomotor Hyperactivity of Dopamine Transporter Knockout (DAT-KO) Rats

Animals without DAT have a disrupted re-uptake of dopamine resulting in remarkably elevated extracellular dopamine levels and spontaneous locomotor hyperactivity [39]. Hyperdopaminergic DAT-KO mice and rats are routinely used in search of potential antipsychotic drugs [39,40]. As has been demonstrated in previous studies, TAAR1 agonists are able to mitigate locomotor hyperactivity induced by the lack of DAT [10,38,39]. Thus, we next evaluated the effects of LK00764 (1–10 mg/kg, i.p.) on spontaneous locomotor hyperactivity of DAT-KO rats (n = 9).

**Figure 6:**
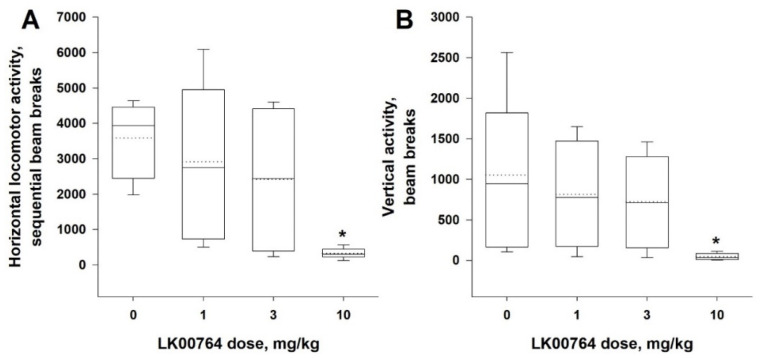
Effect of compound **62** (LK00764) locomotor activity of DAT-KO rats. Administration of compound **62** (LK00764, 1–10 mg/kg, i.p.) decreased both horizontal (**A**) and vertical activity (**B**) of DAT-KO rats. Data are represented as medians (solid lines), means (dotted lines), and 25th and 75th percentiles in a box-whisker plot, with whiskers indicating 10th and 90th percentiles. * *p* < 0.05 (Dunnett’s test) compared to vehicle-treated rats (n = 9 per group).

We found that the administration of compound **62** (LK00764) was accompanied by a significant dose-dependent decrease in both horizontal (the main effect of the factor ‘dose’: χ^2^ = 9.69, df = 4, *p* < 0.05) and vertical (the main effect of the factor ‘dose’: χ^2^ = 9.69, df = 4, *p* < 0.05) activity. The Dunnett’s test revealed that only the highest tested dose, 10 mg/kg, significantly (*p* < 0.05) diminished locomotor hyperactivity of the mutants (Figure 6A,B).

#### 3.4.3. Effects on Stress-Induced Hyperthermia in Rats

**Figure 7:**
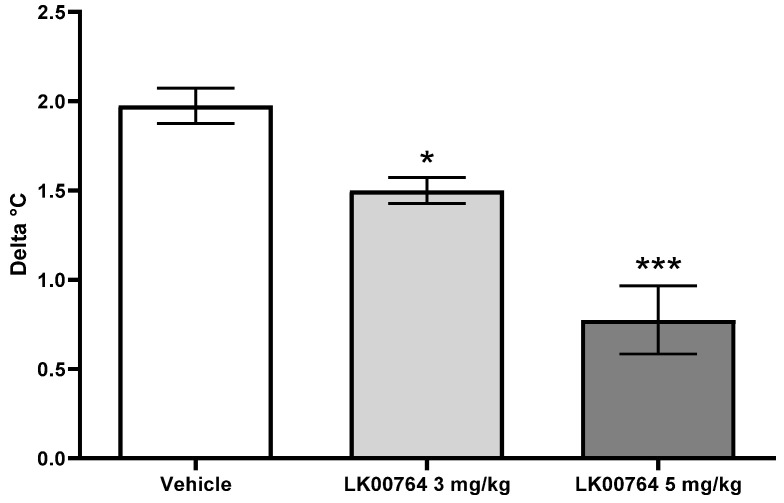
Effect of compound **62** (LK00764) on stress-induced hyperthermia in rats (3 and 5 mg/kg, i.p., n = 8 per group); * *p* < 0.05, *** *p* < 0.05, Dunn’s test.

Another behavioral paradigm known to be affected by TAAR1 agonists is the stress-induced hyperthermia test, which is believed to be a sensitive approach to evaluate the anxiolytic action of compounds [10, 42, 39a]. Thus, compound **62** (LK00764) was evaluated in this test at doses of 3 and 5 mg/kg, i.p. (Figure 7). At both doses, this compound was effective in suppressing stress-induced hyperthermia in rats (*p* < 0.05 and *p* < 0.001 for 3 and 5 mg/kg, respectively; Dunn’s test).

## 4. Conclusions

TAAR1 agonism is emerging as a principally new treatment option for a number of mental conditions, including schizophrenia [7]. Particularly intriguing is the recently demonstrated ability of the TAAR1 agonist Ulotaront to exert potent antipsychotic activity without blocking D_2_ dopamine receptors, thereby avoiding common side-effects of D_2_ dopamine receptor antagonists [8,9]. Here, we have reported the identification of the novel potent TAAR1 agonist compound **62** (LK00764) with pronounced in vivo activity. Compound **62** (LK00764) demonstrated efficacy in rats through the use of four behavioral tests typically employed in the screening of antipsychotic drugs and known to demonstrate a response to TAAR1 agonists. Further preclinical studies, including the comparison of the pharmacodynamic effects of compound **62** to those of the most advanced TAAR1 agonists as well as classical antipsychotics, are necessary to evaluate the efficacy, safety, and tolerability of this potent and efficacious TAAR1 agonist for the potential development of this compound as a new pharmacotherapy option for schizophrenia and other psychiatric disorders.

## Figures and Tables

**Table 1:** Agonistic activity of 2-(4*H*-1,2,4-triazol-3-yl)ethan-1-amine analogs **6**–**57** of screening hit compounds **1**–**2** with respect to TAAR1 receptor *^a^*.

Compound	R	E_max_ *^b^*	EC_50_ (nM)
**6**	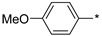	56	>1000
**7**	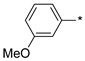	132	910
**8**	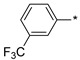	108	210
**9**	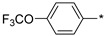	116	310
**10**	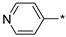	47	>1000
**11**	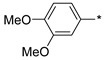	inactive	
**12**	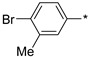	87	812
**13**	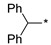	inactive	
**14**	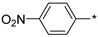	inactive	
**15**	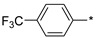	inactive	
**16**	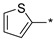	138	>1000
**17**	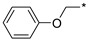	inactive	
**18**	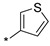	inactive	
**19**	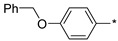	113	26
**20**	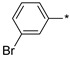	124	218
**21**	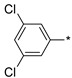	112	23
**22**	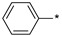	118	283
**23**	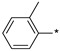	116	468
**24**	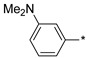	inactive	
**25**	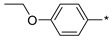	139	550
**26**	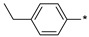	71	>1000
**27**	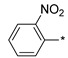	69	>1000
**28**	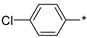	107	522
**29**	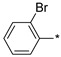	111	696
**30**	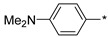	122	730
**31**	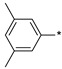	117	813
**32**	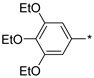	inactive	
**33**	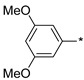	55	>1000
**34**	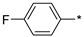	169	>1000
**35**	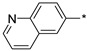	41	>1000
**36**	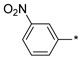	110	440
**37**	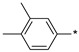	70	>1000
**38**	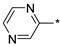	inactive	
**39**	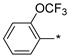	inactive	
**40**	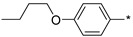	107	34
**41**	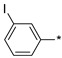	80	890
**42**	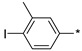	76	>1000
**43**	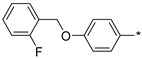	114	45
**44**	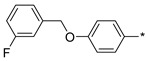	112	30
**45**	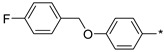	114	22
**46**	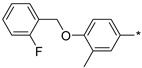	82	398
**47**	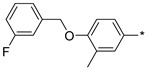	106	142
**48**	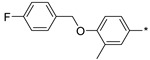	98	163
**49**	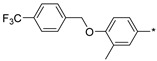	inactive	
**50**	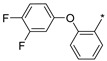	inactive	
**51**	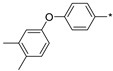	77	900
**52**	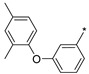	79	>1000
**53**	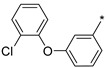	71	847
**54**	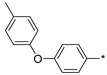	94	150
**55**	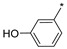	88	>1000
**56**	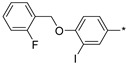	87	316
**57**	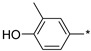	90	>1000

*^a^* Mean from 3 different BRET assays (errors were in the range of ±5–10% of the reported values). *^b^* Maximum observed activation of TAAR1 relative to that observed for 1 μM of β-PEA.

**Table 2:** Agonistic activity of biphenyl analogs **58**–**67** of screening hit compounds **1**–**2** with respect to TAAR1 receptor *^a^*.

Compound	R	E_max_ *^b^*	EC_50_ (nM)
**58**	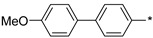	97	6
**59**	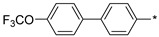	95	13
**60**	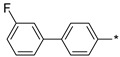	95	26
**61**	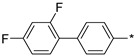	93	18
**62** (LK00764)	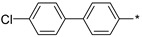	101	4
**63**	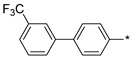	76	250
**64**	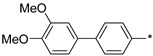	104	185
**65**	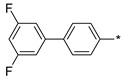	105	13
**66**	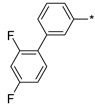	91	300
**67**	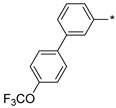	94	96

*^a^* Mean from 3 different BRET assays (errors were in the range of ±5–10% of the reported values). *^b^* Maximum observed activation of TAAR1 relative to that observed for 1 μM of β-PEA.

## Data Availability

All data are available from the corresponding authors on request.

## Supplementary Materials

The following supporting information can be downloaded at: https://www.mdpi.com/article/10.3390/biom12111650/s1: Copies of ^1^H and ^13^C NMR spectra.
