# Supplementary material for: Discovery of Trace Amine-Associated Receptor 1 (TAAR1) Agonist 2-(5-(4′-Chloro-[1,1′-biphenyl]-4-yl)-4H-1,2,4-triazol-3-yl)ethan-1-amine (LK00764) for the Treatment of Psychotic Disorders
Source: Biomolecules. 2022 Nov 7;12(11):1650. doi: 10.3390/biom12111650 (PMC9687812; doi:10.3390/biom12111650)

Supplementary data for

Discovery of Trace Amine Associated Receptor 1 (TAAR1) Agonist 2-(5-(4'-Chloro-[1,1'-biphenyl]-4-yl)-4H-1,2,4-triazol-3-yl)ethan-1-amine (LK00764) for the Treatment of Psychotic Disorders

Mikhail Krasavin*, Alexey Lukin, Ilya Sukhanov, Andrei Gerasimov, Savelii Kuvarzin, Evgeniya V. Efimova, Mariia Dorofeikova, Anna Nichugovskaya, Andrey Matveev, Kirill Onokhin, Konstantin Zakharov, Maxim Gureev and Raul R. Gainetdinov*

Copies of ^{1}H and ^{13}C NMR spectra 2-64

^{1}H and ^{13}C NMR spectra of compound **4**

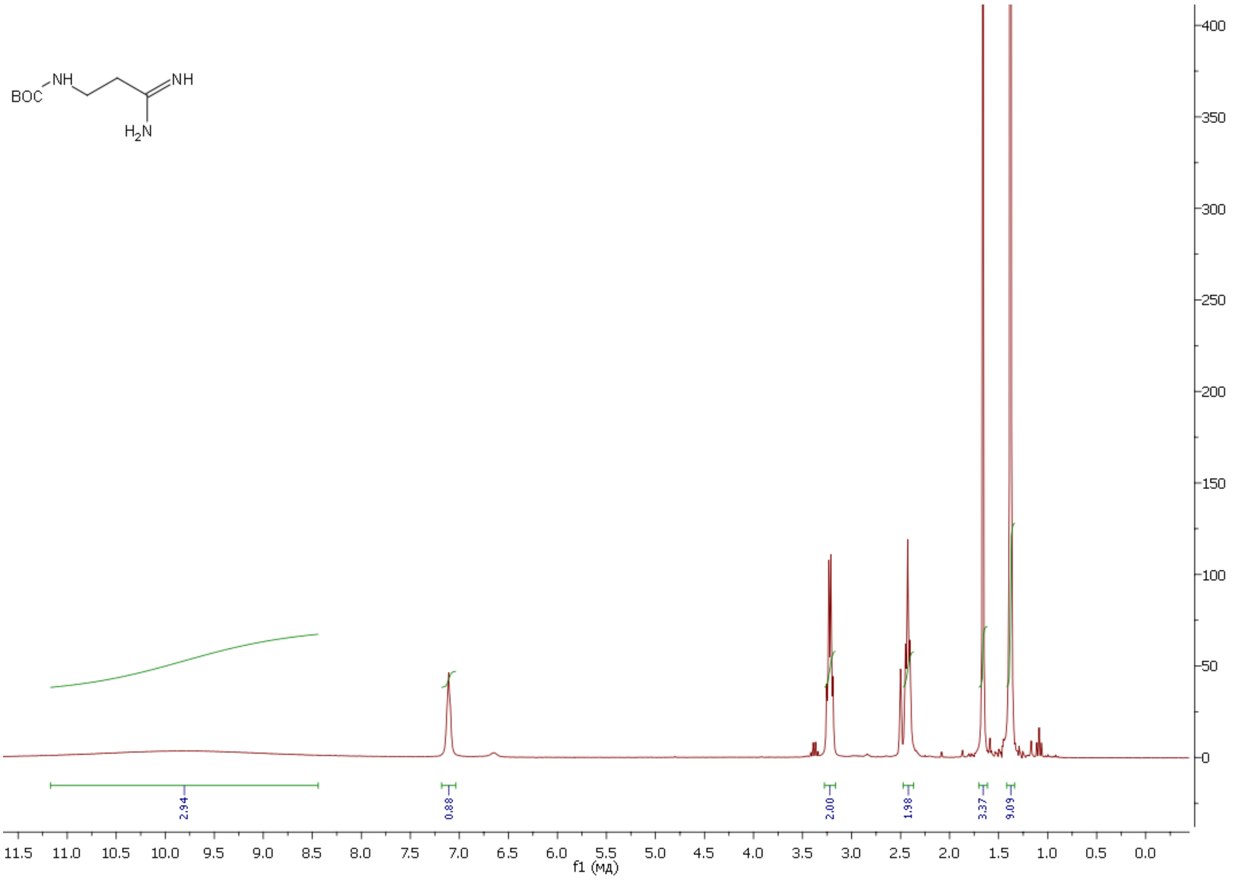

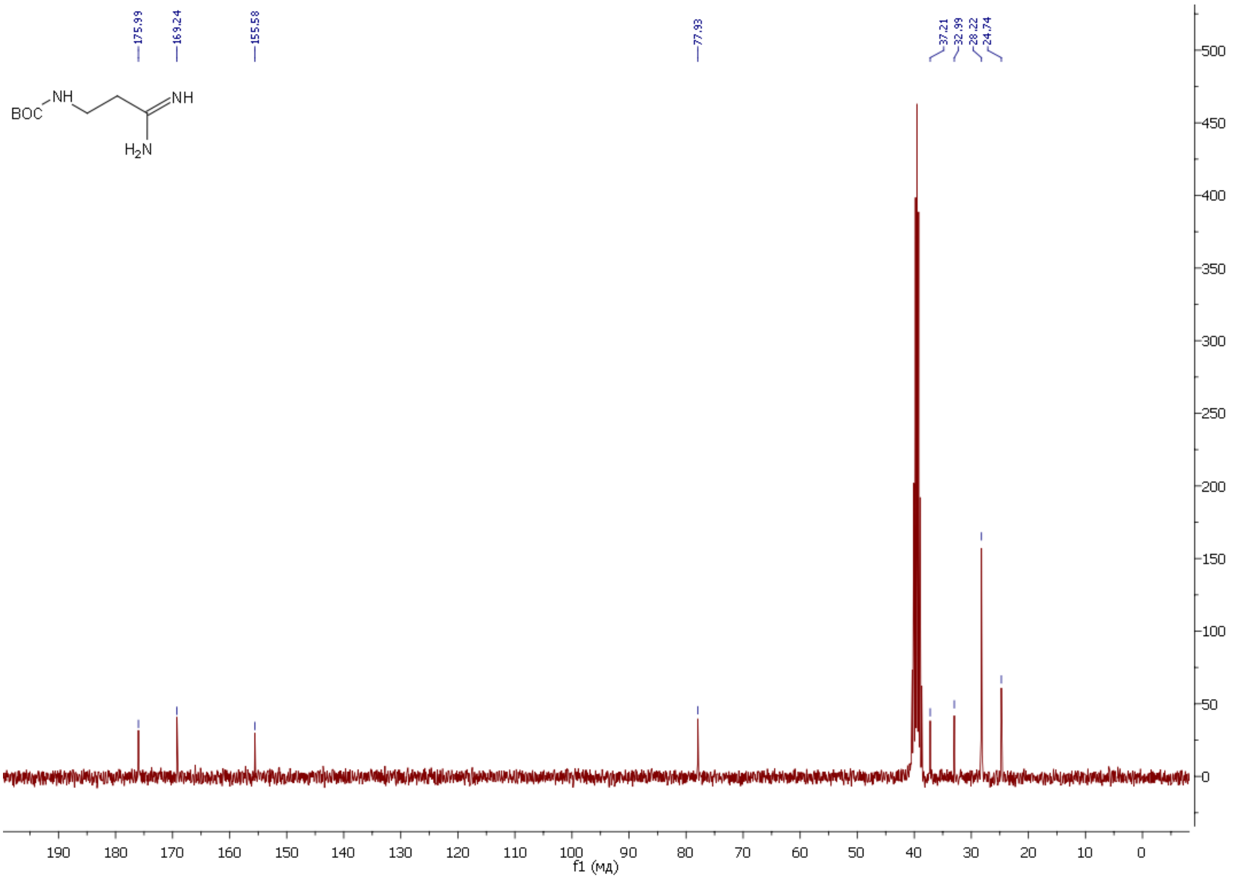

^{1}H and ^{13}C NMR spectra of compound **7**

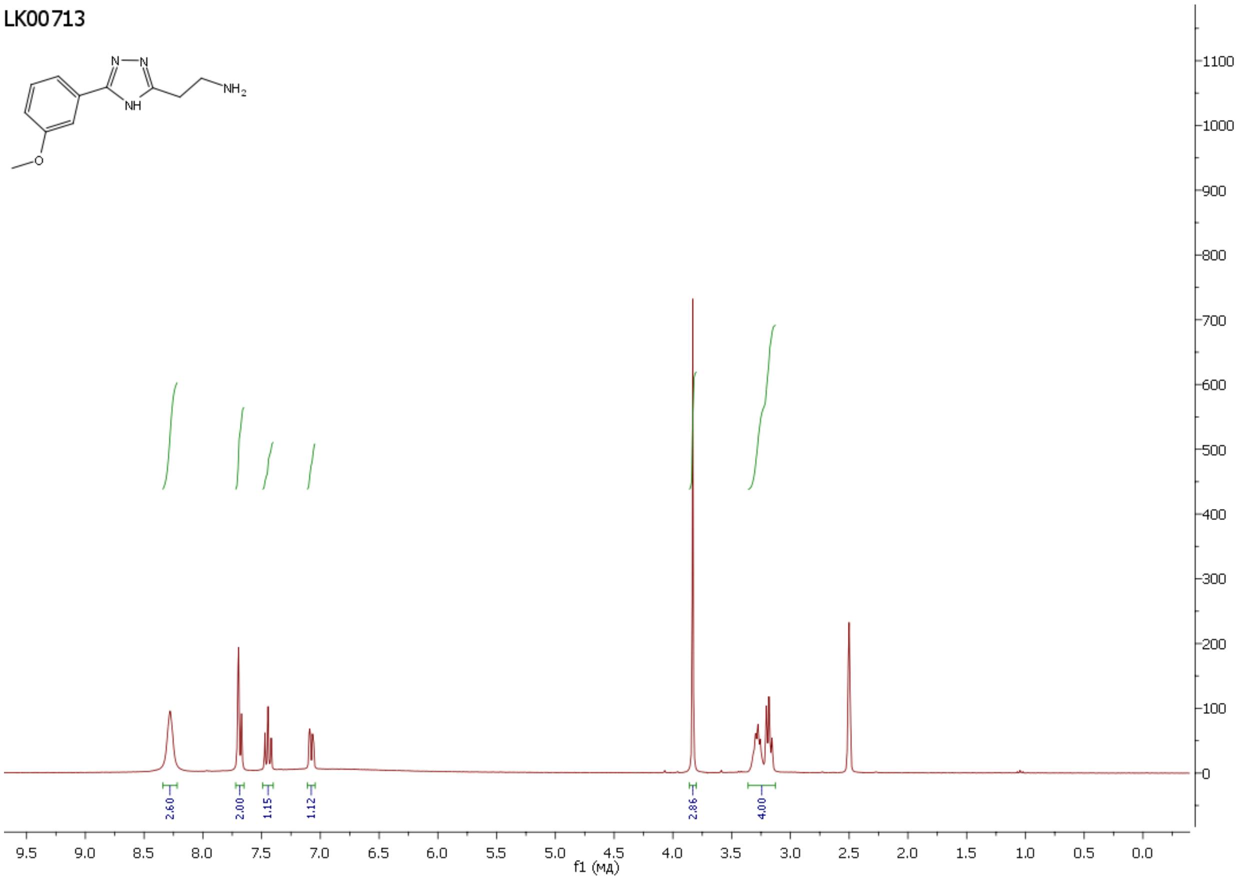

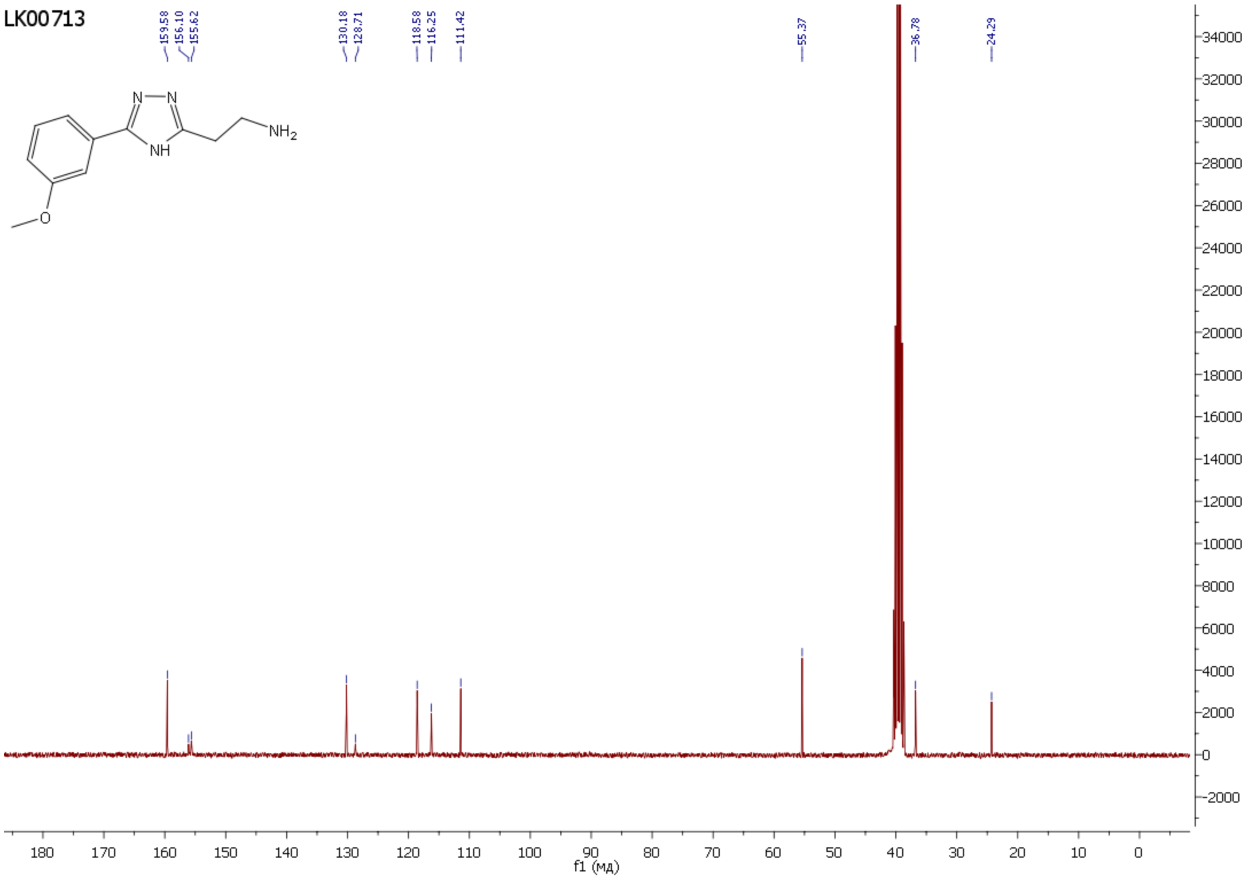

^{1}H and ^{13}C NMR spectra of compound **8**

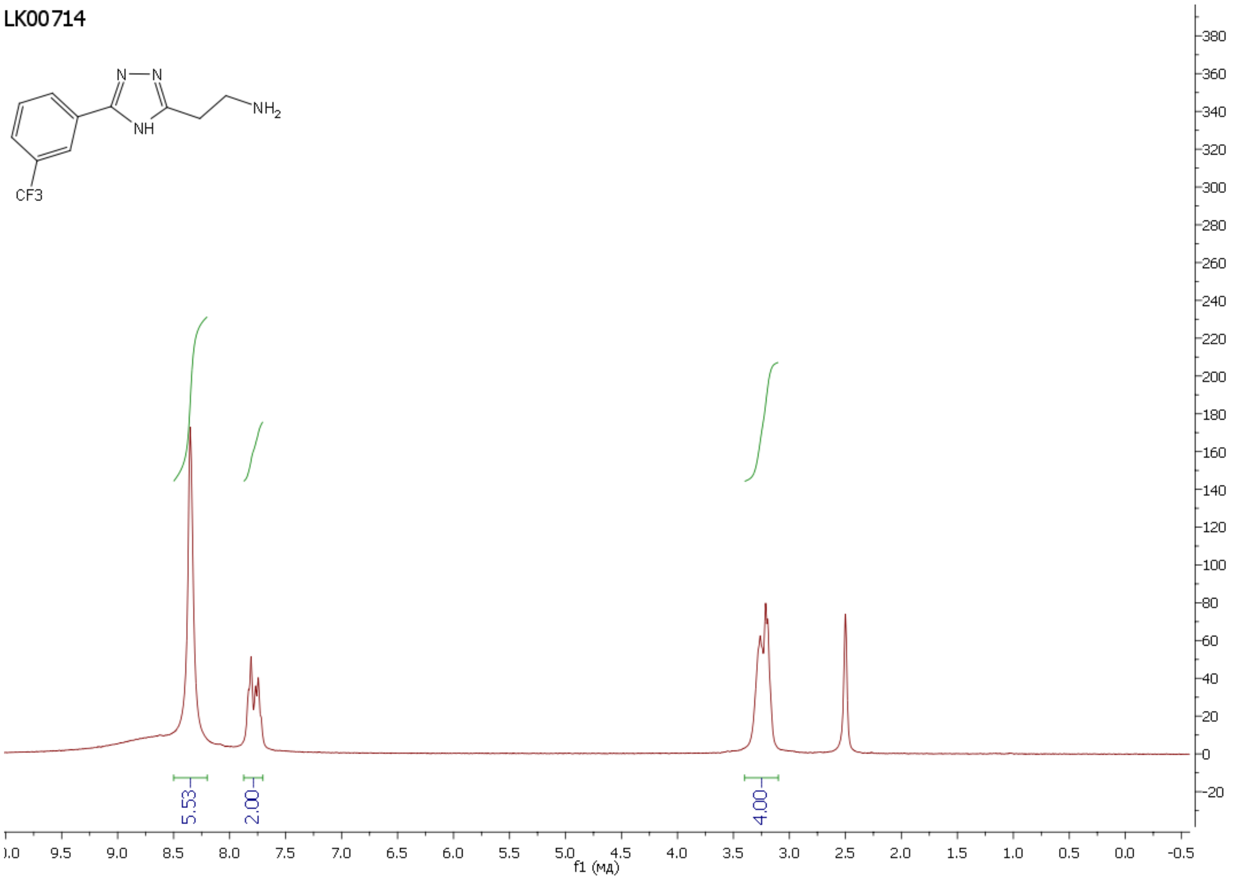

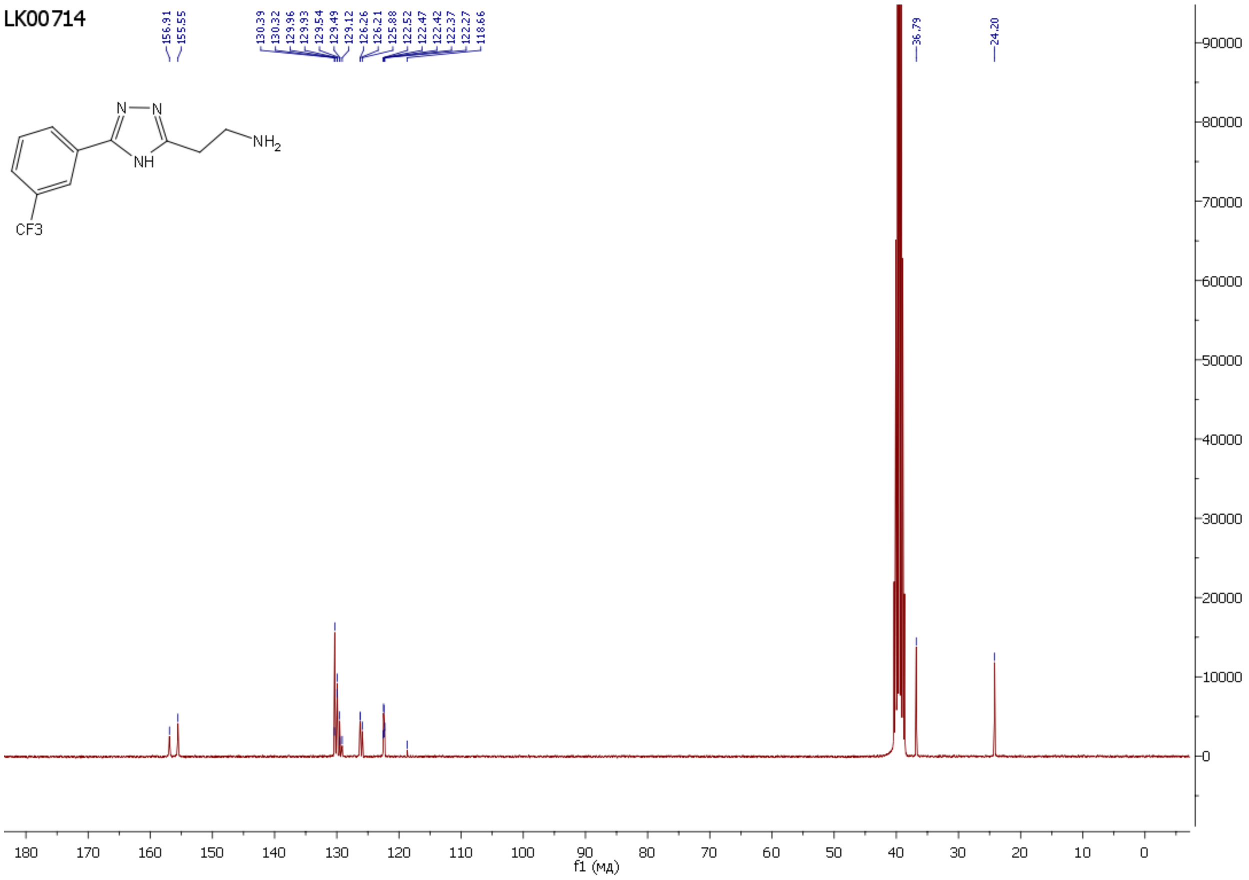

^{1}H and ^{13}C NMR spectra of compound **9**

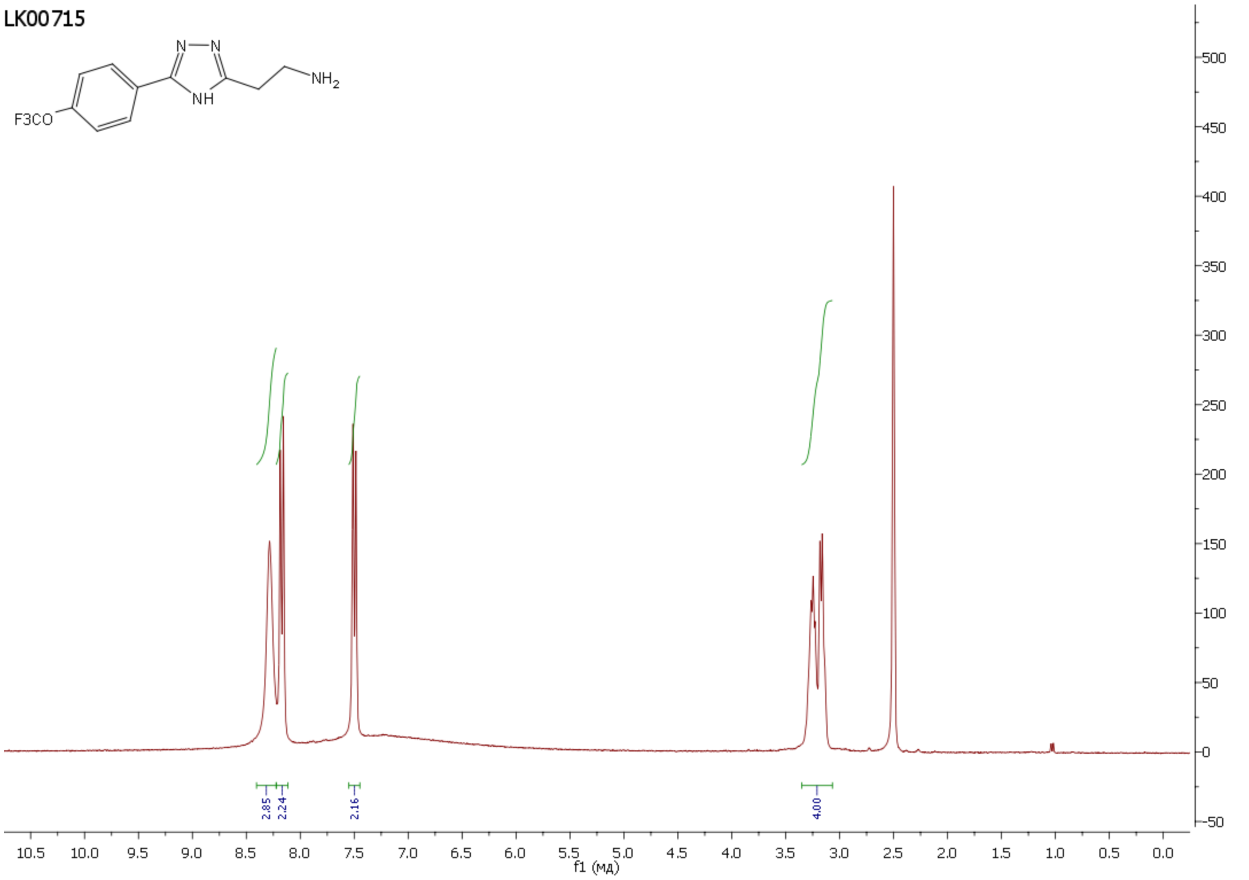

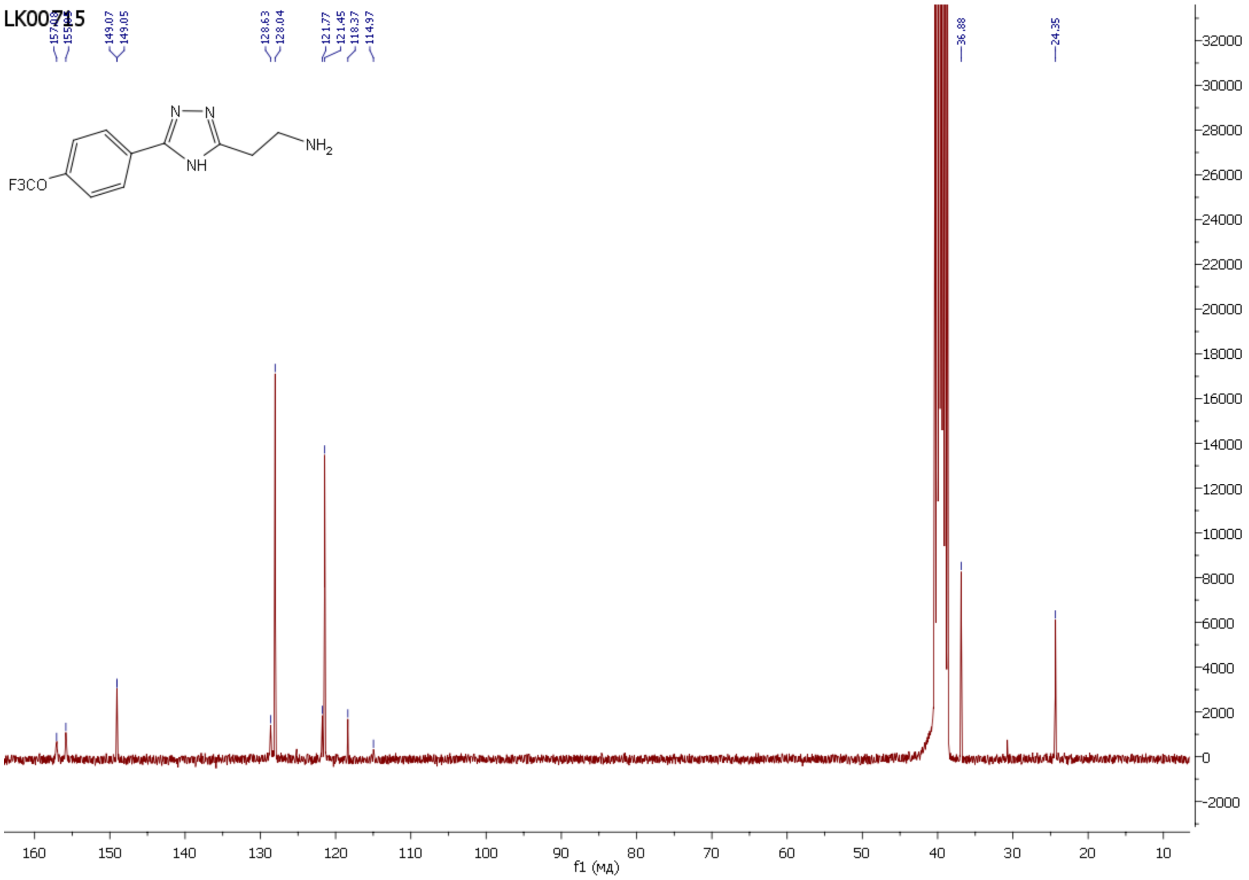

^{1}H and ^{13}C NMR spectra of compound **10**

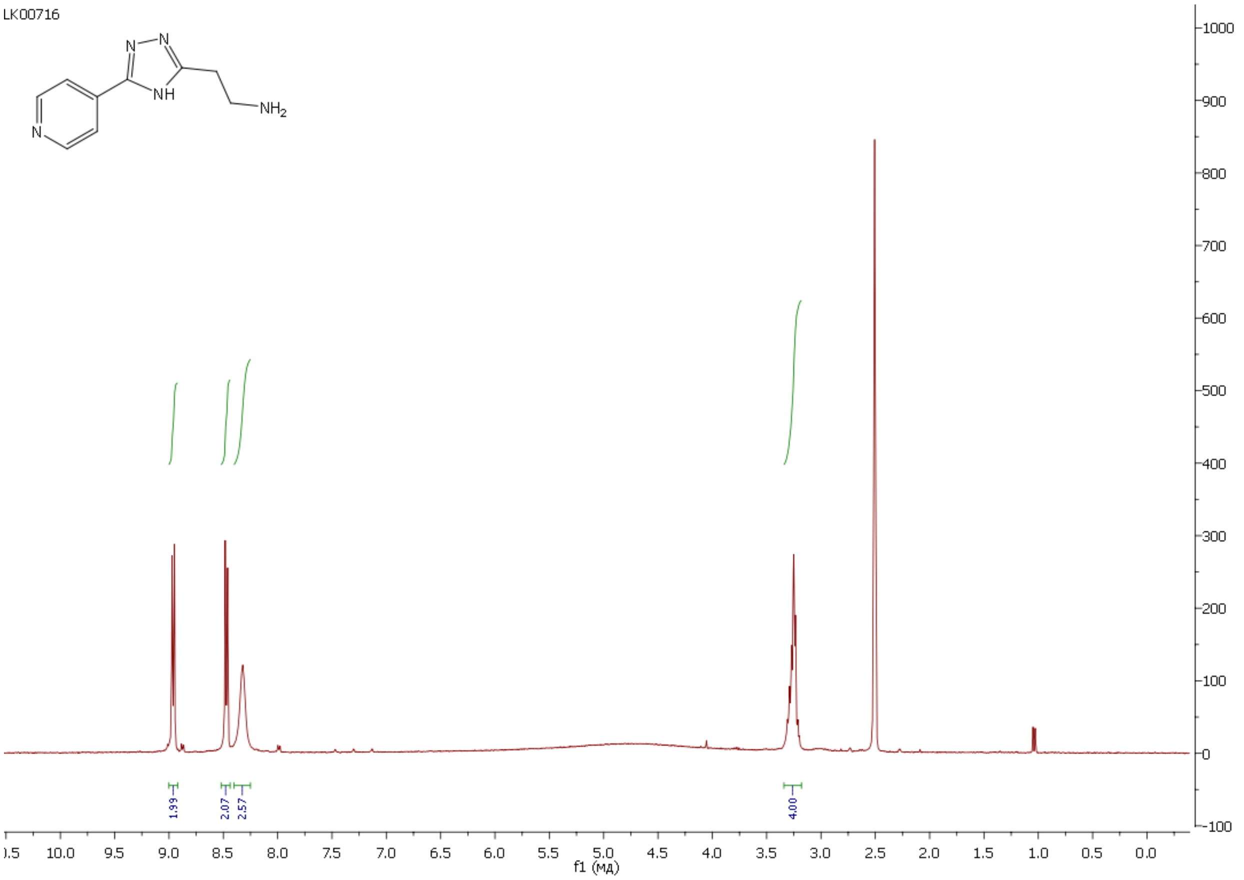

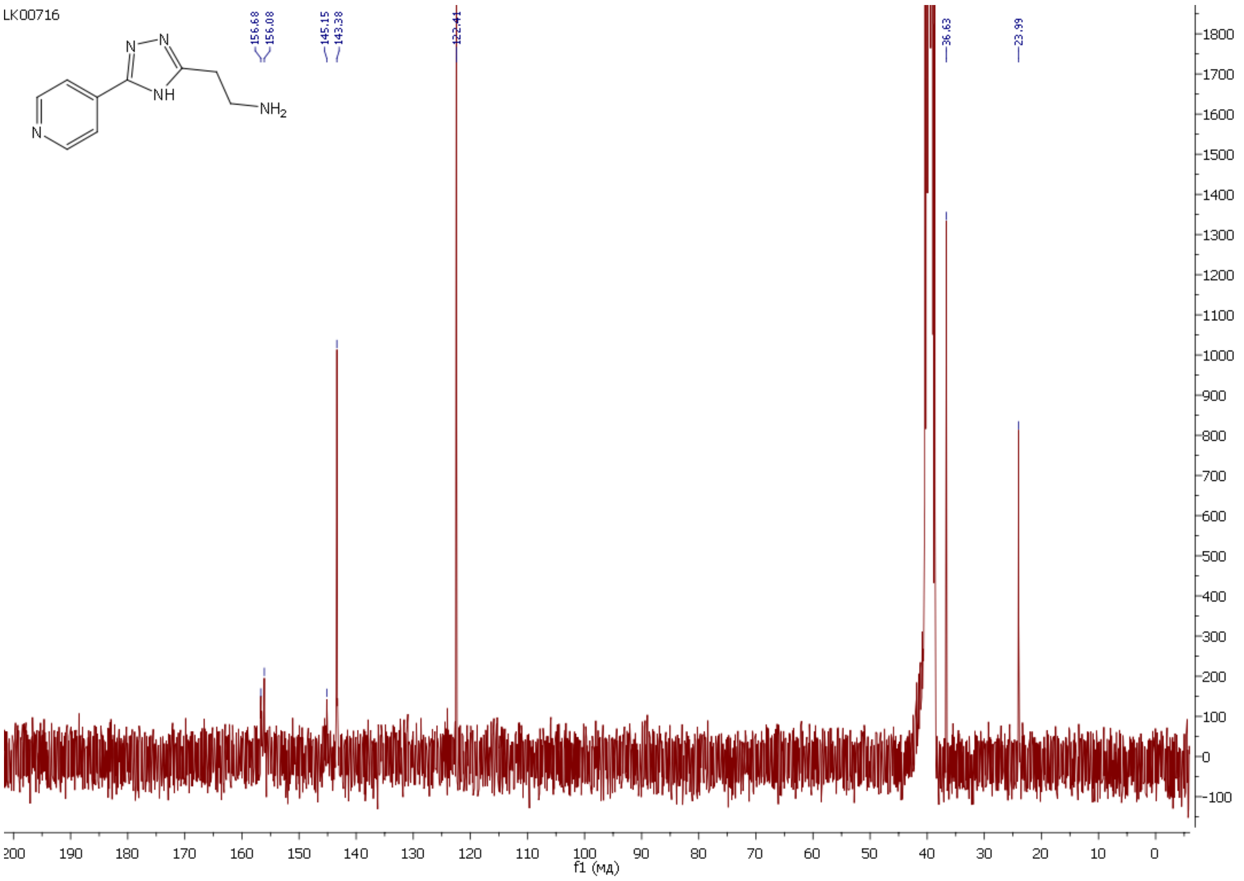

^{1}H and ^{13}C NMR spectra of compound **11**

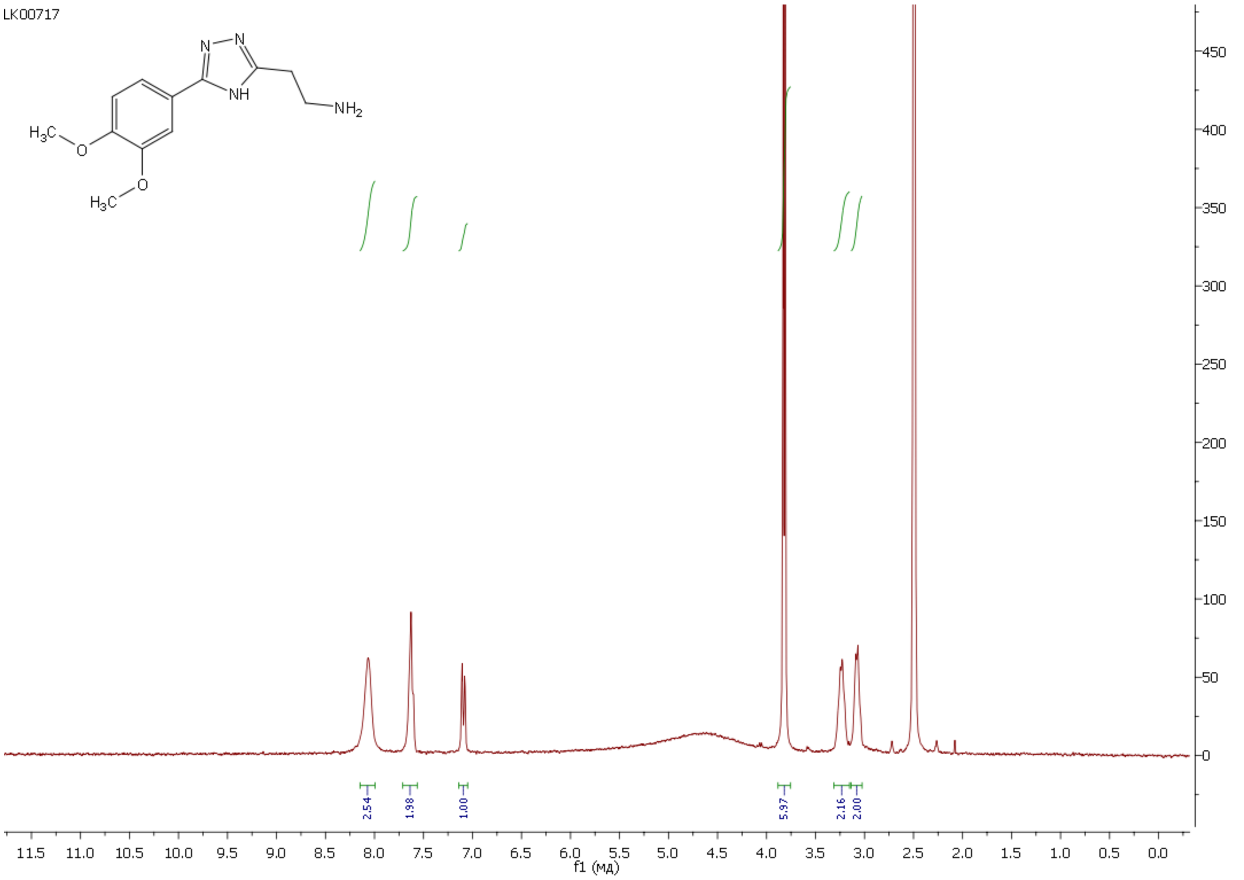

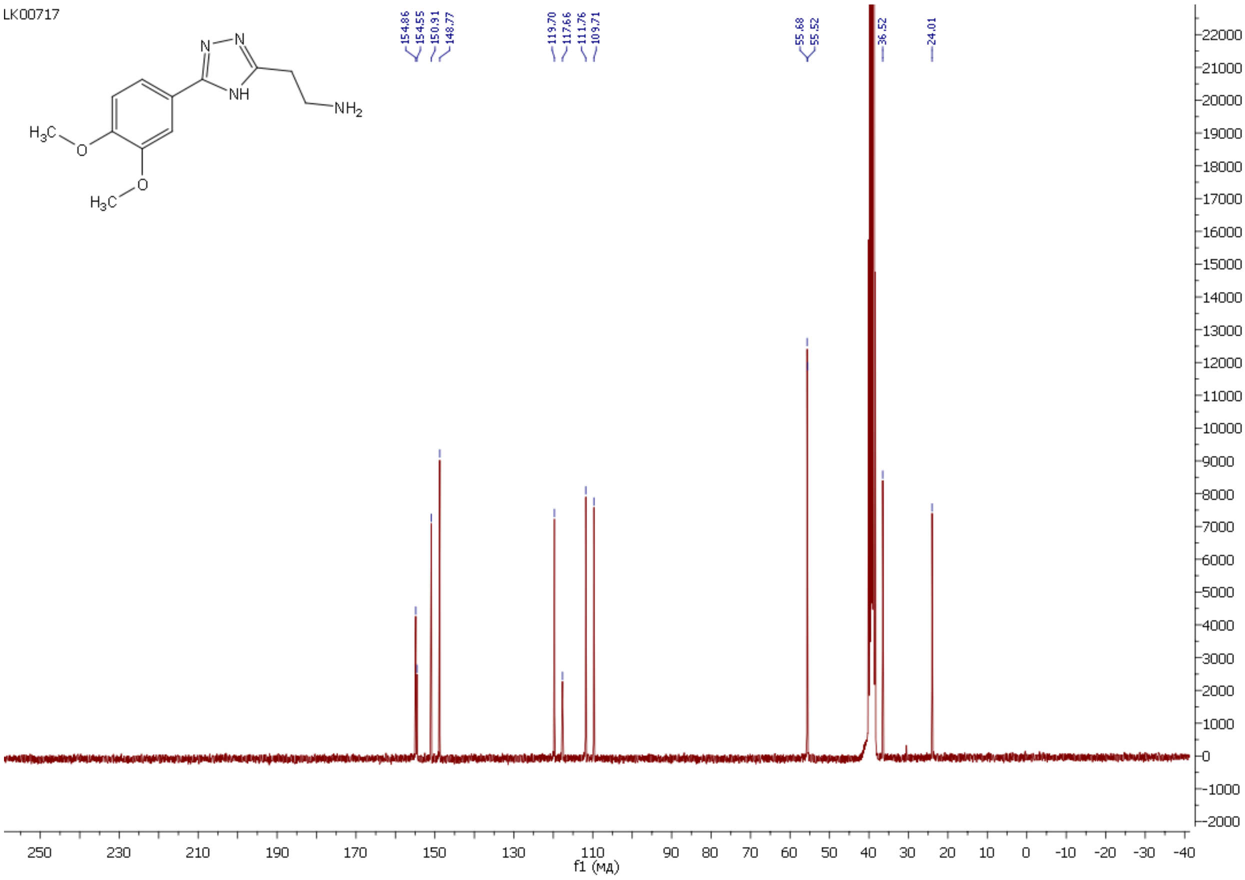

^{1}H and ^{13}C NMR spectra of compound **12**

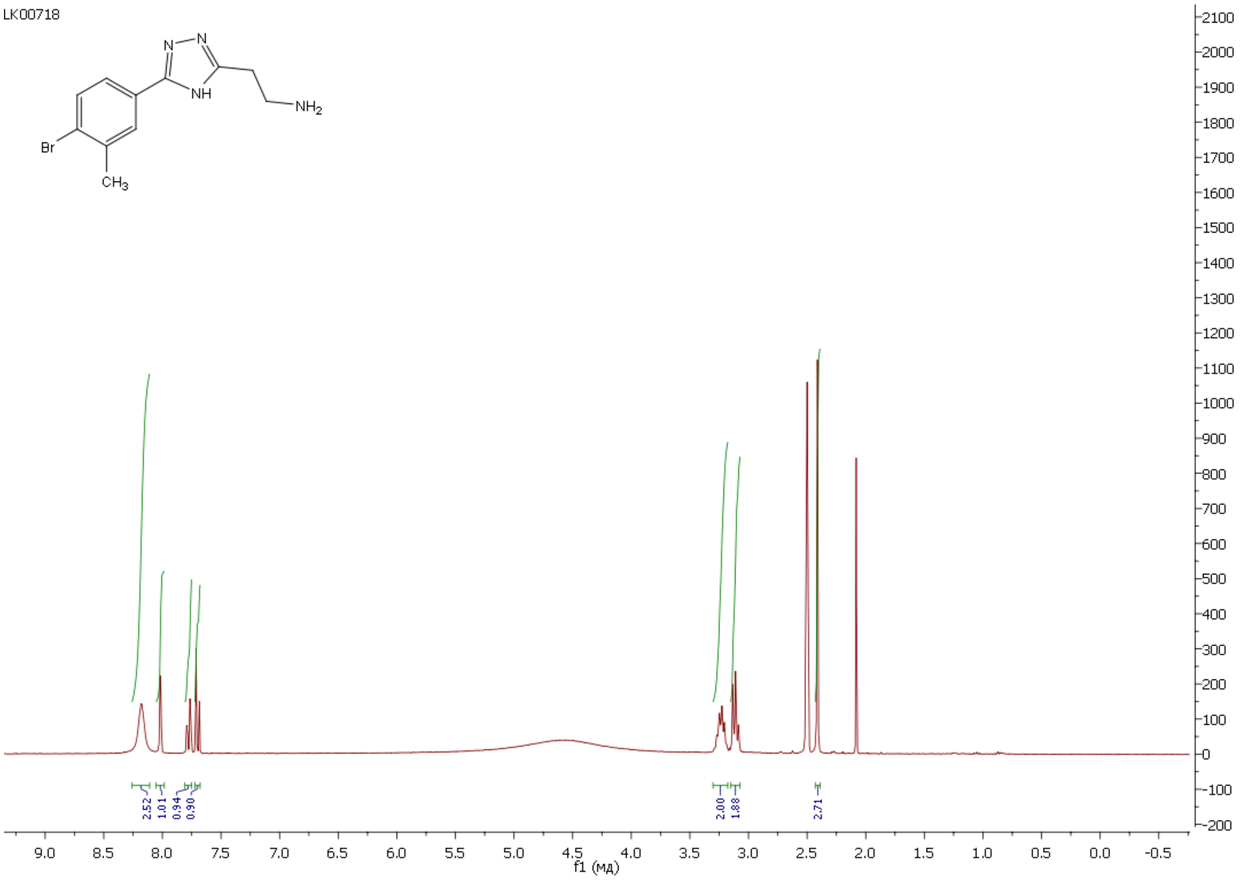

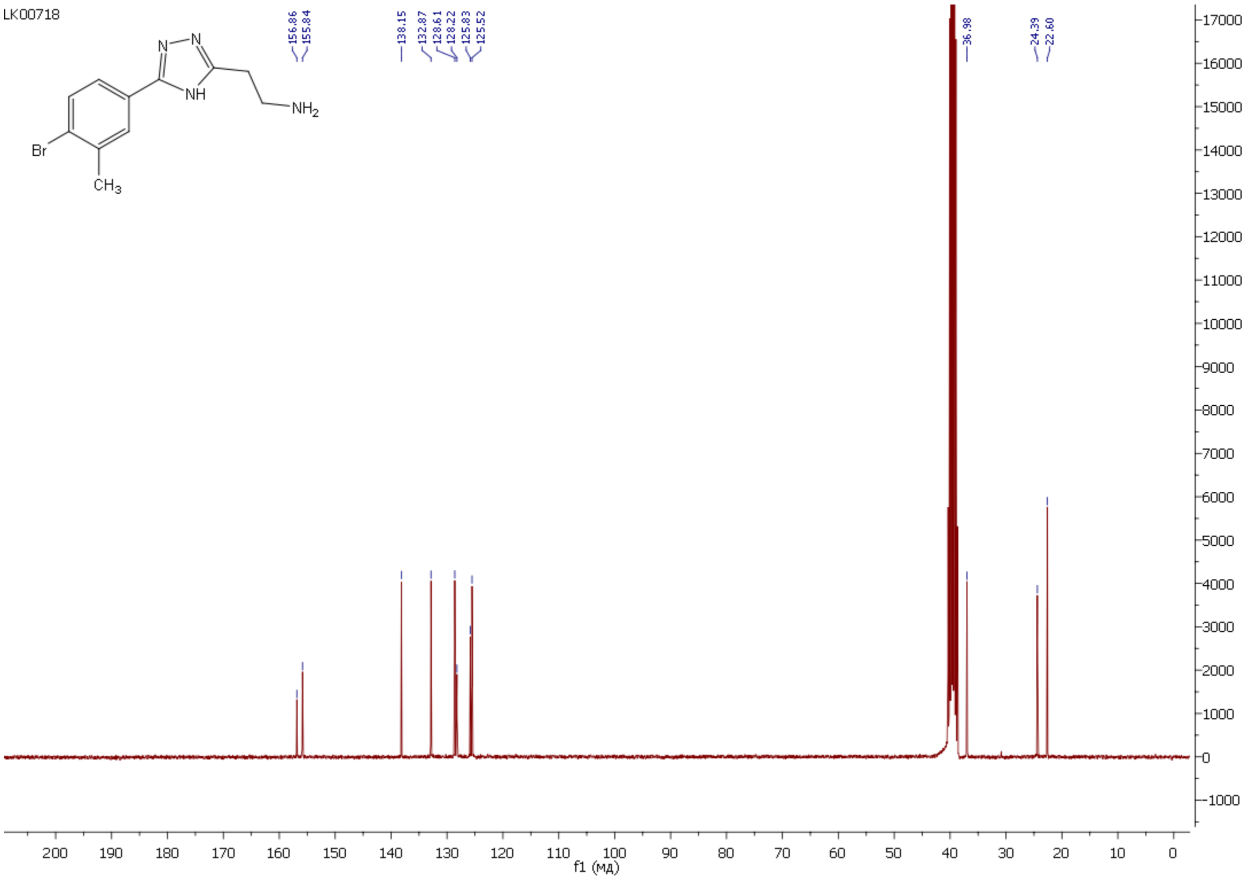

^{1}H and ^{13}C NMR spectra of compound **13**

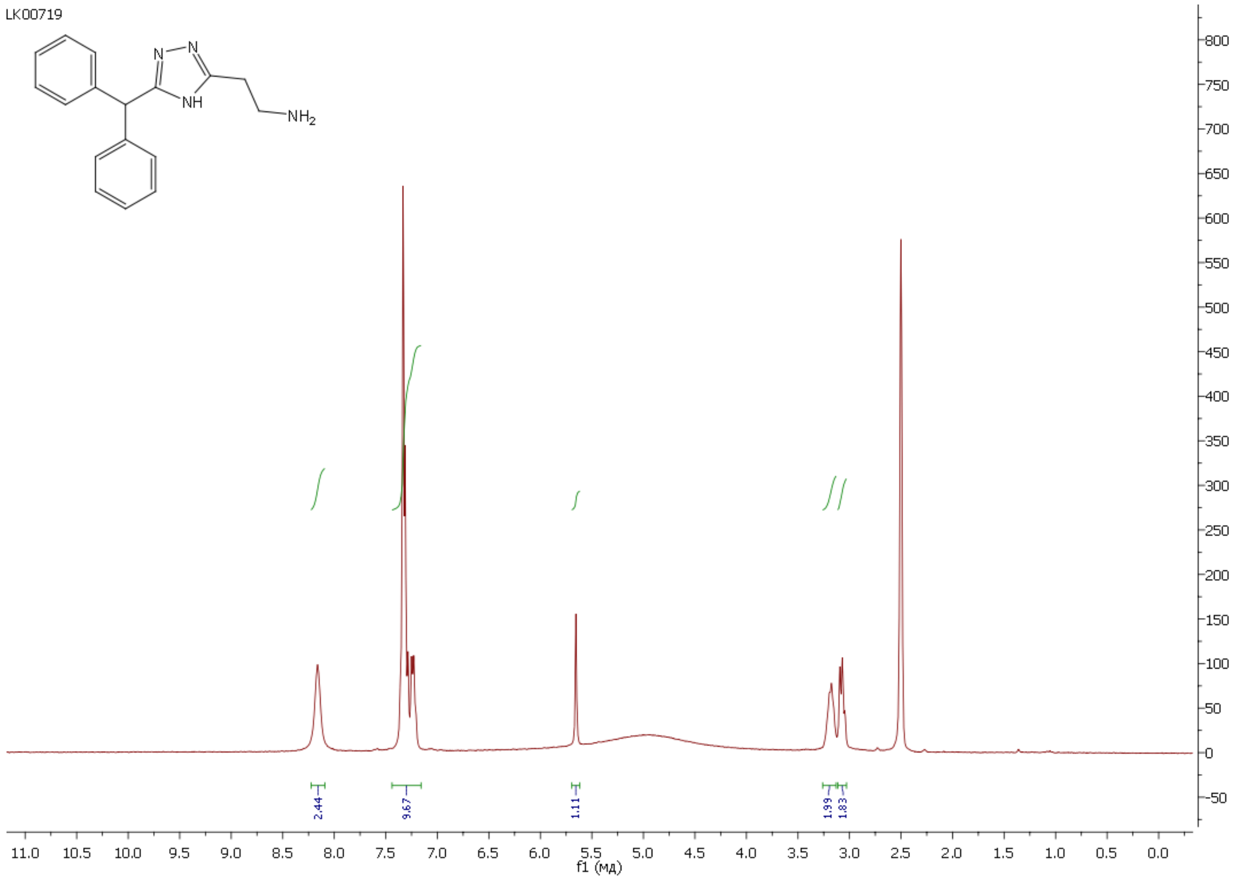

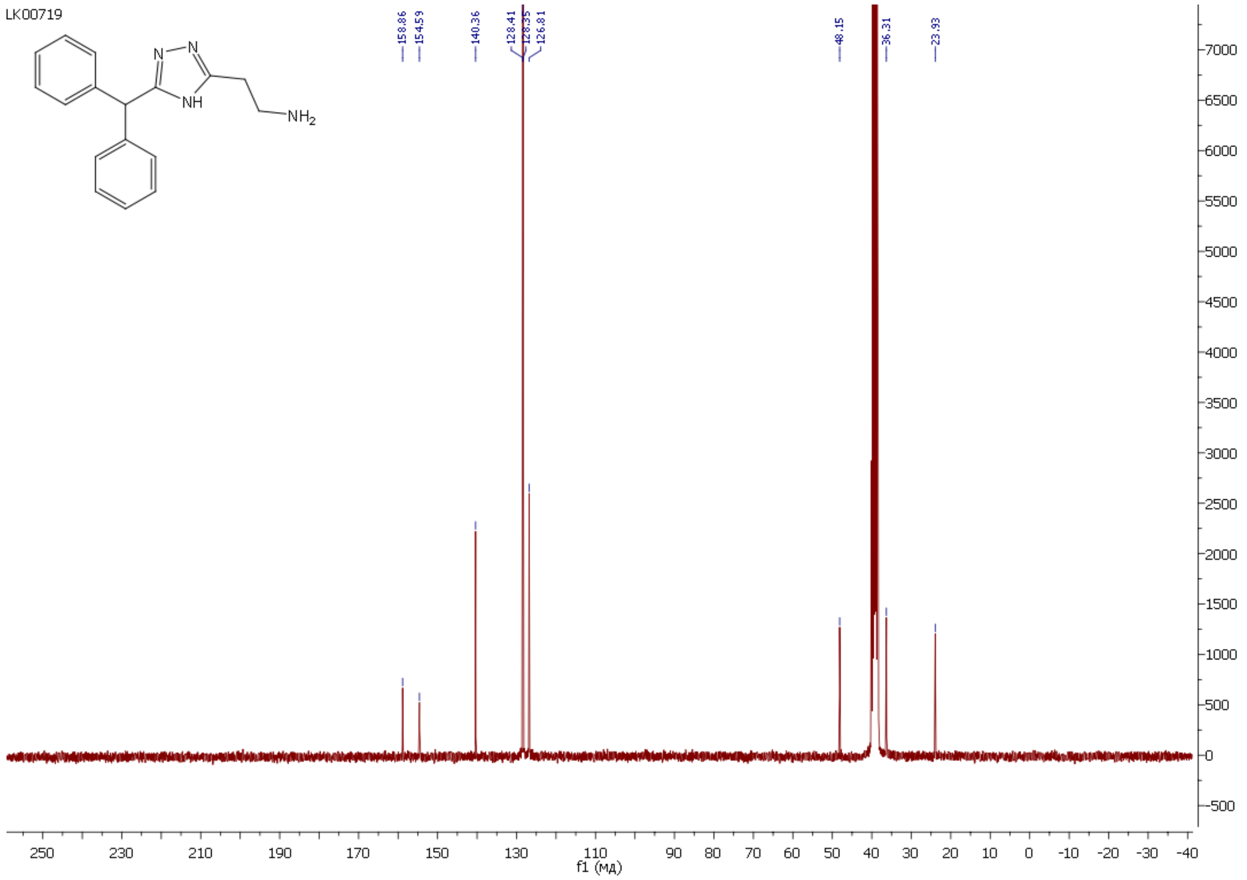

^{1}H and ^{13}C NMR spectra of compound **14**

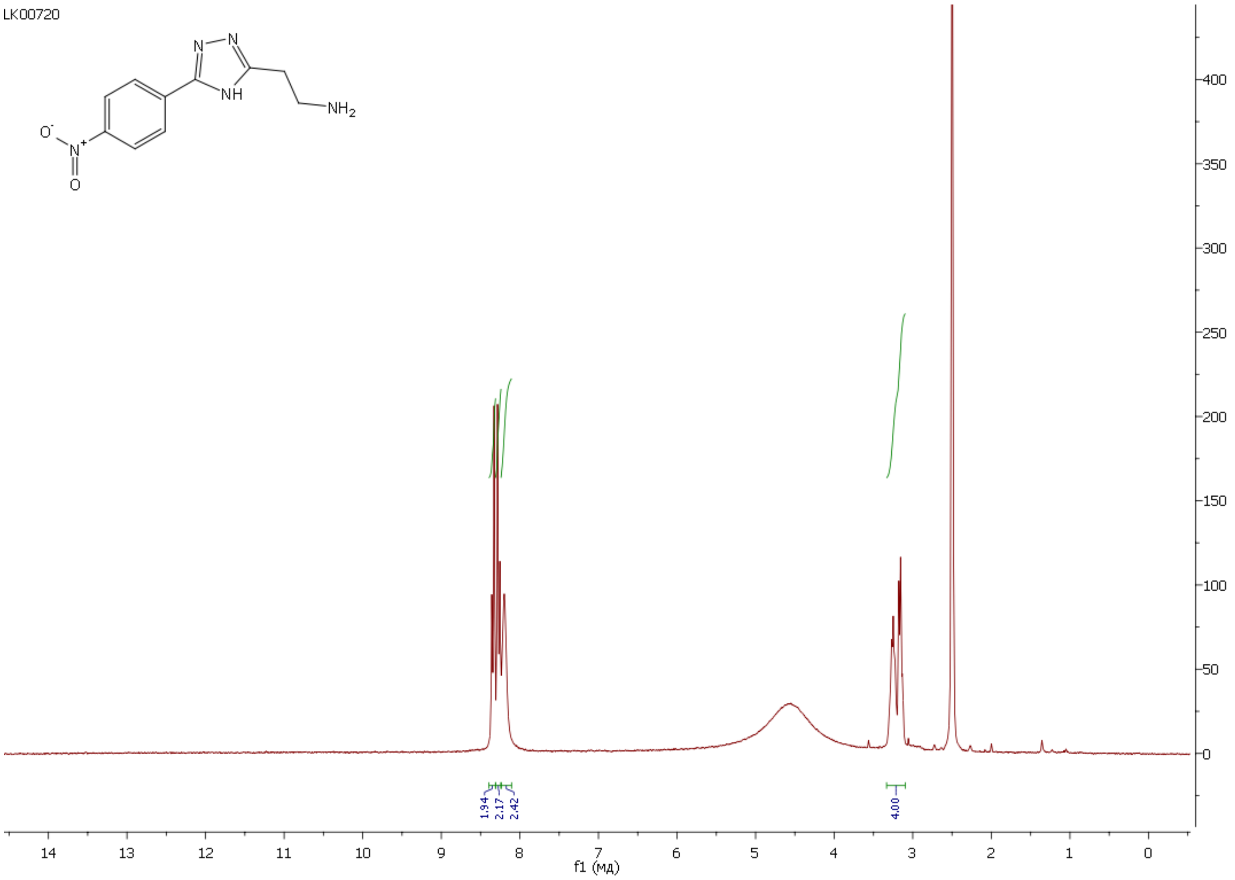

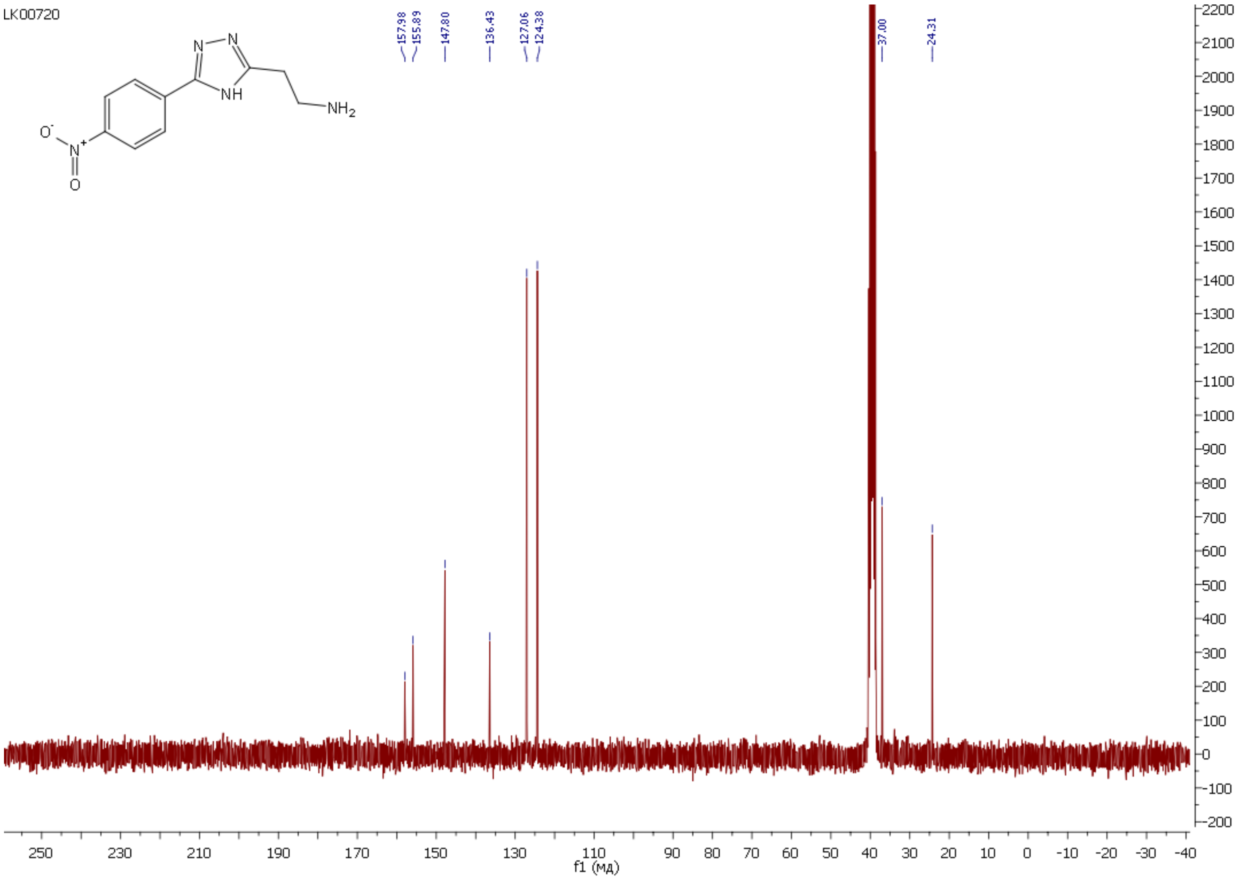

^{1}H and ^{13}C NMR spectra of compound **15**

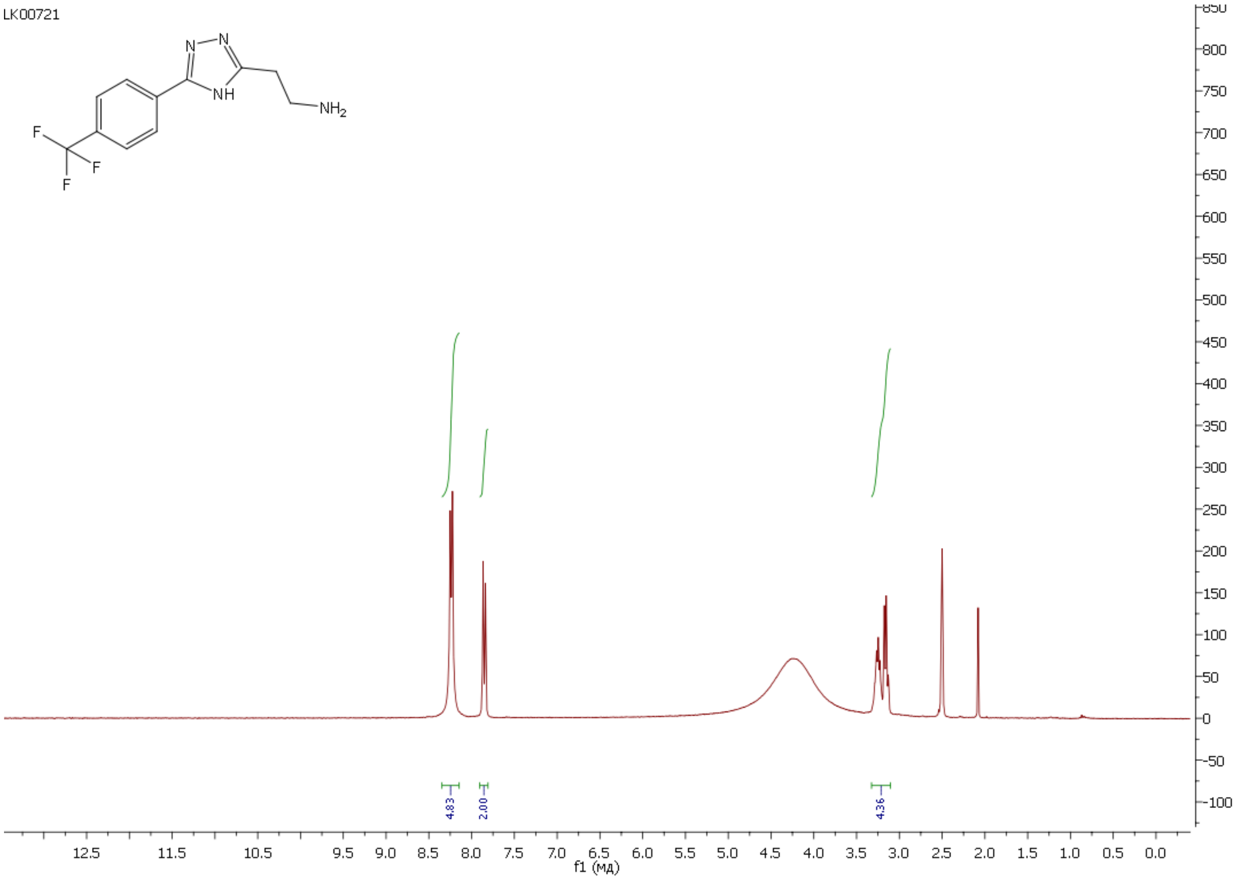

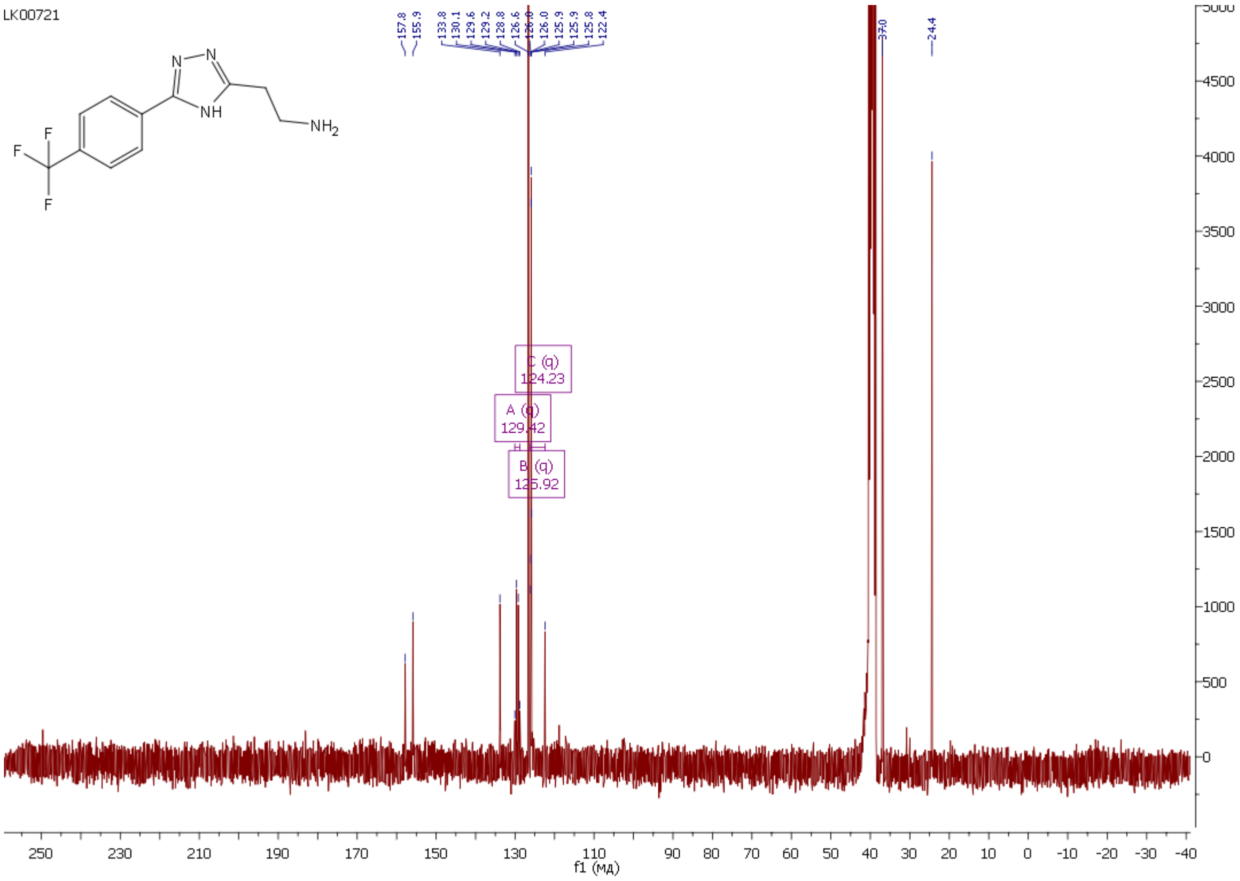

^{1}H and ^{13}C NMR spectra of compound **16**

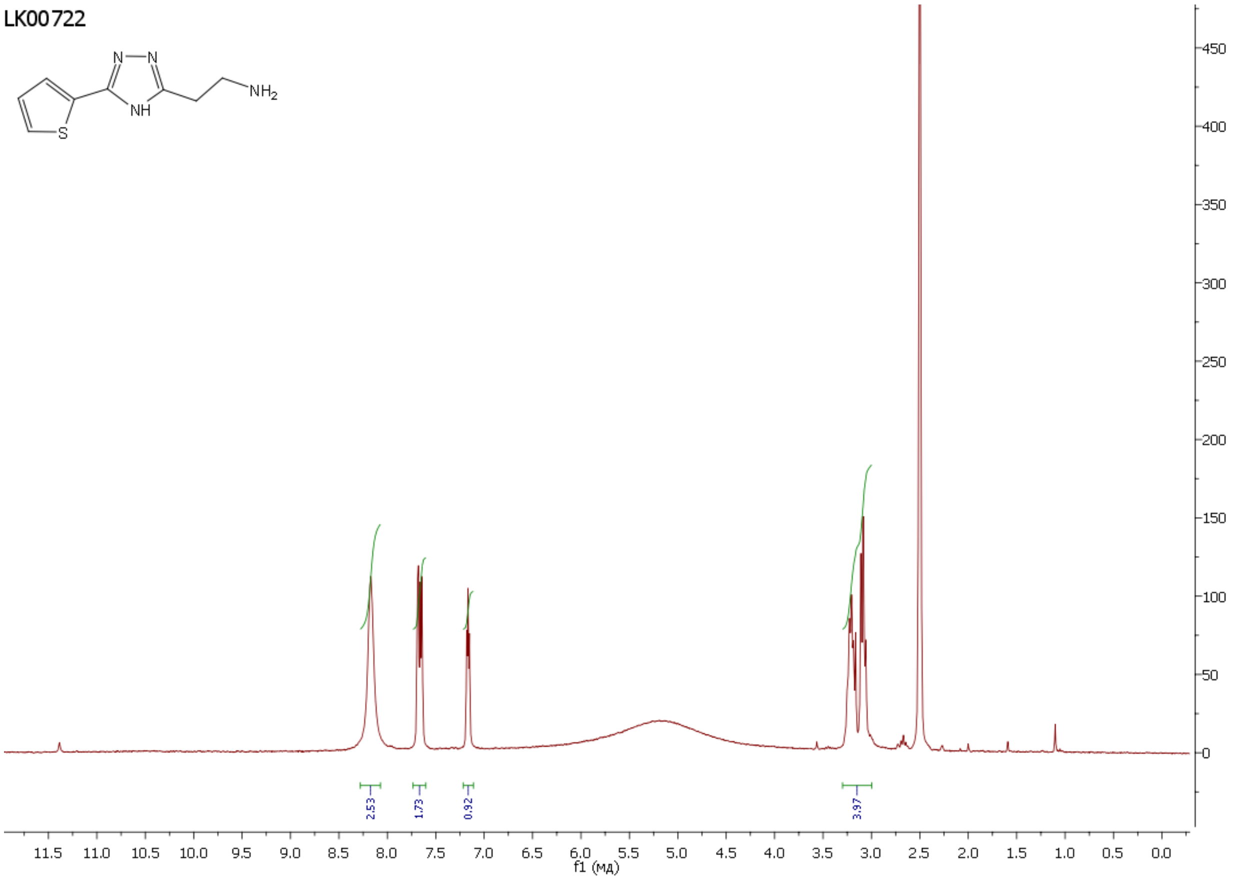

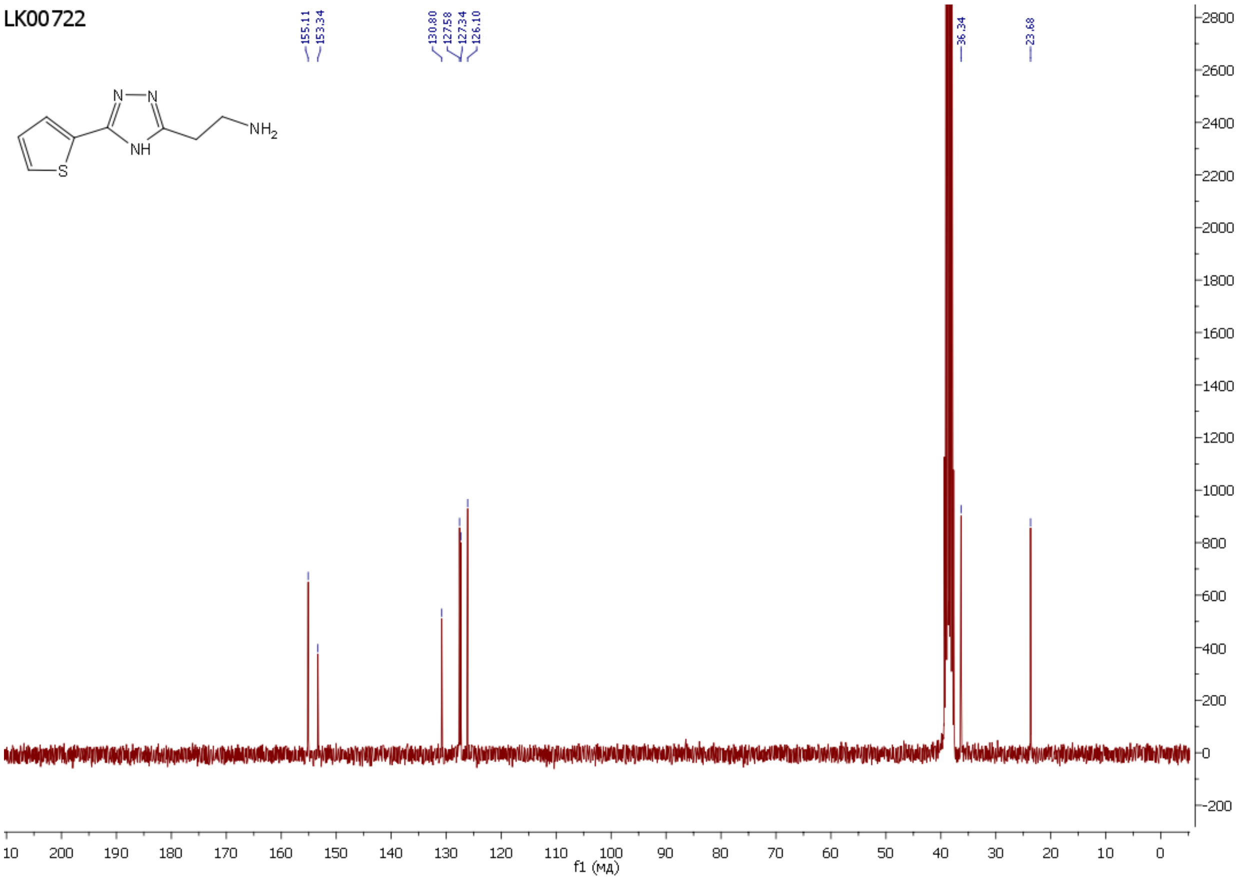

^{1}H and ^{13}C NMR spectra of compound **17**

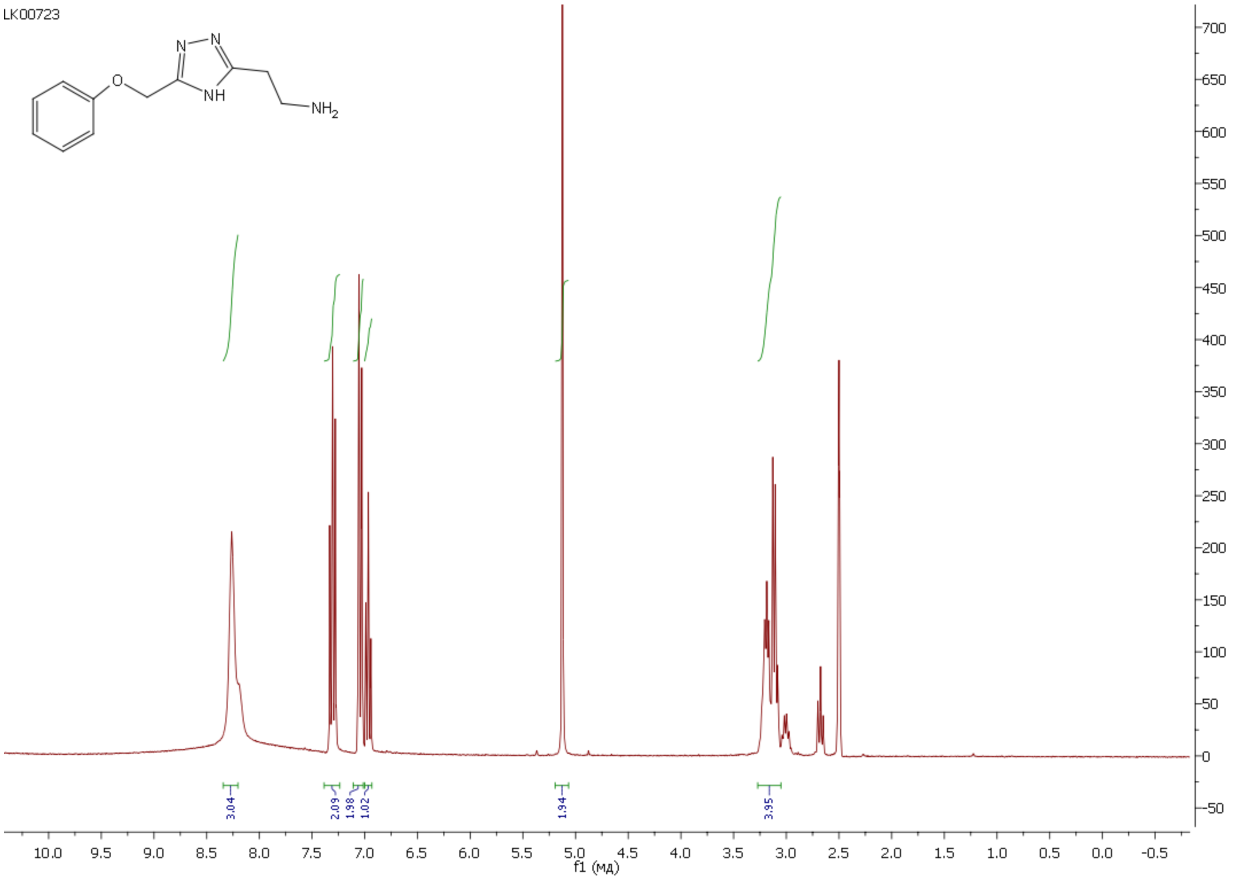

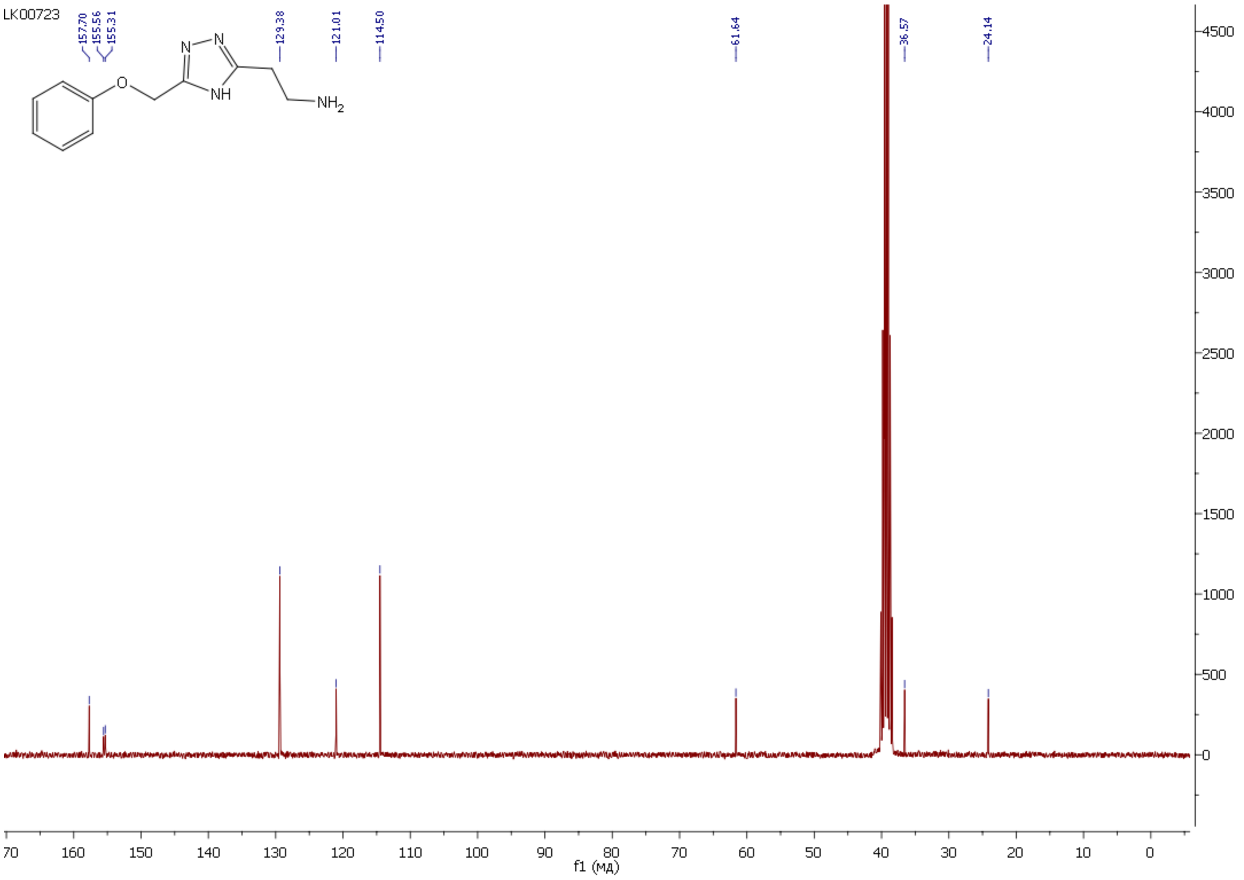

^{1}H and ^{13}C NMR spectra of compound **18**

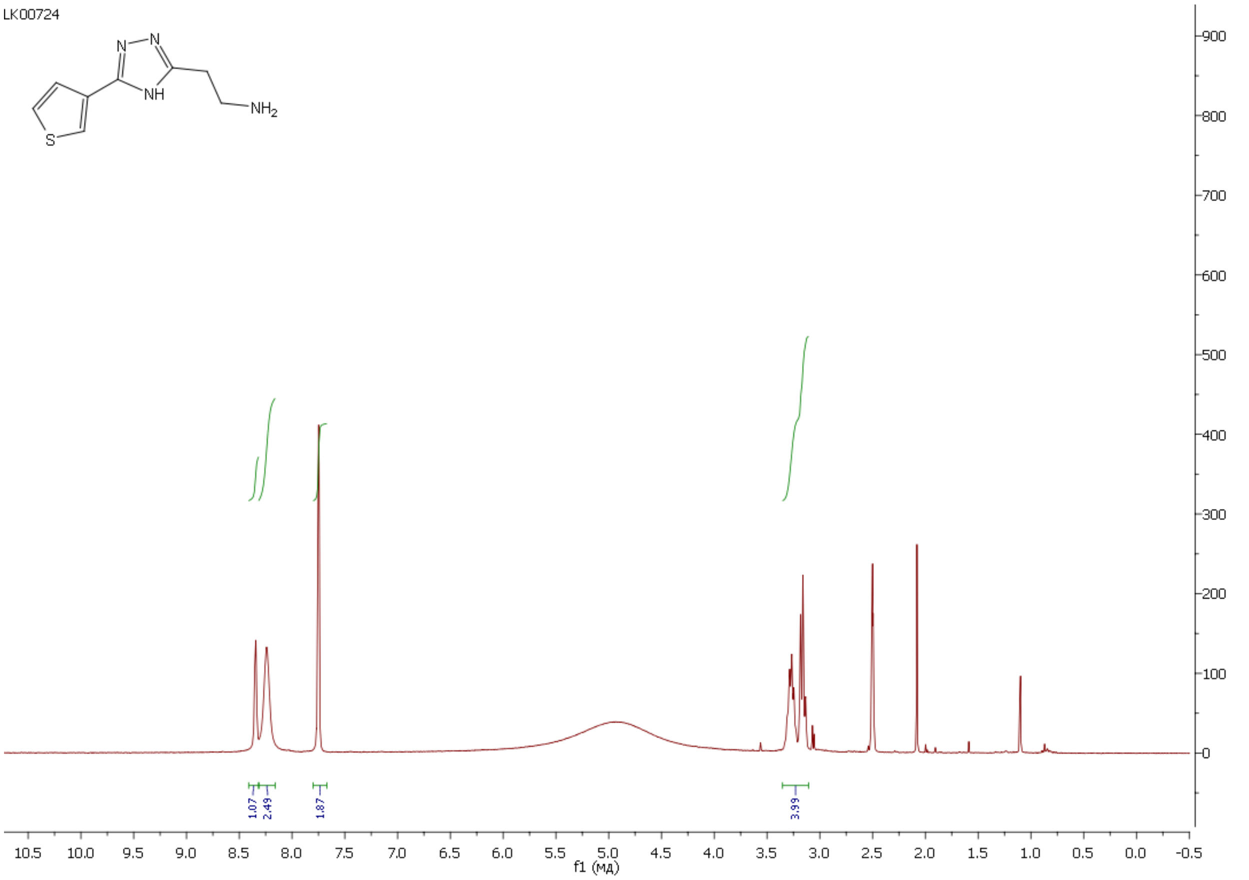

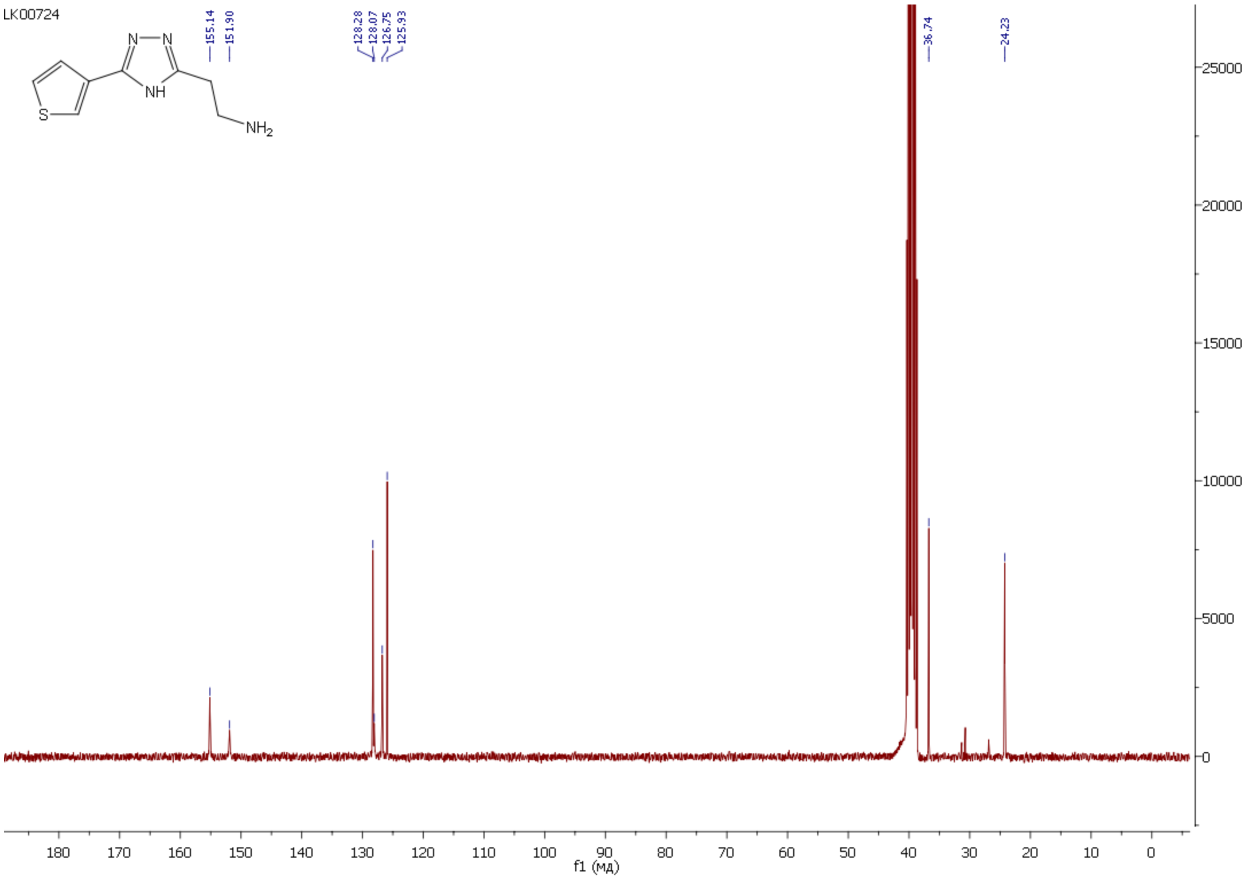

^{1}H and ^{13}C NMR spectra of compound **19**

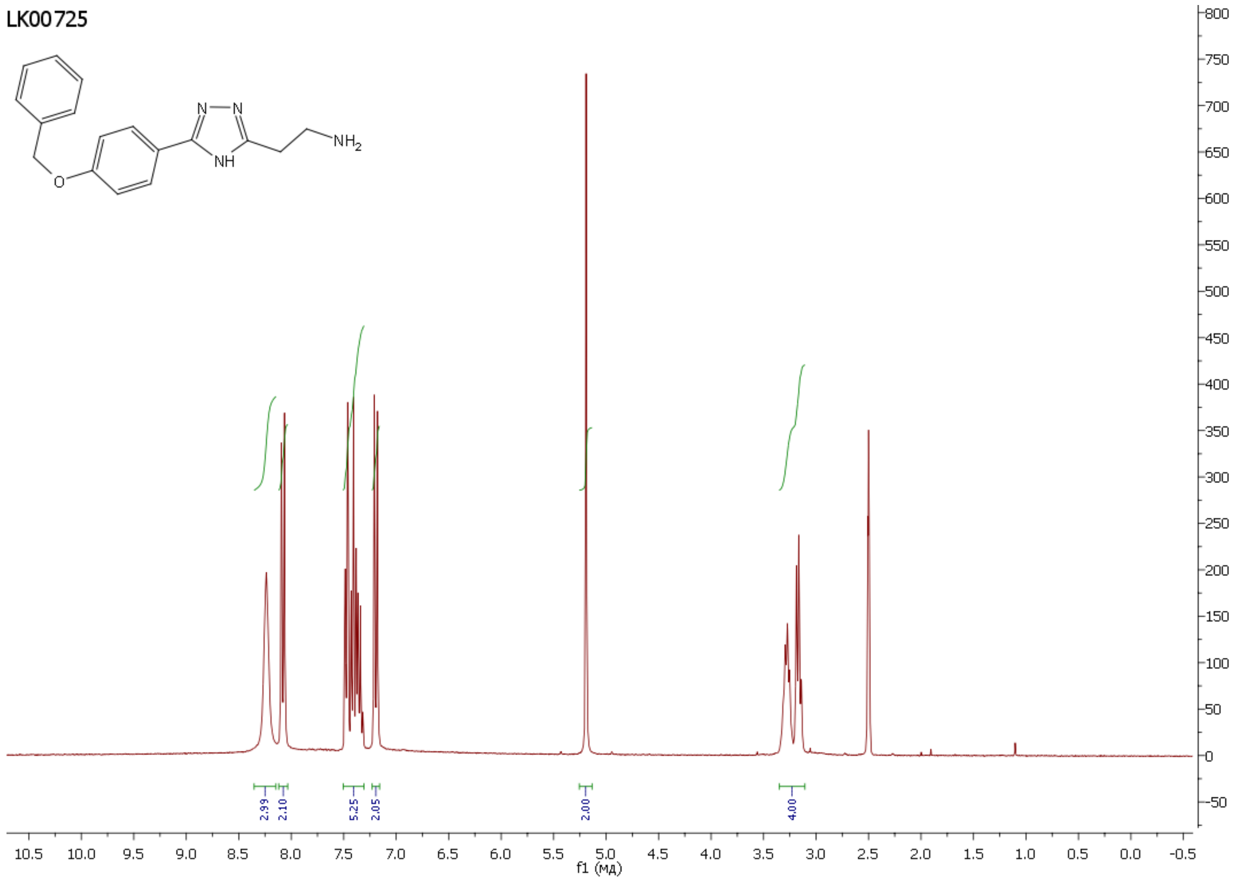

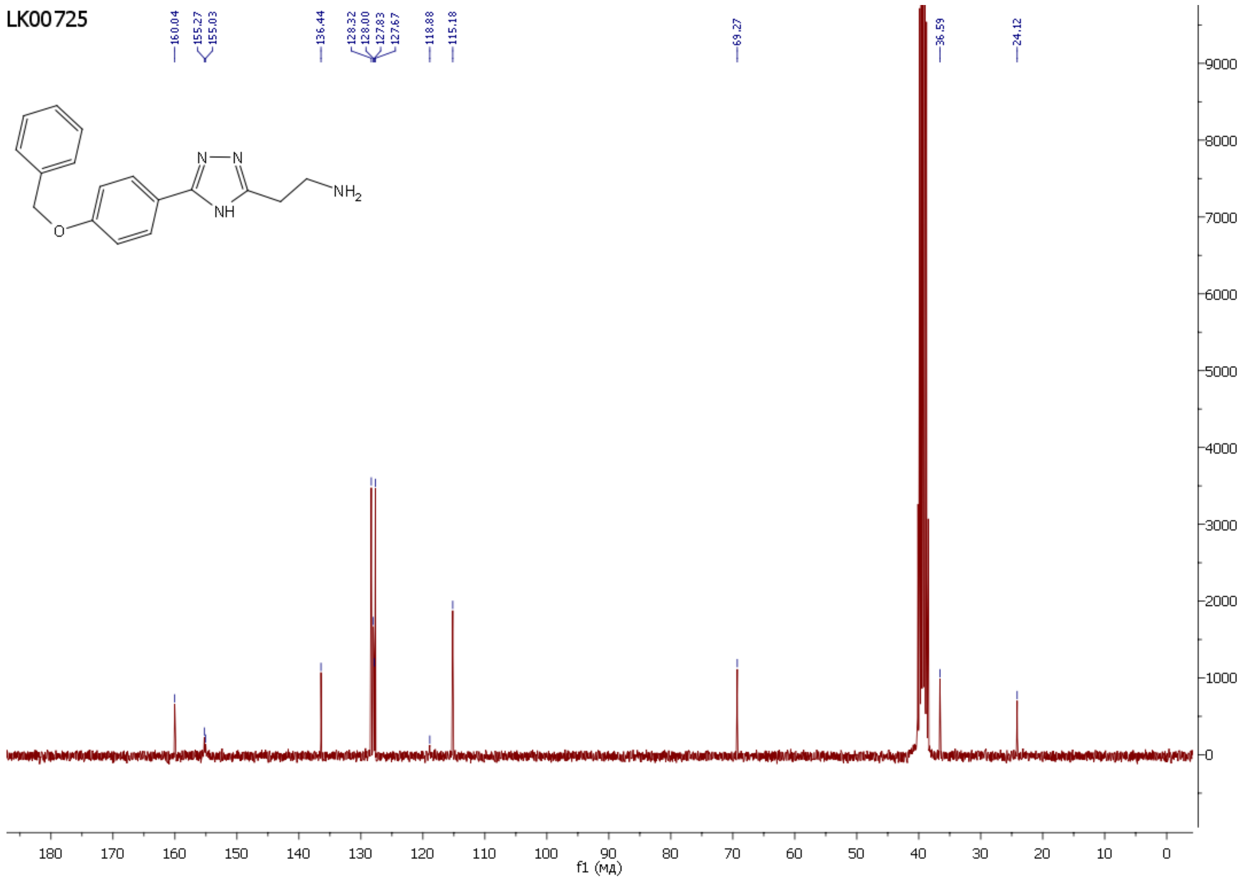

^{1}H and ^{13}C NMR spectra of compound **20**

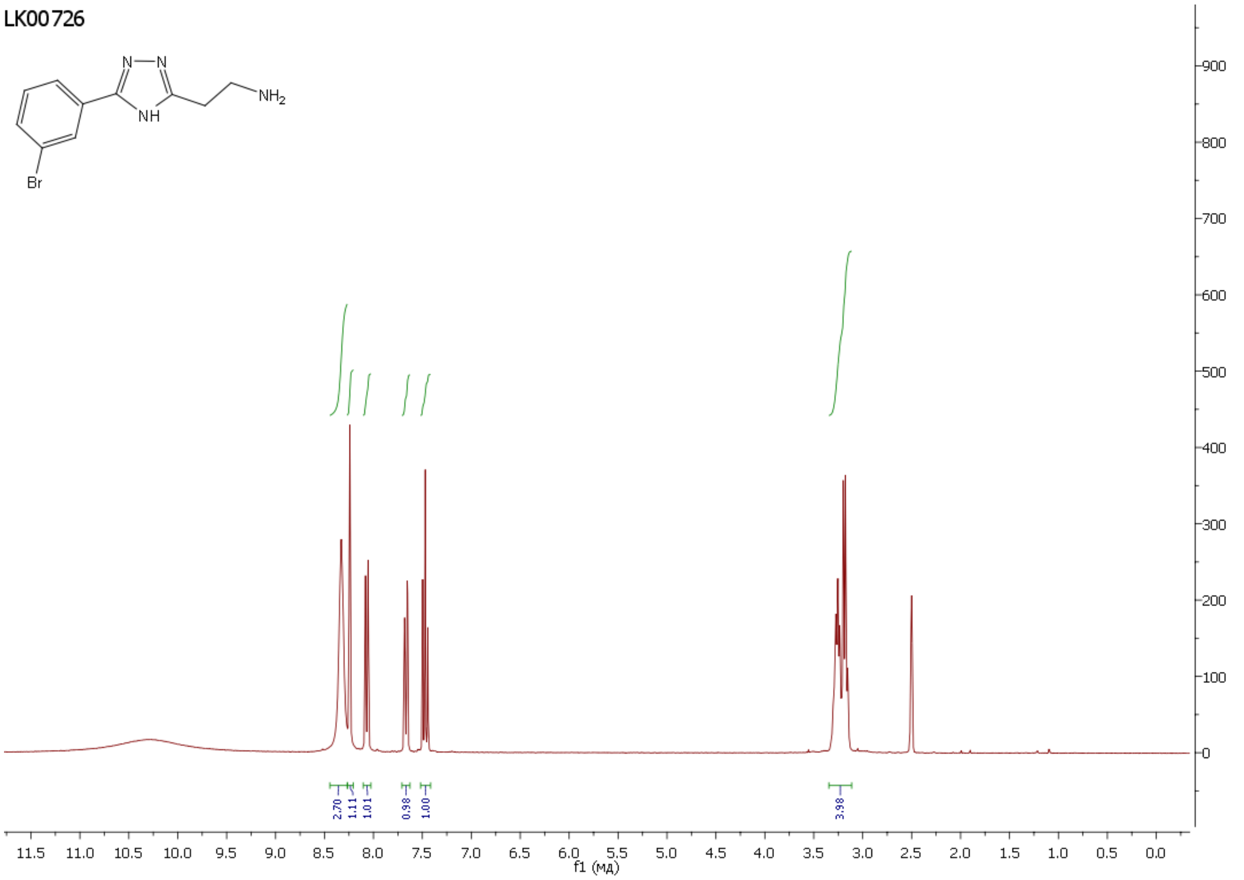

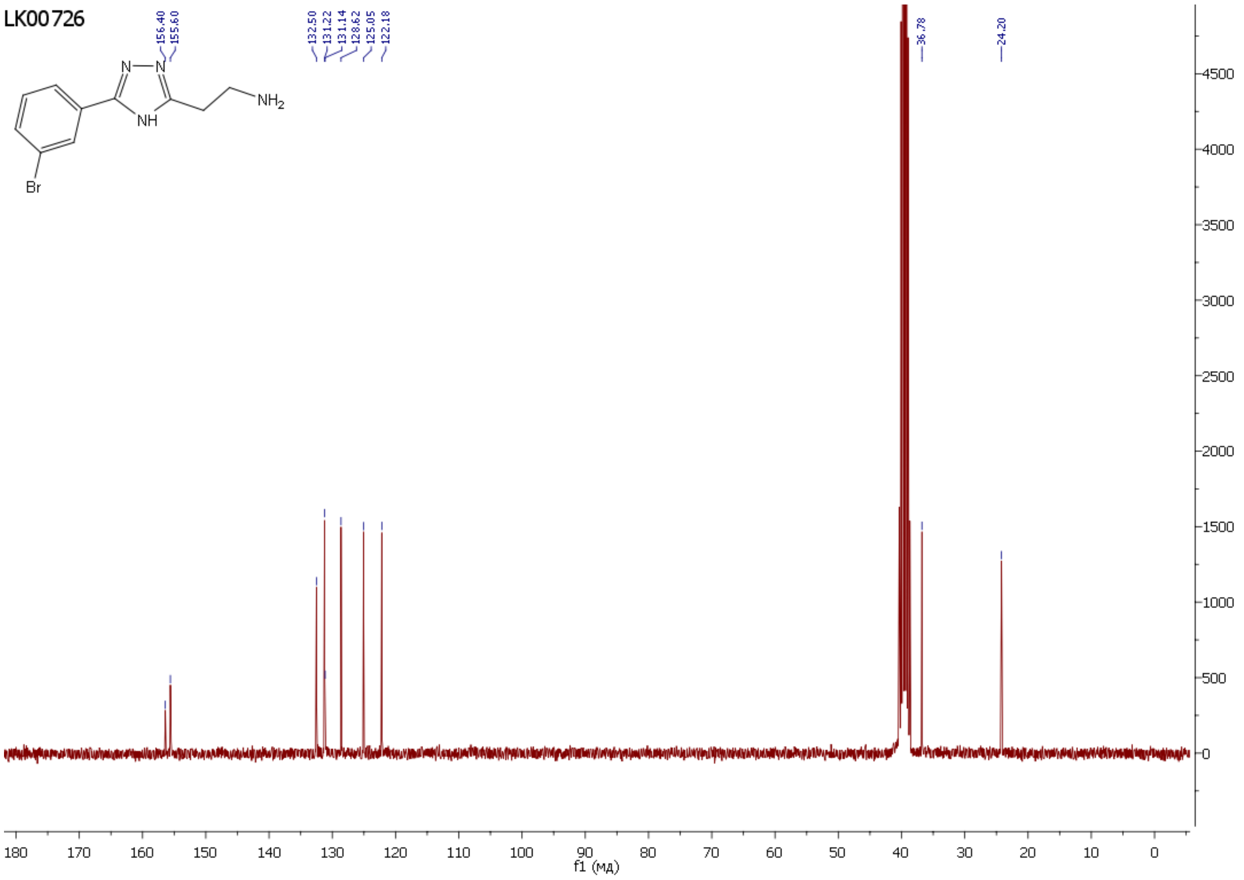

^{1}H and ^{13}C NMR spectra of compound **21**

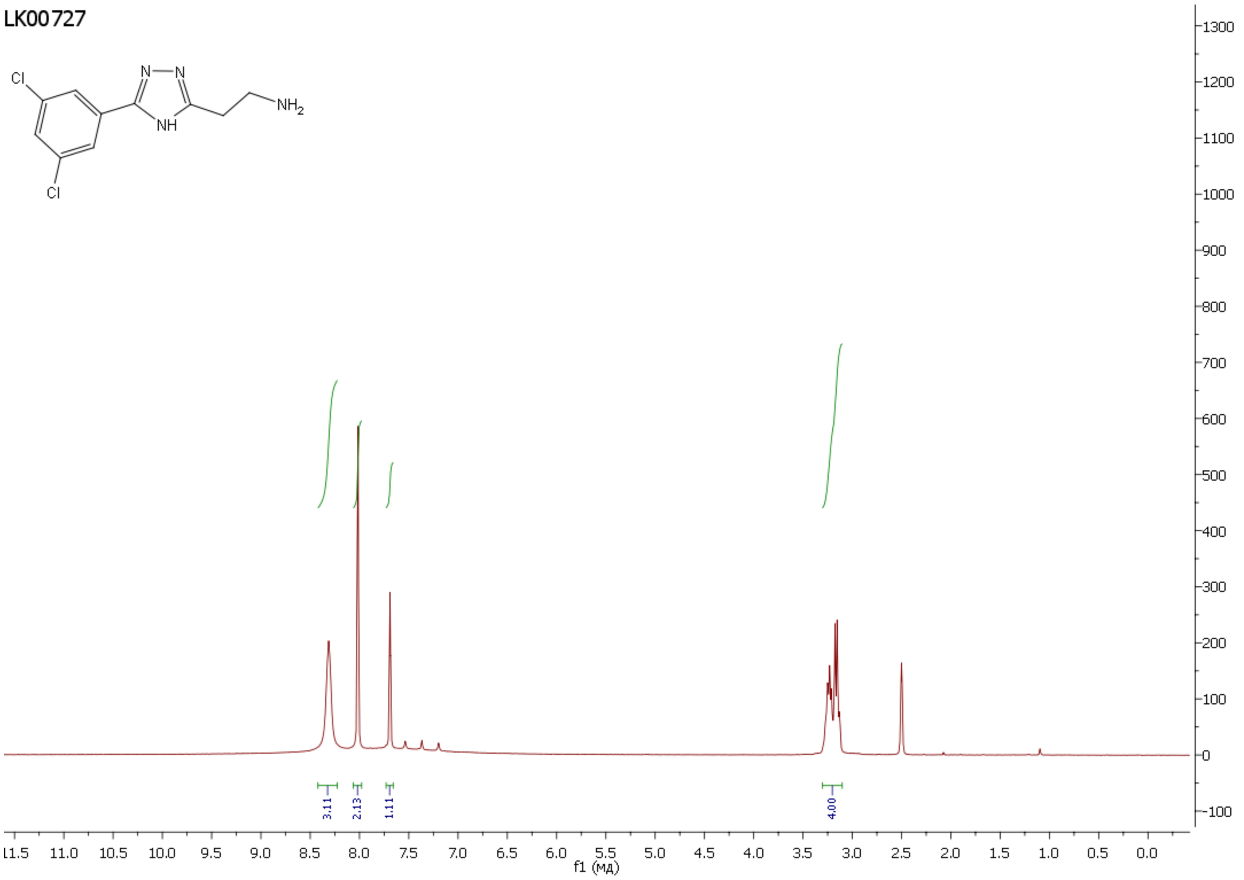

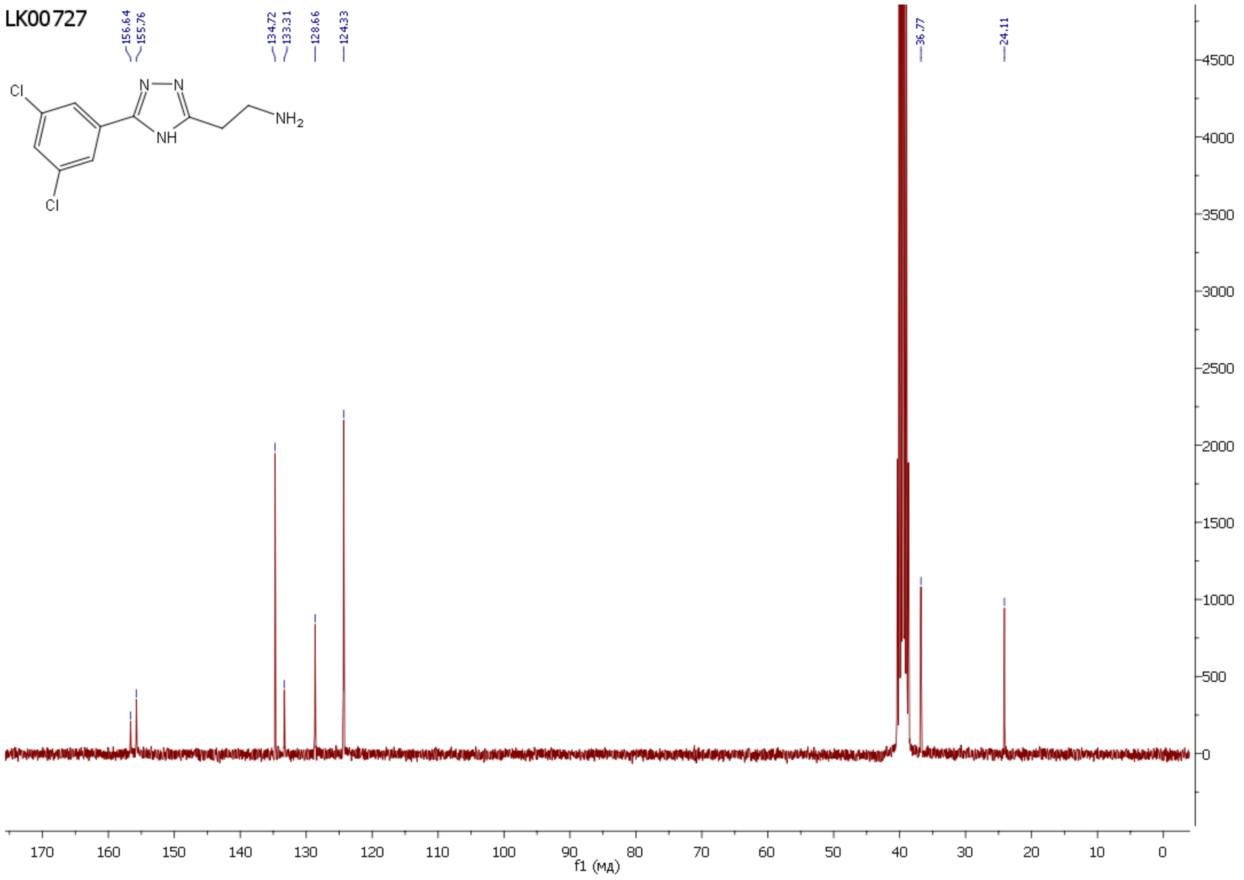

^{1}H and ^{13}C NMR spectra of compound **22**

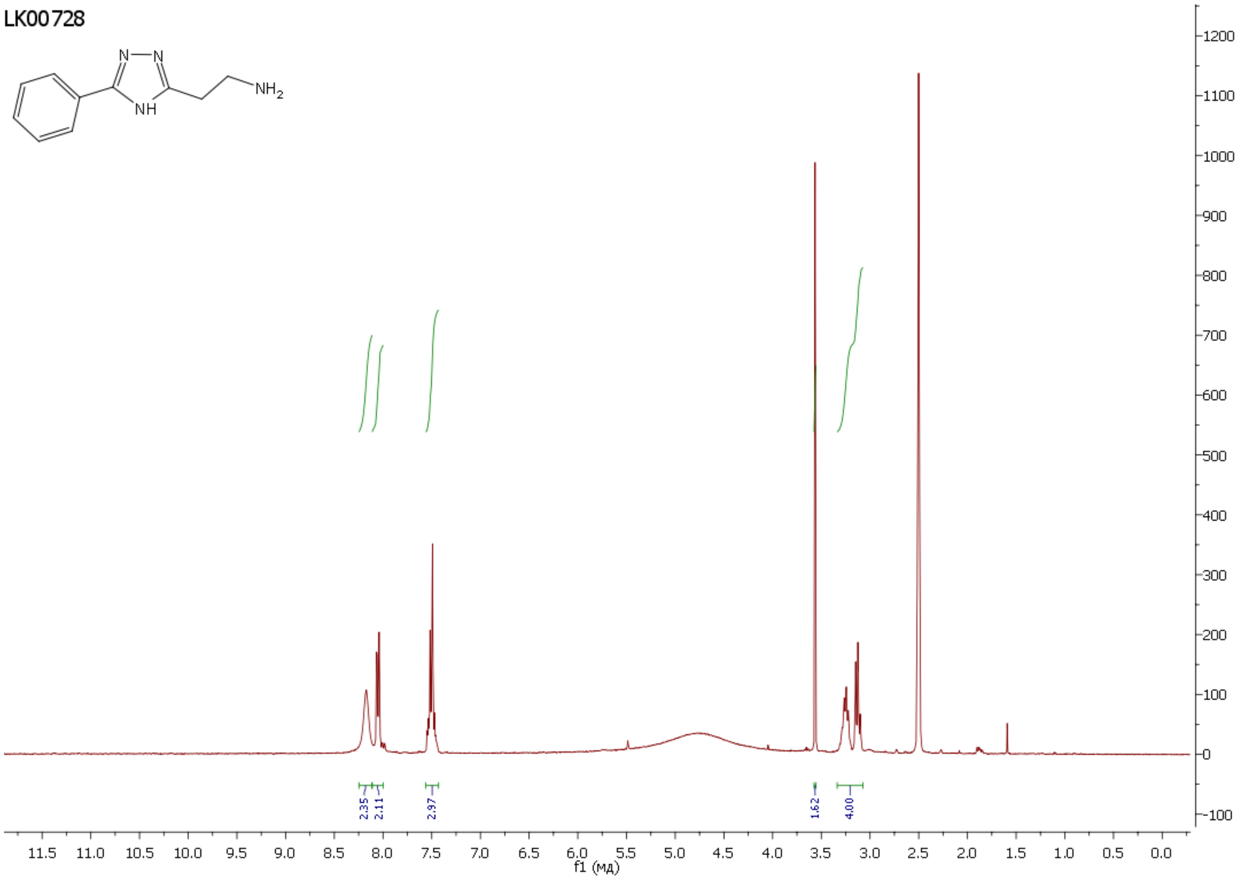

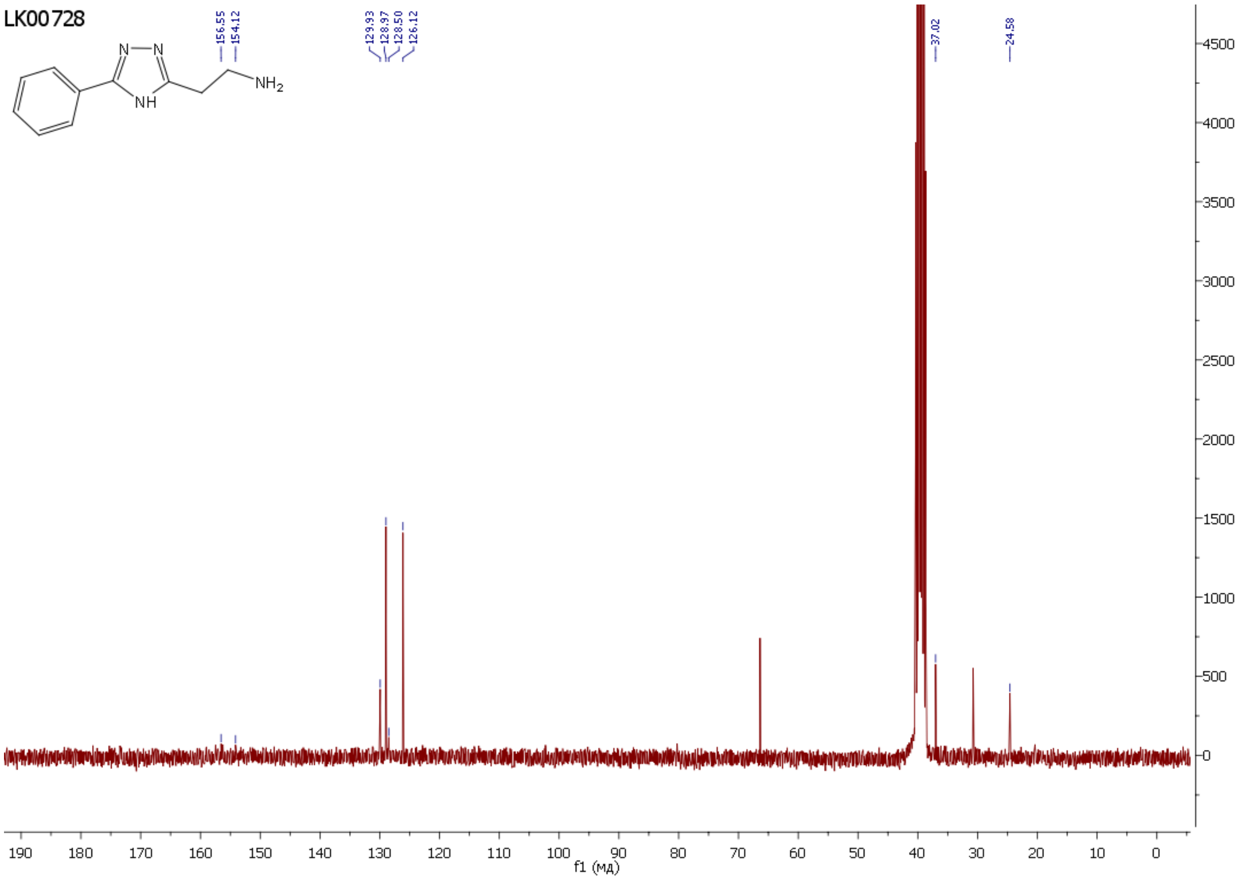

^{1}H and ^{13}C NMR spectra of compound **23**

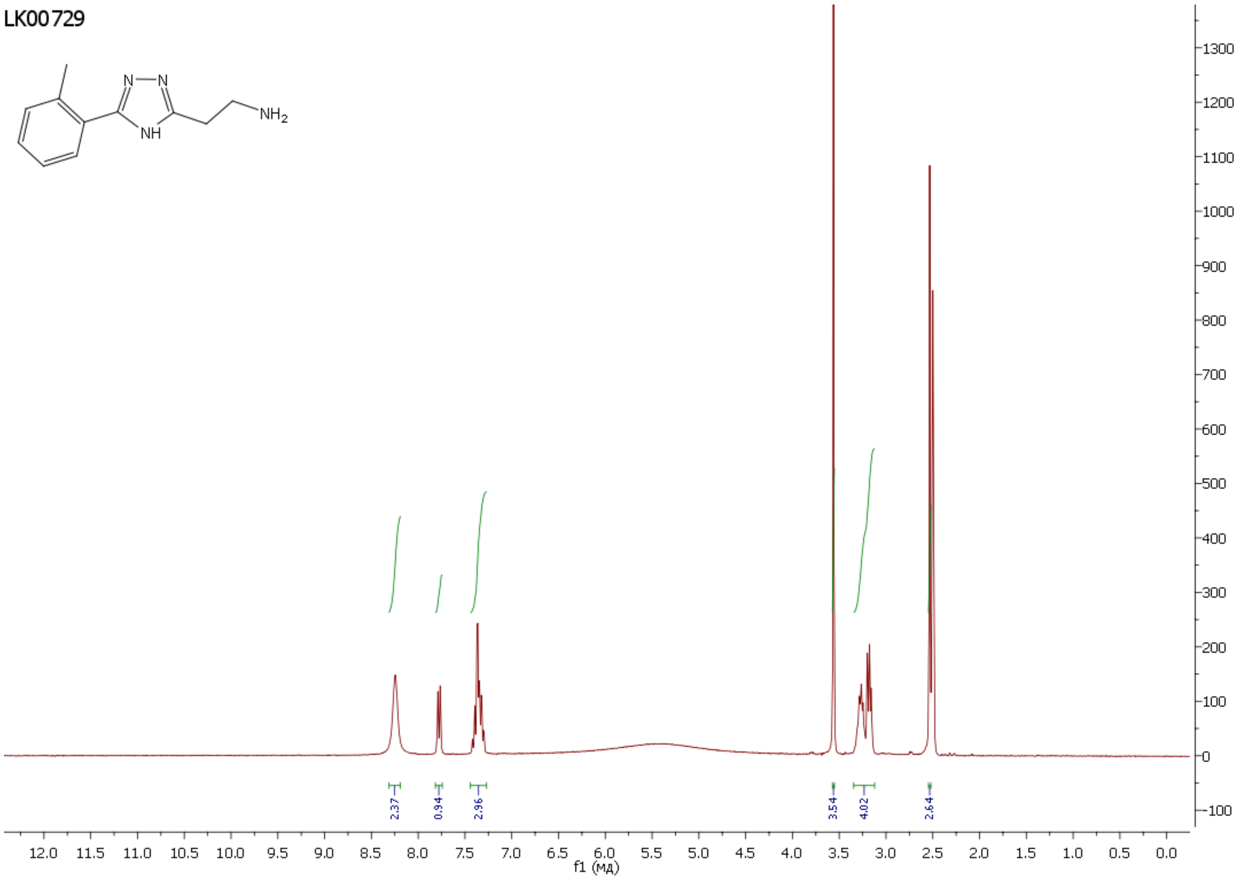

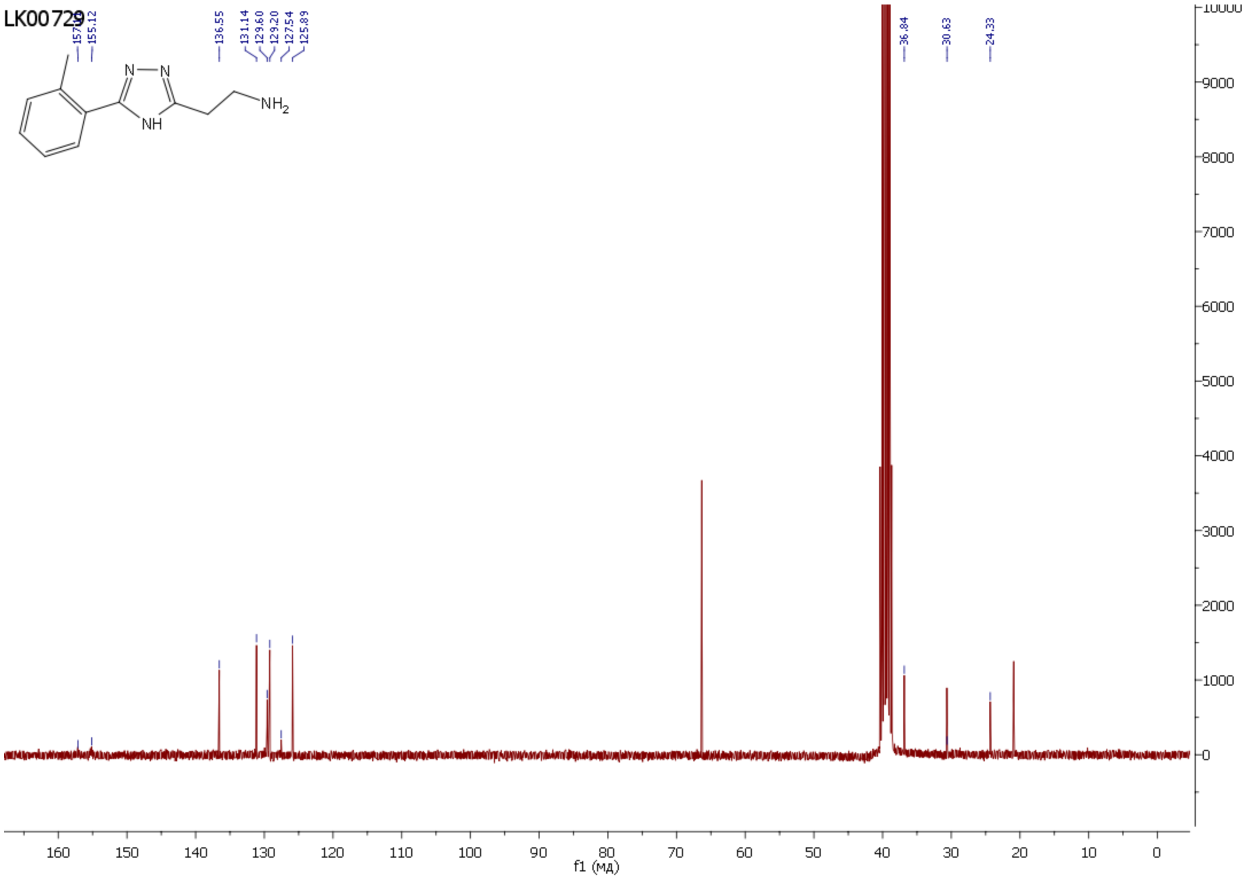

^{1}H and ^{13}C NMR spectra of compound **24**

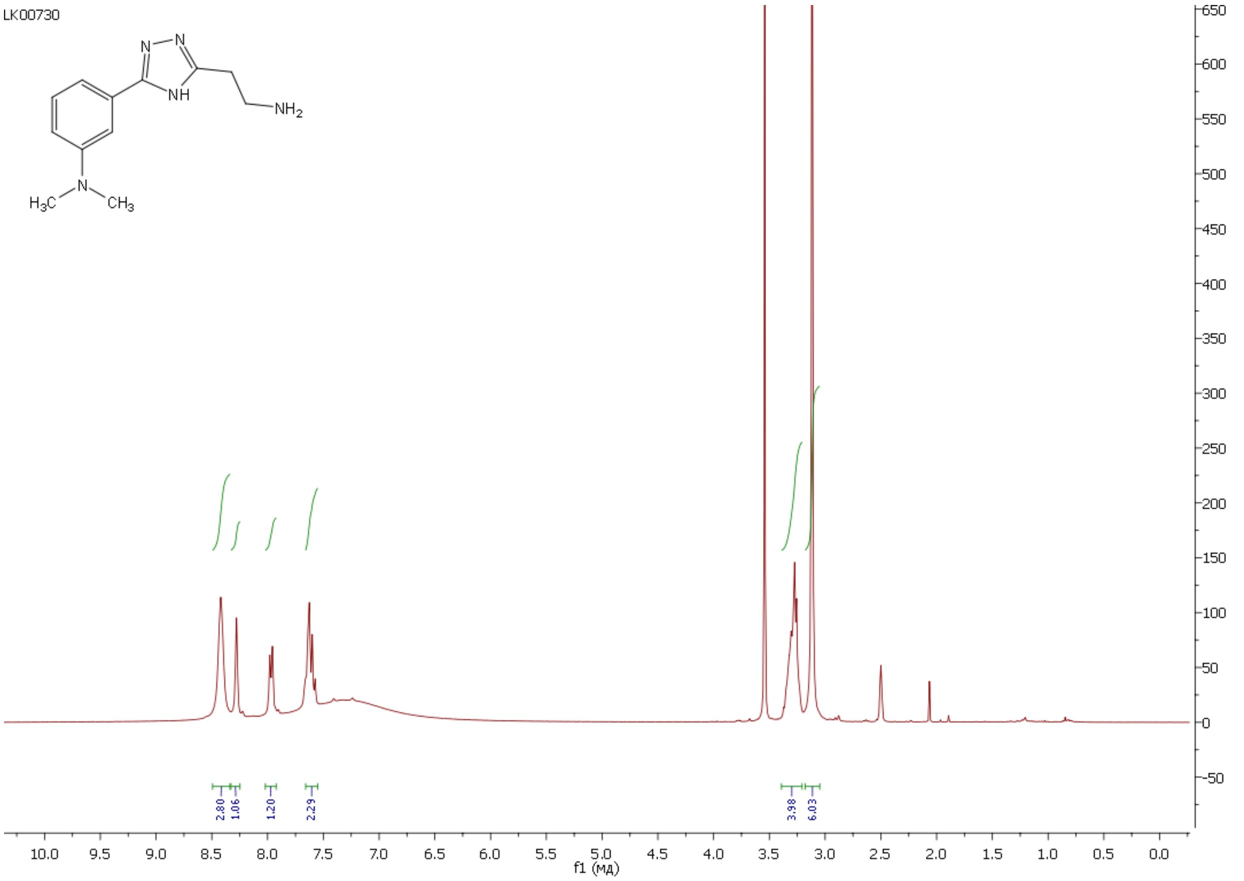

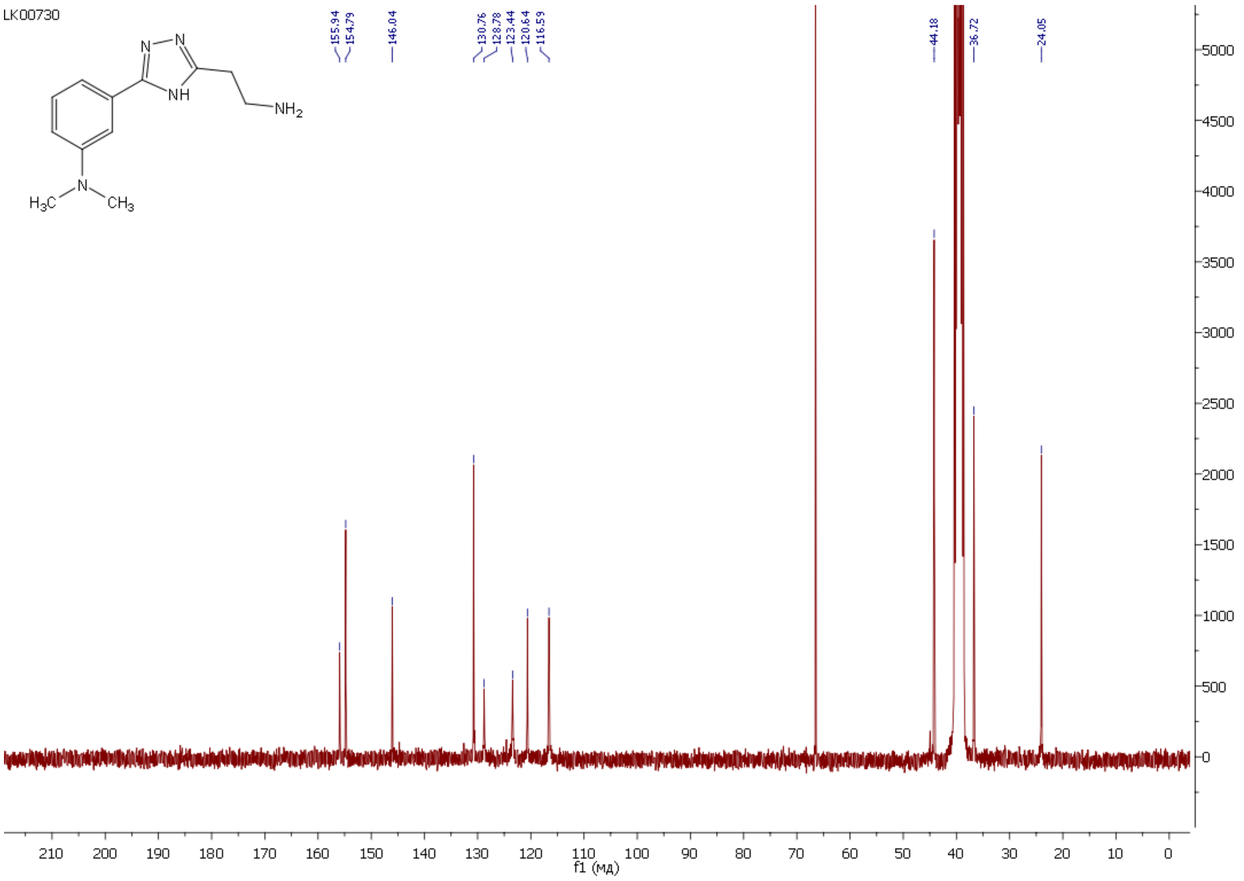

^{1}H and ^{13}C NMR spectra of compound **25**

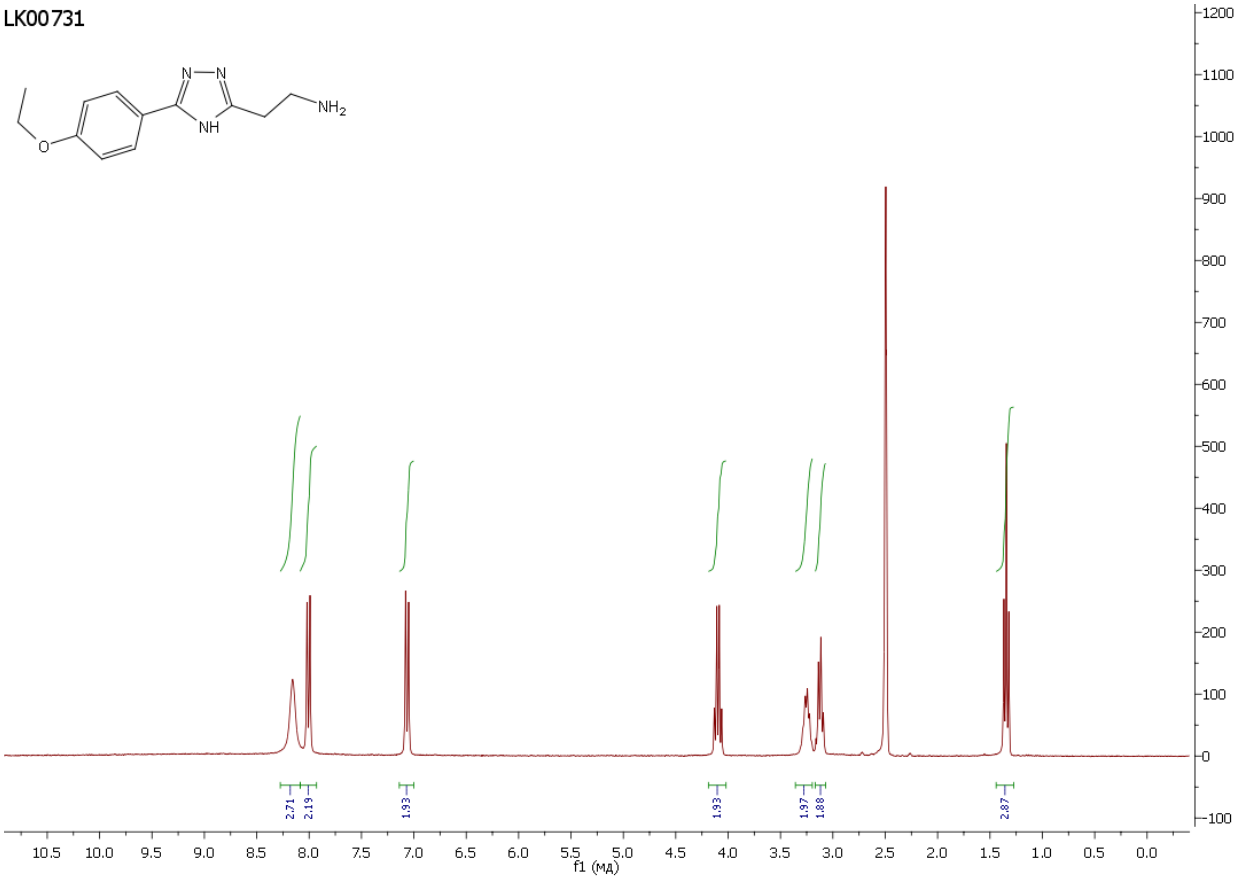

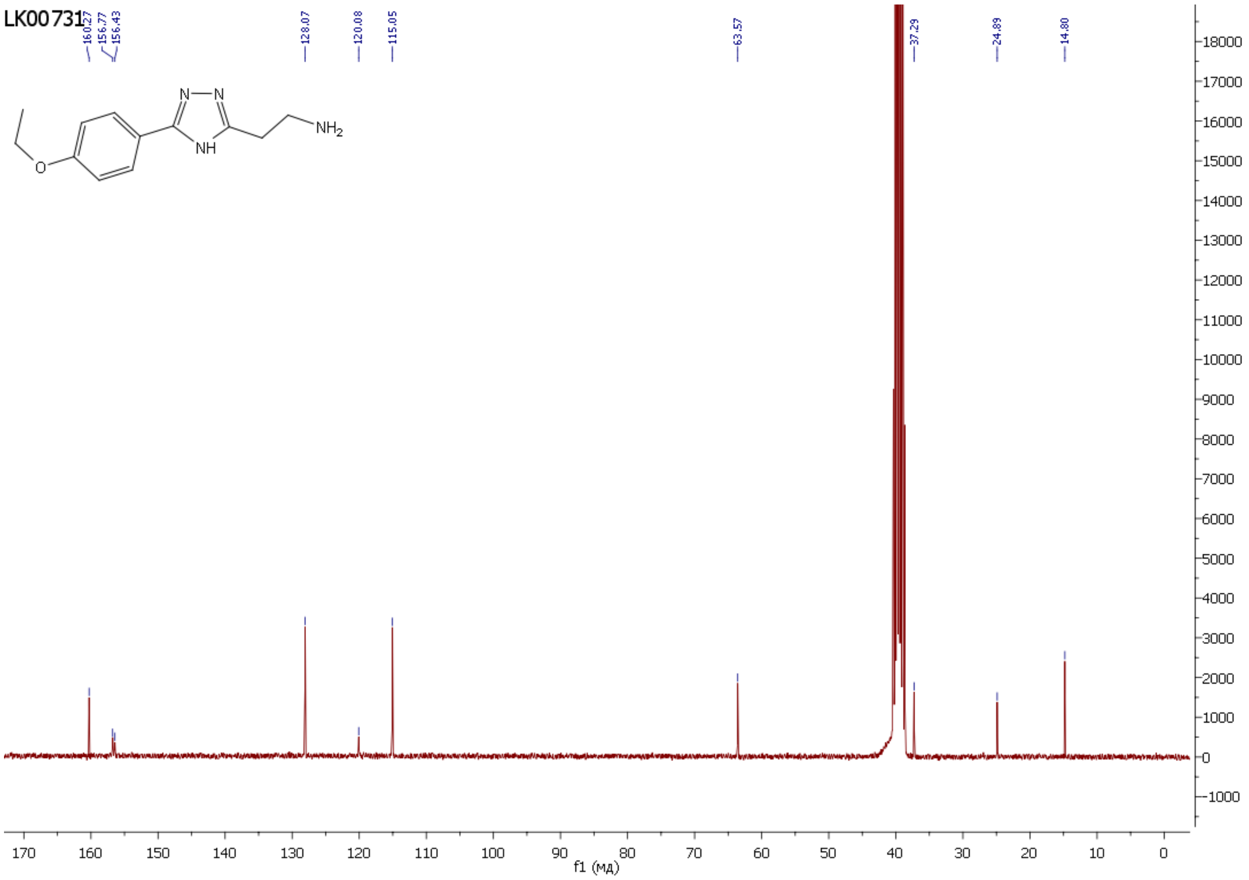

^{1}H and ^{13}C NMR spectra of compound **26**

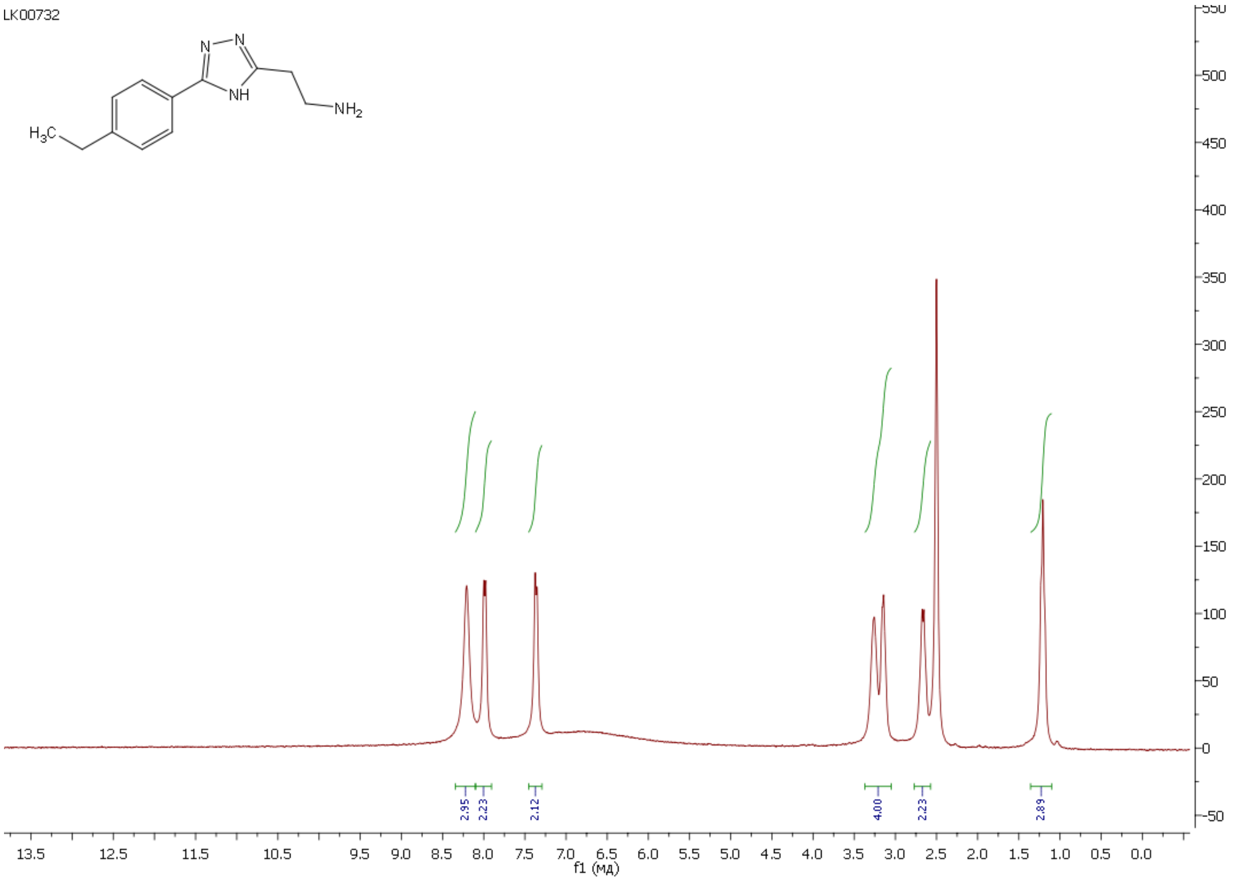

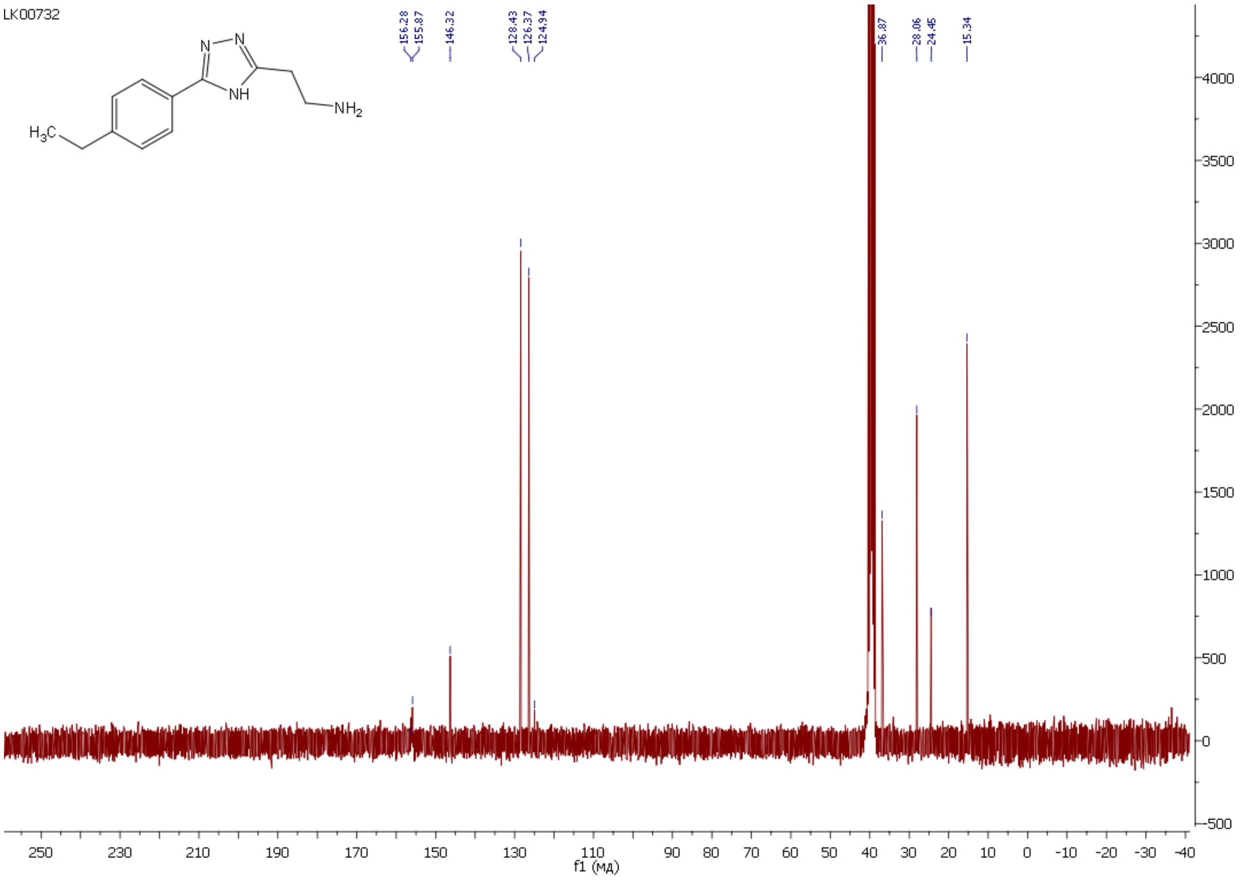

^{1}H and ^{13}C NMR spectra of compound **27**

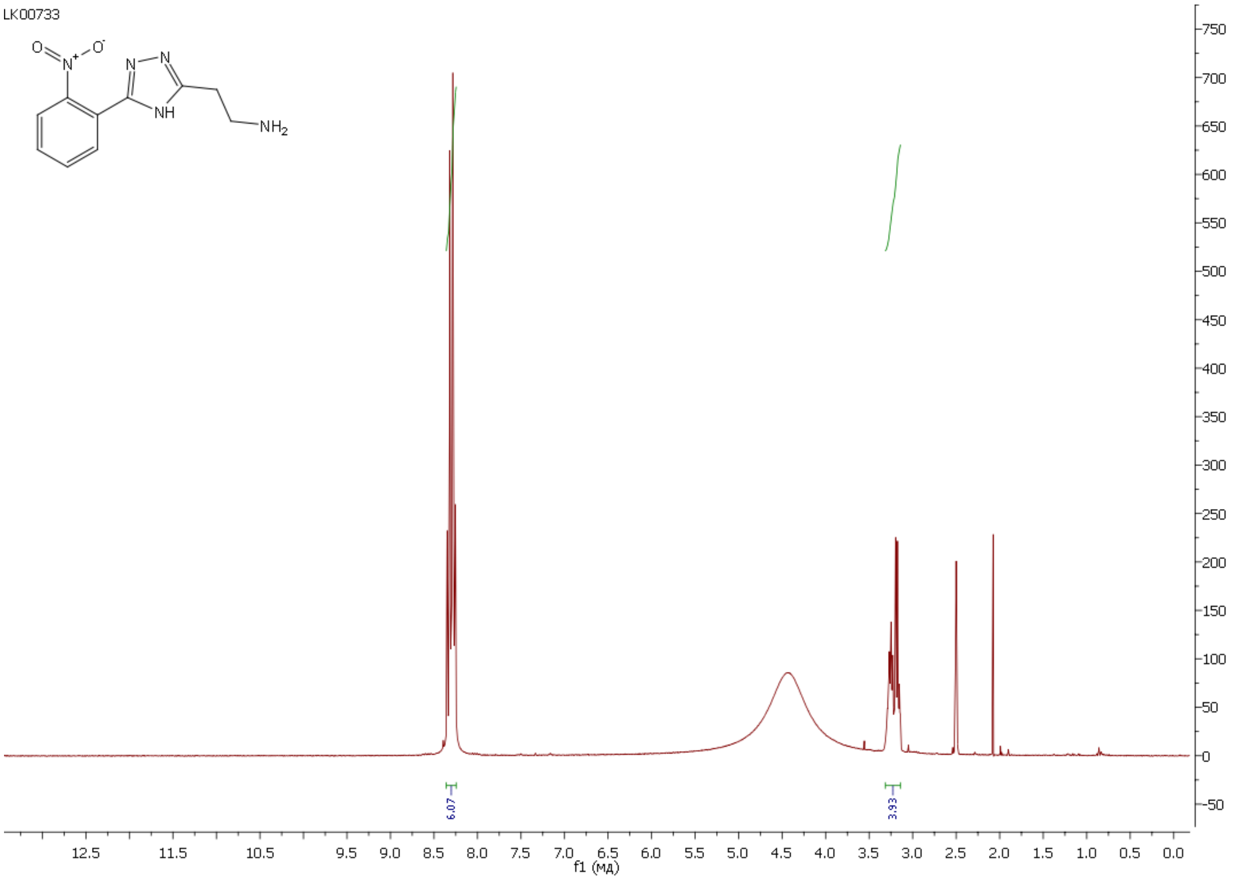

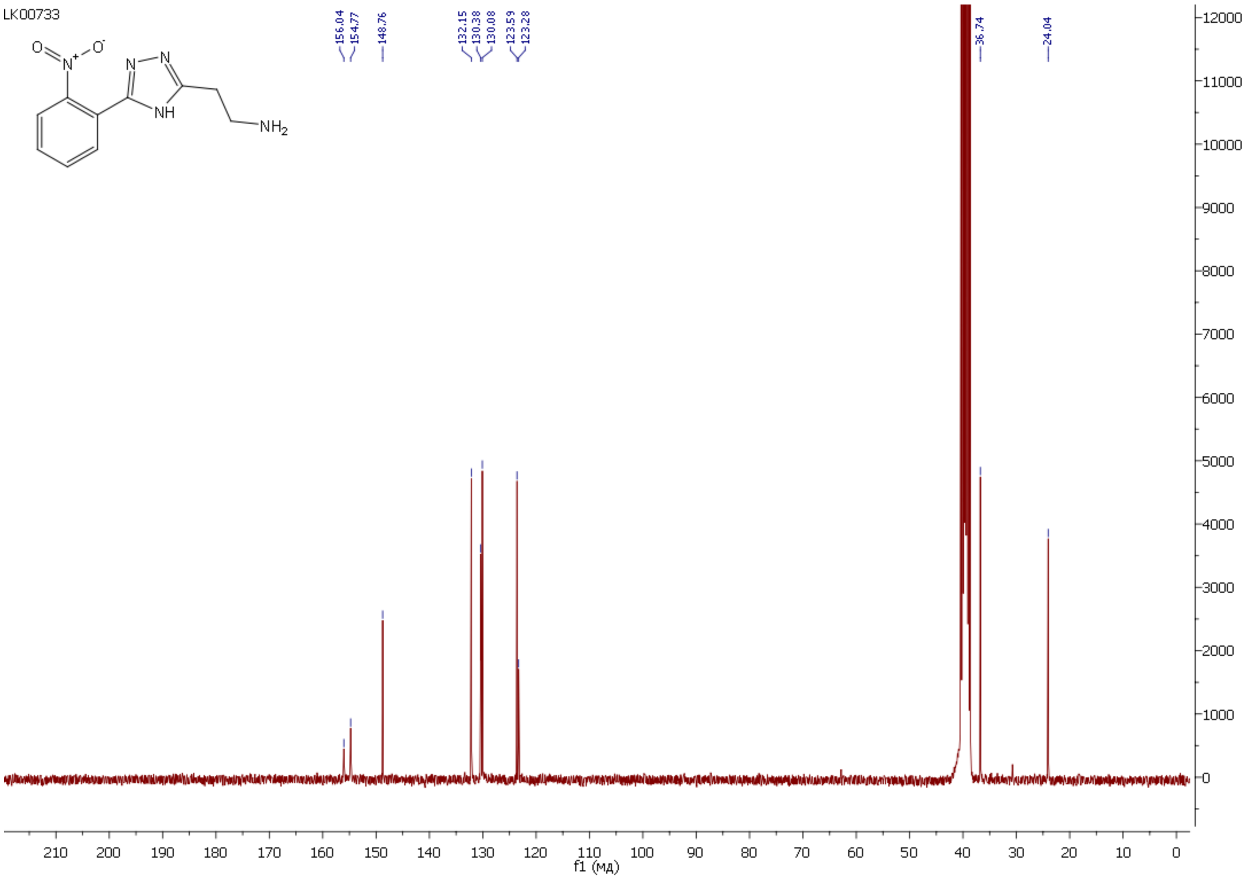

^{1}H and ^{13}C NMR spectra of compound **28**

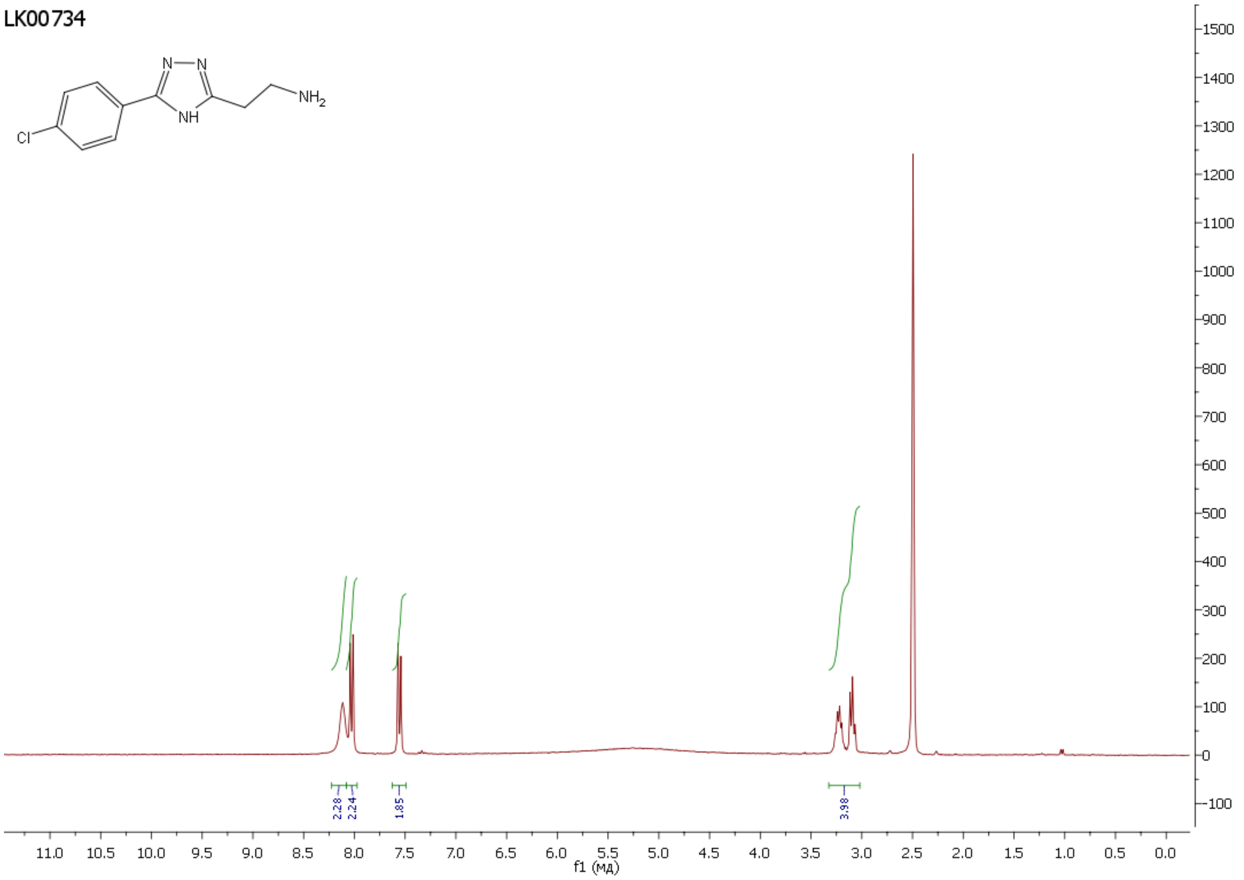

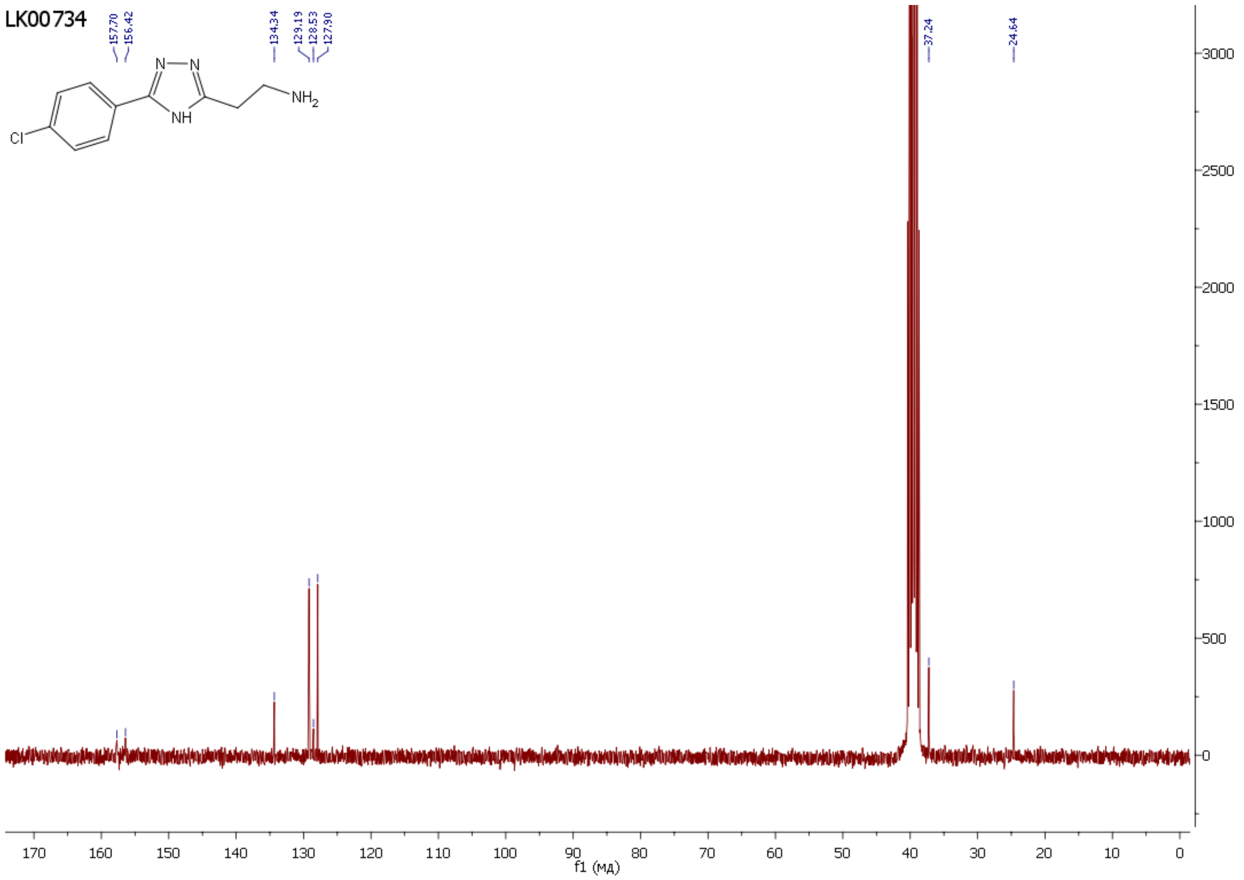

^{1}H and ^{13}C NMR spectra of compound **29**

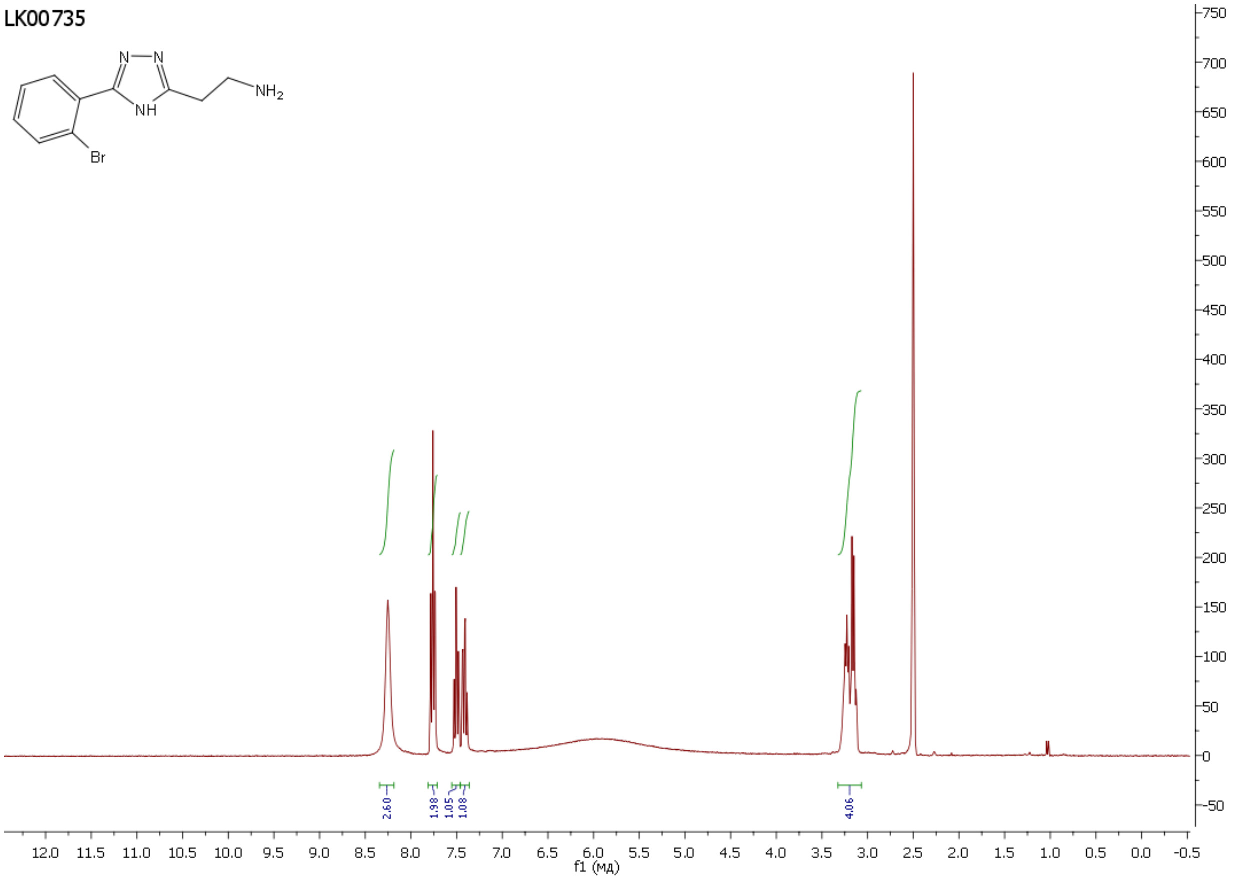

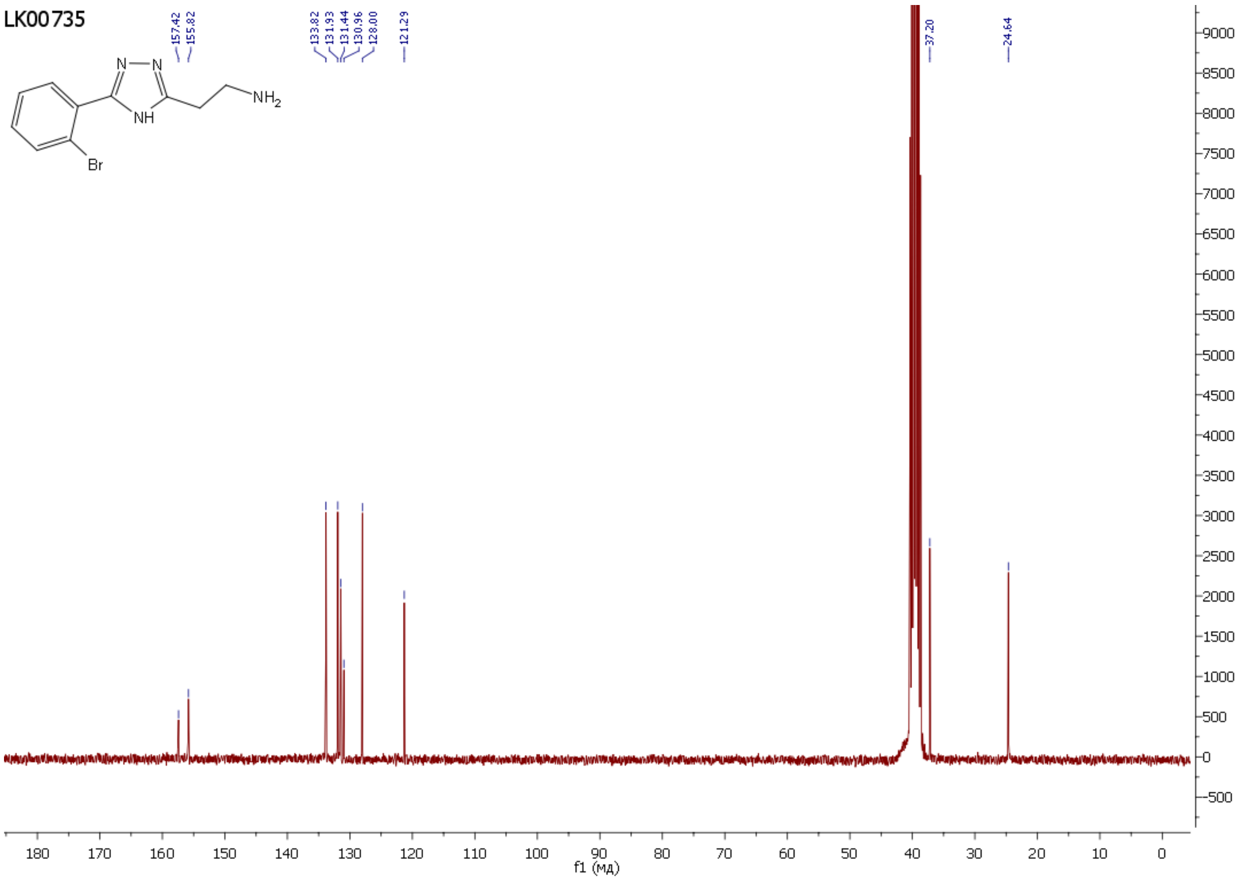

^{1}H and ^{13}C NMR spectra of compound **30**

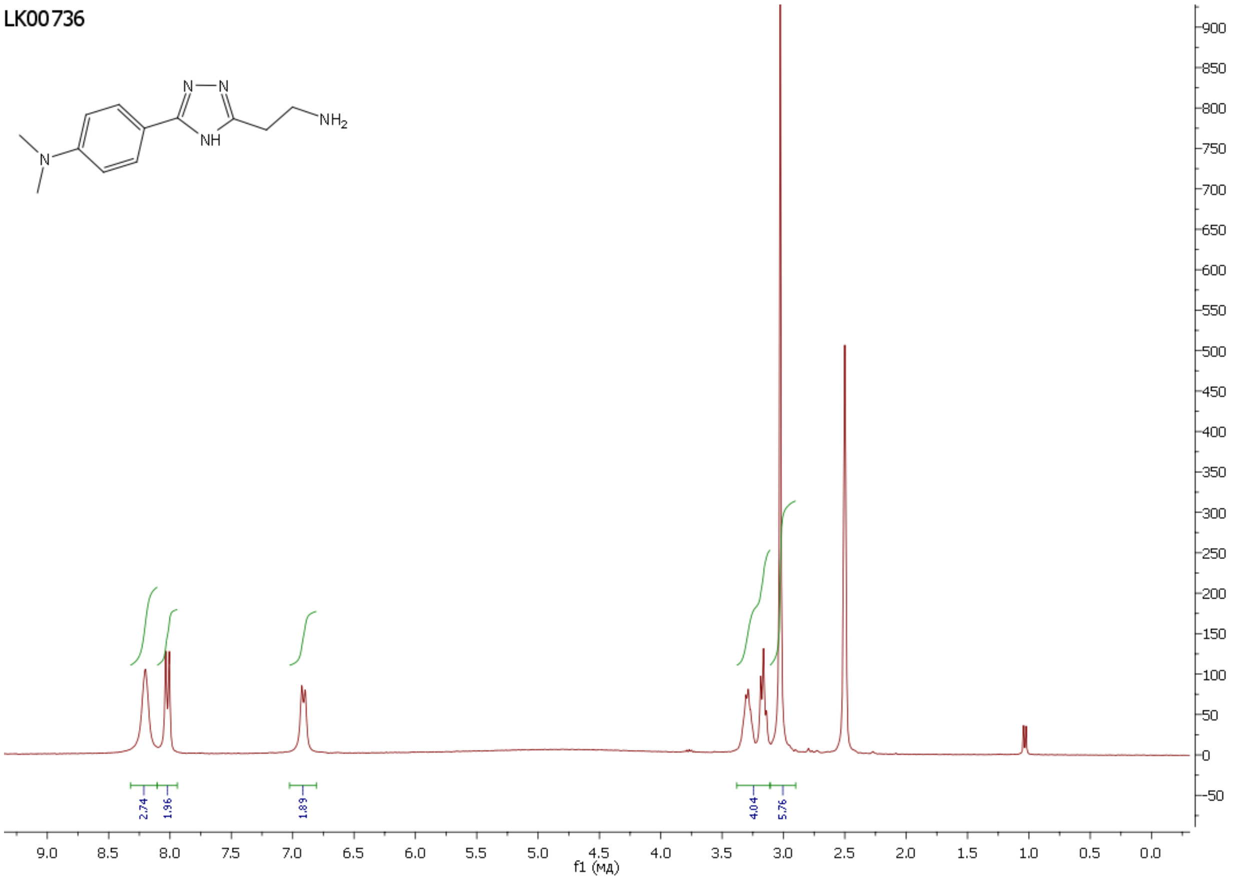

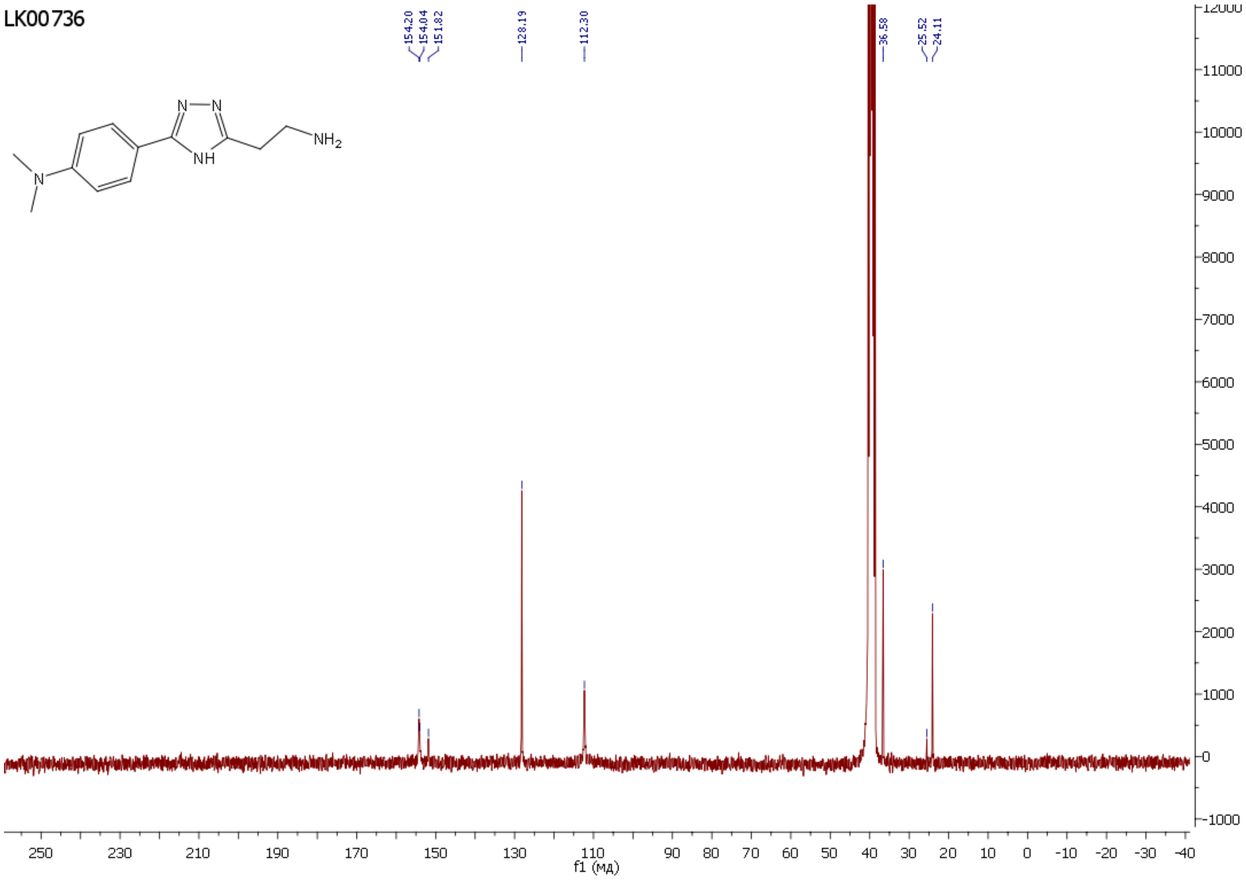

^{1}H and ^{13}C NMR spectra of compound **31**

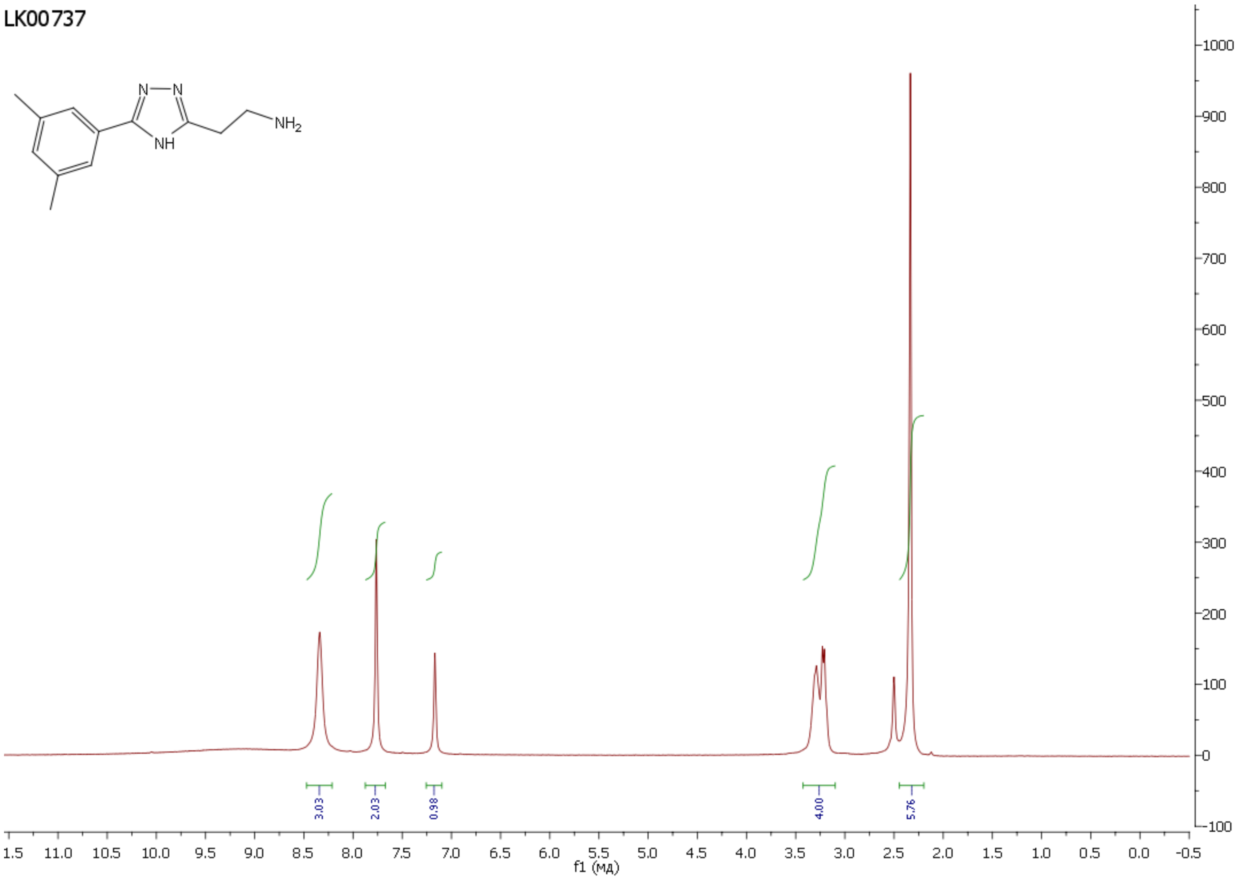

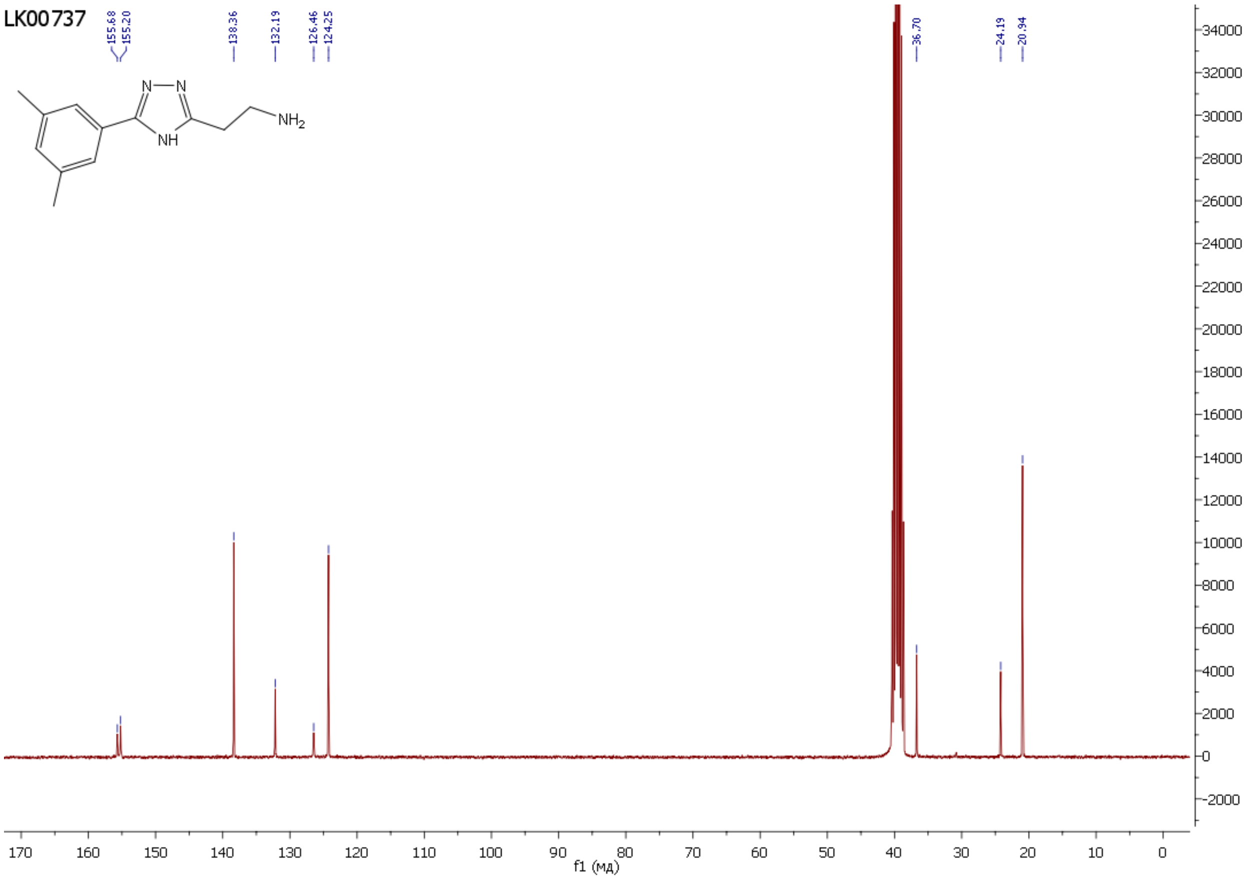

^{1}H and ^{13}C NMR spectra of compound **32**

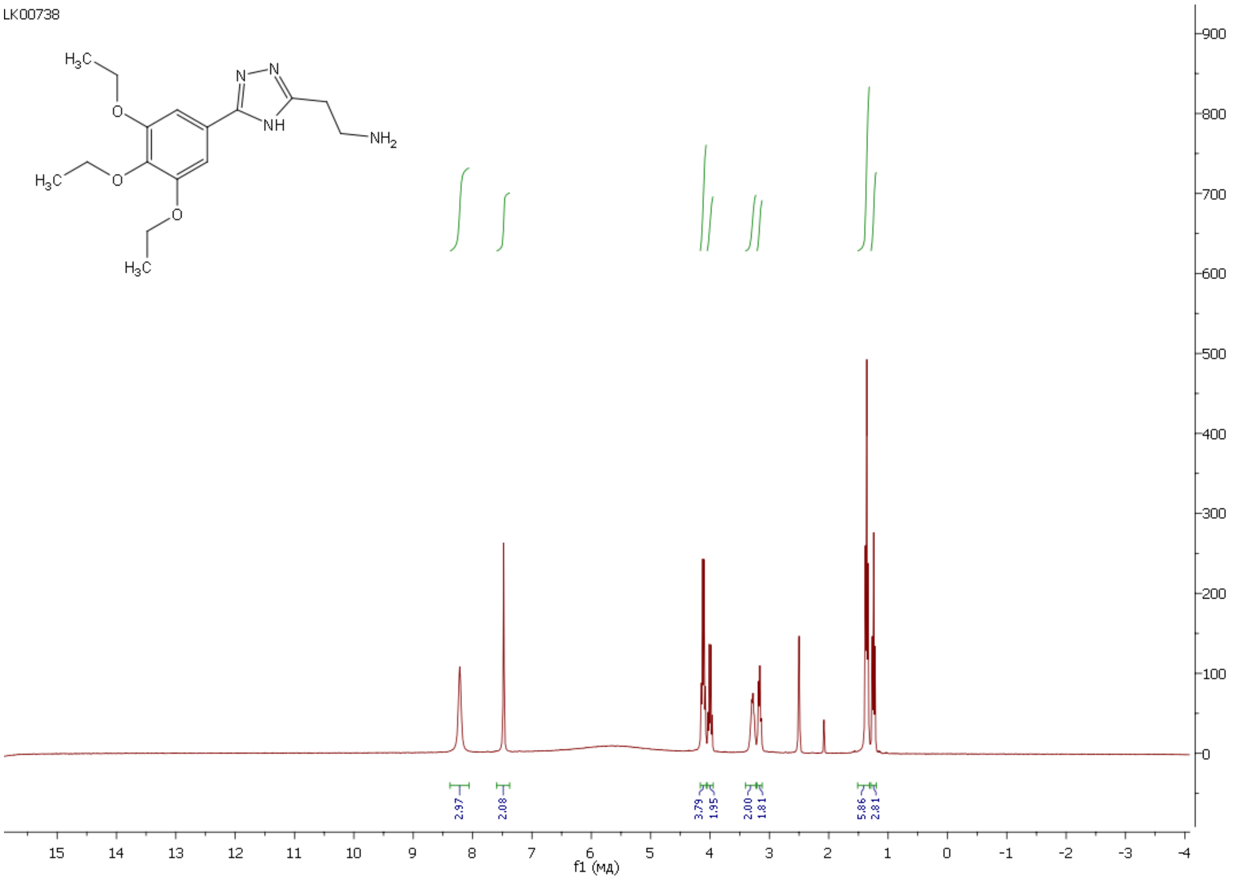

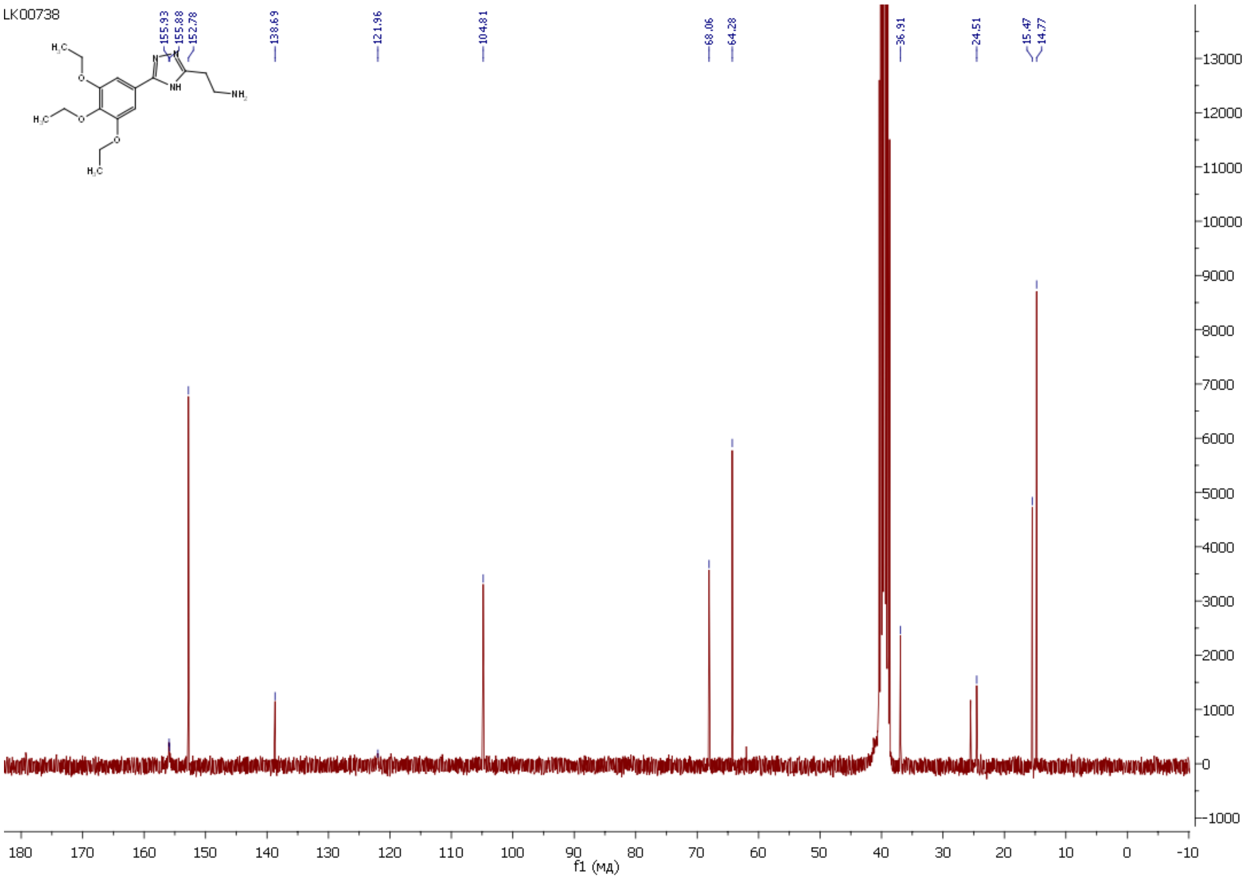

^{1}H and ^{13}C NMR spectra of compound **33**

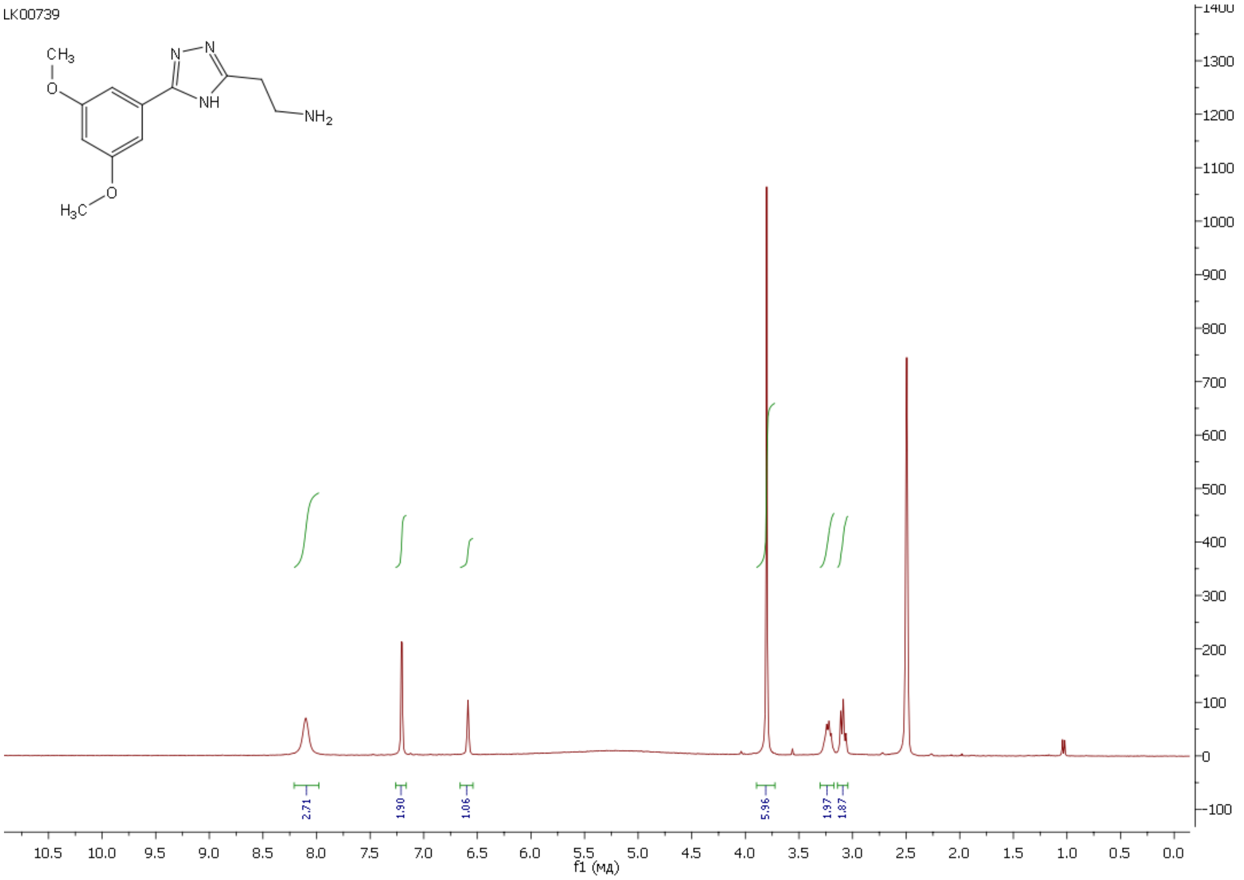

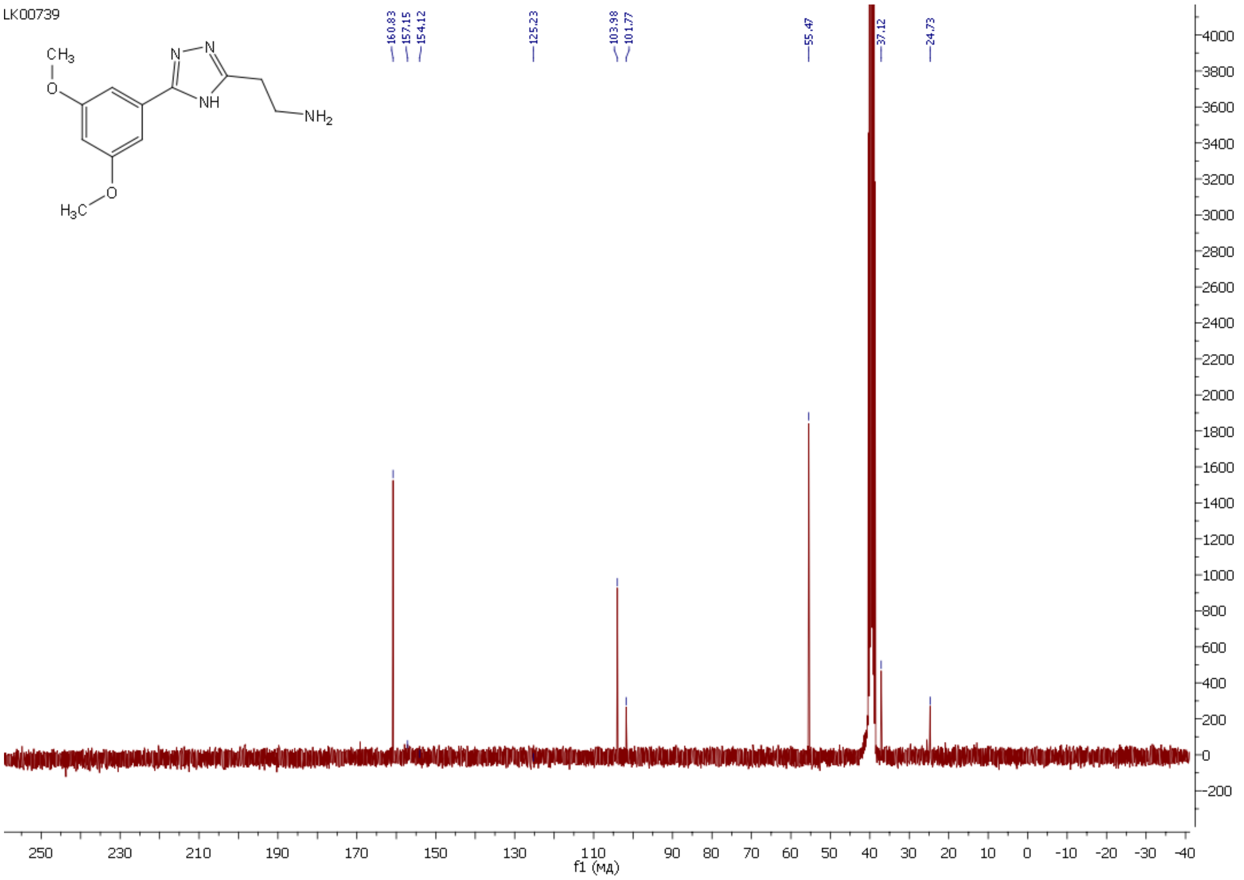

^{1}H and ^{13}C NMR spectra of compound **34**

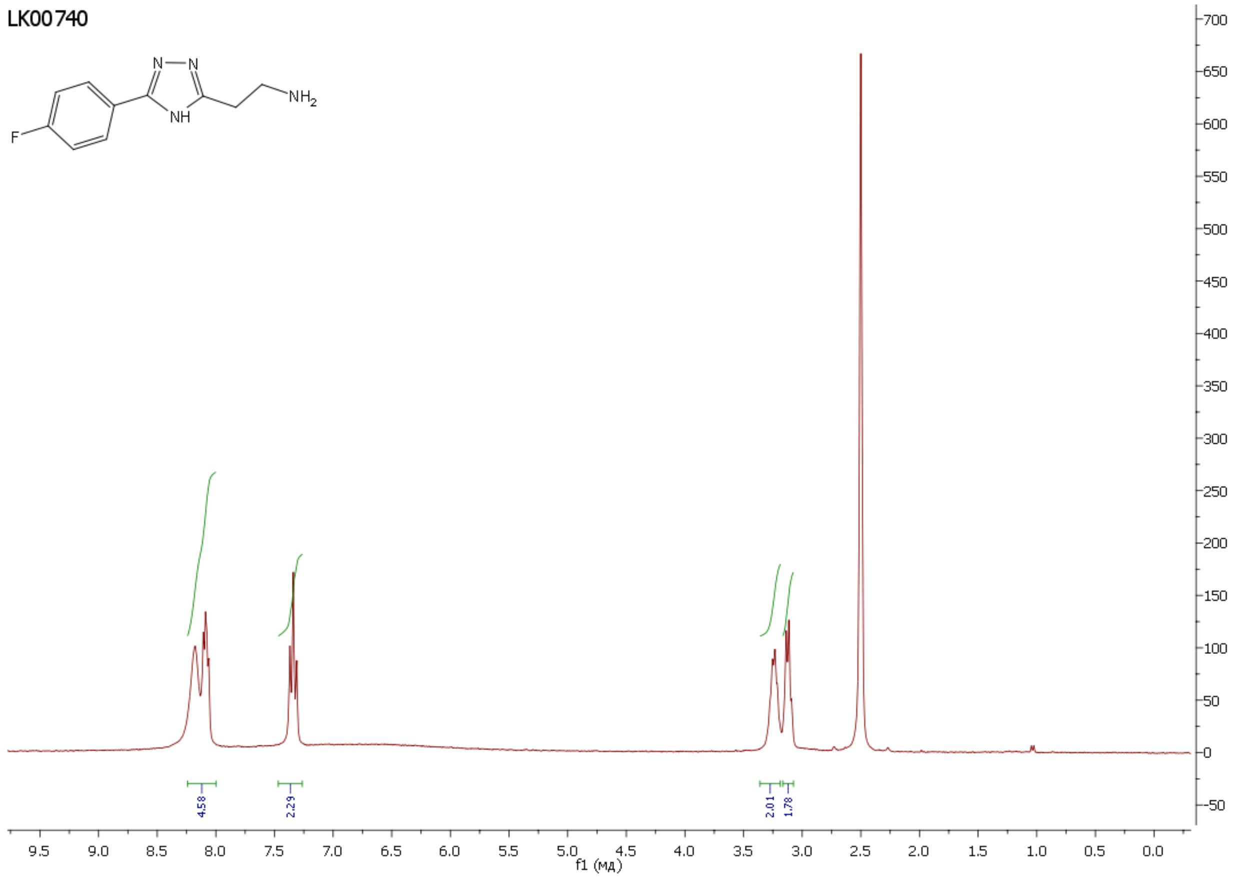

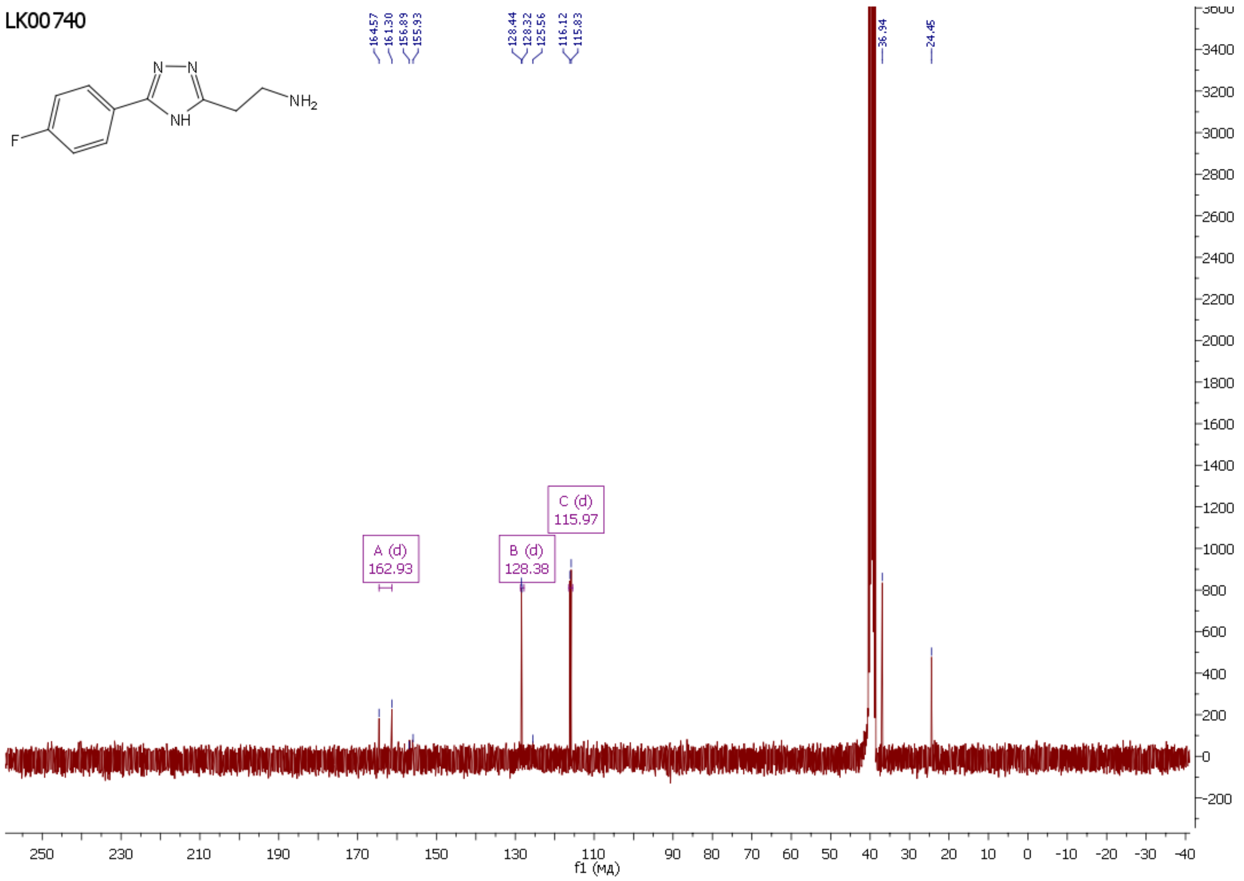

^{1}H and ^{13}C NMR spectra of compound **35**

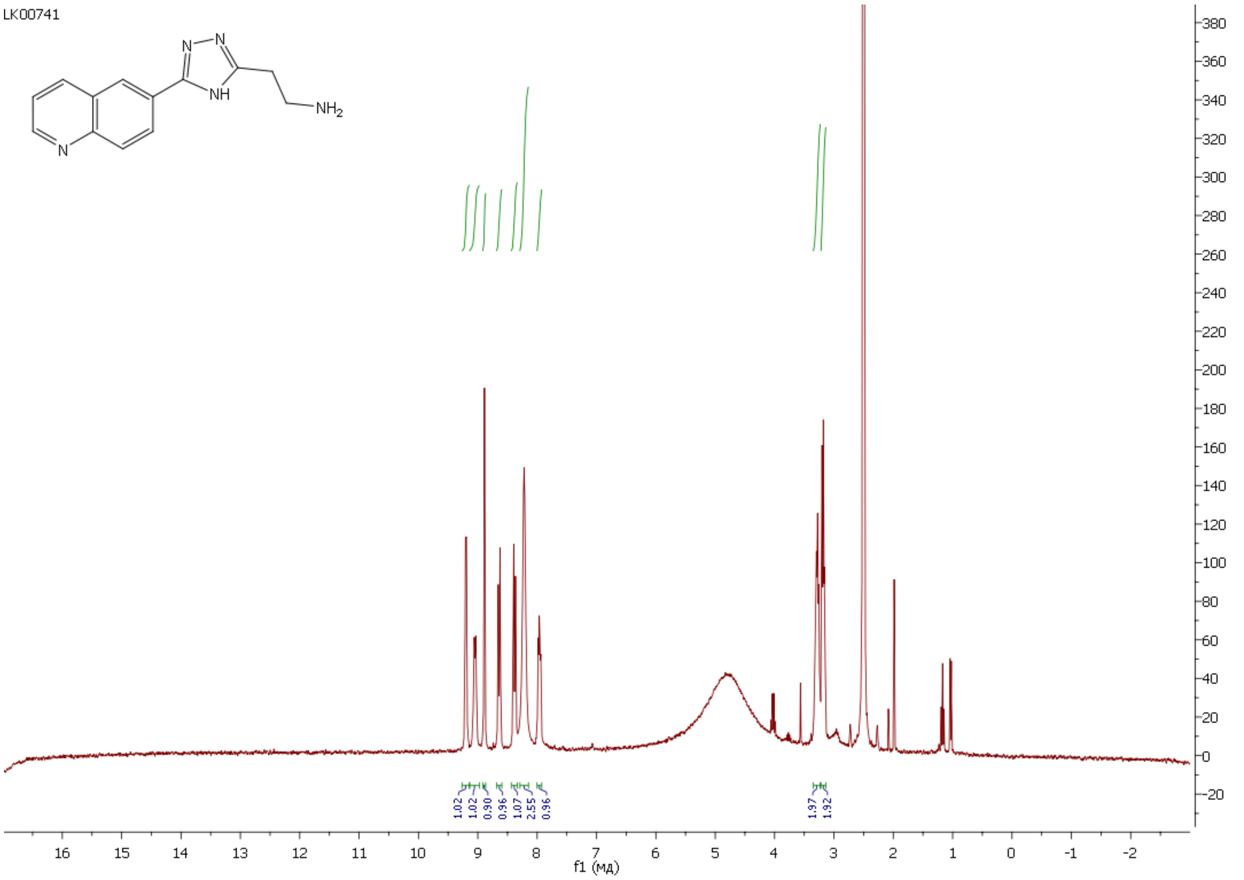

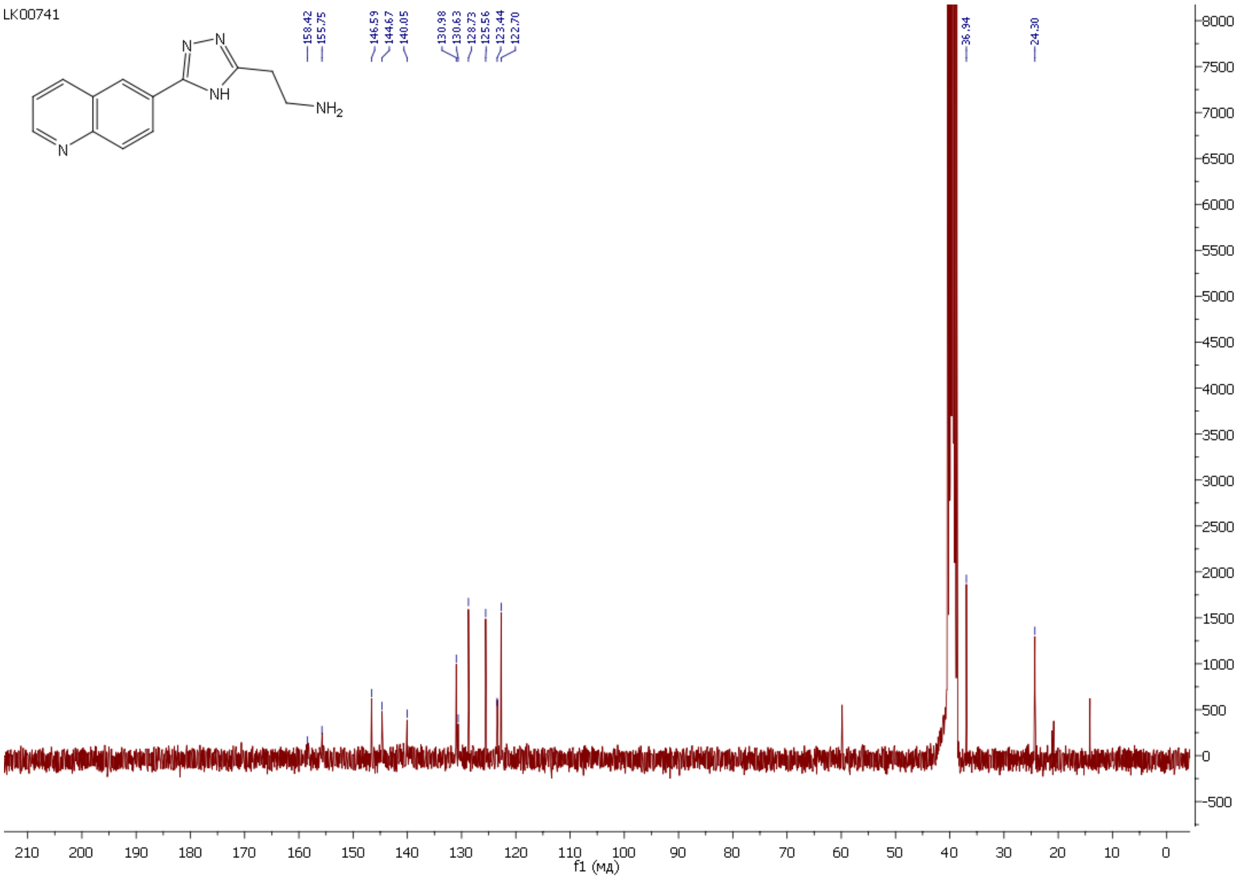

^{1}H and ^{13}C NMR spectra of compound **36**

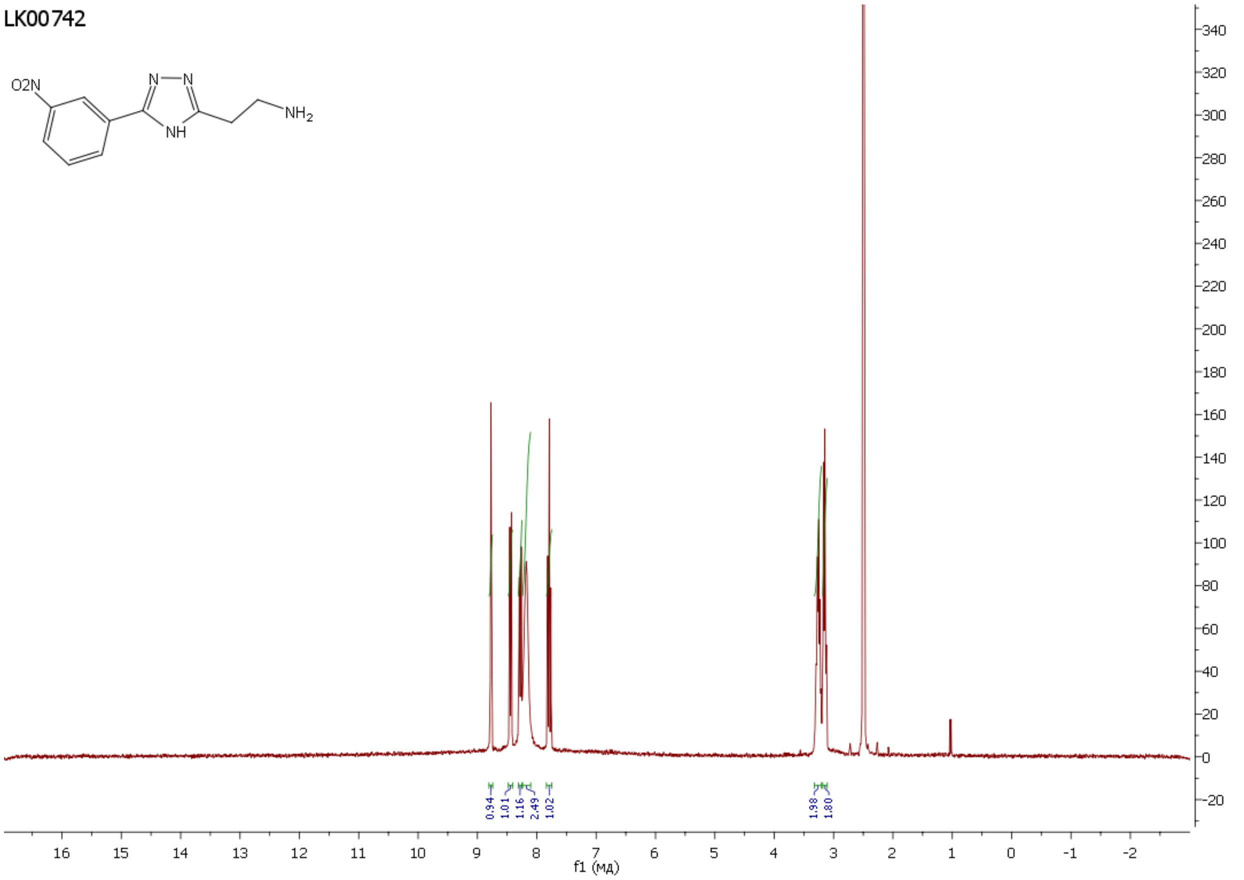

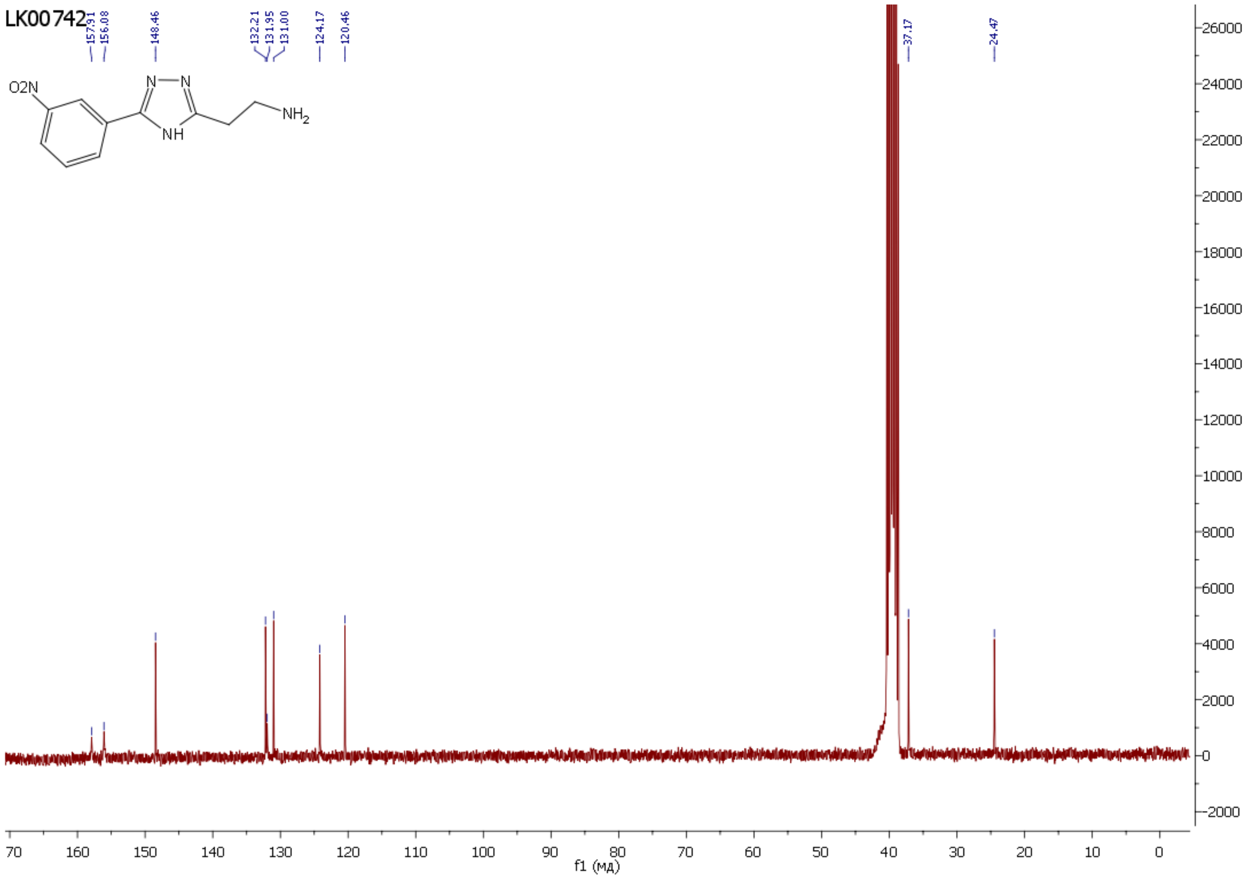

^{1}H and ^{13}C NMR spectra of compound **37**

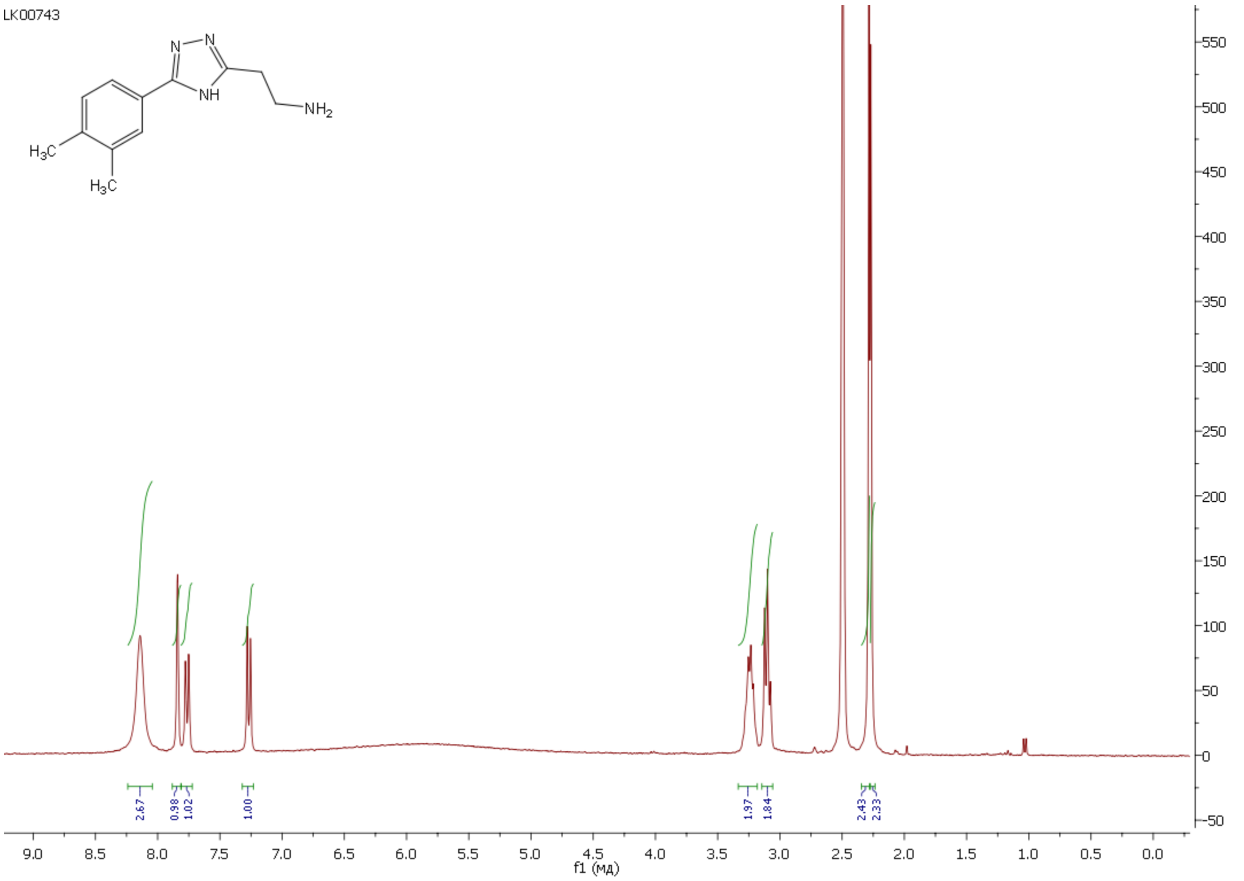

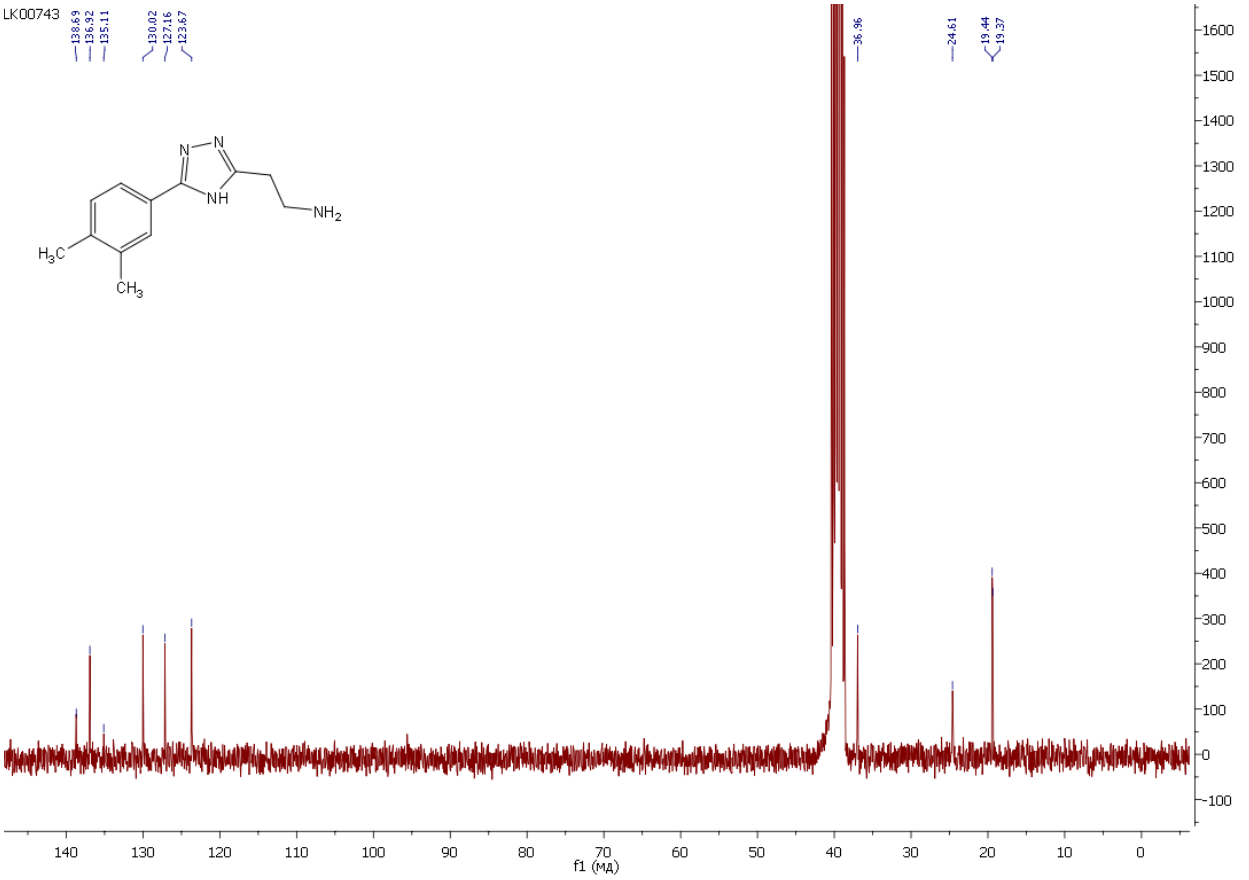

^{1}H and ^{13}C NMR spectra of compound **38**

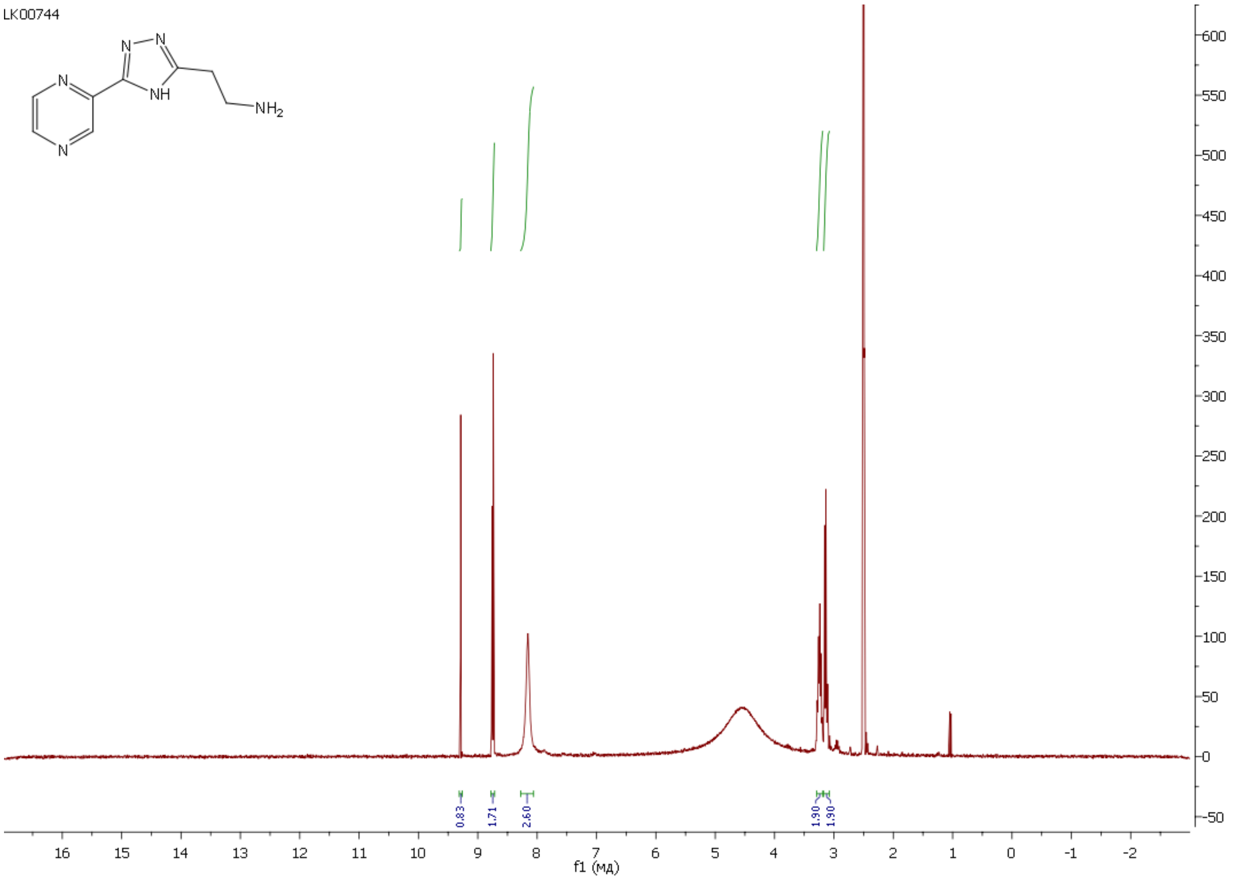

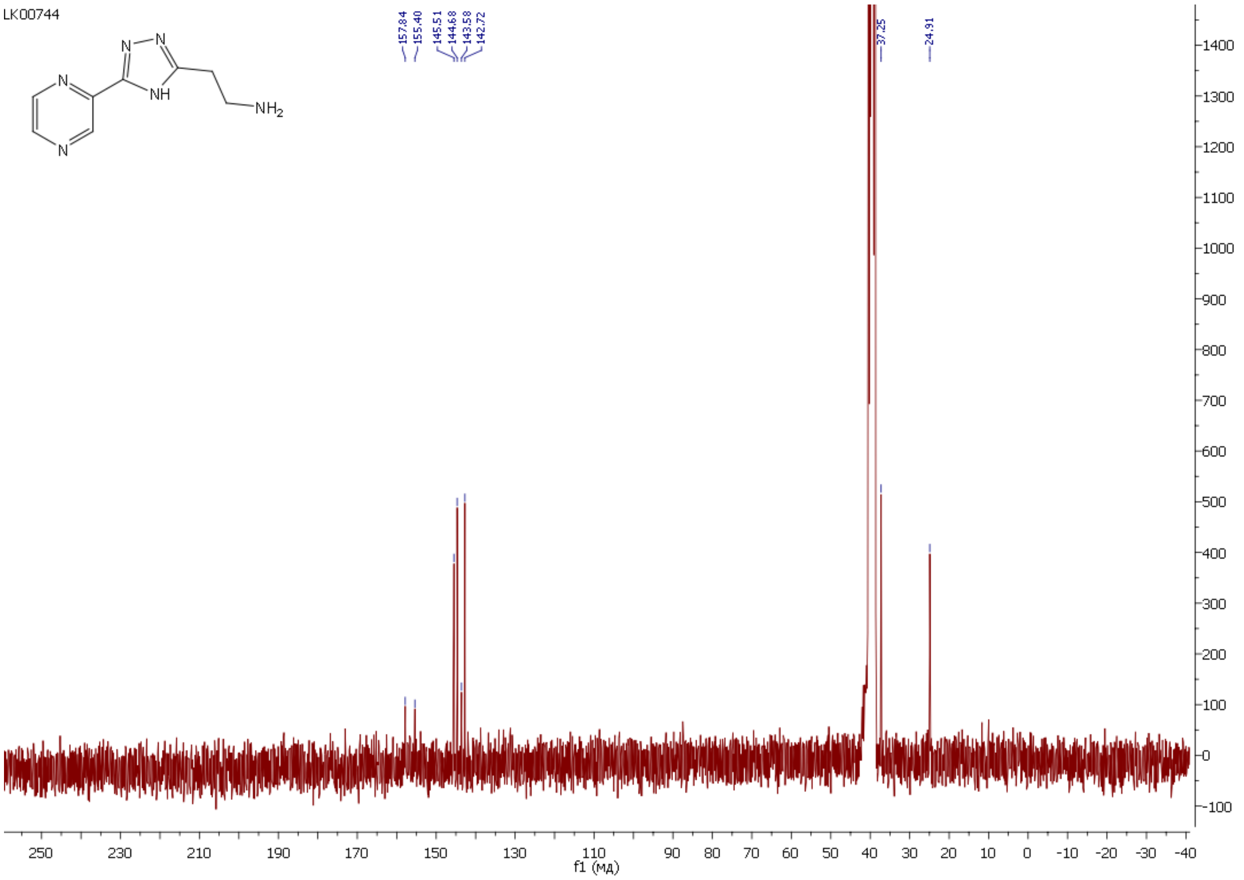

^{1}H and ^{13}C NMR spectra of compound **39**

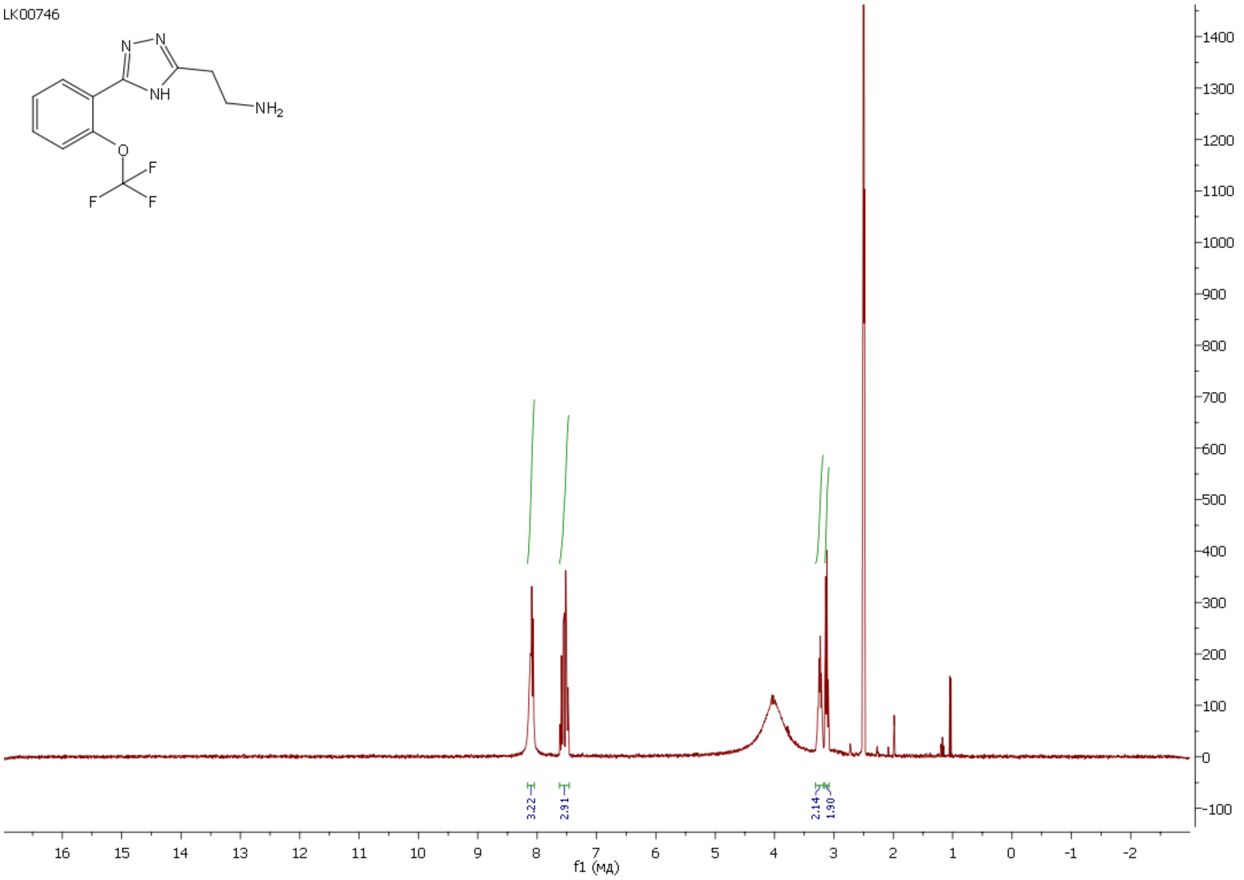

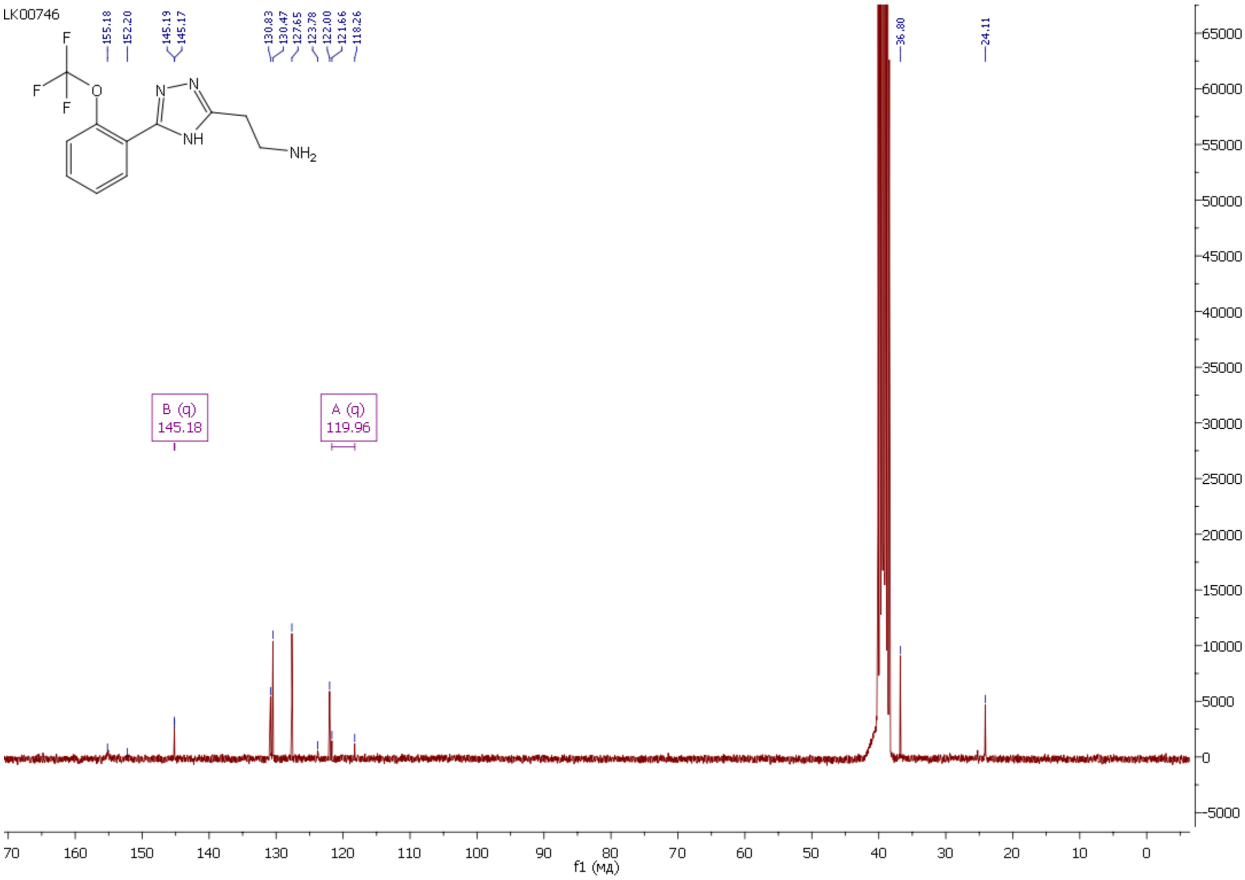

^{1}H and ^{13}C NMR spectra of compound **40**

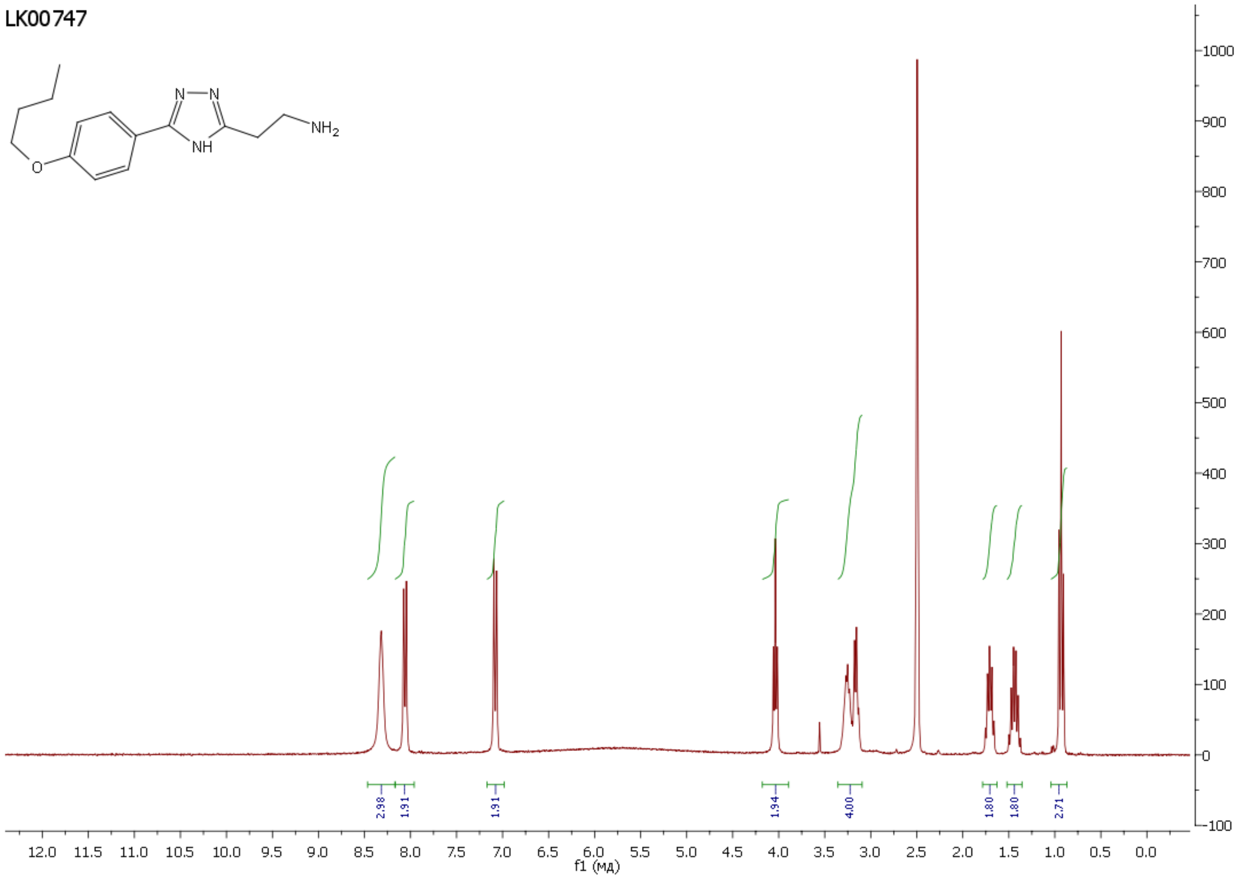

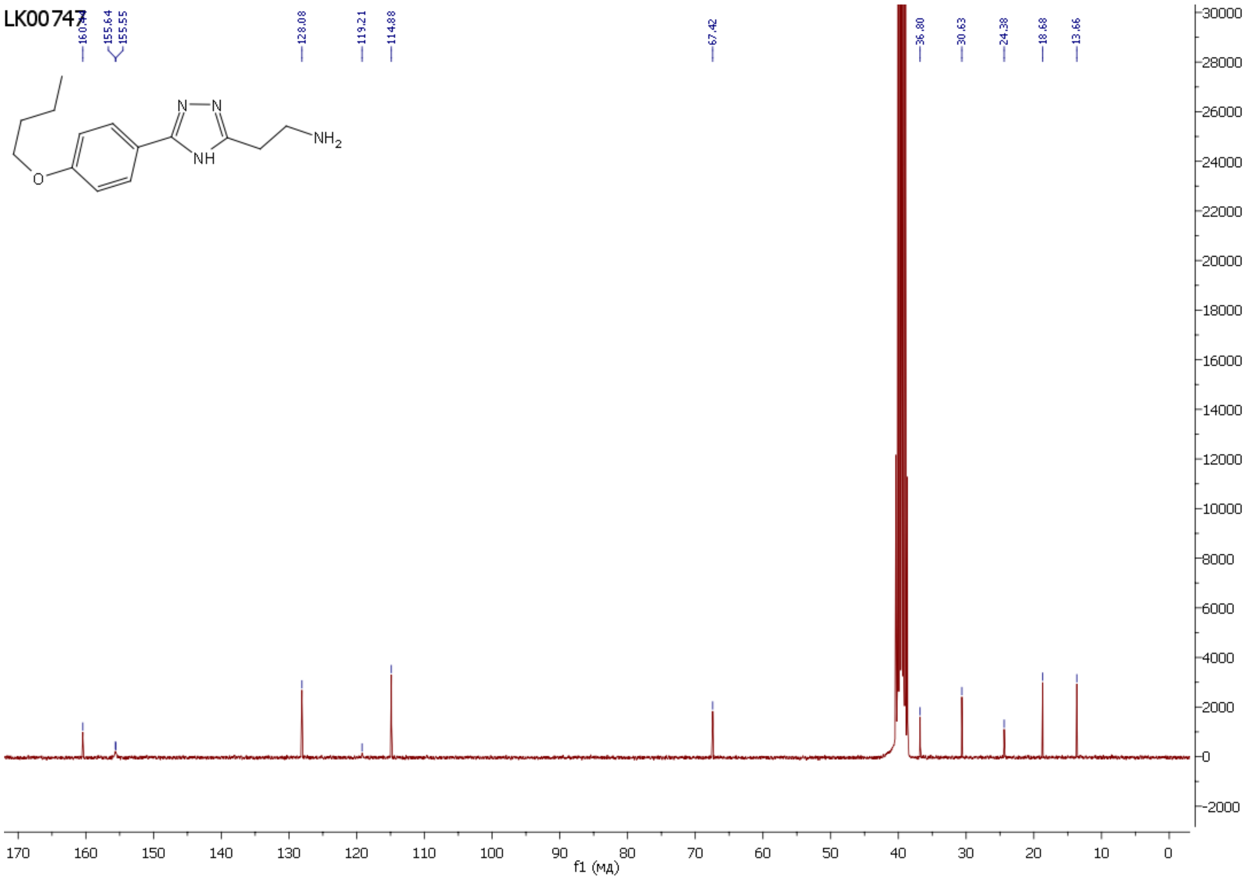

^{1}H and ^{13}C NMR spectra of compound **41**

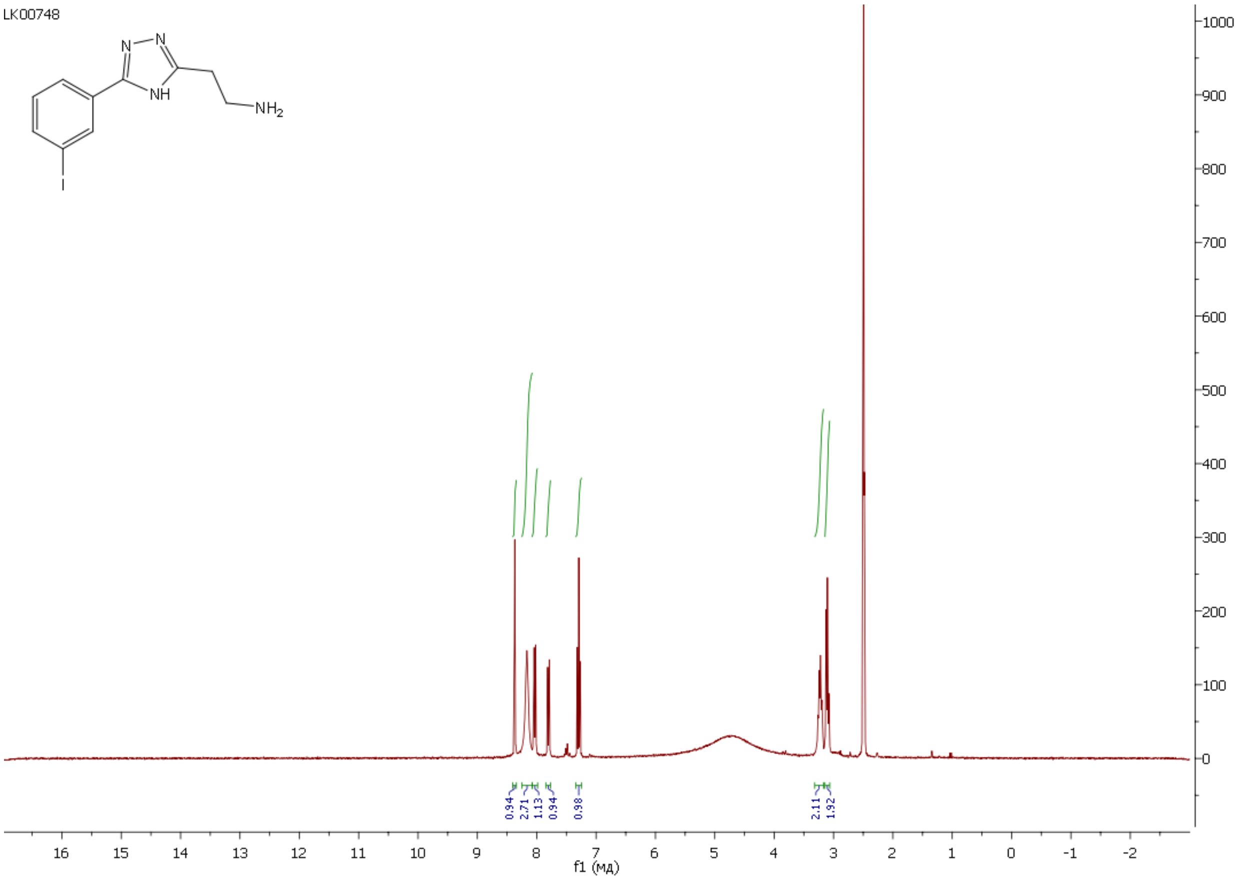

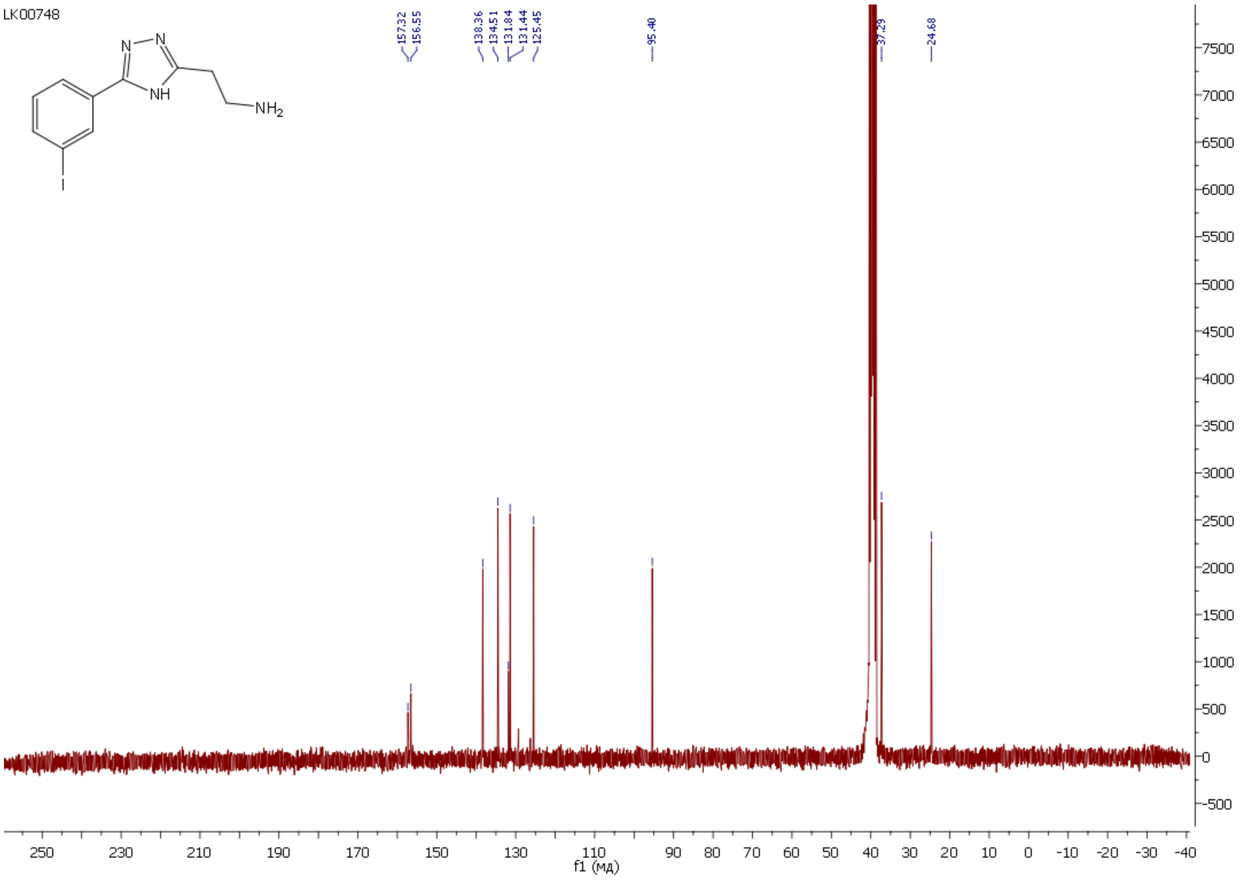

^{1}H and ^{13}C NMR spectra of compound **42**

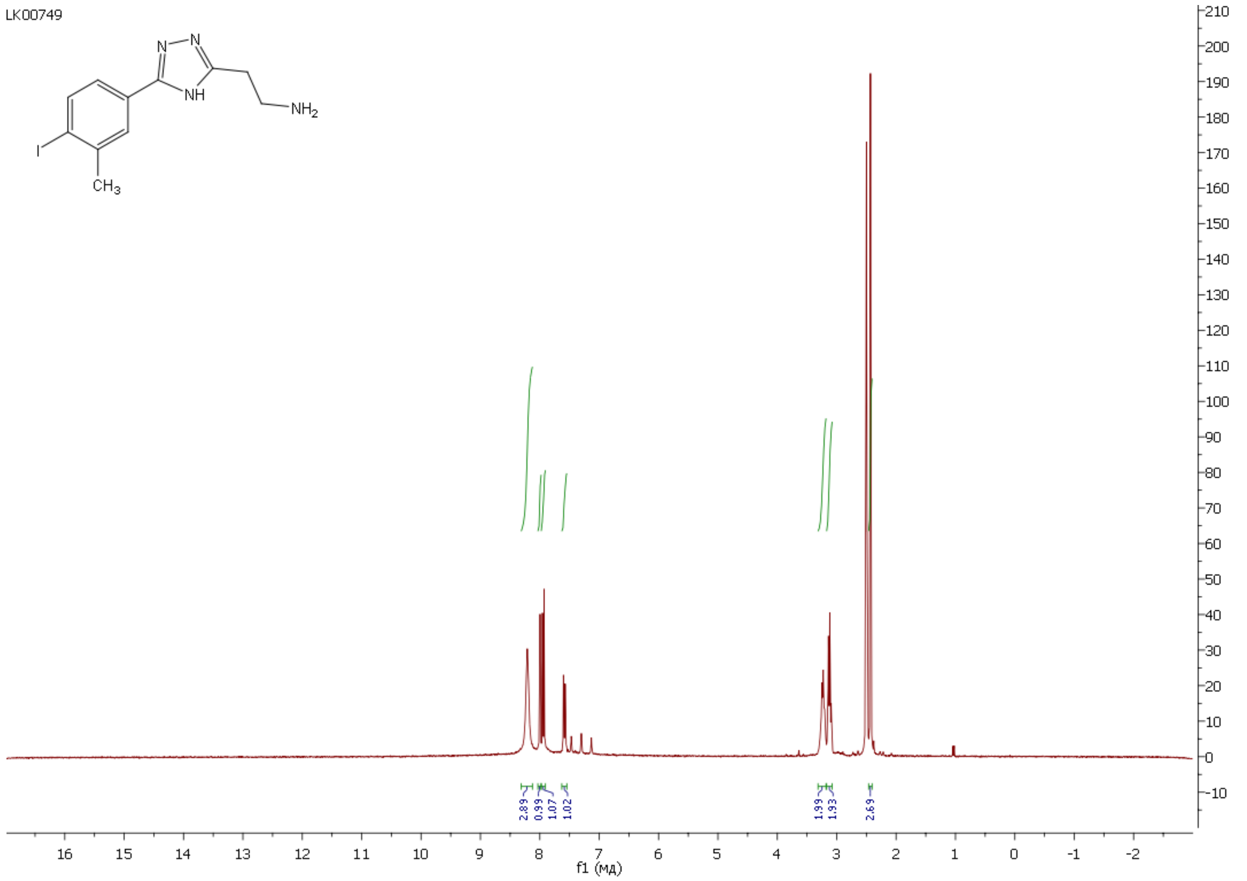

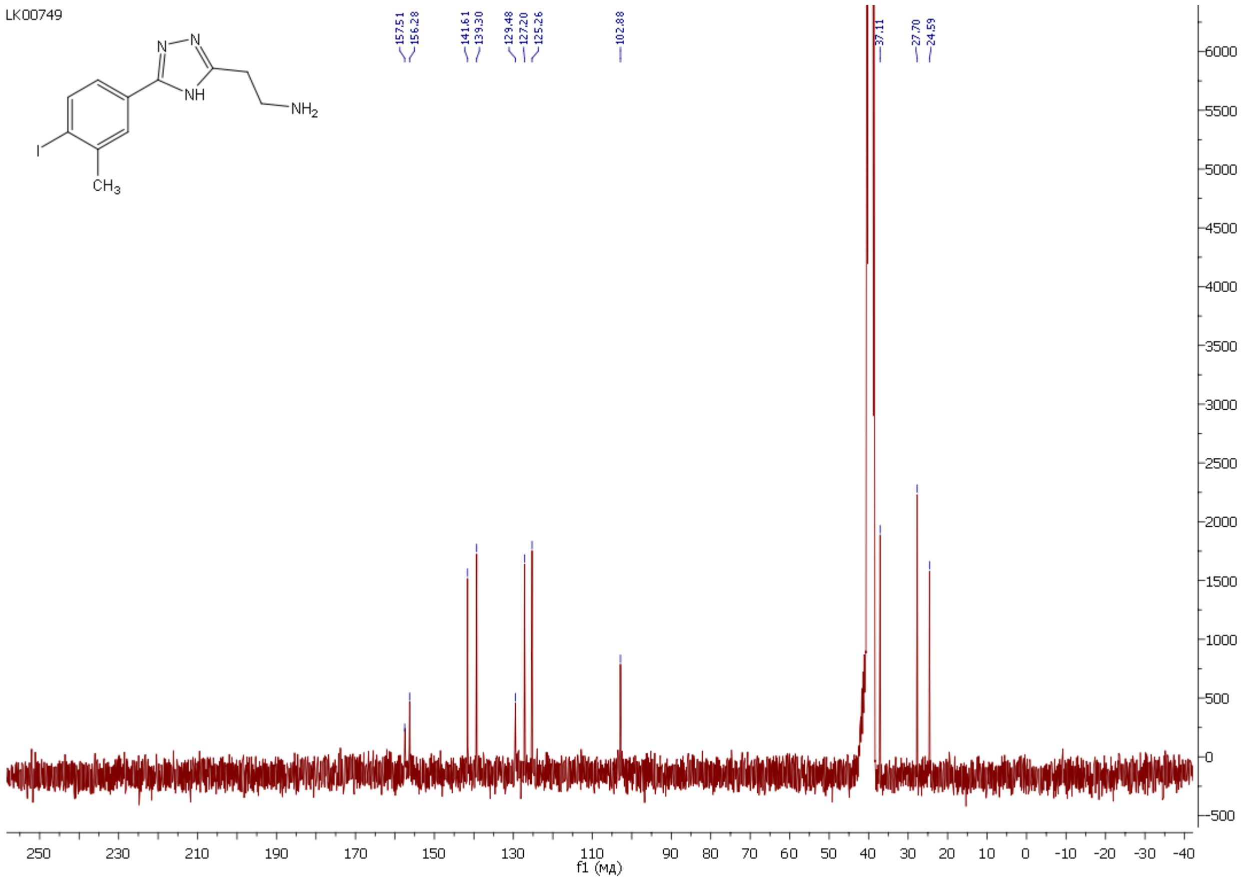

^{1}H and ^{13}C NMR spectra of compound **43**

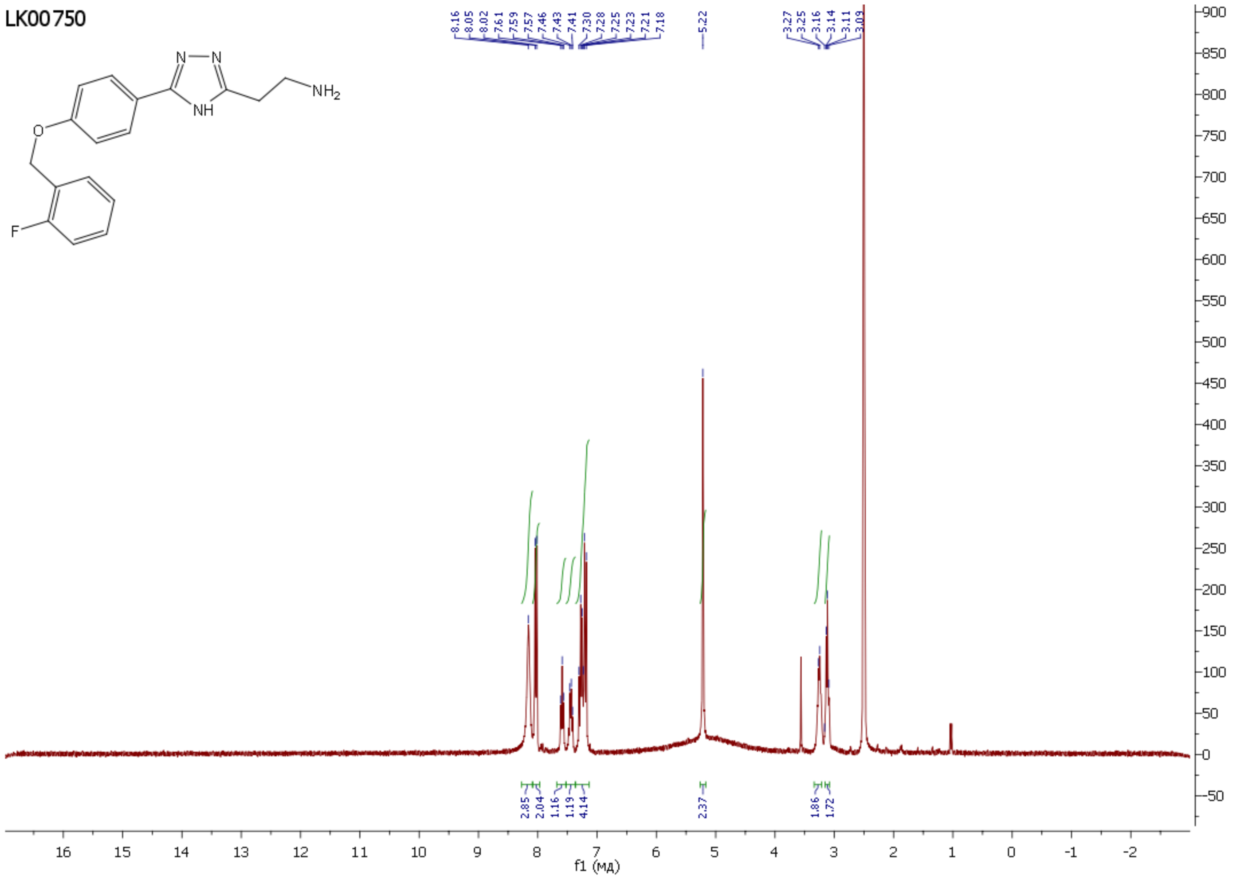

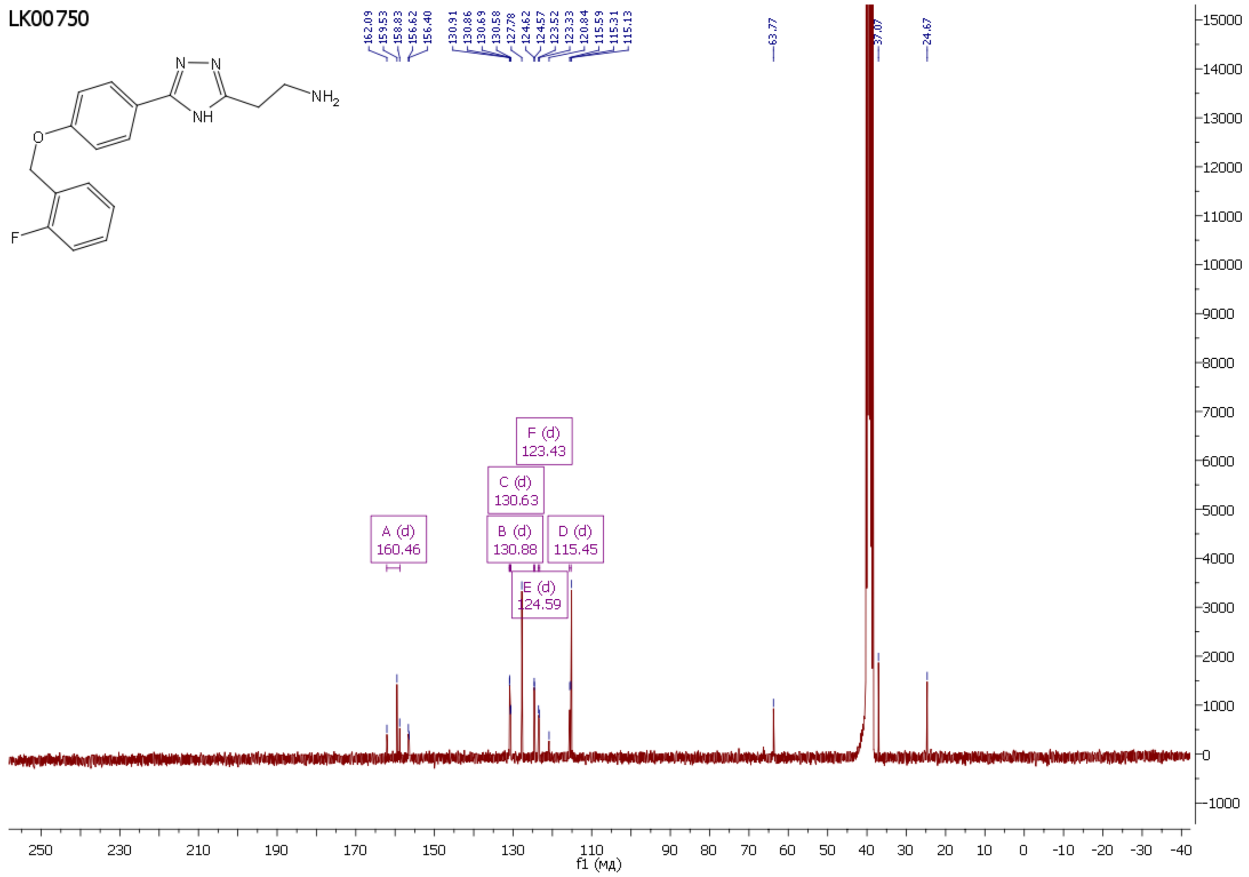

^{1}H and ^{13}C NMR spectra of compound **44**

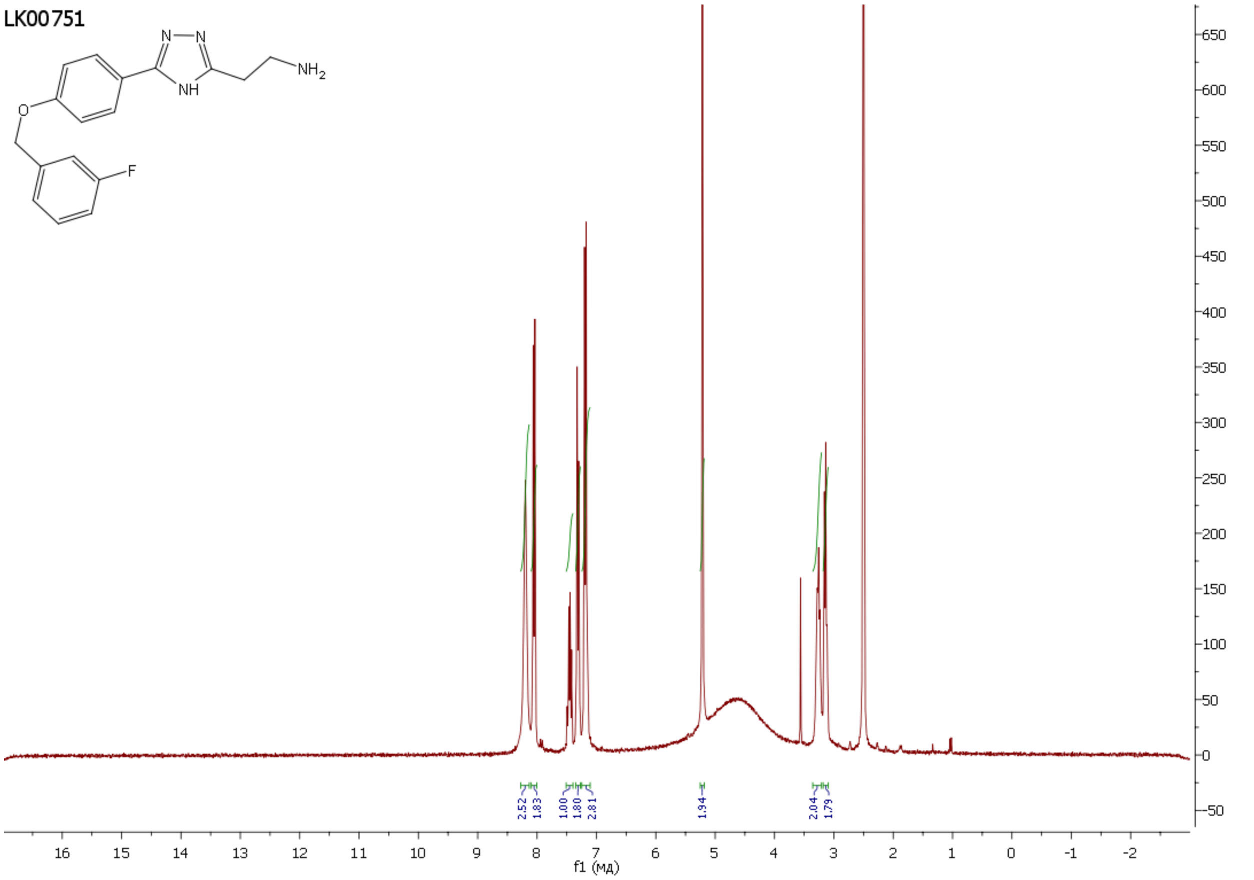

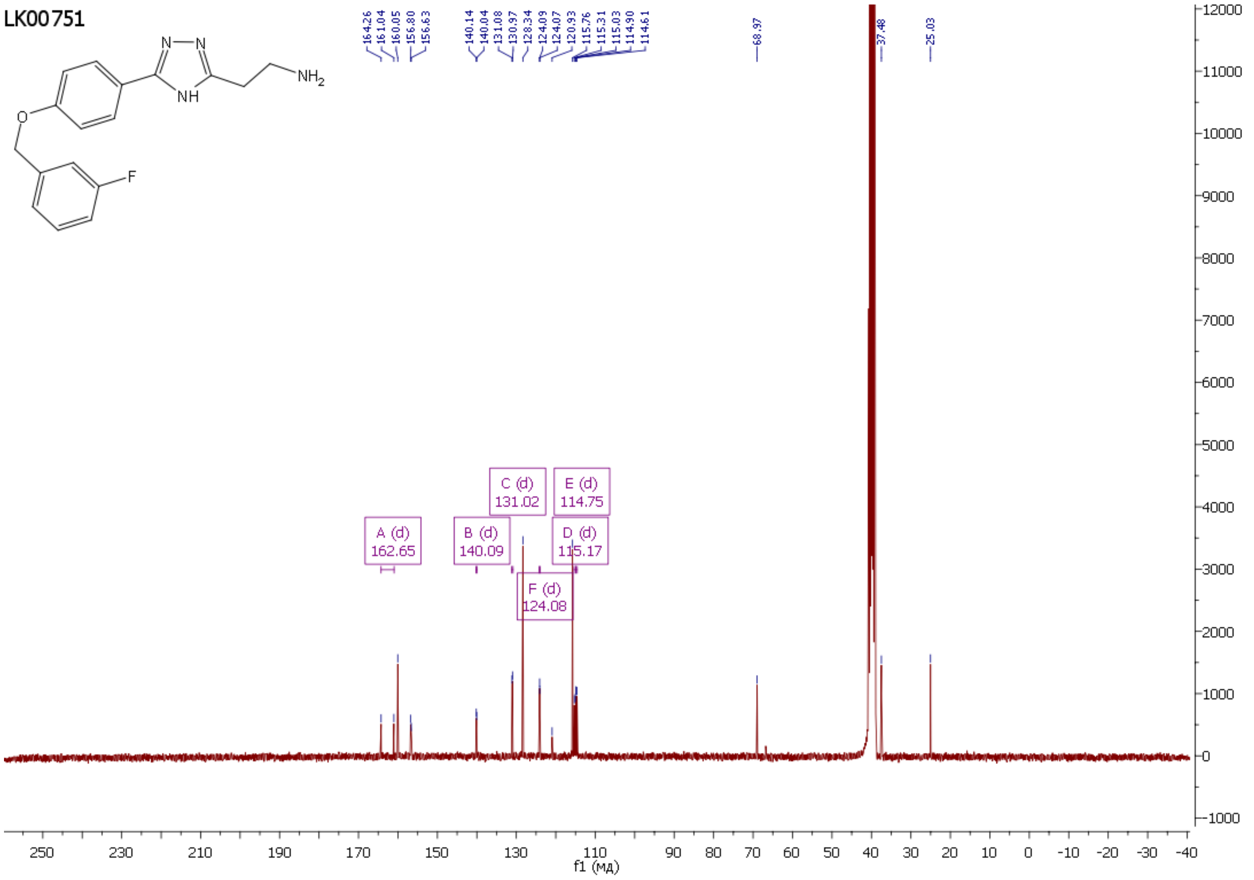

^{1}H and ^{13}C NMR spectra of compound **45**

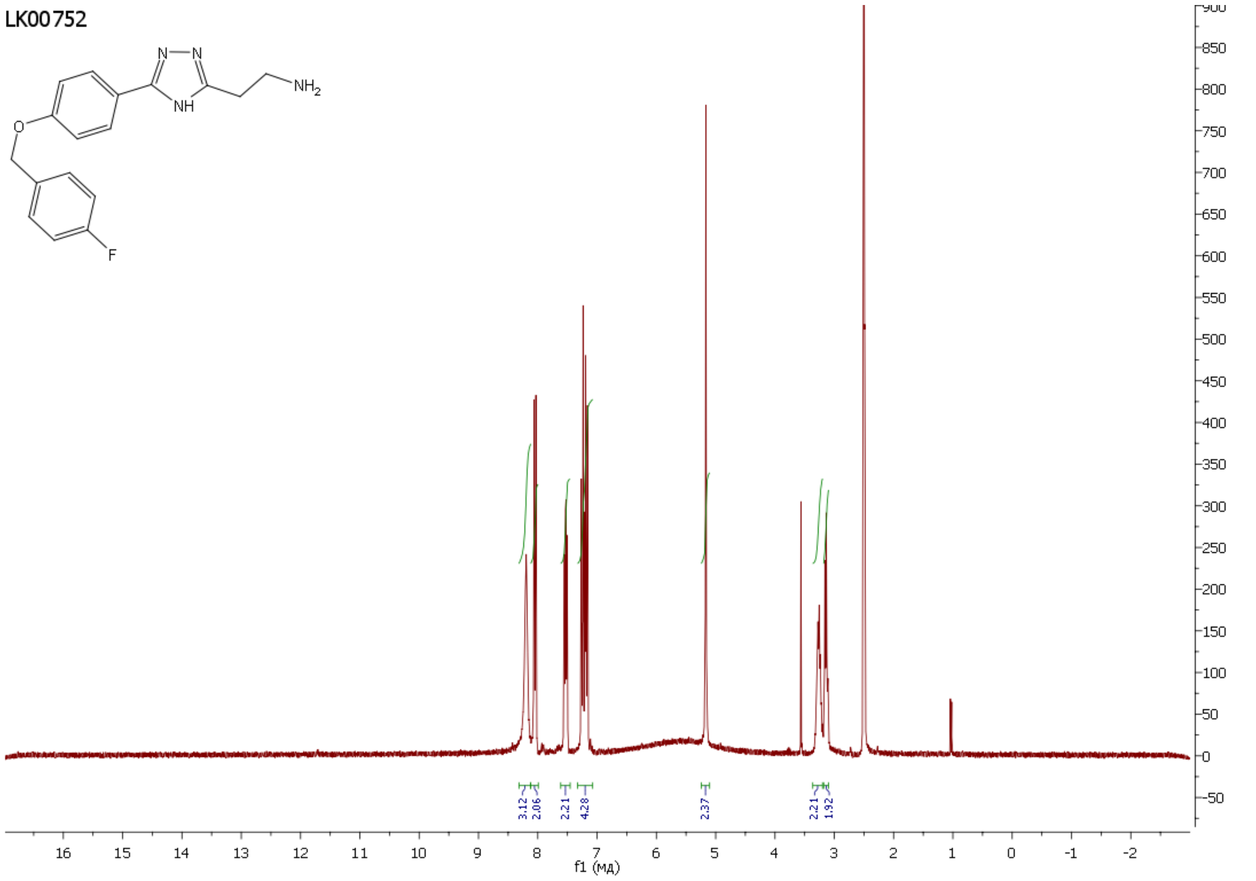

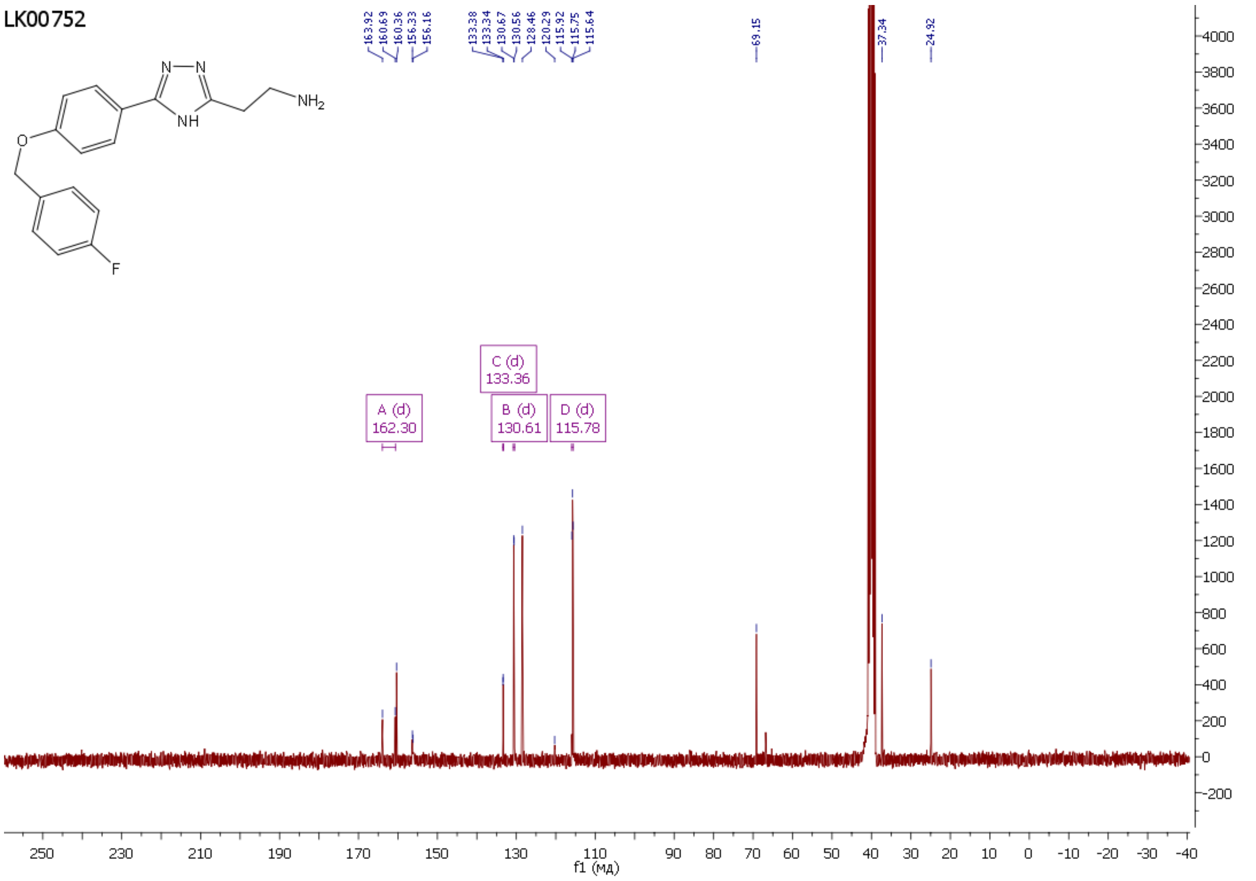

^{1}H and ^{13}C NMR spectra of compound **46**

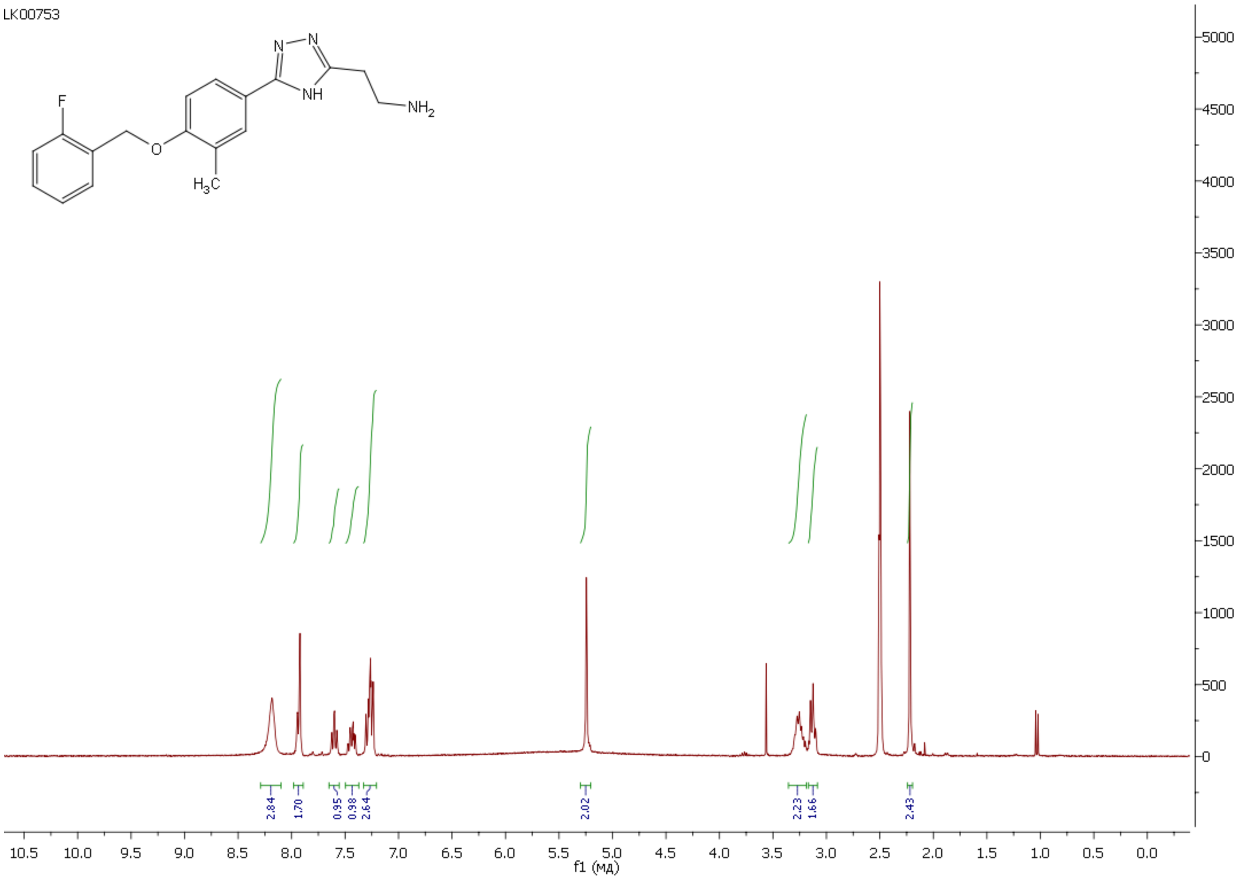

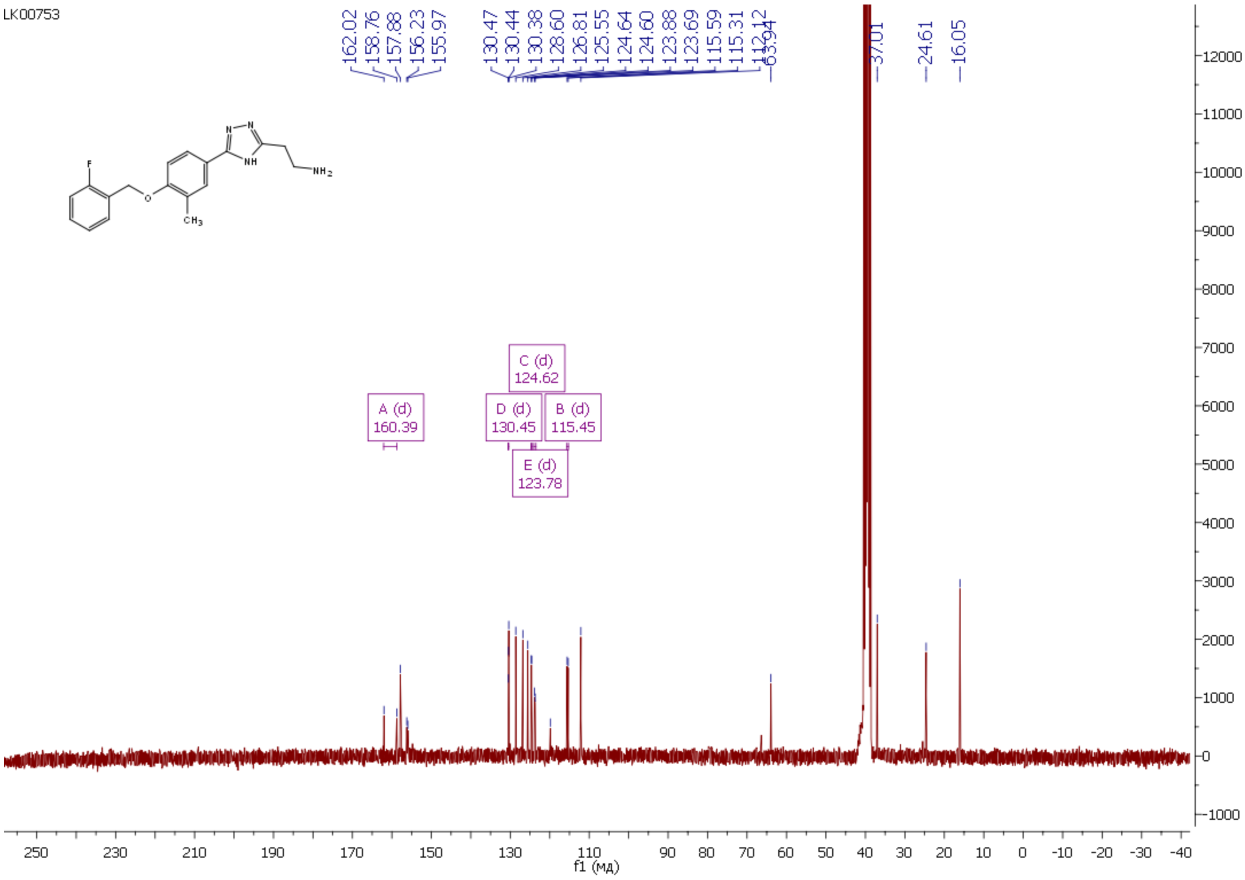

^{1}H and ^{13}C NMR spectra of compound **47**

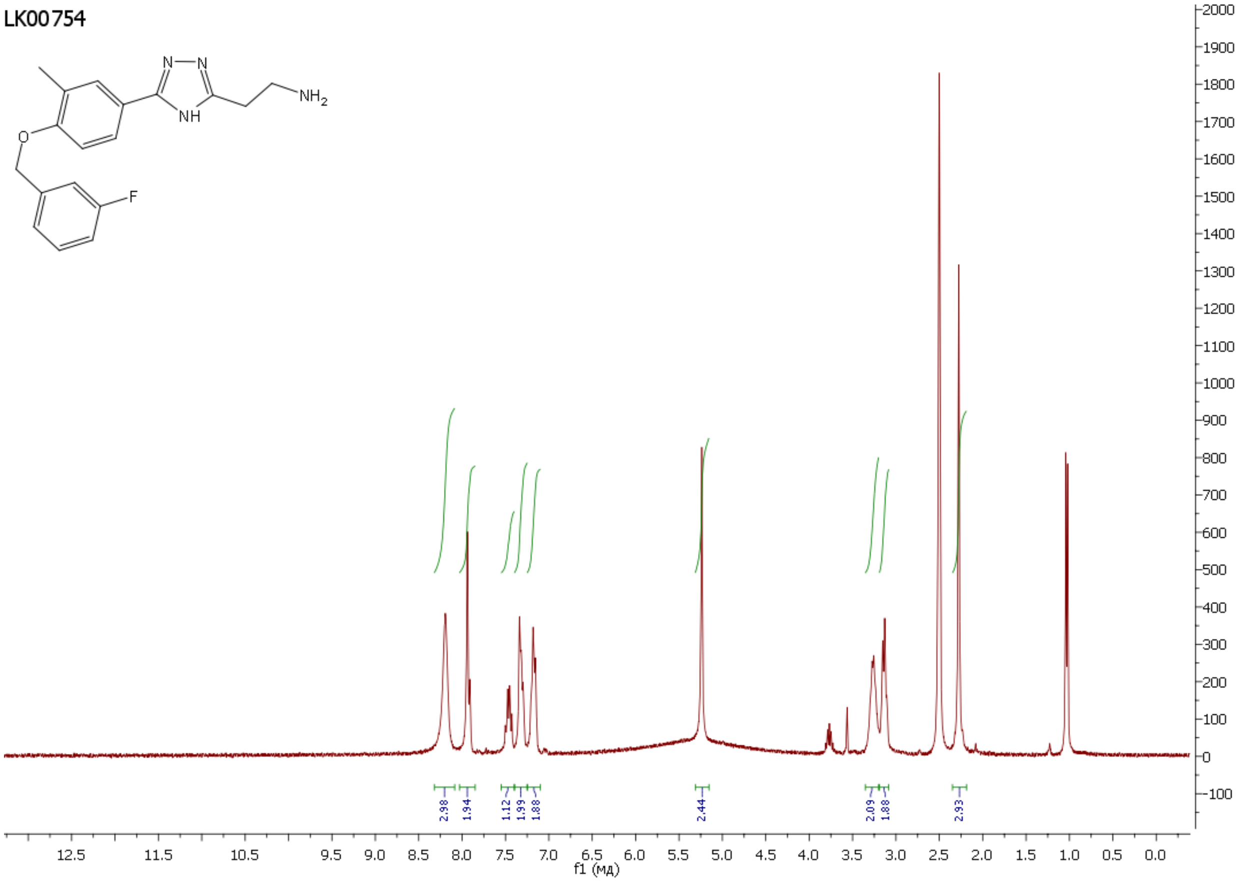

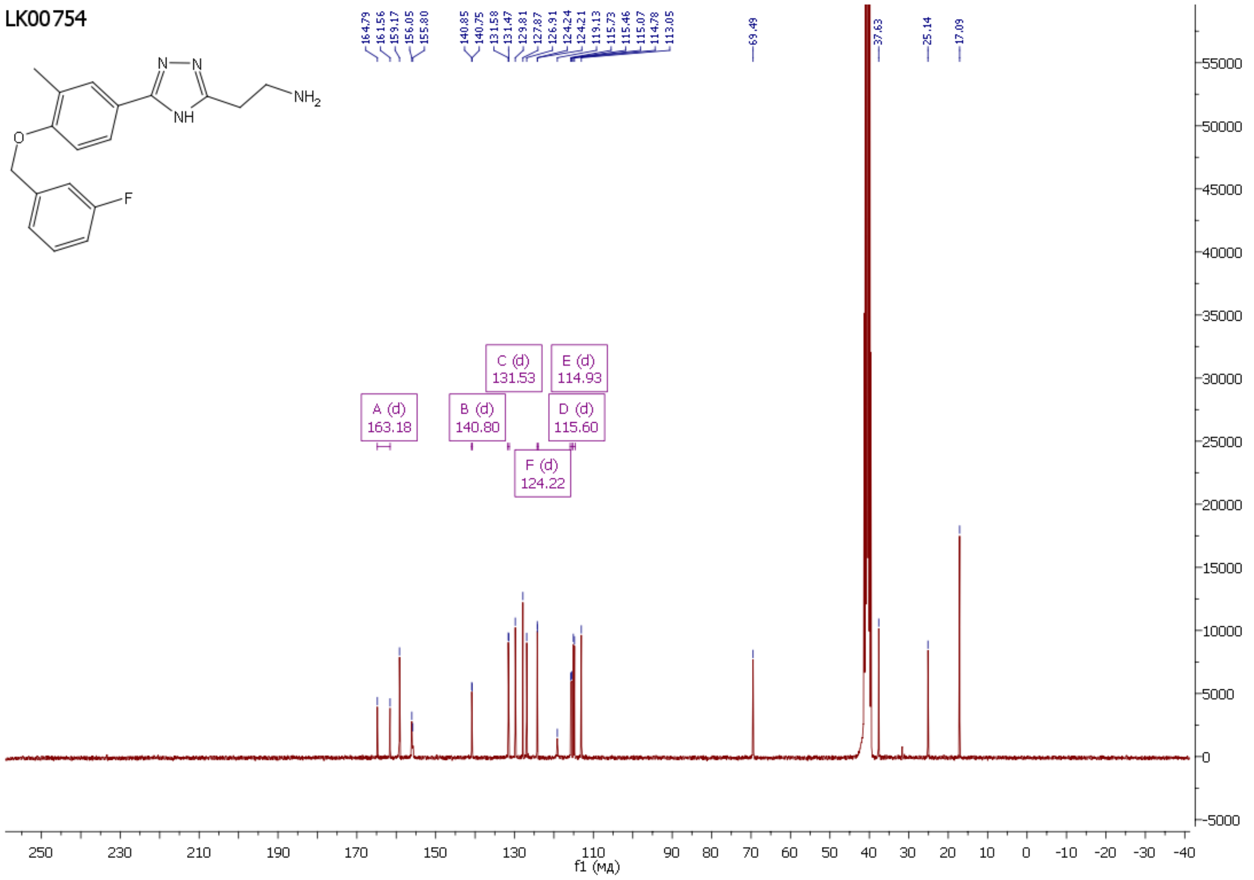

^{1}H and ^{13}C NMR spectra of compound **48**

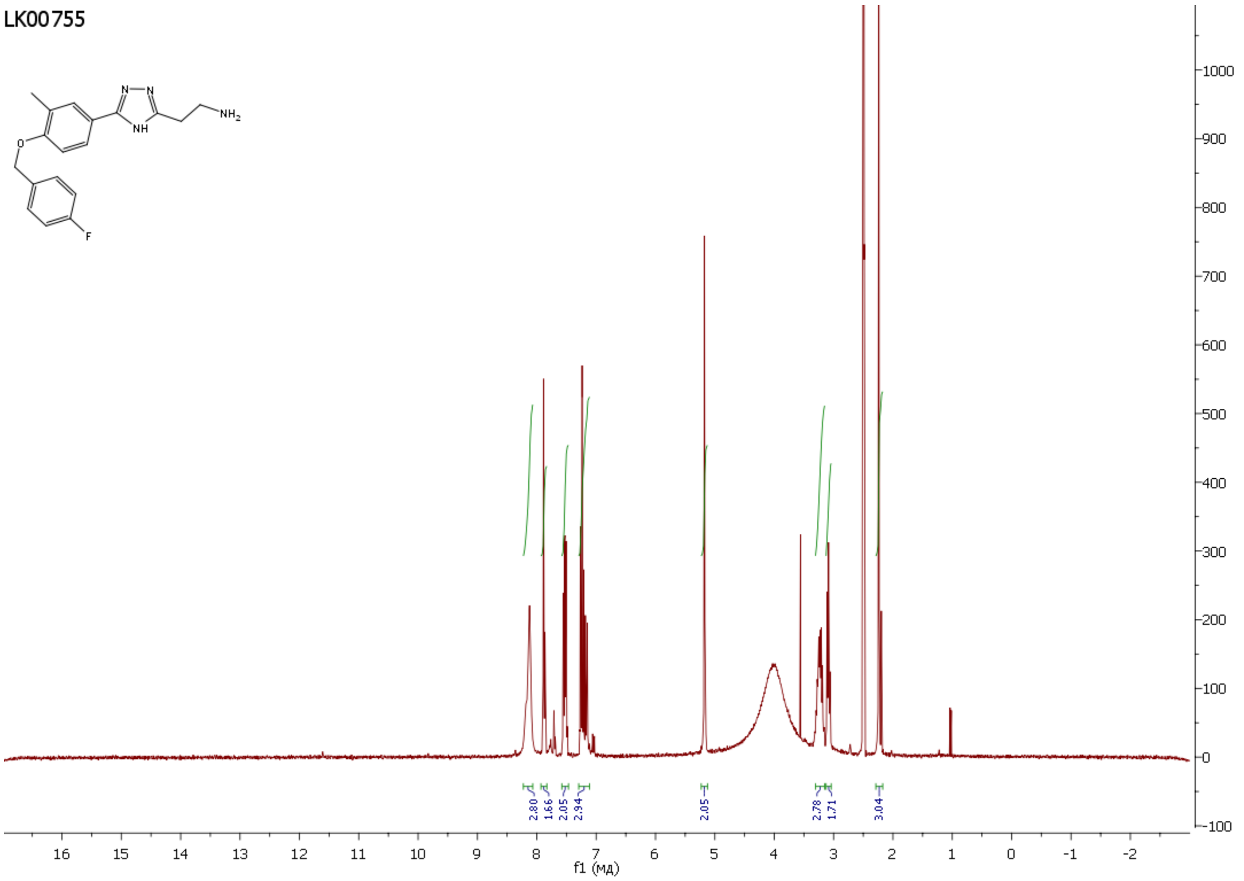

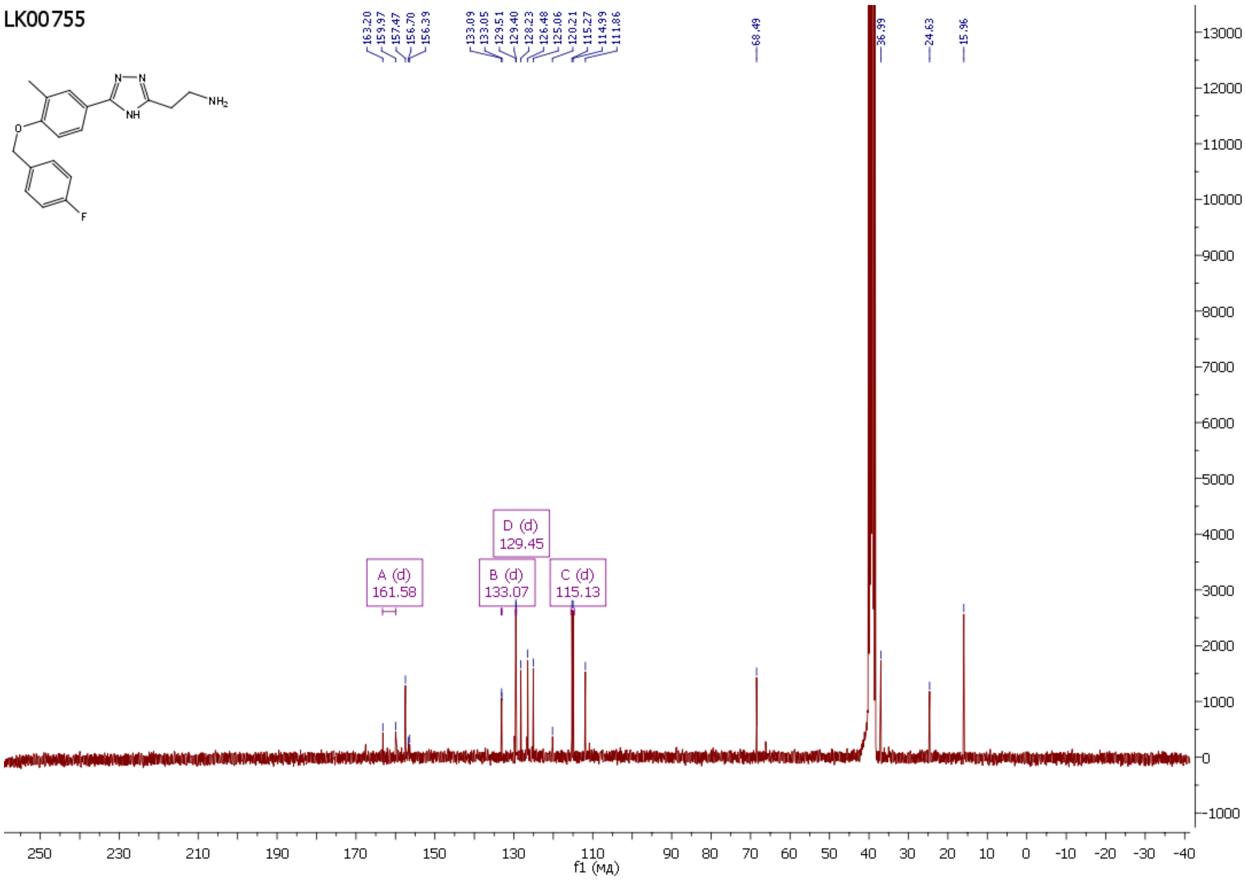

^{1}H and ^{13}C NMR spectra of compound **49**

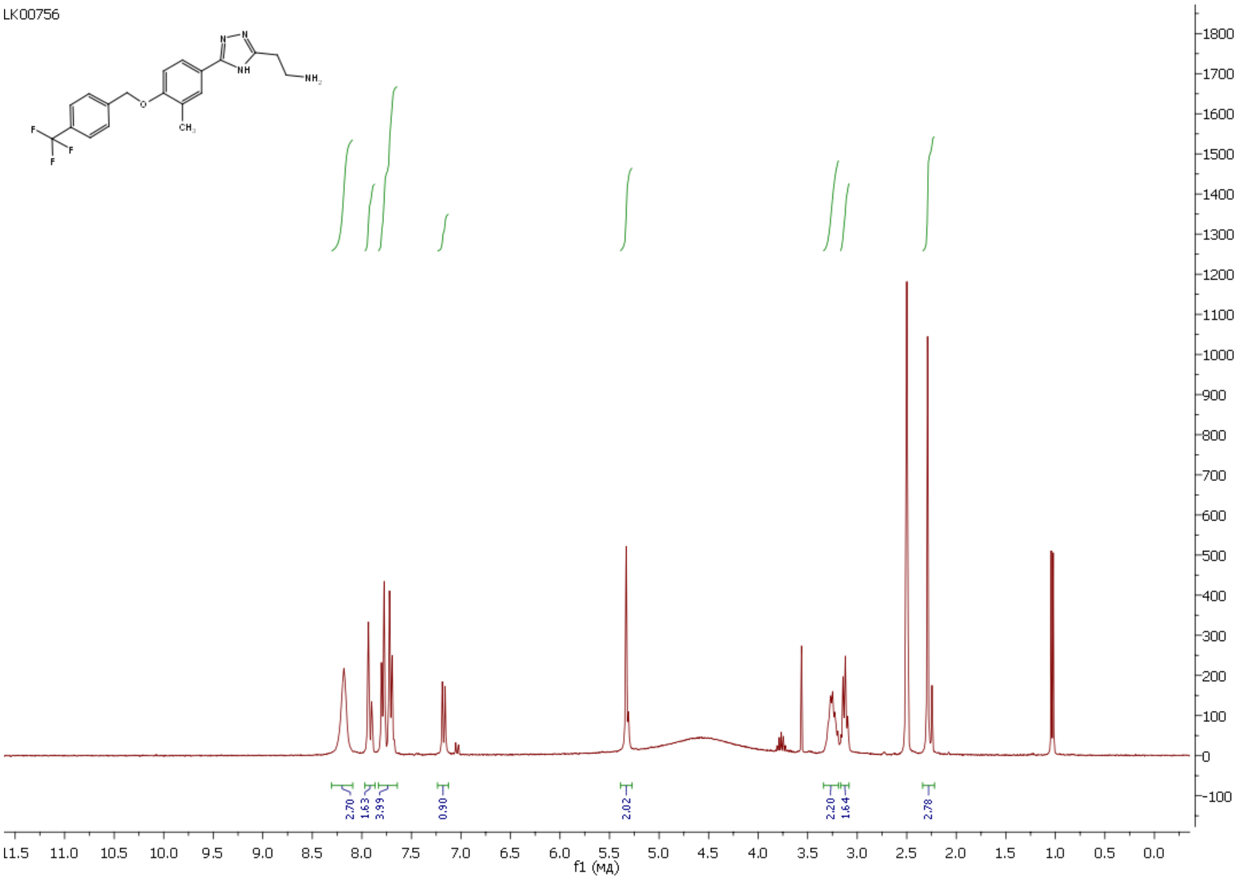

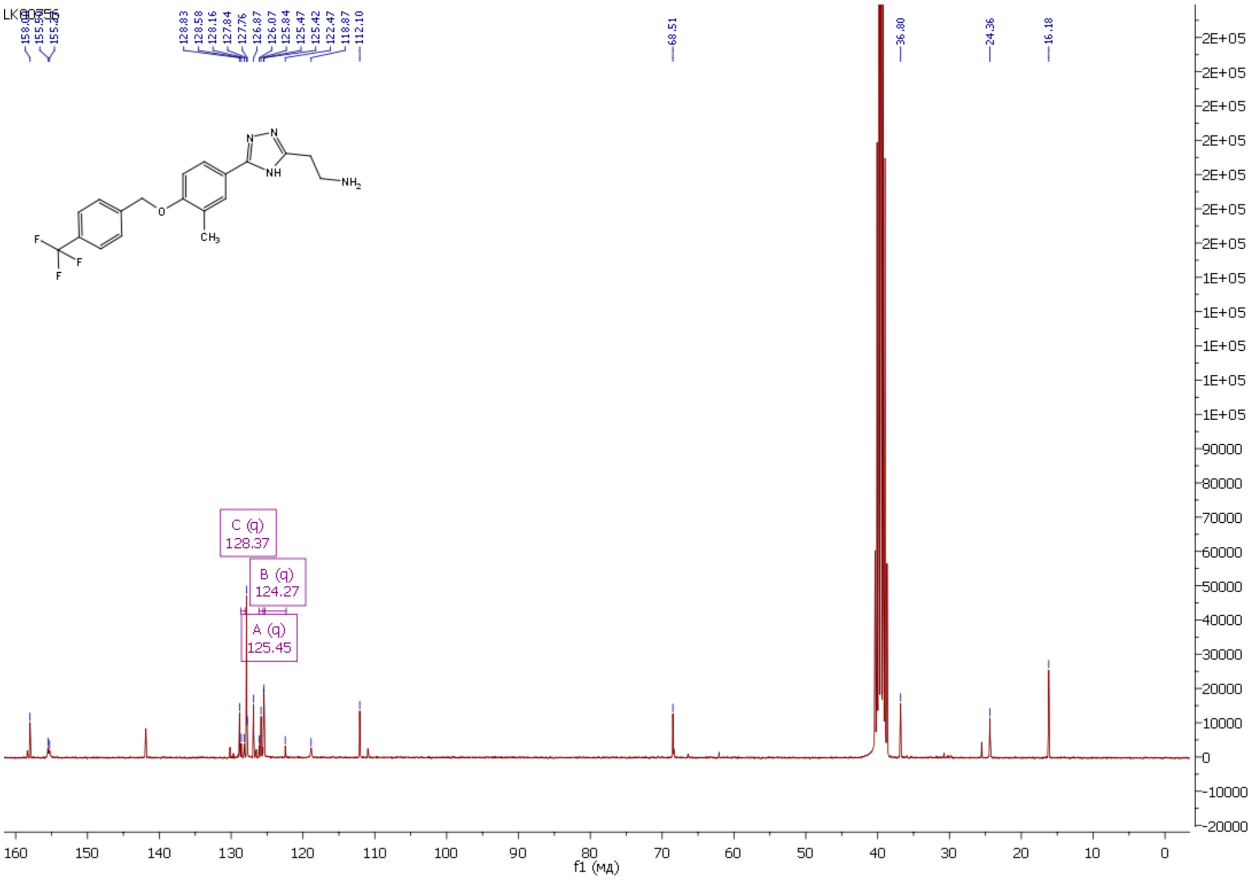

^{1}H and ^{13}C NMR spectra of compound **50**

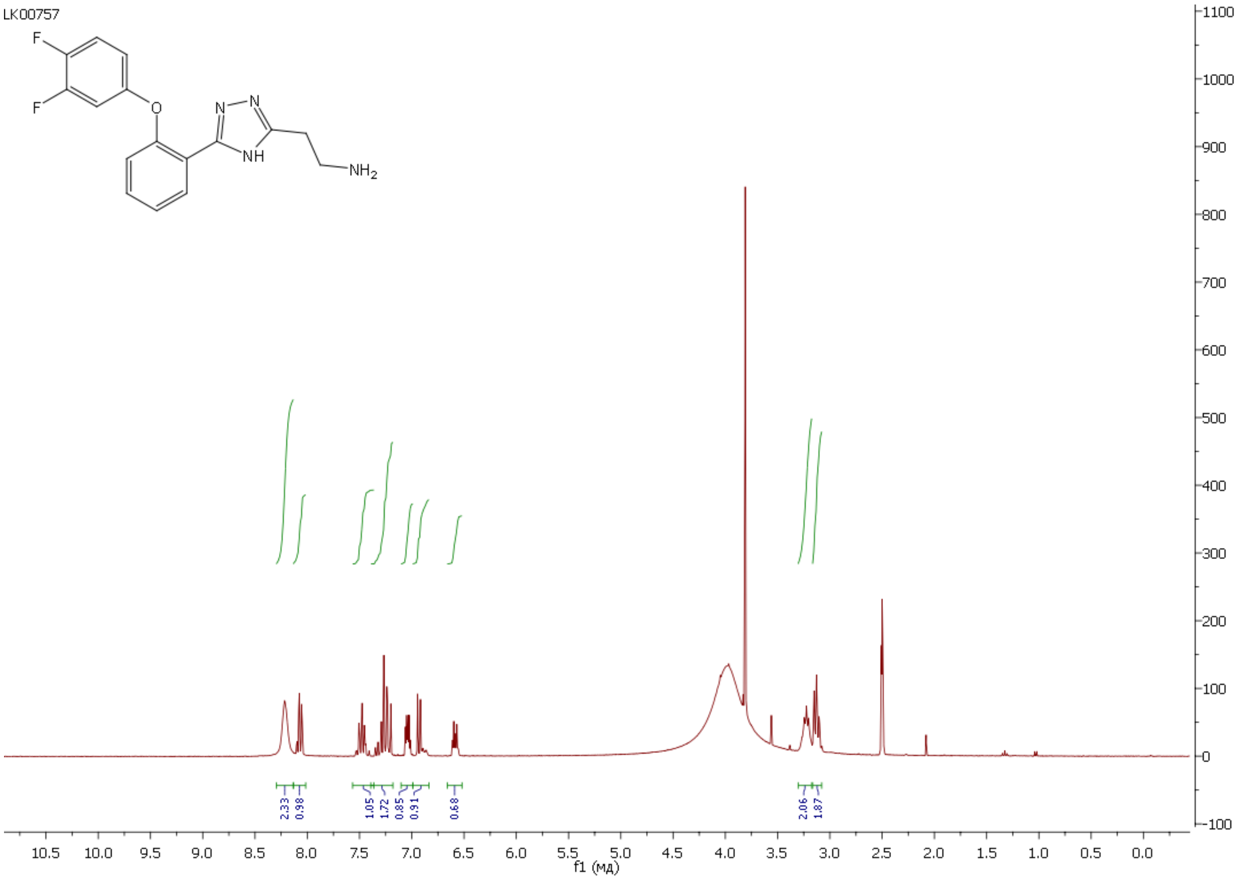

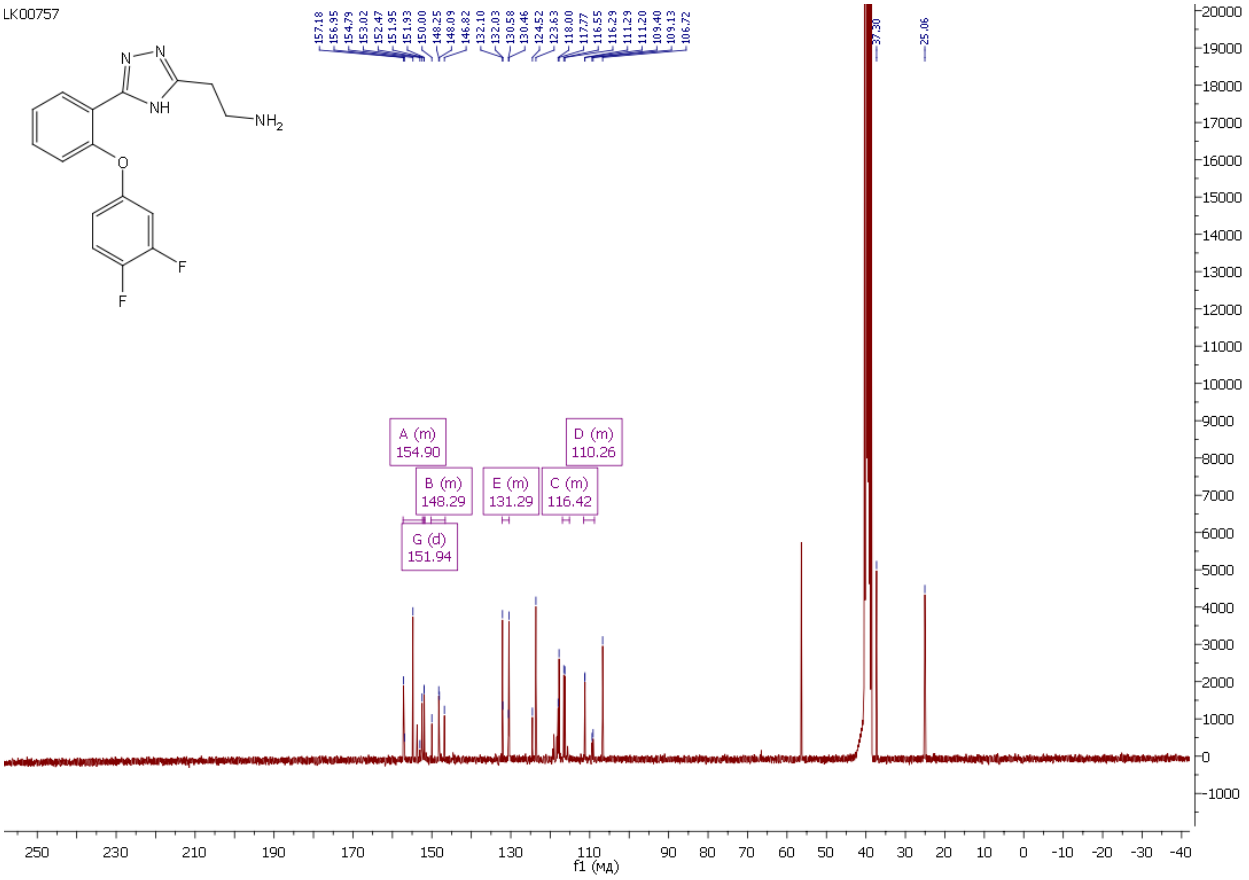

^{1}H and ^{13}C NMR spectra of compound **51**

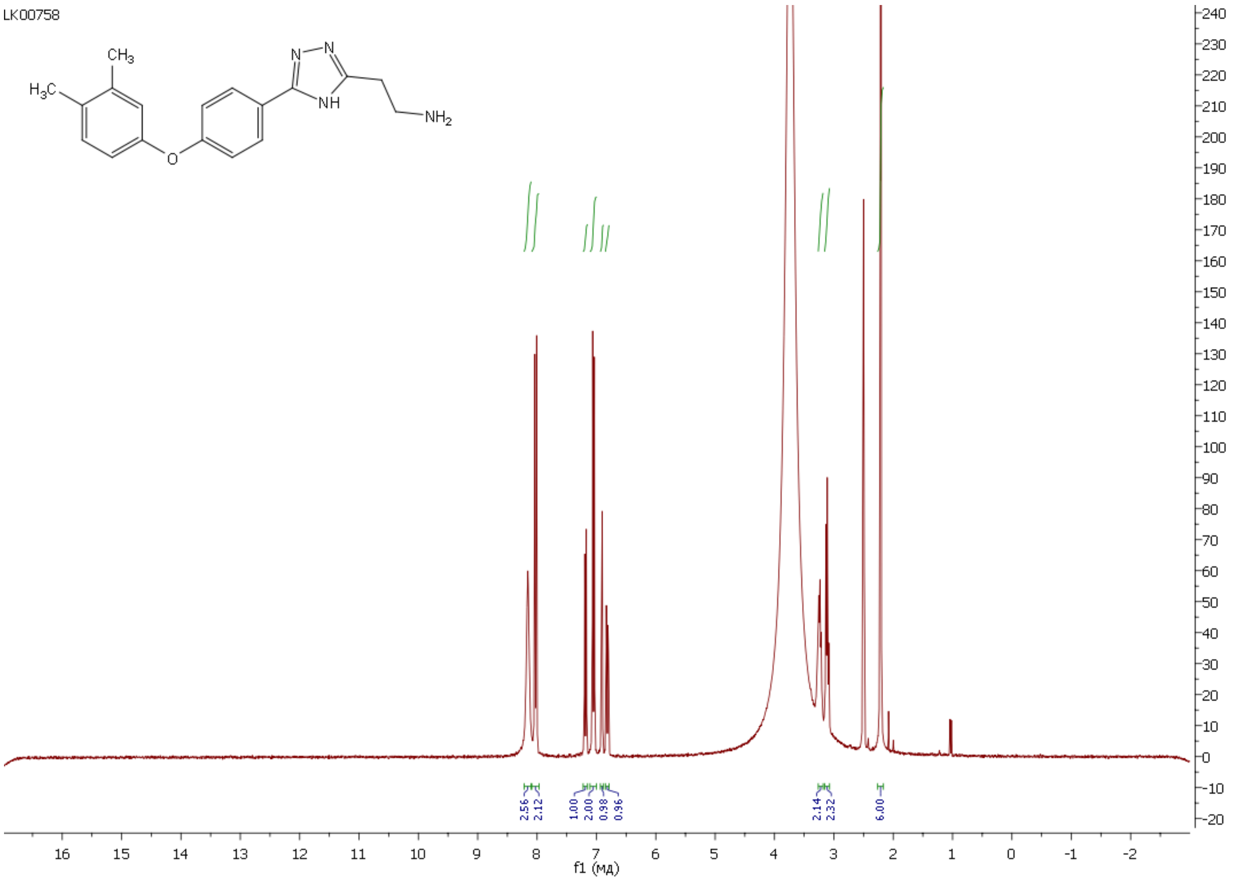

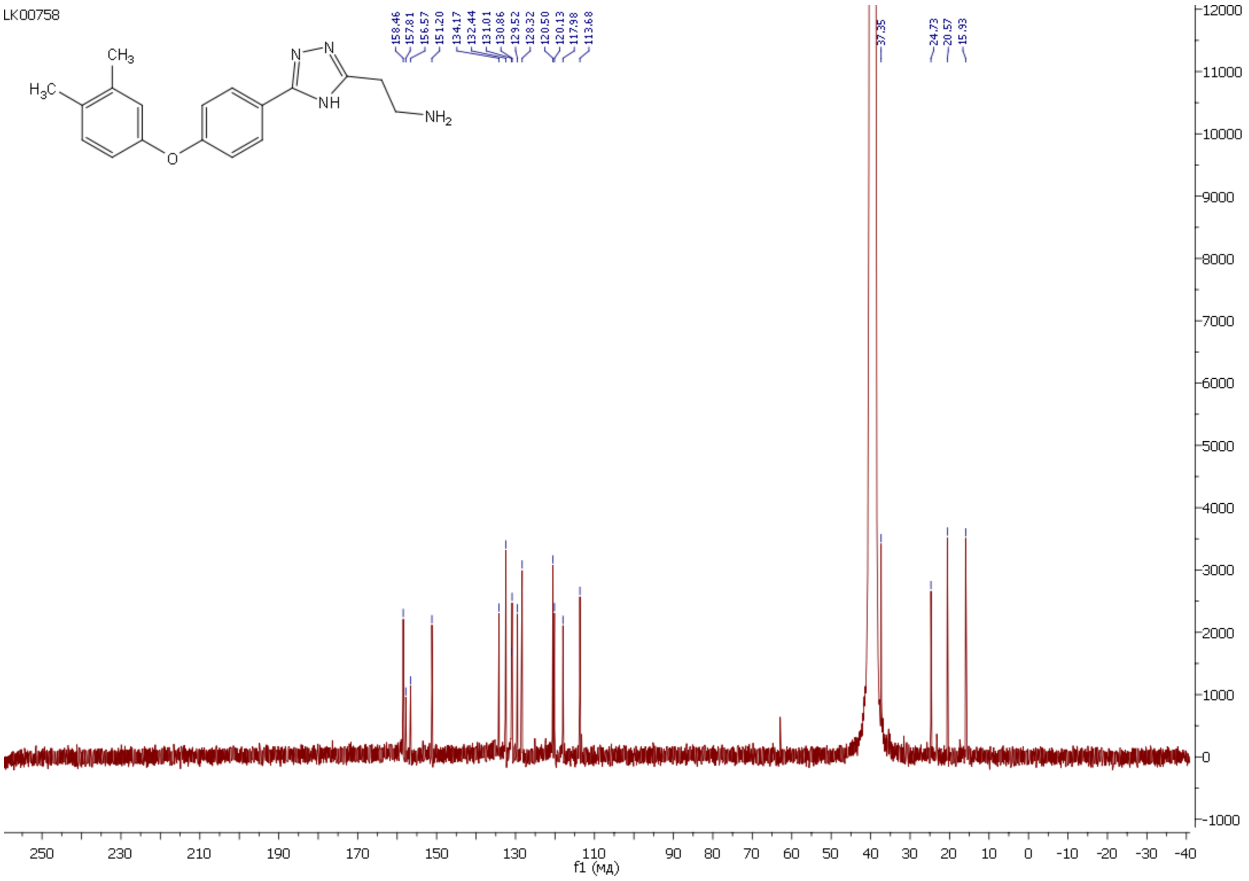

^{1}H and ^{13}C NMR spectra of compound **52**

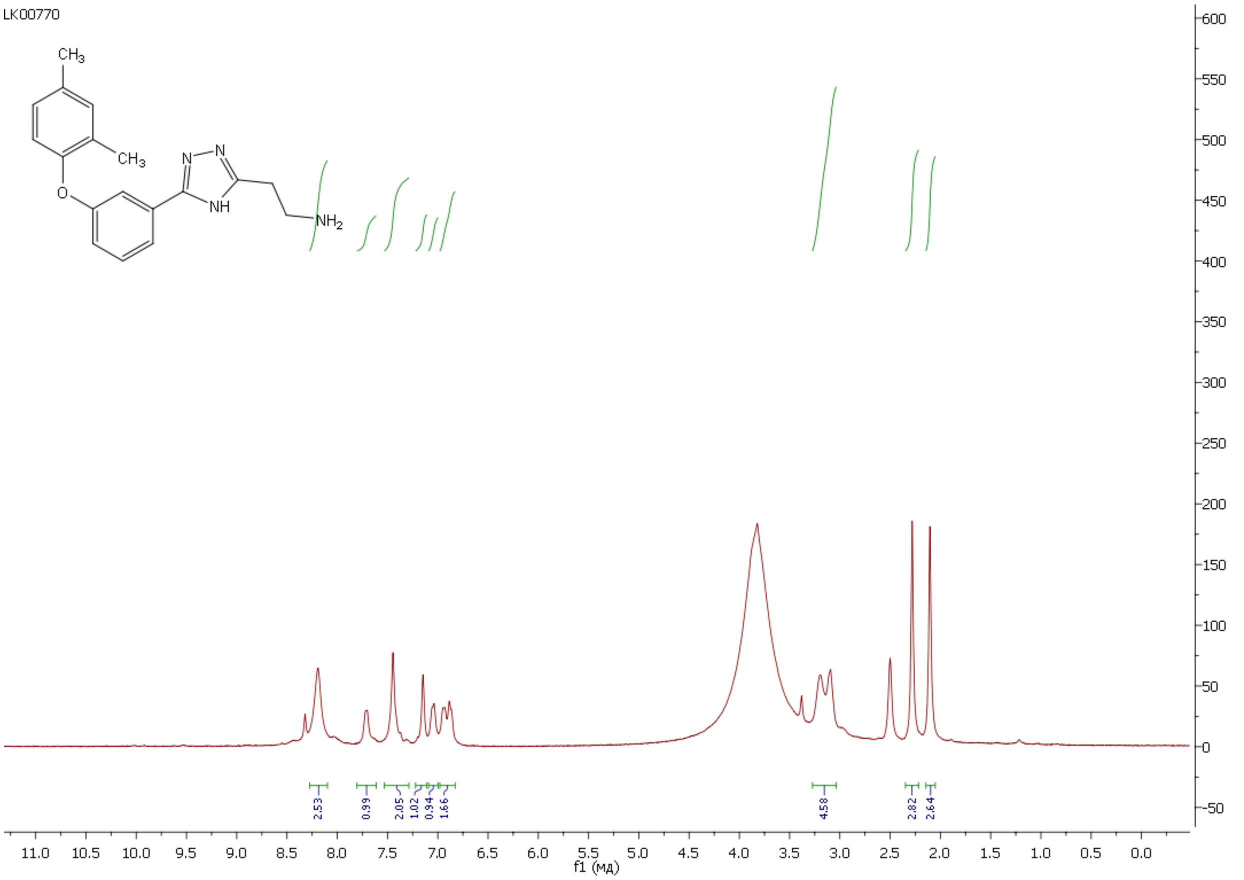

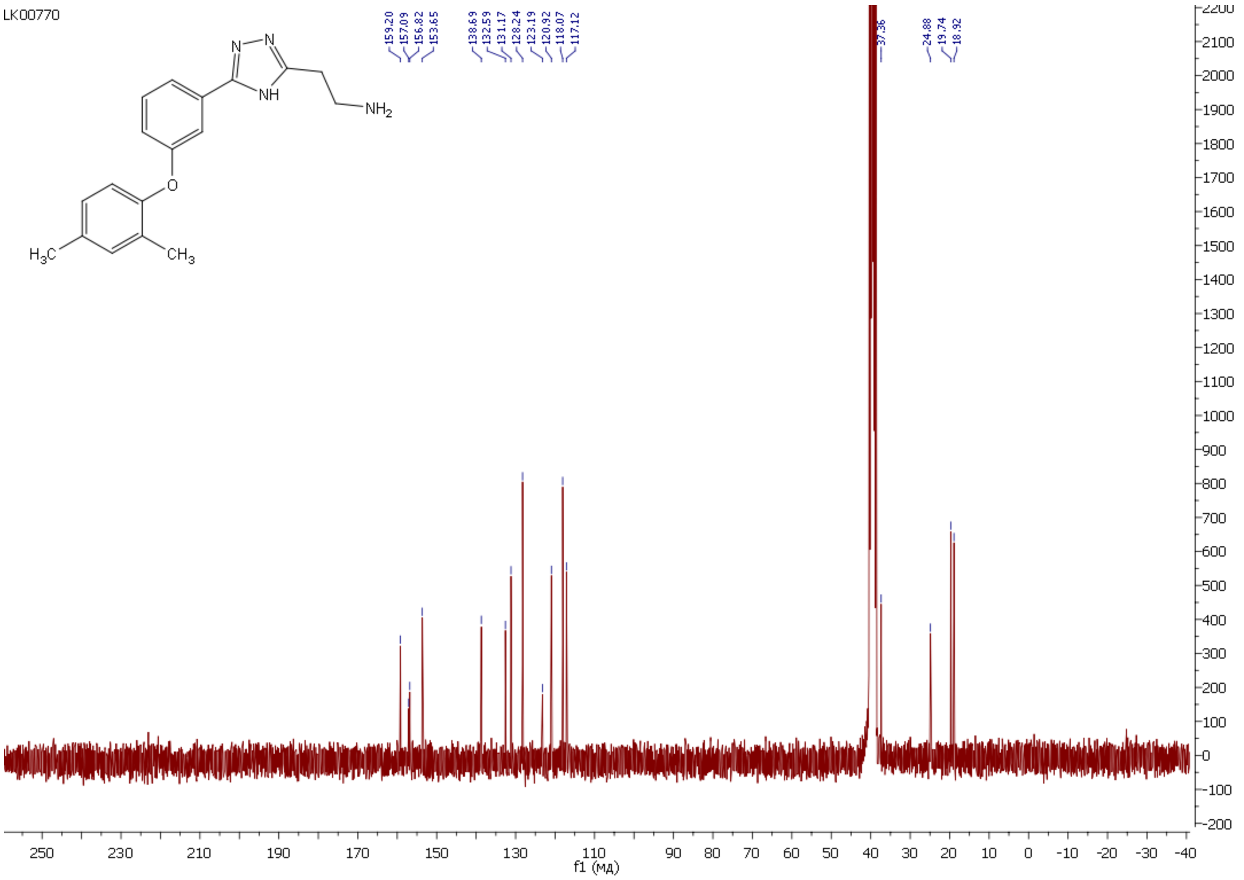

^{1}H and ^{13}C NMR spectra of compound **53**

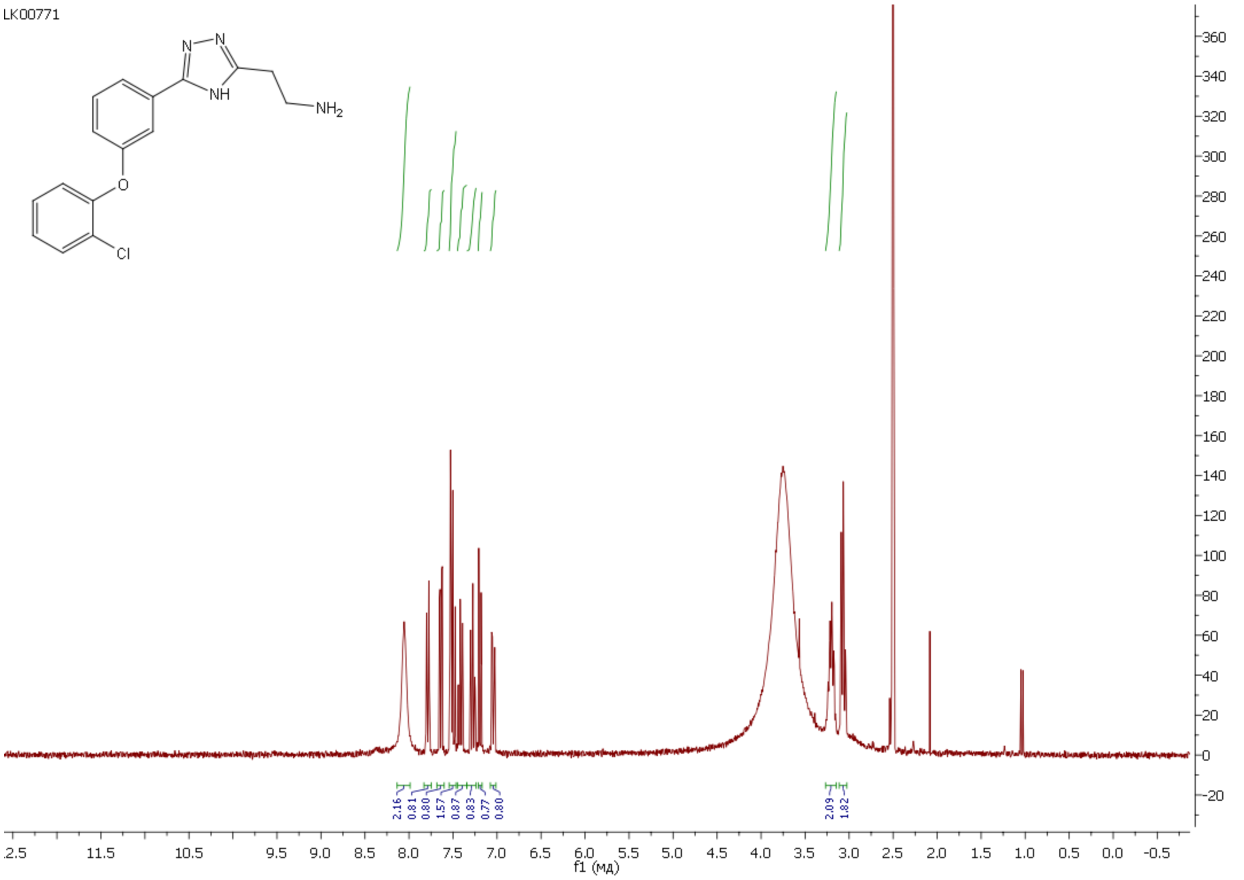

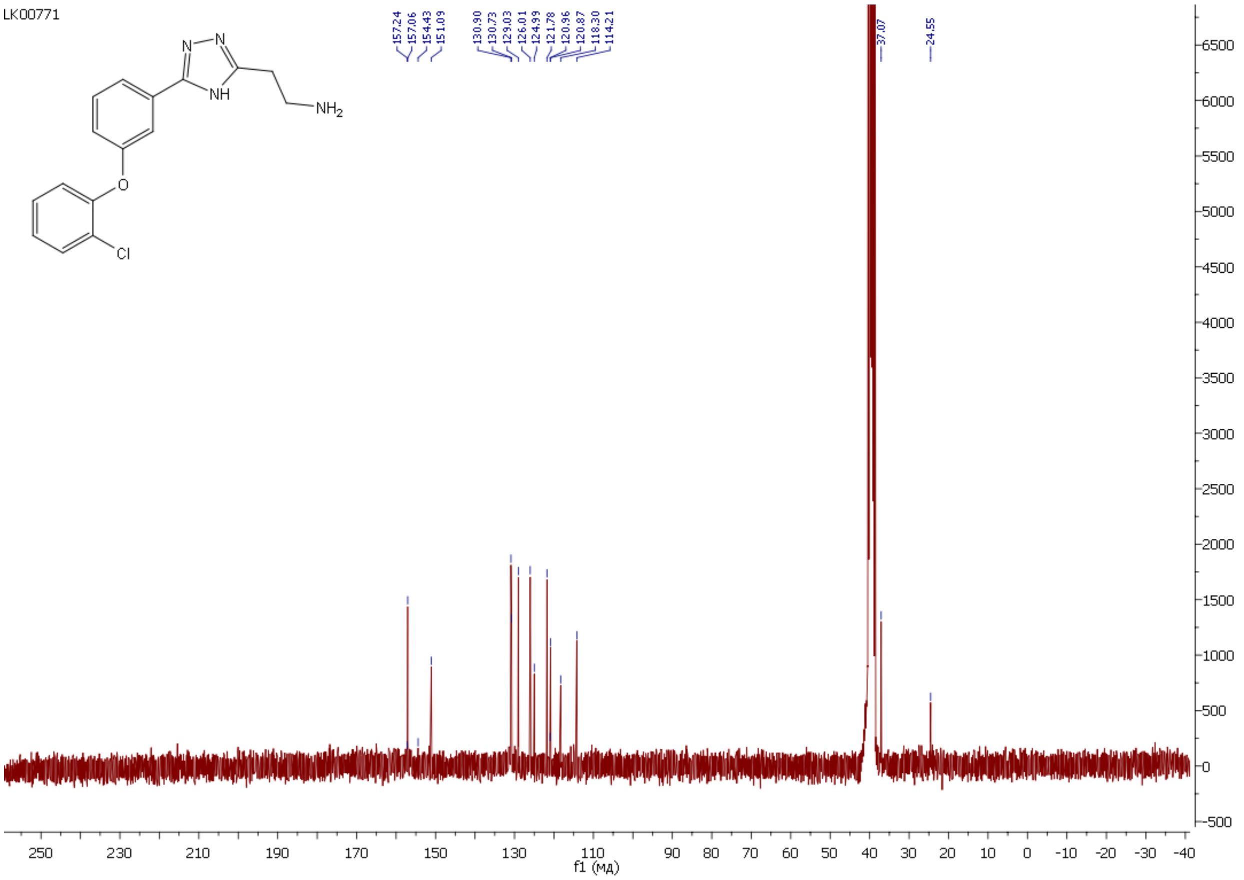

^{1}H and ^{13}C NMR spectra of compound **54**

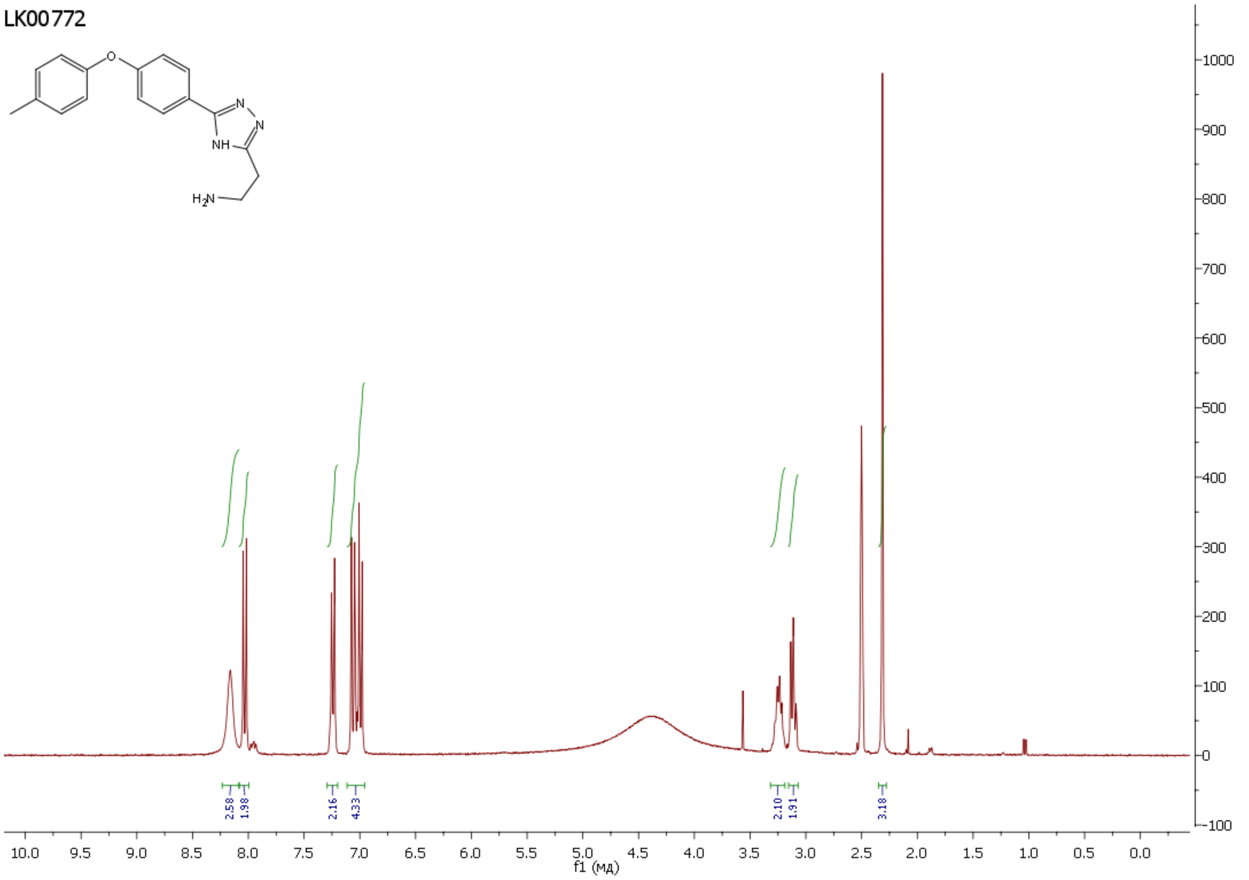

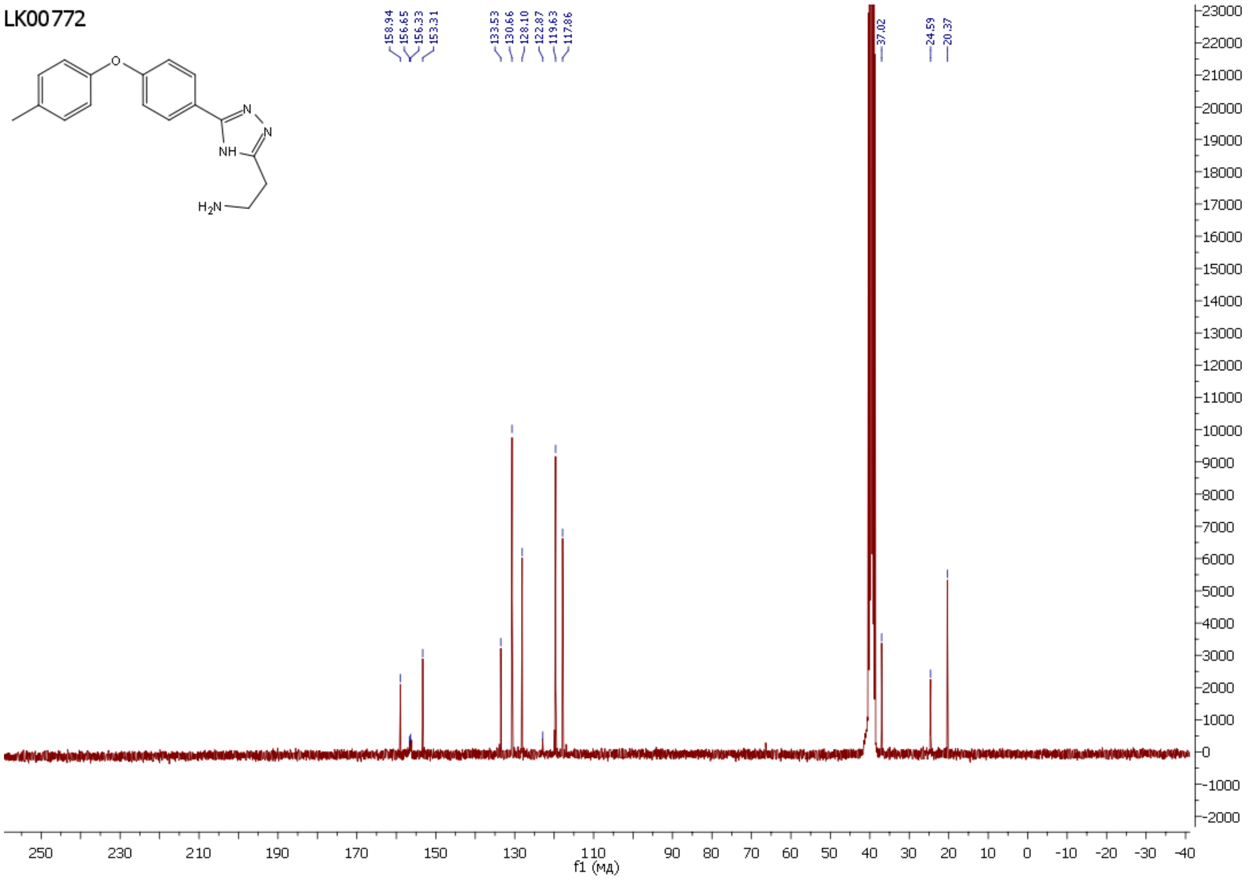

^{1}H and ^{13}C NMR spectra of compound **55**

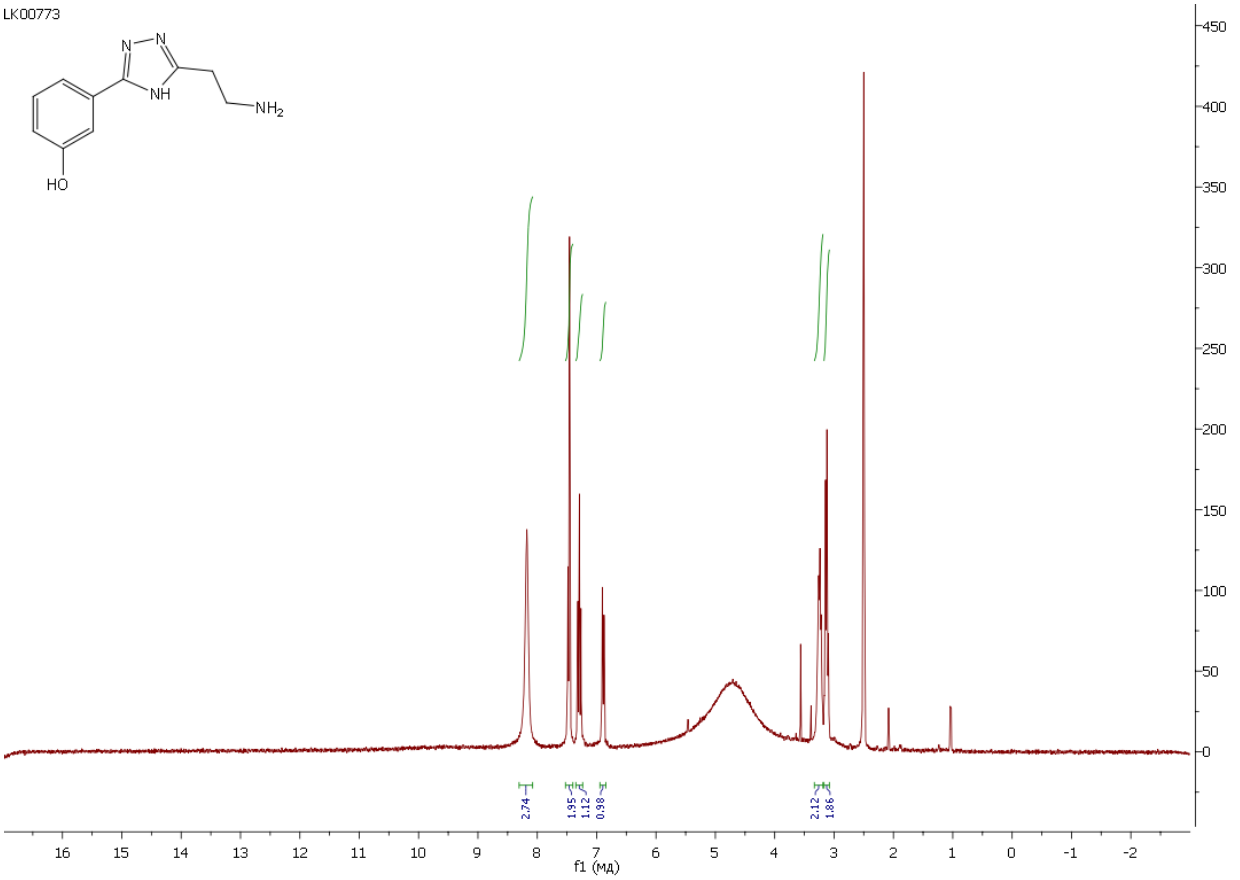

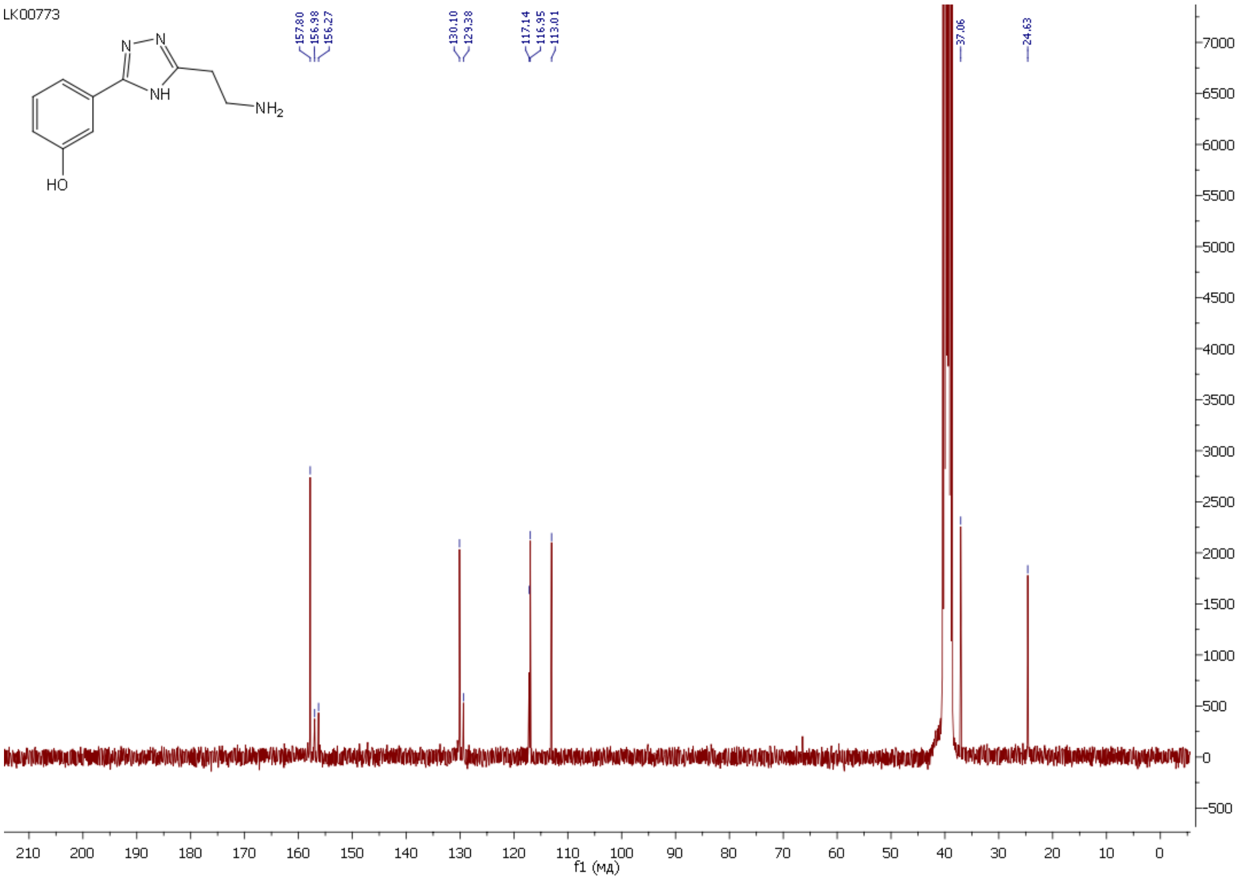

^{1}H and ^{13}C NMR spectra of compound **56**

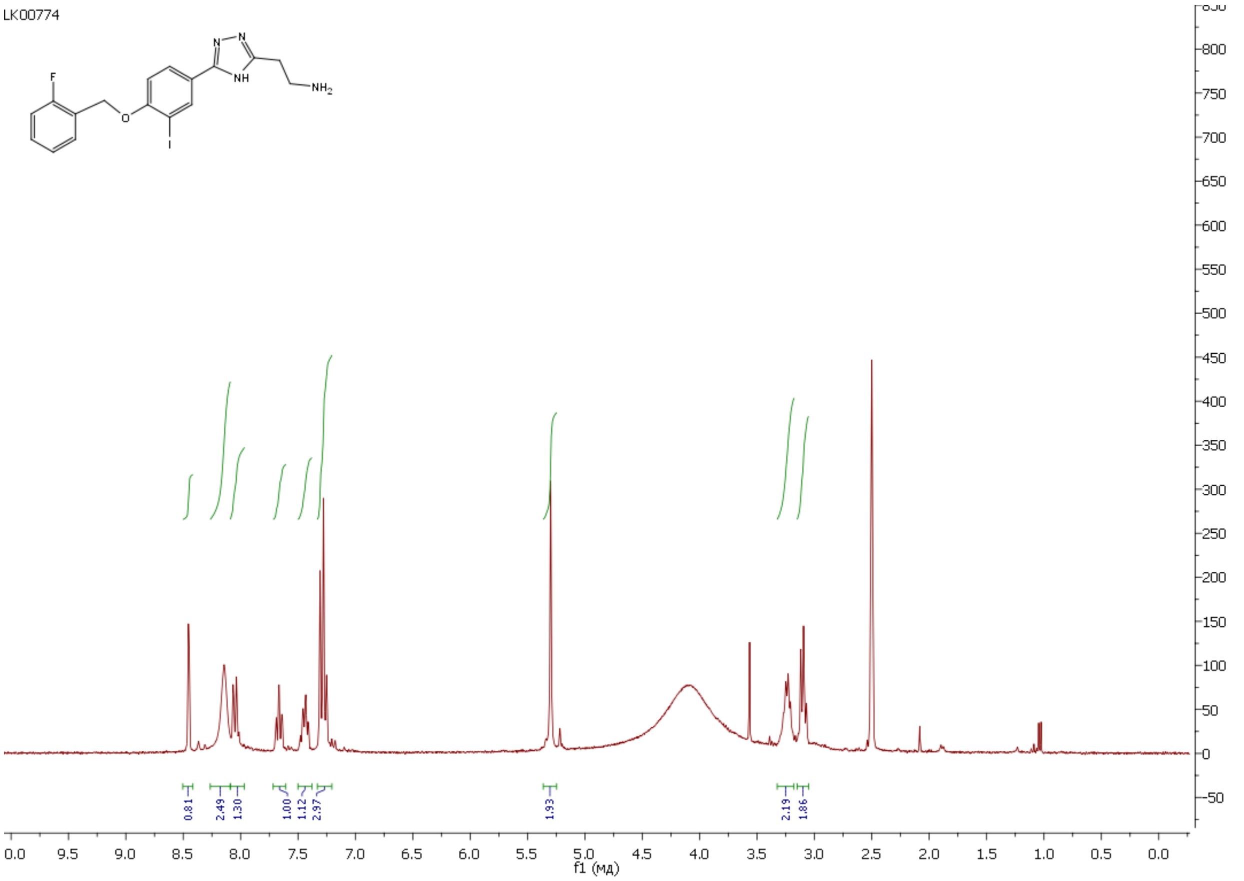

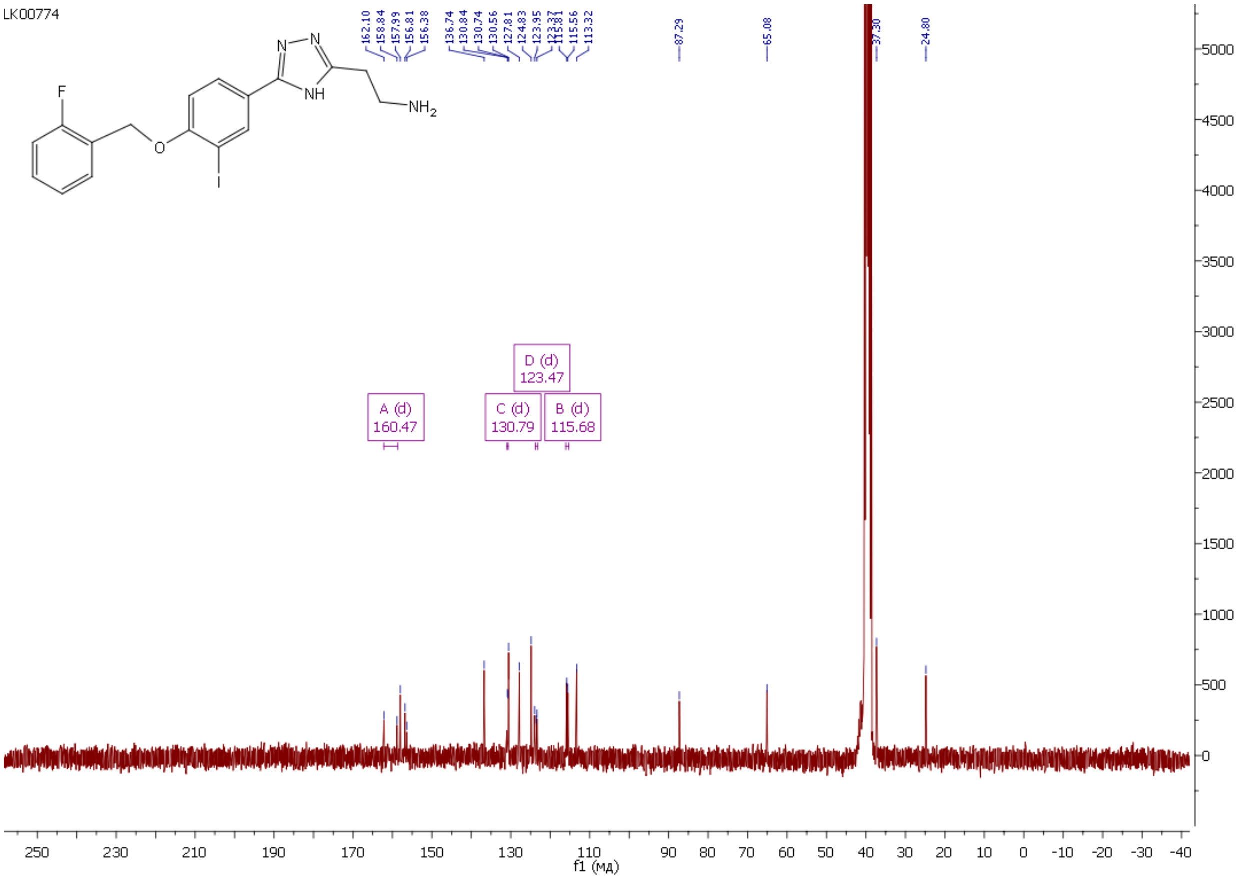

^{1}H and ^{13}C NMR spectra of compound **57**

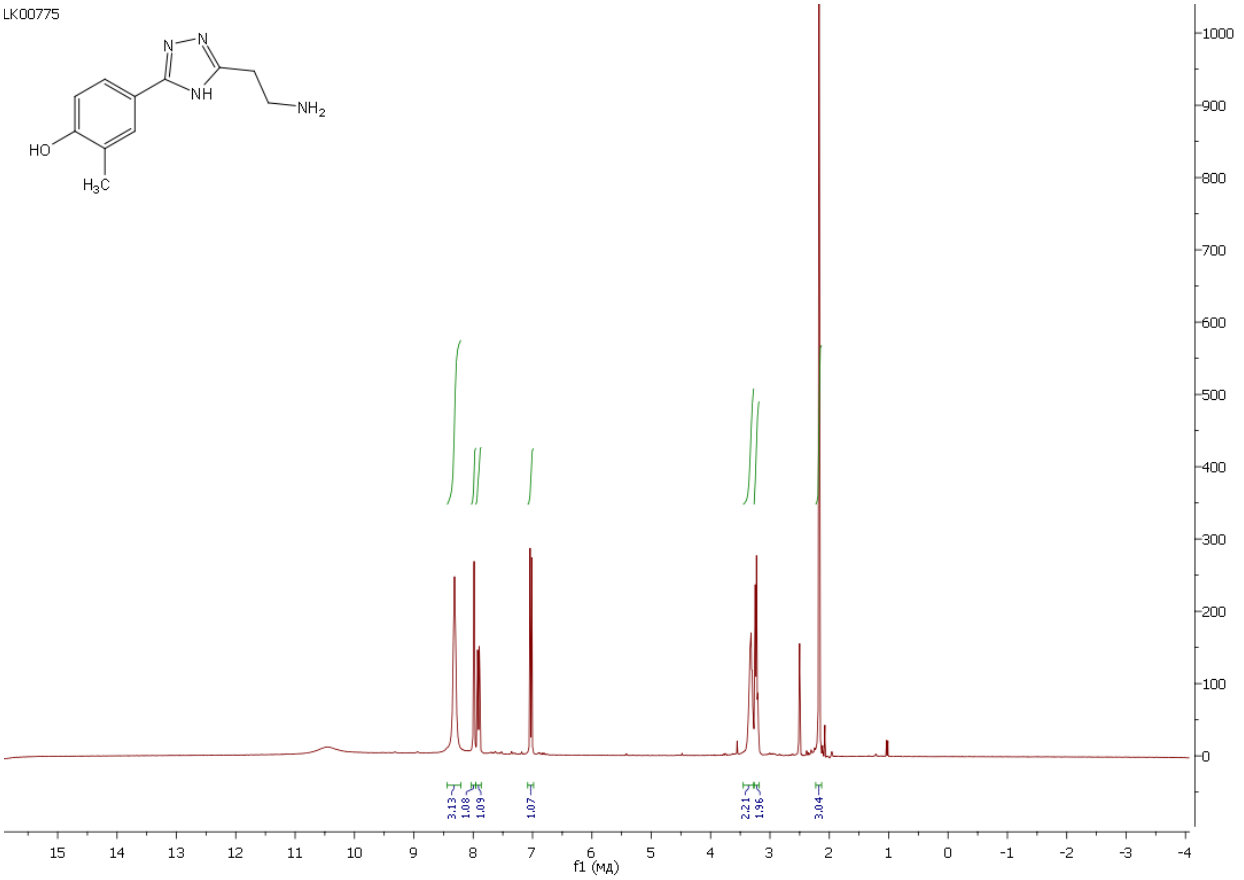

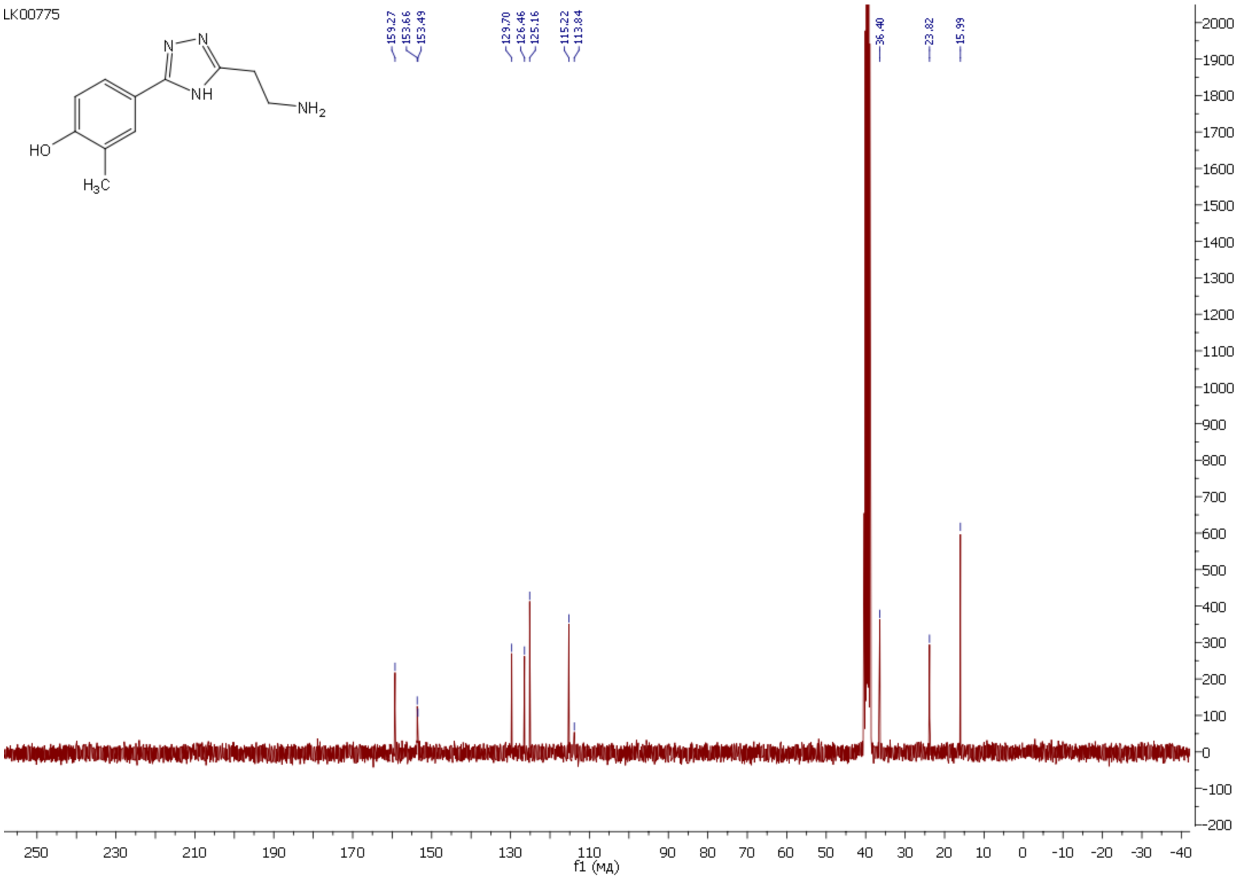

^{1}H and ^{13}C NMR spectra of compound **58**

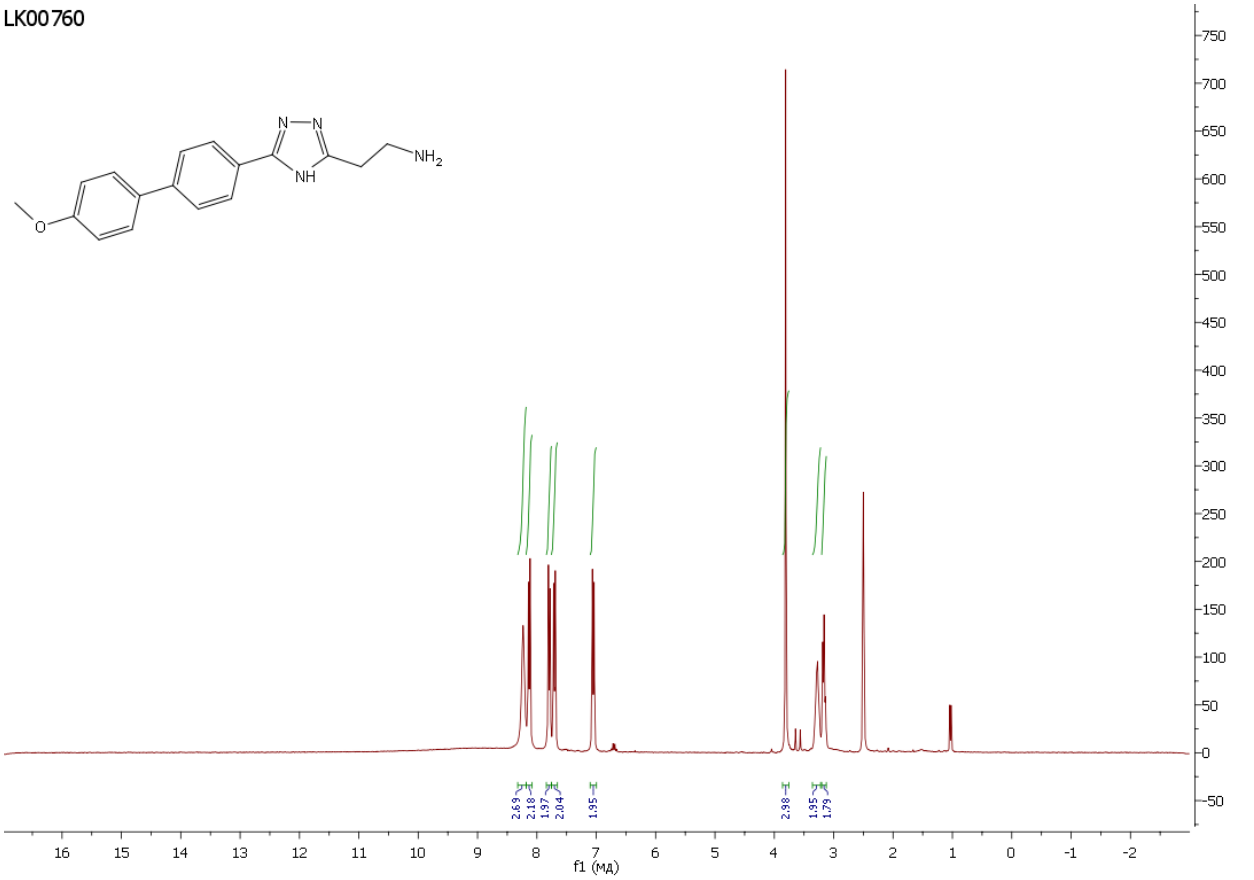

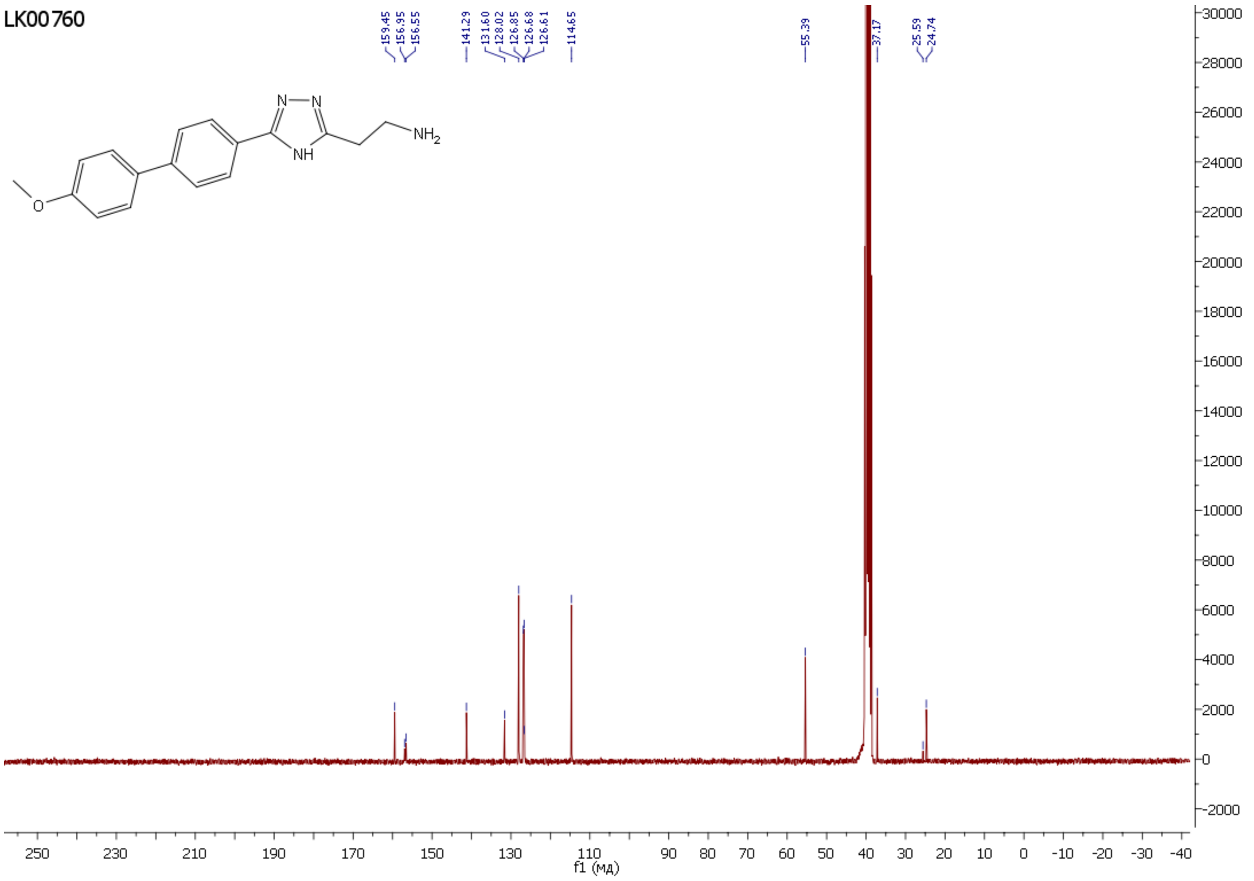

^{1}H and ^{13}C NMR spectra of compound **59**

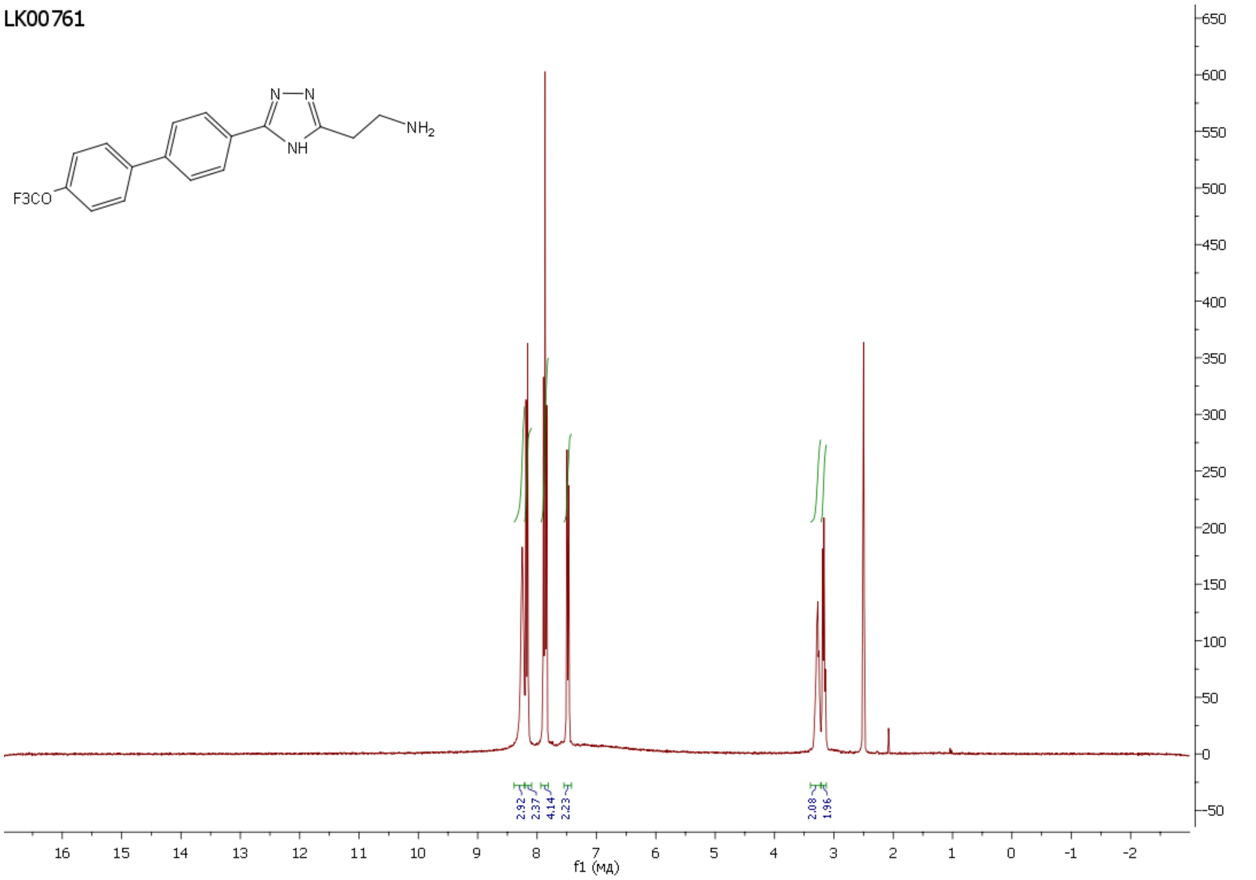

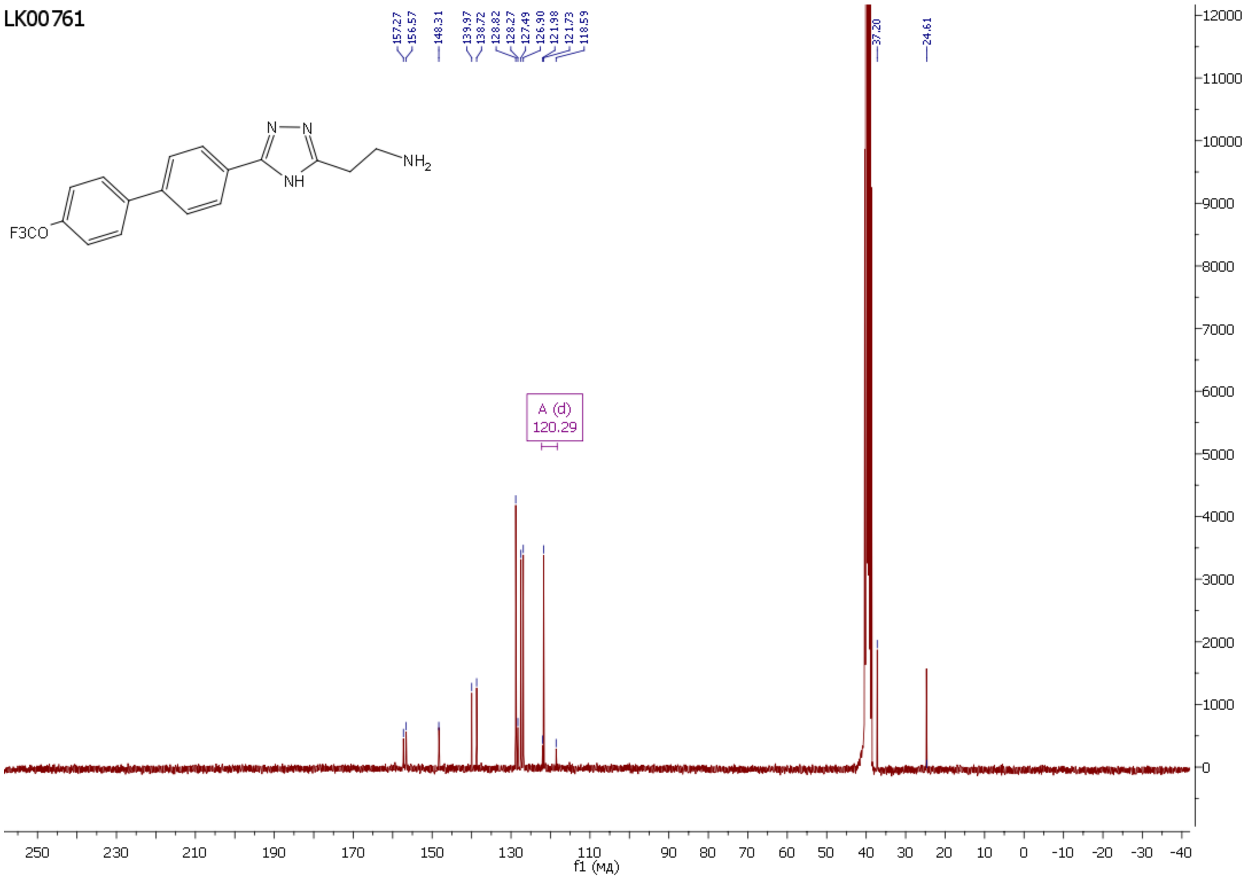

^{1}H and ^{13}C NMR spectra of compound **60**

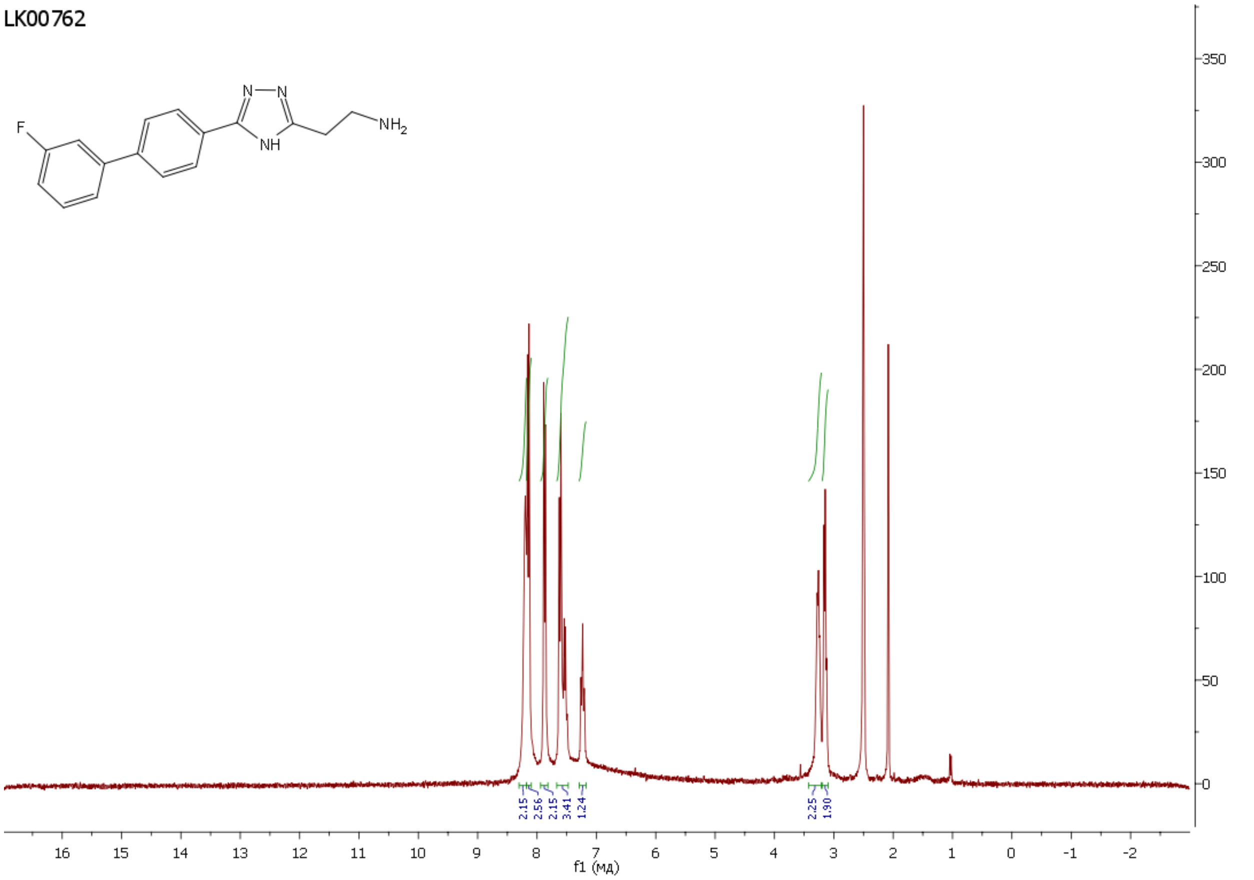

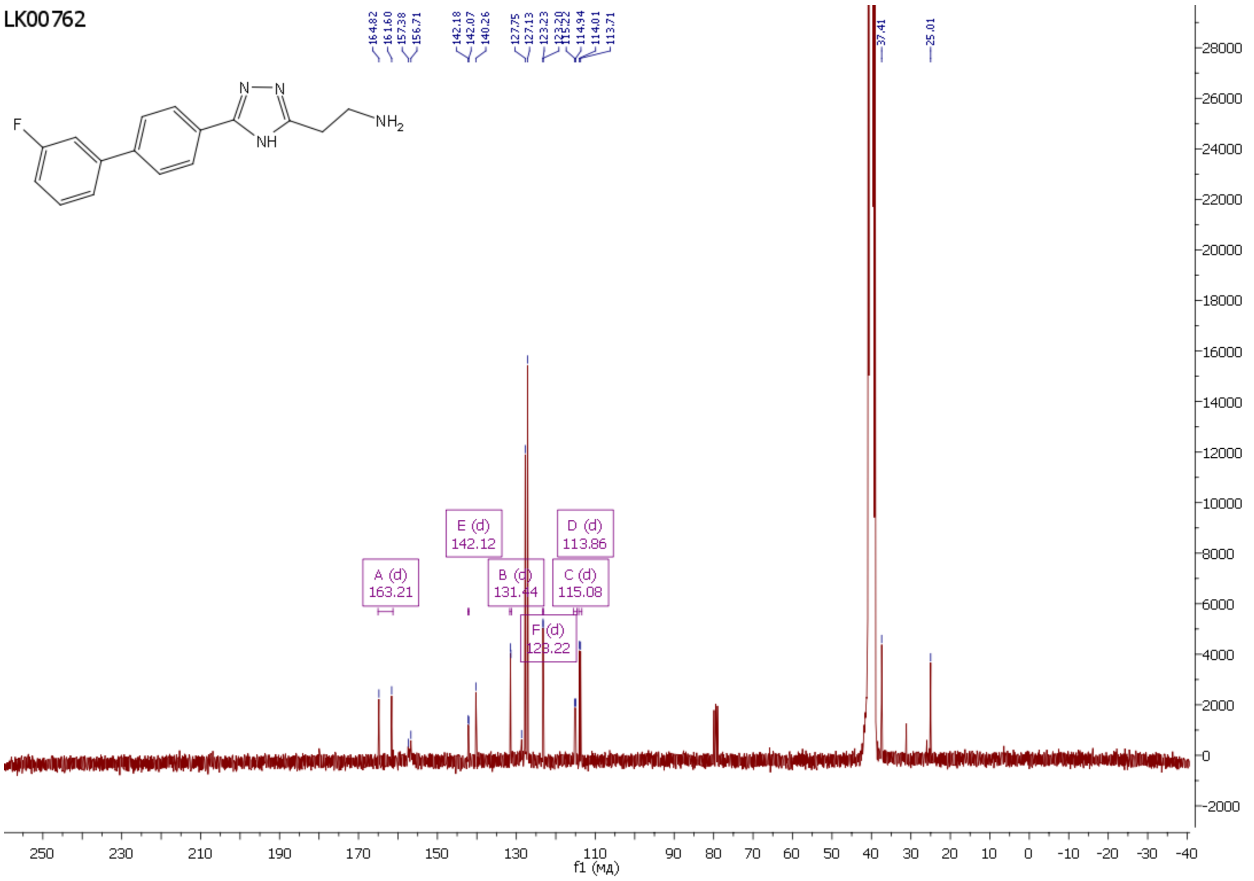

^{1}H and ^{13}C NMR spectra of compound **61**

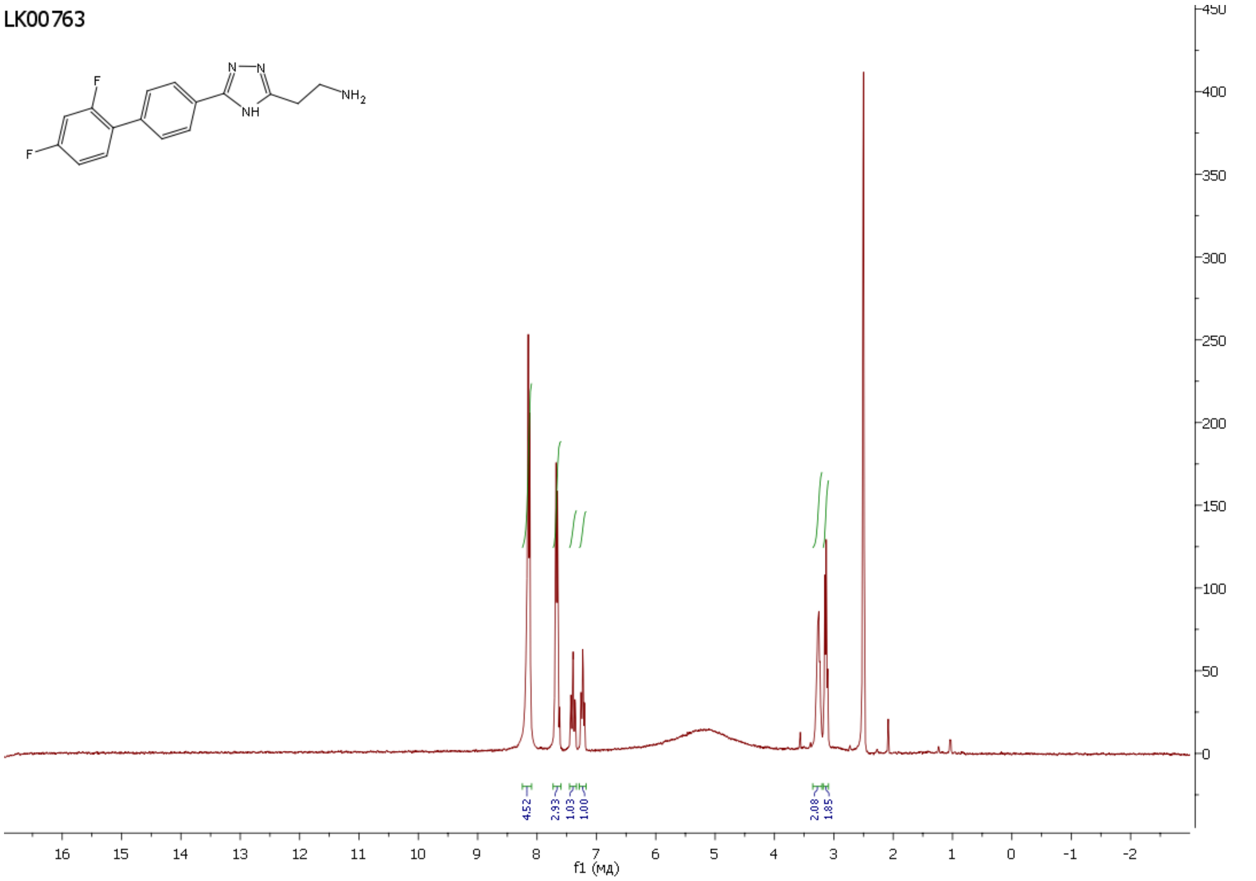

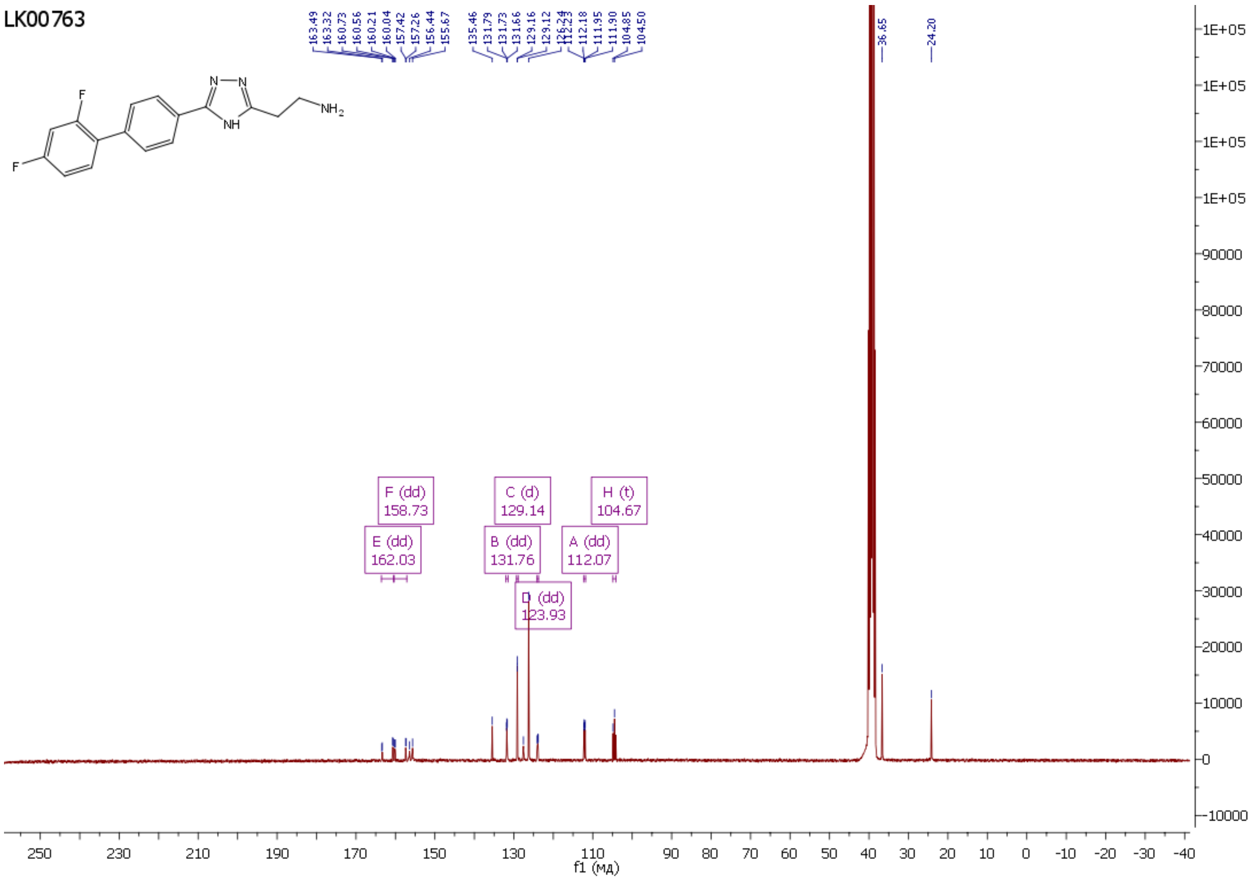

^{1}H and ^{13}C NMR spectra of compound **62**

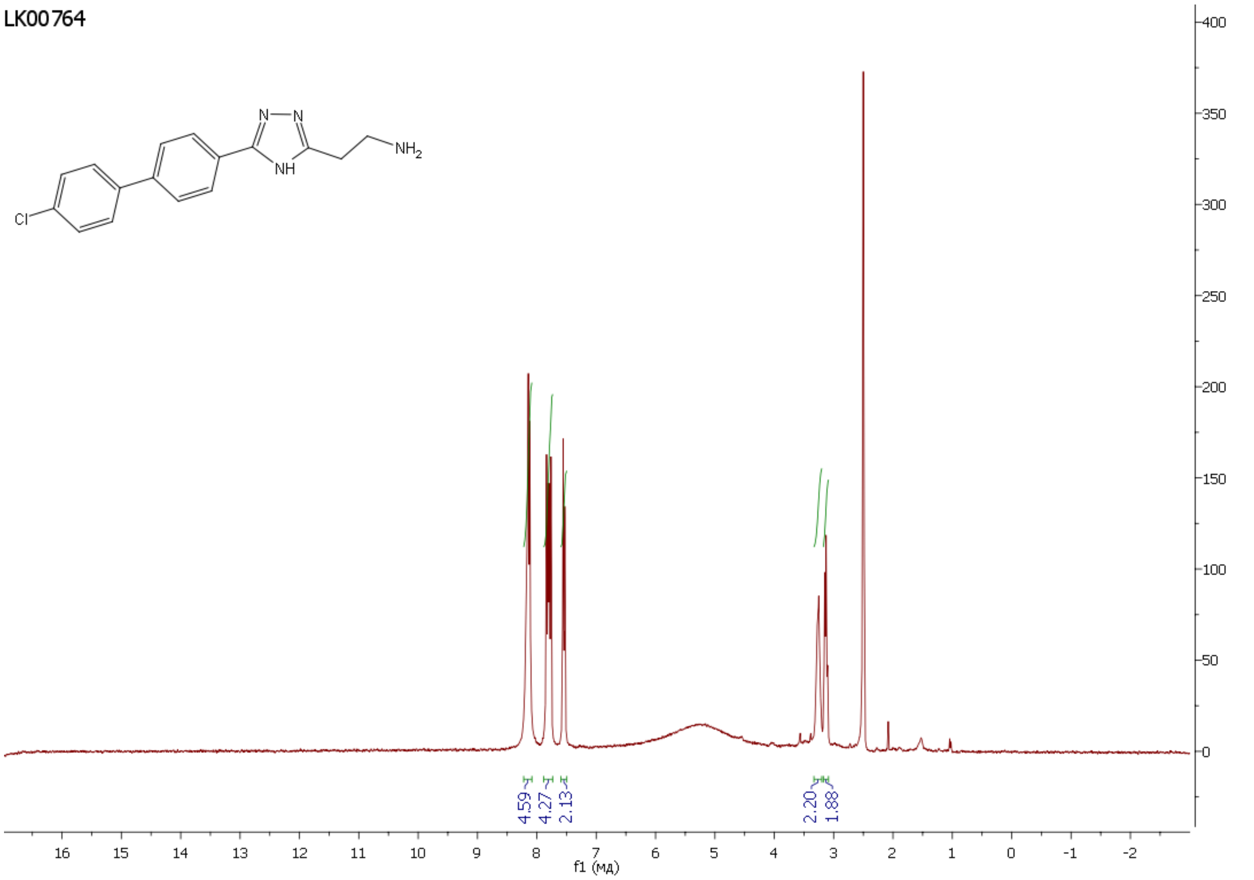

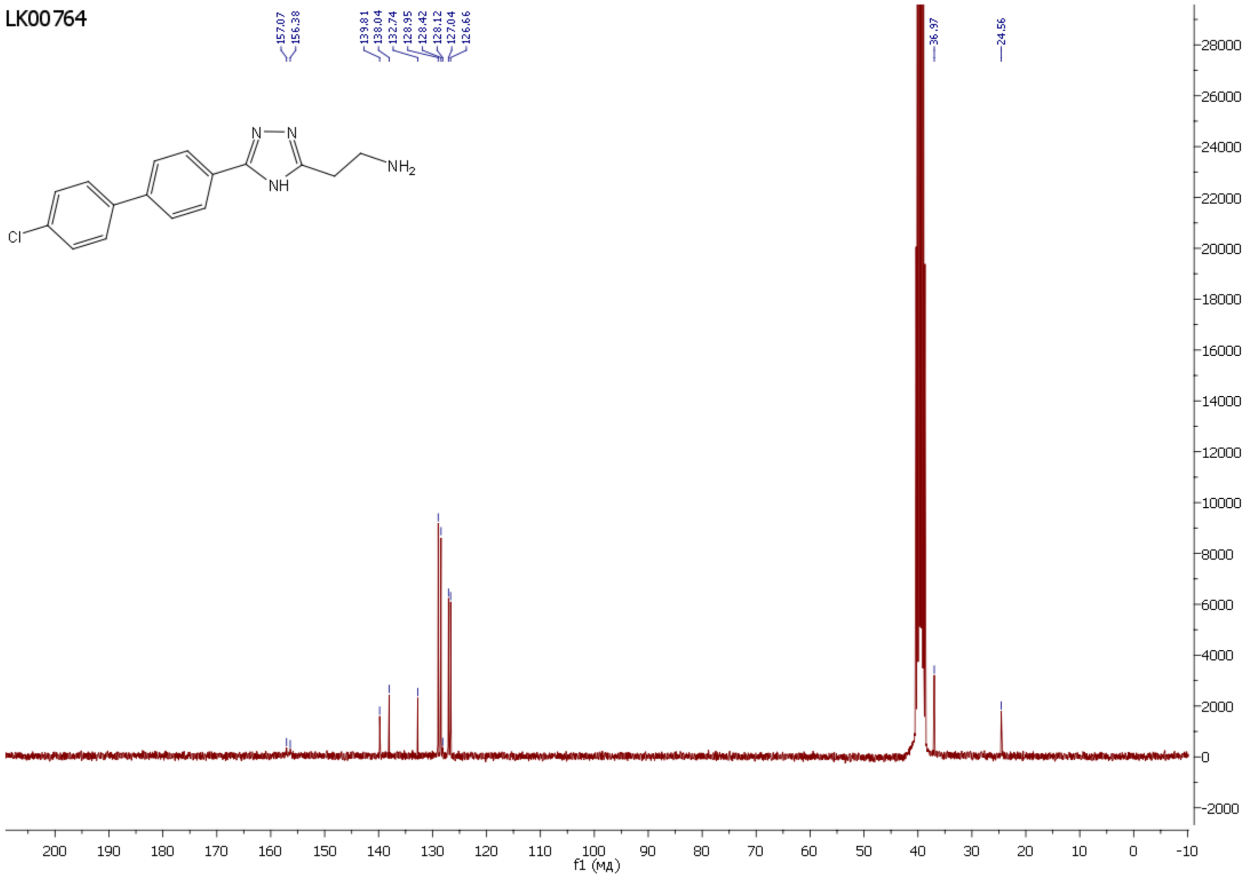

^{1}H and ^{13}C NMR spectra of compound **63**

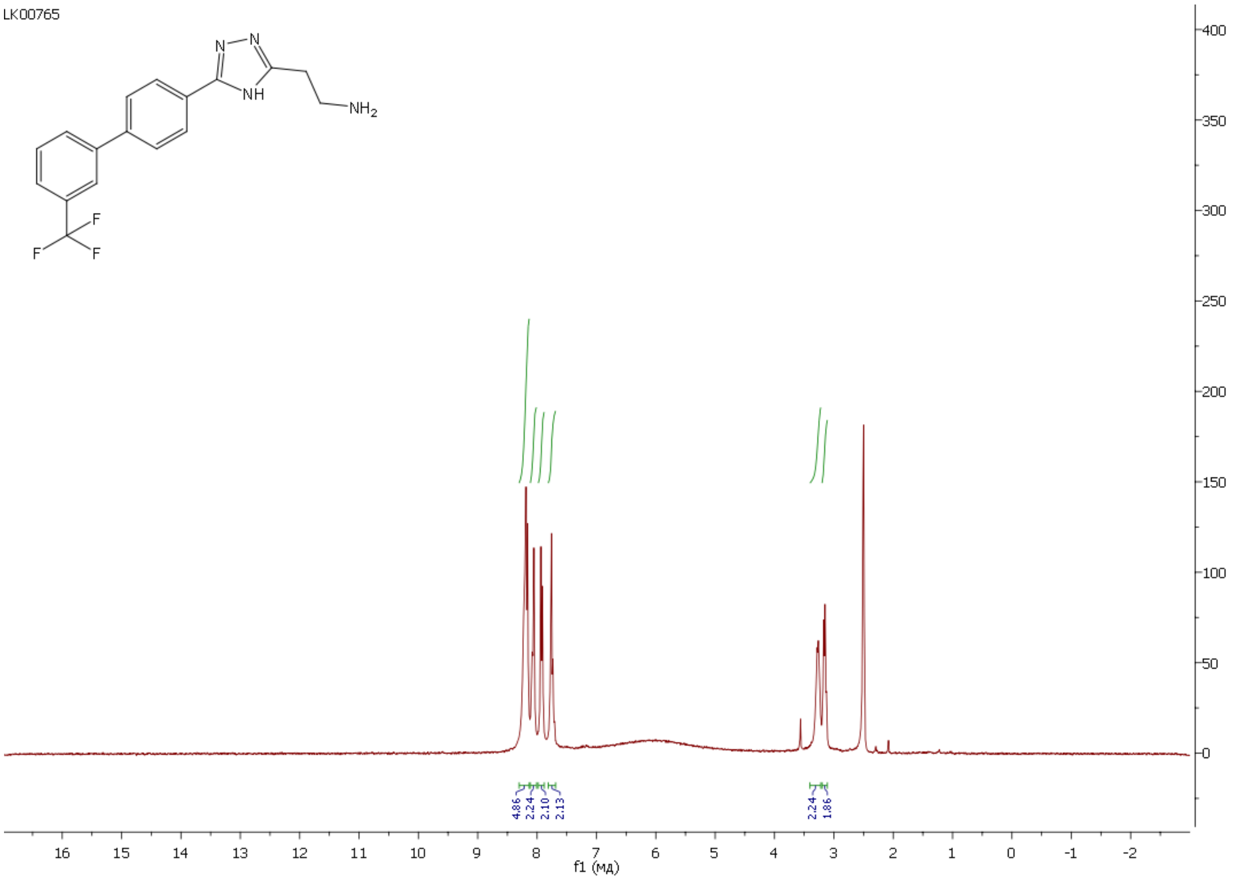

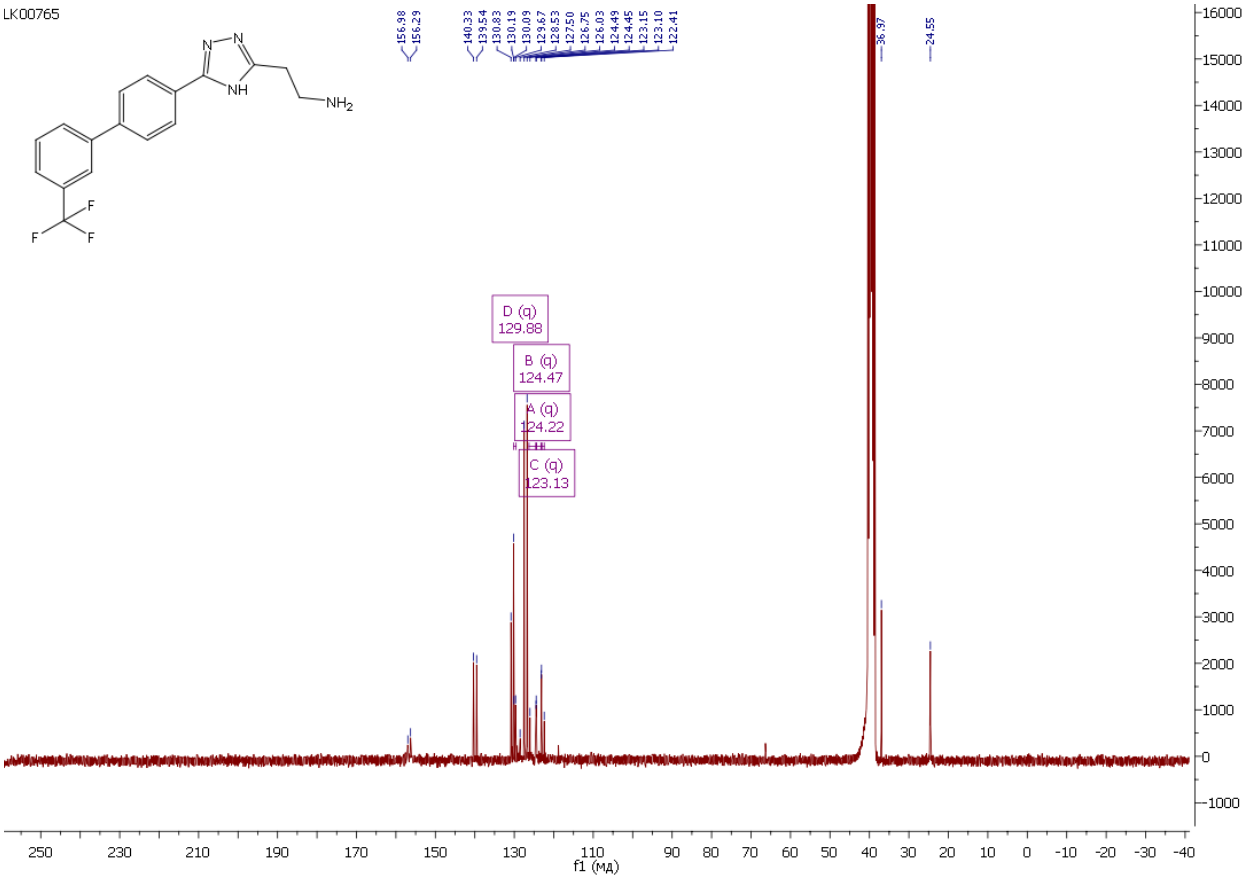

^{1}H and ^{13}C NMR spectra of compound **64**

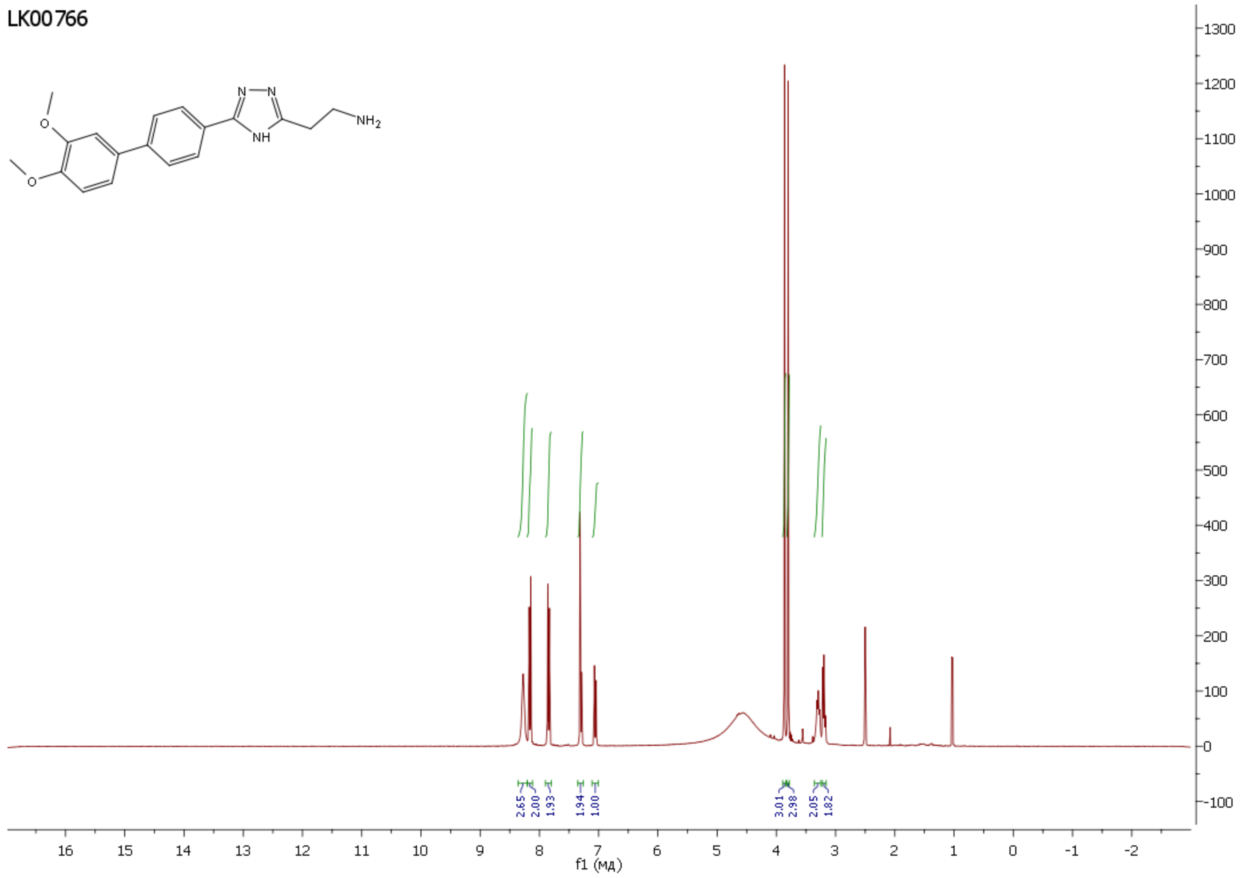

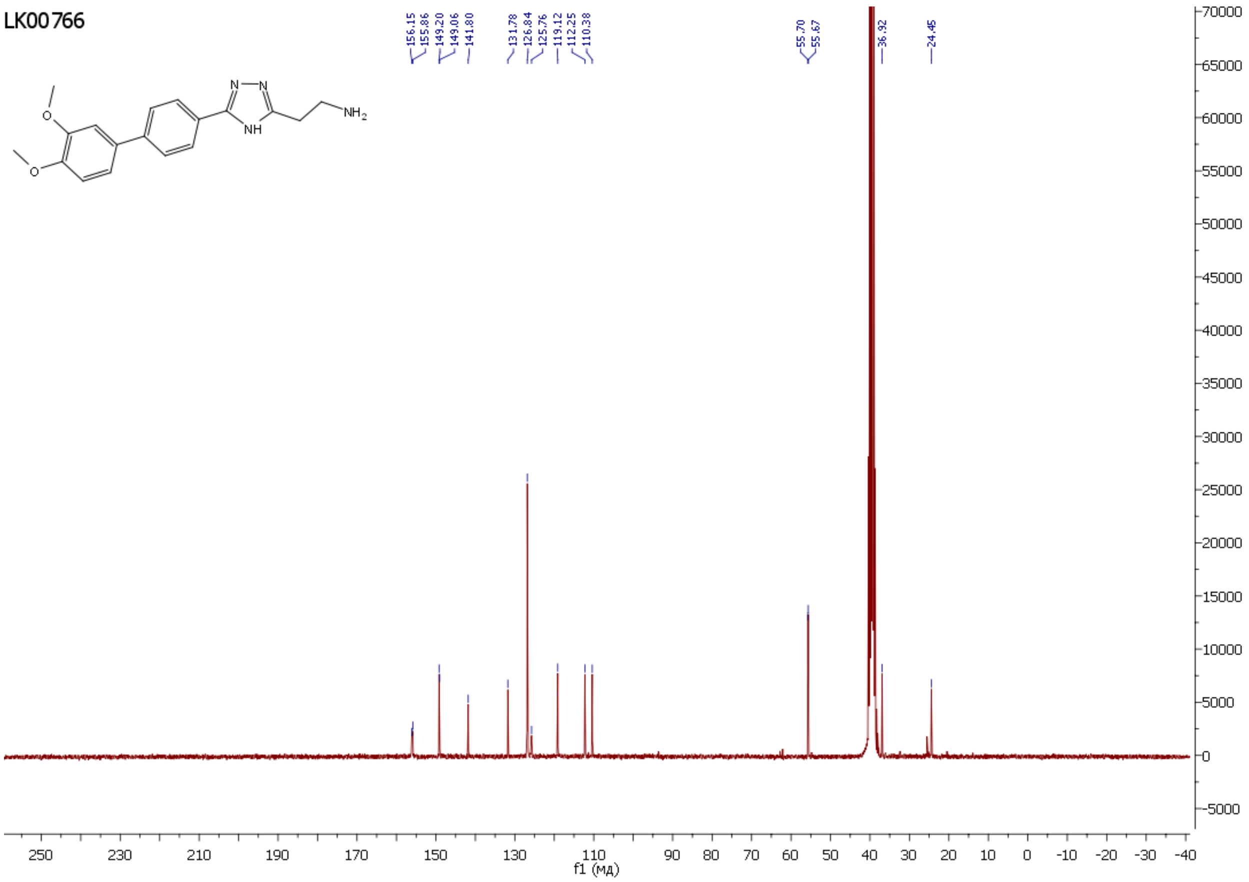

^{1}H and ^{13}C NMR spectra of compound **65**

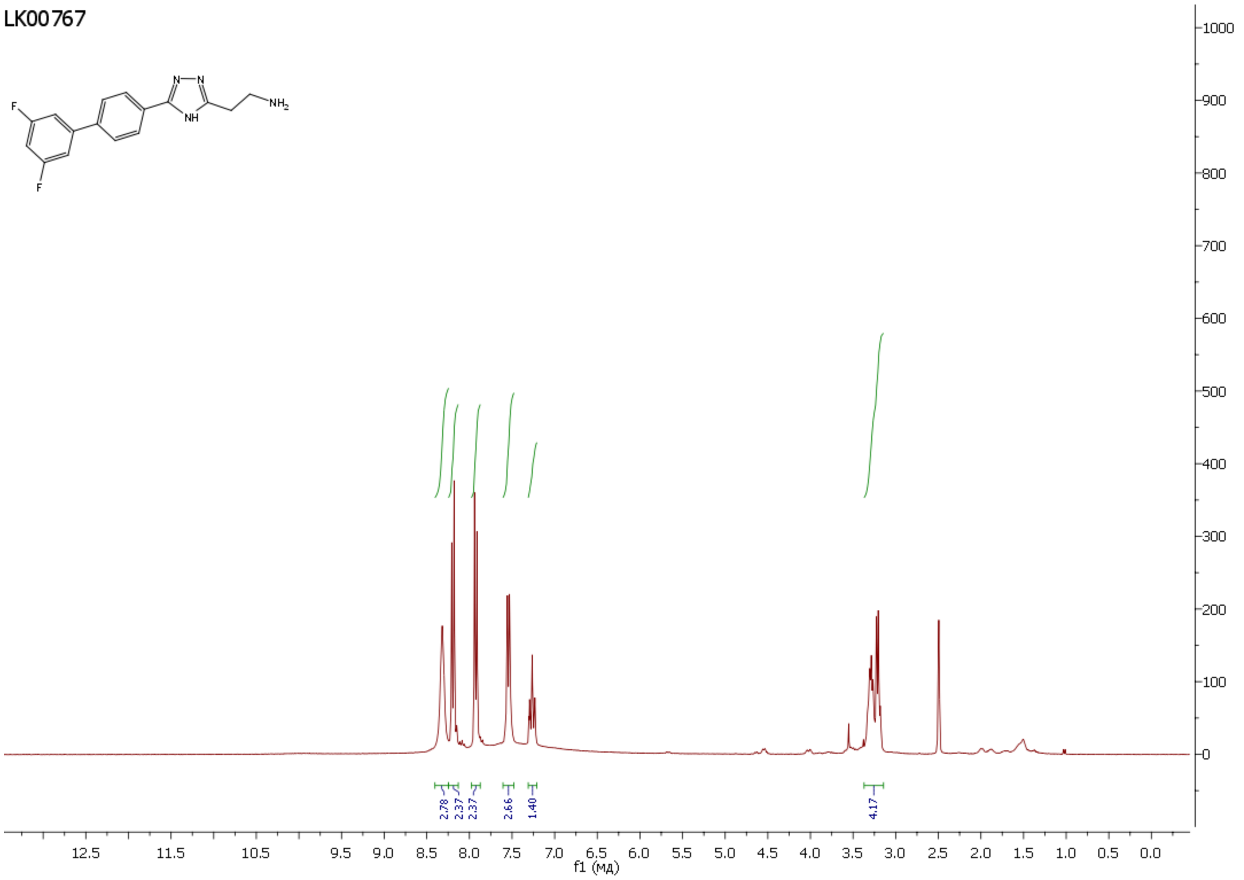

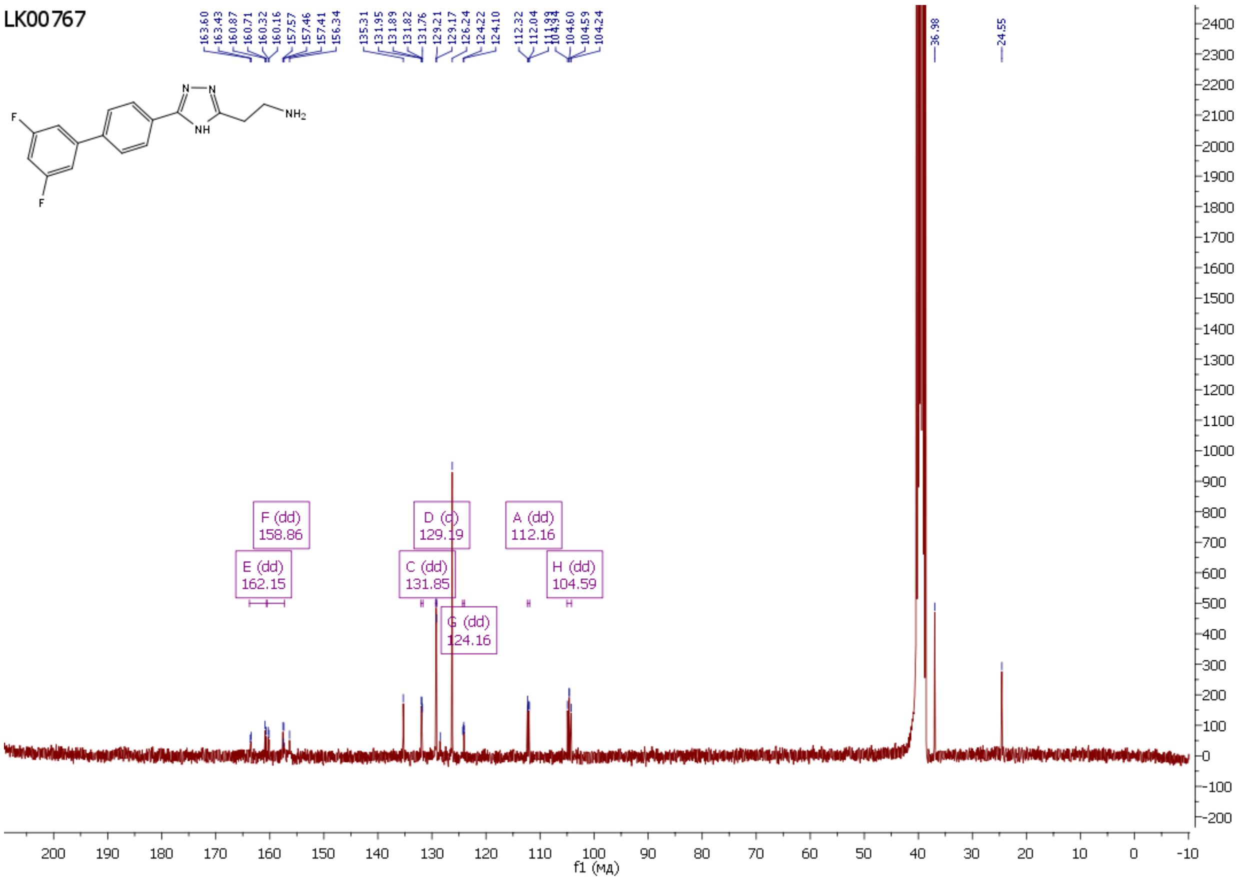

^{1}H and ^{13}C NMR spectra of compound **66**

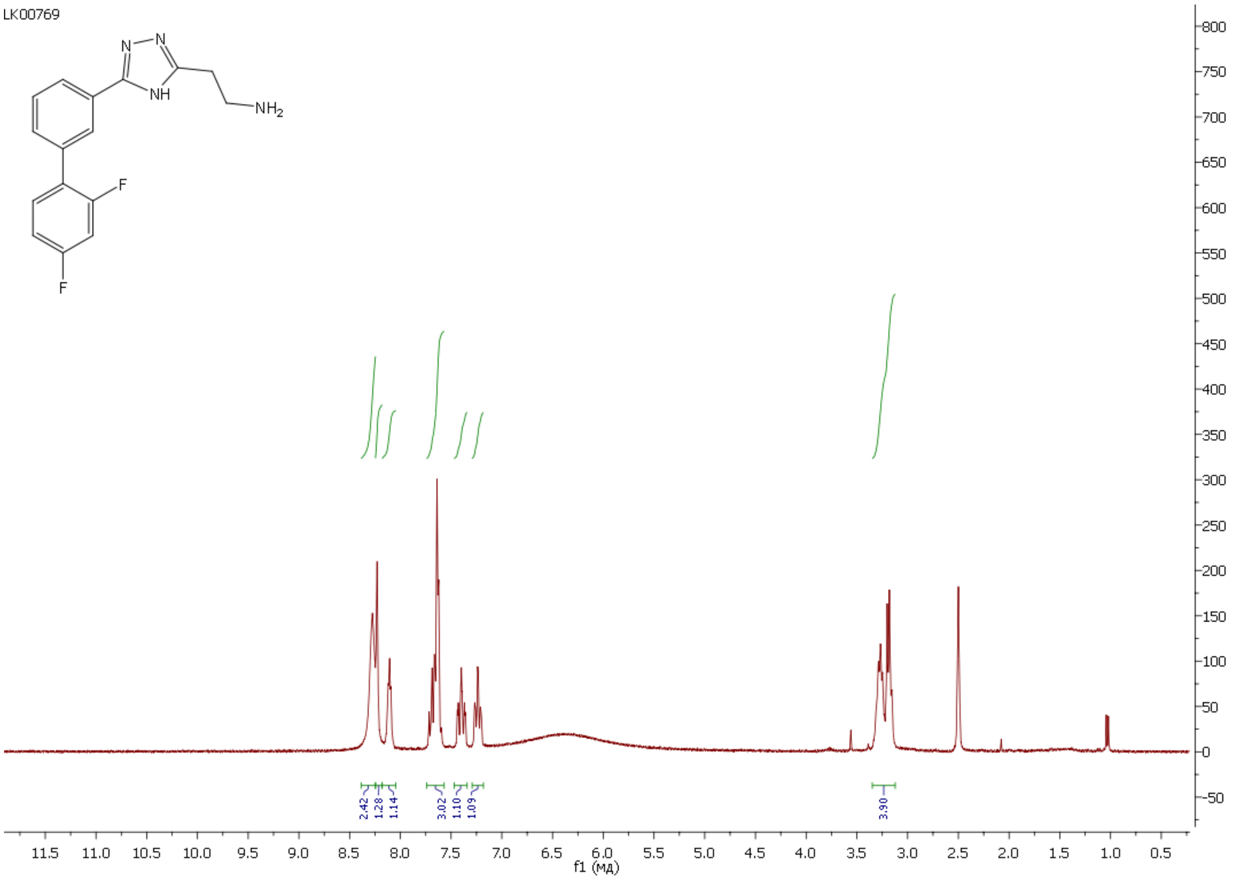

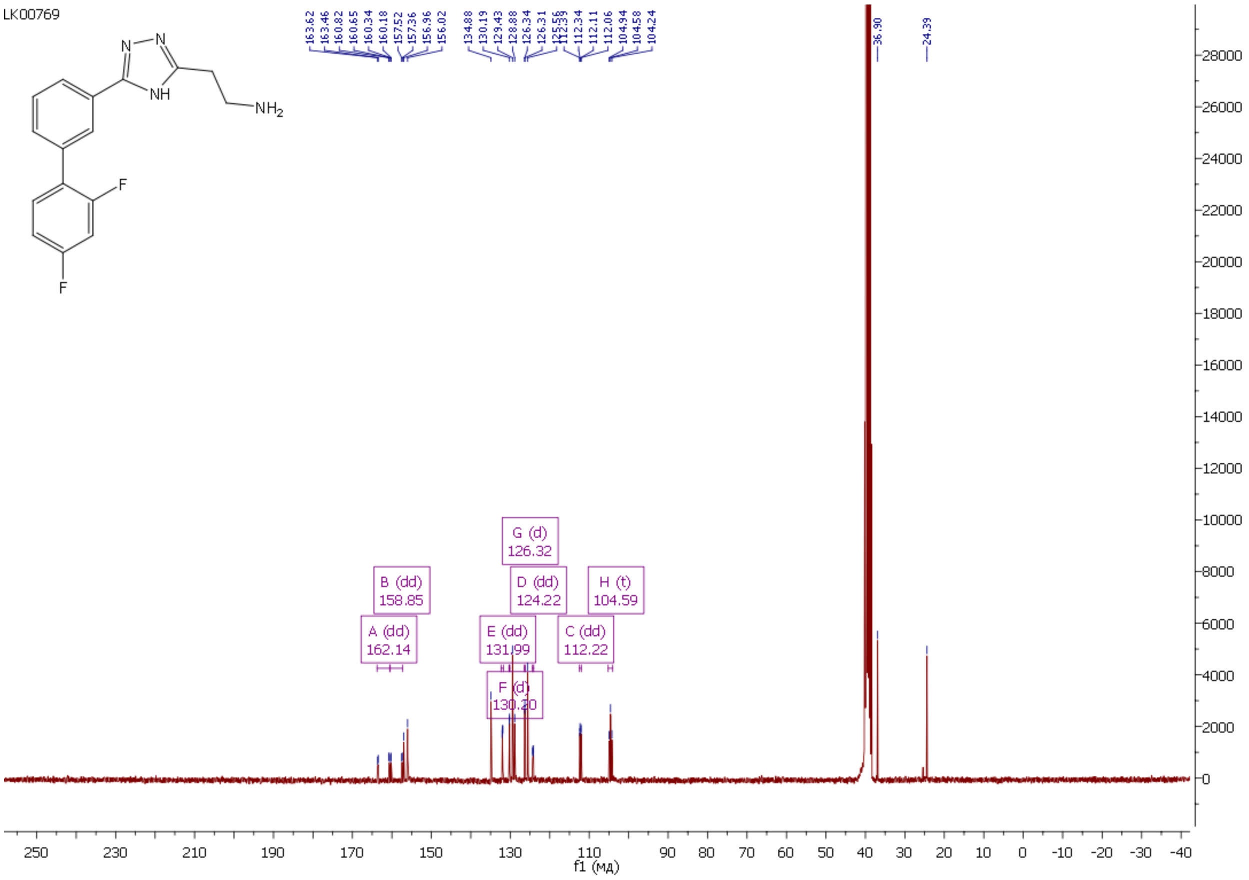

^{1}H and ^{13}C NMR spectra of compound **67**

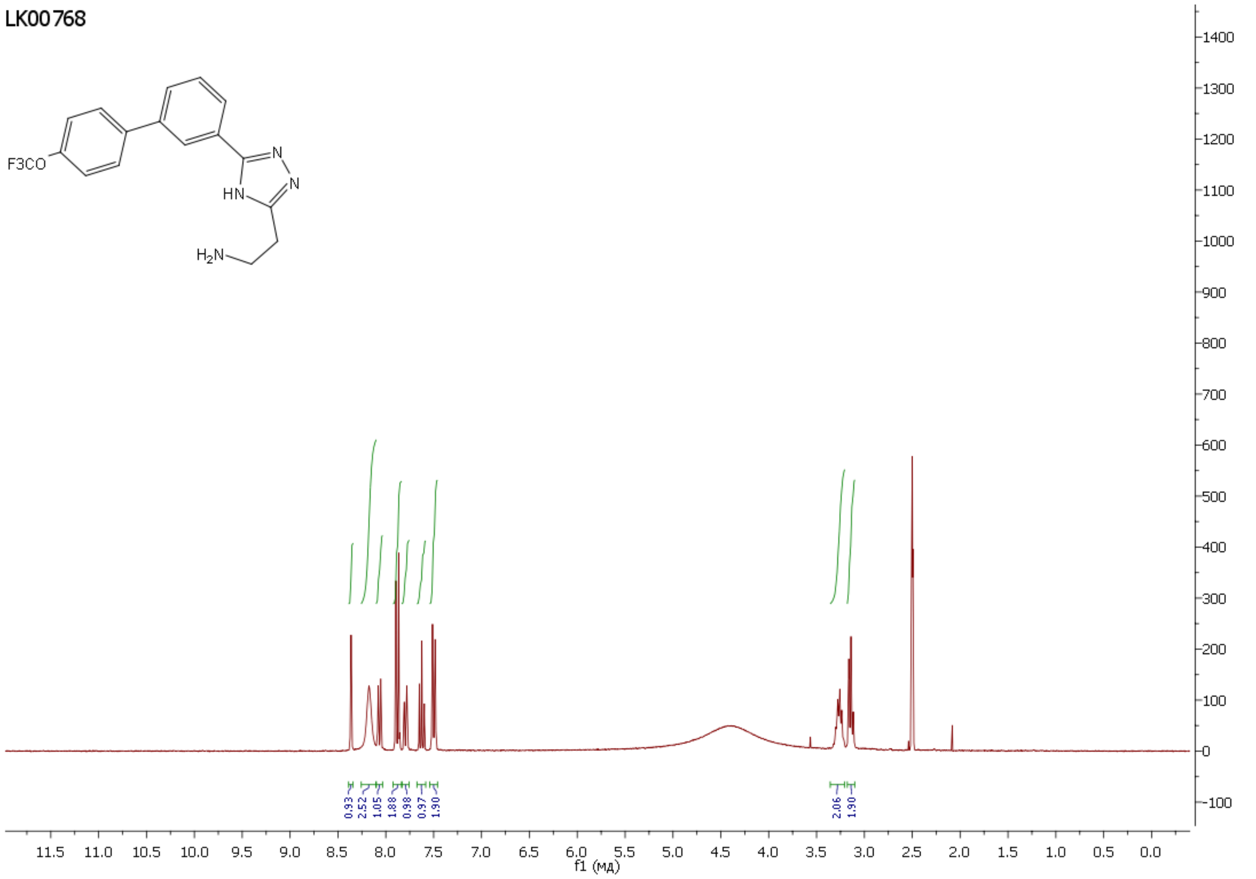

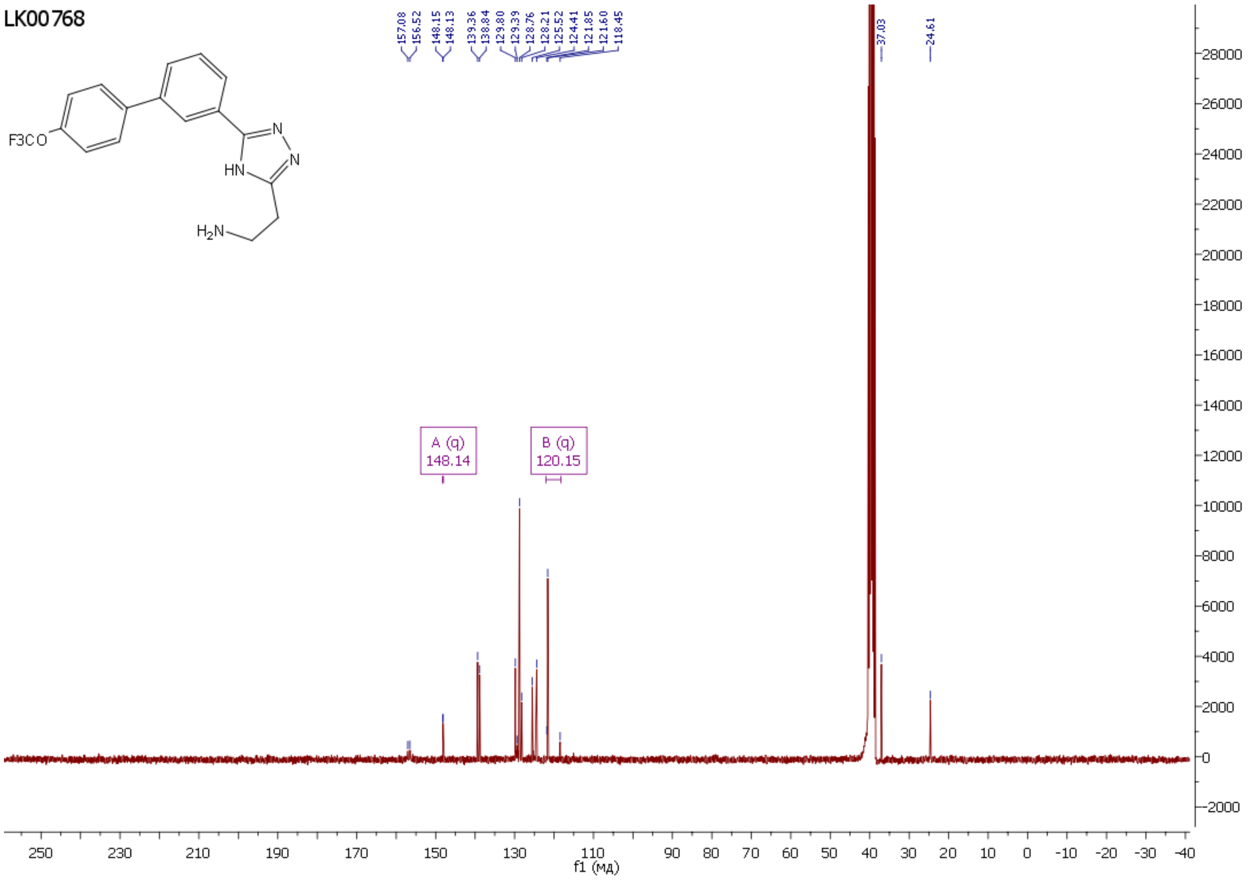

Supplement: Supplementary file 1 [file biomolecules-12-01650-s001.zip › biomolecules-1982916-supplementary.pdf]
